# Chirality- and sequence-selective successive self-sorting via specific homo- and complementary-duplex formations

**DOI:** 10.1038/ncomms8236

**Published:** 2015-06-08

**Authors:** Wataru Makiguchi, Junki Tanabe, Hidekazu Yamada, Hiroki Iida, Daisuke Taura, Naoki Ousaka, Eiji Yashima

**Affiliations:** 1 Department of Molecular Design and Engineering, Graduate School of Engineering, Nagoya University, Chikusa-ku, Nagoya 464-8603, Japan

## Abstract

Self-recognition and self-discrimination within complex mixtures are of fundamental importance in biological systems, which entirely rely on the preprogrammed monomer sequences and homochirality of biological macromolecules. Here we report artificial chirality- and sequence-selective successive self-sorting of chiral dimeric strands bearing carboxylic acid or amidine groups joined by chiral amide linkers with different sequences through homo- and complementary-duplex formations. A mixture of carboxylic acid dimers linked by *racemic*-1,2-cyclohexane *bis*-amides with different amide sequences (NHCO or CONH) self-associate to form homoduplexes in a completely sequence-selective way, the structures of which are different from each other depending on the linker amide sequences. The further addition of an enantiopure amide-linked amidine dimer to a mixture of the racemic carboxylic acid dimers resulted in the formation of a single optically pure complementary duplex with a 100% diastereoselectivity and complete sequence specificity stabilized by the amidinium–carboxylate salt bridges, leading to the perfect chirality- and sequence-selective duplex formation.

## Abstract (web summary)

The recognition and self-sorting of chiral molecules is a vital feature of many biomolecules. Here, the authors report chirality- and sequence-specific self-sorting of organic strands containing carboxylic acid or amidine groups, leading to selective duplex formation.

## Graphical abstract

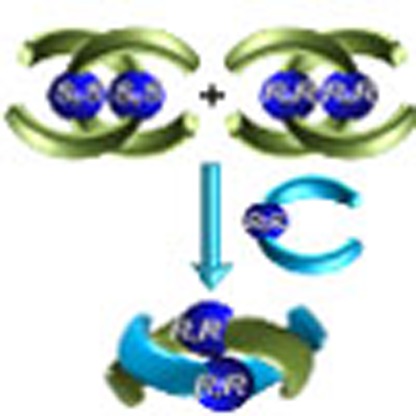

High-fidelity self-recognition and self-discrimination are of principal importance in living systems^1^, which enable biological macromolecules, such as proteins and DNA, to self-organize into uniform quaternary and double helical structures with a controlled handedness, respectively, through noncovalent interactions even within complex mixtures of subunits or molecular strands with a similar shape, size and sequence, thereby providing sophisticated functions that are essential for human life^1^,^2^. Such an incredible self-sorting performance observed in biological systems ultimately relies on the preprogrammed monomer sequences and homochirality of their building blocks and components^2^,^3^. Recent advances in the total chemical synthesis of unnatural D-proteins^4^,^5^ and L-DNA^6^ clearly revealed the indispensable role of the homochirality along with the monomer sequences of natural L-proteins and D-DNA towards chiral specificities in enantioselective reactions and complementary double-helix formations, respectively.

In organic and supramolecular chemistry, the control of the size and shape^7^,^8^,^9^,^10^,^11^,^12^,^13^, and topology^14^,^15^ as well as the handedness^16^,^17^,^18^,^19^,^20^,^21^,^22^,^23^,^24^,^25^,^26^,^27^,^28^,^29^,^30^,^31^ of supramolecular assemblies from mixtures of a variety of components via spontaneous self-sorting has become one of the urgent and emerging topics^10^,^32^,^33^ because it will not only enrich our understanding of the principles underling the precise recognition behaviour of biological macromolecules but also contribute to the development of novel supramolecular catalysts^34^, optoelectrical devices^35^ and sensors^36^. In most cases^32^,^33^, however, self-sorting has been achieved in a size- and shape-7,8,9,10,11,12,13 or topology-selective way^14^,^15^,^28^ through the formation of macrocycles^11^,^15^, capsules or cages^13^,^17^,^22^ and helices^23^,^24^,^27^,^30^,^31^,^37^, while a limited number of supramolecular systems undergoes chiral self-sorting, producing either homochiral^16^,^17^,^18^,^19^,^20^,^21^,^22^,^23^,^24^,^25^,^26^,^27^,^28^,^29^,^30^,^31^,^32^ or heterochiral assemblies^32^,^38^,^39^,^40^, which is mostly relevant to the biological processes as well as the state-of-the-art asymmetric catalysis^41^ and optical resolution on crystallization^42^.

Here we report artificial chirality- and sequence-selective chiral self-sorting of dimeric strands consisting of carboxylic acid or amidine groups joined by chiral amide linkers with different sequences (NHCO or CONH) through specific homoduplex and subsequent hetero- (complementary-) duplex formations with implications for biological double helices in DNA. DNA-like double helices have a significant advantage in the precise recognition of the monomer sequence^14^,^43^ and chain length between the complementary strands^44^. Although hydrogen-bond-driven peptide nucleic acids^45^ and metal-coordinate-bonded helicates^7^,^8^,^9^,^46^ are known to show self-sorting with respect to their sequences and/or chain lengths, chirality- and sequence-selective successive self-sorting is currently unknown.

## Results

### Design and synthesis of carboxylic acid and amidine dimers

**Figure 1:**
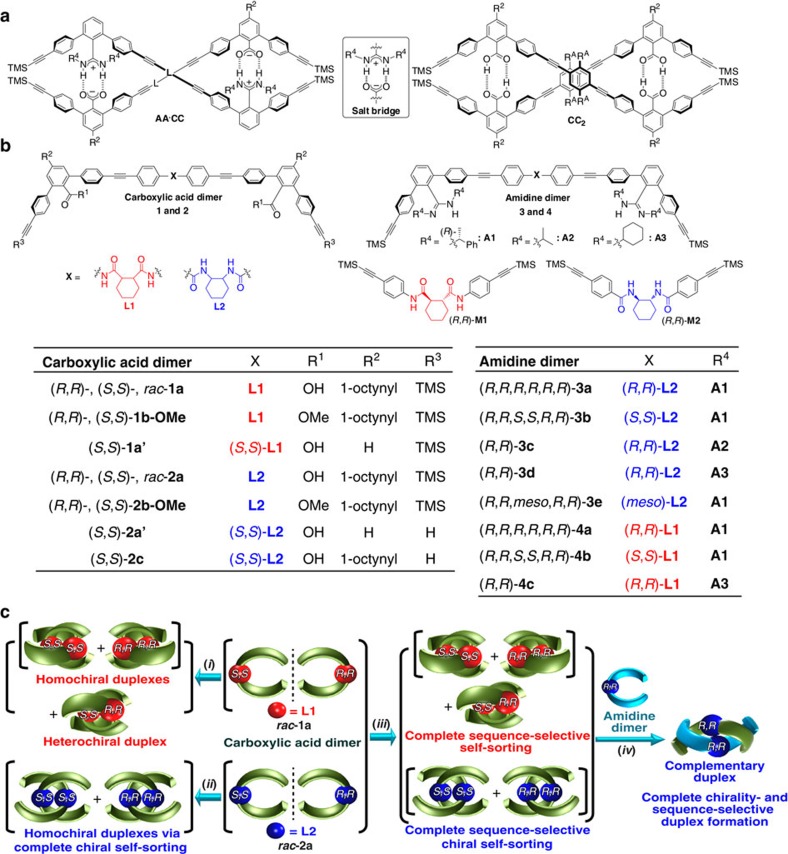
Chirality- and sequence-selective successive self-sorting. (**a**) Structures of heterodouble helix (**AA**·**CC**) and homodouble helix (**CC**_2_). (**b**) Structures of carboxylic acid and amidine dimer strands linked through chiral or *meso* amide residues (X=**L1** or **L2**). (**c**) Schematic representation of chirality- and sequence-selective successive self-sorting via specific homo- and complementary-duplex formations. Carboxylic acid dimers of *rac*-**1a** and *rac*-**2a** with different linker amide sequences form homo/hetero- (*i*) and homochiral (*ii*) duplexes, respectively. A mixture of *rac*-**1a** and *rac*-**2a** completely self-sorts in sequence-selective way to give duplexes of **1a** and **2a** (*iii*). Only one enantiomer among *rac*-**1a** and *rac*-**2a** ((*S*,*S*)- or (*R*,*R*)-**2a**) forms a complementary duplex with an amidine dimer linked through the same linker amide chirality and sequence to that of **1a** or **2a** via salt bridges, leading to perfect chirality- and sequence-selective duplex formation (*iv*).

Our molecular design is mainly based on the previously reported heterodouble helices composed of complementary dimer strands with *m*-terphenyl backbones intertwined through amidinium–carboxylate salt bridges (Fig. 1a, **AA·CC**)^44^,^47^,^48^,^49^,^50^. Owing to the high tolerance of the salt bridges towards various functional groups, a variety of linkers (L) could be introduced while maintaining the double-stranded helical structures with a one-handed helical sense biased by the chirality introduced on the amidine residues^49^. During the course of our study, we also found that an achiral carboxylic acid dimer joined by a *p*-diethynylbenzene linker with *n*-octyl (R^A^) substituents self-associated into a racemic homodouble helix through interstrand hydrogen bonds between the carboxy groups (Fig. 1a, (**CC**_**2**_))^51^.

We anticipated that either a right- or left-handed homodouble helix would be induced in the carboxylic acid dimers when chiral linkers^52^, such as (*S*,*S*)- or (*R*,*R*)-*trans*-1,2-cyclohexane-based (*c*Hex) *bis*-amide derivatives, were introduced between the monomer units (**1** and **2**, Fig. 1b). Incidentally, we have found a complete sequence-selective chiral self-sorting in a mixture of racemic (*rac*)-**1a** and *rac*-**2a** mediated by unique interstrand multihydrogen-bond-driven homoduplex formations, the structures of which are significantly different from our expected self-associated homodouble helices, and are further different from each other depending on the linker amide sequences (NHCO-*c*Hex (**L1**) or CONH-*c*Hex (**L2**), Fig. 1c). The further addition of an enantiopure amide-linked amidine dimer (**3** or **4**) with a particular linker amide sequence to a mixture of *rac*-**1a** and *rac*-**2a** results in the formation of a single optically pure complementary duplex with a 100% diastereoselectivity and sequence specificity stabilized by the amidinium–carboxylate salt bridges (Fig. 1c). In addition, we show that the diastereoselectivities during the complementary duplex formations, however, greatly rely on the sequence and chirality of the amide linkers of the amidine dimers relative to those of the carboxylic acid dimers and are almost independent of the amidine chirality. We also demonstrate a vital role of multihydrogen bonds formed between the amide linkers that determines the observed unique chirality- and sequence-selective homo- and complementary self-sorting.

A series of chiral dimers of carboxylic acids (**1**, **2**) and amidines (**3**, **4**) linked through chiral or *meso* amide residues with different sequences was prepared according to the reported methods (see Supplementary Methods)^47^,^50^,^53^.

### Self-sorting behaviour of carboxylic acid dimers

**Figure 2:**
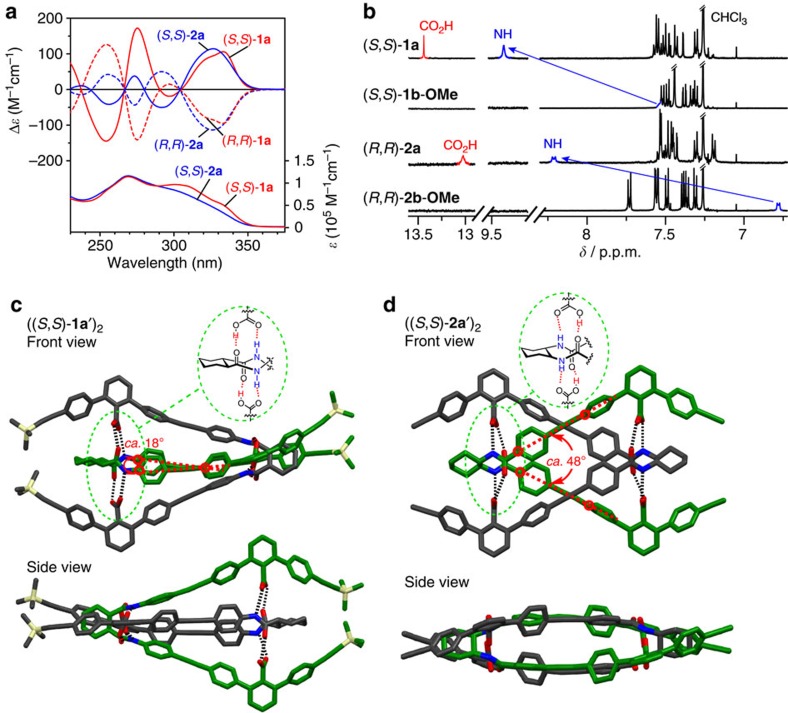
Homoduplex formations of optically active carboxylic acid dimers. (**a**) CD spectra (upper) of (*S*,*S*)-**1a** and -**2a** (0.50 mM) and (*R*,*R*)-**1a** and -**2a** (0.50 mM) in CDCl_3_ at 25 °C. Absorption spectra (bottom) of (*S*,*S*)-**1a** and -**2a** (0.50 mM) in CDCl_3_ at 25 °C are also shown. (**b**) Partial ^1^H NMR spectra of (*S*,*S*)-**1a** and -**1b-OMe** (1.0 mM) and (*R*,*R*)-**2a** and -**2b-OMe** (1.0 mM) in CDCl_3_ at 25 °C. The amide NH resonances were assigned using ^1^H 2D NMR spectroscopy (Supplementary Figs 11–13). (**c**,**d**) X-ray crystal structures of (*S*,*S*)-**1a′** (**c**) and (*S*,*S*)-**2a′** (**d**). Hydrogen atoms and solvent molecules are omitted for clarity. Black dotted lines represent interstrand hydrogen bonds. Average twist angles between the diphenylethynylene groups (red dotted lines) connecting to the amide linker, in which the twist angles are defined as the dihedral angles formed by the four carbon atoms (marked with red open circles), and interstrand hydrogen-bonding schemes are also shown.

We first investigated the self-association behaviour of the carboxylic acid dimers (*R*,*R*)- or (*S*,*S*)-**1a** and -**2a**. The circular dichroism (CD) spectra of the enantiomeric (*R*,*R*)- and (*S*,*S*)-strands of **1a** and **2a** in CDCl_3_ showed intense split-type Cotton effects that are mirror images of each other (Fig. 2a). However, the CD intensities significantly decreased in dimethyl sulfoxide (DMSO; Supplementary Fig. 1) because of dissociation into single strands in DMSO because DMSO is a strong hydrogen-bond acceptor and strongly hampers the interstrand hydrogen bonds; therefore, the CD spectral patterns and intensities of (*R*,*R*)-**1a** as well as (*R*,*R*)-**2a** in DMSO were similar to those of the carboxy-protected (*R*,*R*)-**1b-OMe** and -**2b-OMe** in CDCl_3_, respectively. The formation of homochiral duplexes **1a**_2_ and **2a**_2_ in CDCl_3_ was also supported by the corresponding dimer peaks [**1a**_2_+Na]^+^ and [**2a**_2_+Na]^+^ observed in their matrix-assisted laser desorption/ionization time-of-flight mass spectra (Supplementary Fig. 2).

The dimerization constants (*K*_d_) of (*R*,*R*)-**1a** and (*S*,*S*)-**2a** in CHCl_3_ at 25 °C were then estimated to be *ca.* 3.2 × 10^7^ and *ca.* 5.0 × 10^5 ^M^−1^ by extrapolation of the *K*_d_ values measured in CHCl_3_/THF (tetrahydrofuran) and CHCl_3_/CH_3_CN mixtures with various ratios, respectively, because the *K*_d_ values in pure CHCl_3_ are too high to accurately estimate (Supplementary Fig. 3). These significantly higher *K*_d_ values as compared with those of the model compounds (*R*,*R*)-**M1** and -**M2** lacking the *m*-terphenyl moieties (*K*_d_=*ca.* 20 M^−1^, Supplementary Fig. 4) and **CC** (*K*_d_=*ca.* 1.4 × 10^3 ^M^−1^)^51^ suggest the strong multiple interstrand hydrogen bonds, which were consistent with the significant downfield shifts of the amide NH proton resonances of (*S*,*S*)-**1a** and (*R*,*R*)-**2a** in their ^1^H NMR spectra in comparison with the corresponding carboxy-protected (*S*,*S*)-**1b-OMe** and (*R*,*R*)-**2b-OMe** (Fig. 2b). The variable-temperature ^1^H NMR spectral changes of (*R*,*R*)-**1a** and -**2a** also support the strong interstrand hydrogen bonds; the amide NH signals of both (*R*,*R*)-**1a** and -**2a** showed larger negative temperature coefficients (Δ*δ*/Δ*T* (ppb per °C)) in DMSO-*d*_6_ (−5.8 and −4.9) than those in CDCl_3_ (−2.0 and −3.1; Supplementary Figs 5–8)^54^.

The X-ray crystallographic analyses of analogous (*S*,*S*)-**1a′** and -**2a′** whose structures are assumed to be almost identical to those of (*S*,*S*)-**1a** and -**2a**, respectively, except for the pendant and/or terminal substituents (see Fig. 1b), revealed a unique deeply intertwined duplex structure (Fig. 2c,d). Unexpectedly, these structures are completely different from a self-associated homodouble helix-like **CC**_2_ (see Fig. 1a). Each strand adopts a similar ‘U-shape' structure resulting from the (*S*,*S*)-*trans*-1,2-cyclohexyl linker residue, binding together through the eight interstrand hydrogen bonds, in which each amide group at the linker moiety is sandwiched between the two carboxy groups of the other strand. The remarkable structural difference between (*S*,*S*)-**1a′** and -**2a′** in the solid state is that the homoduplex ((*S*,*S*)-**2a′**)_2_ has a well-packed structure due to the almost parallel orientation of the strands (Fig. 2d), while the intertwined strands of (*S*,*S*)-**1a′** are oriented perpendicular to each other, resulting in a less sterically hindered structure (Fig. 2c). This difference in the steric hindrance between ((*S*,*S*)-**1a′**)_2_ and ((*S*,*S*)-**2a′**)_2_ is reasonably correlated with the difference in their self-association affinities; the *K*_d_ value of (*S*,*S*)-**1a** is ∼10^2^ times greater than that of (*S*,*S*)-**2a**. The ^1^H two-dimensional (2D) nuclear Overhauser effect spectroscopy (NOESY) analysis of (*R*,*R*)-**1a** and (*S*,*S*)-**2a** in CDCl_3_ (Supplementary Figs 9–13) showed characteristic interstrand NOE cross-peaks including those between the linker *c*Hex protons and the terminal aromatic protons of the *m*-terphenyl moieties along with the interstrand aromatic protons, indicating that such unique intertwined structures in the solid state were retained in solution. The observed large downfield shifts of the amide NH and CO_2_H protons of (*S*,*S*)-**1a** and (*R*,*R*)-**2a** in their ^1^H NMR spectra (Fig. 2b) also support the structures.

**Figure 3:**
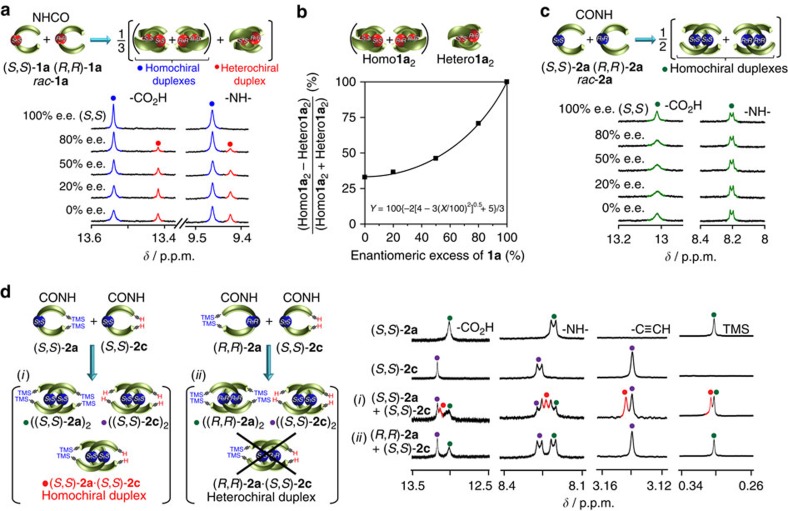
Chiral self-sorting behaviour of *rac*-1a and *rac*-2a. (**a**) ^1^H NMR spectra of carboxylic acid CO_2_H and amide NH proton resonances of **1a** (0.50 mM) with different % e.e. in CDCl_3_ at 25 °C. Blue and red circles denote the signals because of the homochiral and heterochiral duplexes, respectively. Full-scale spectra are shown in Supplementary Fig. 18a. (**b**) Plots of % d.e. of **1a**_2_ versus the % e.e. of **1a** in CDCl_3_ at 25 °C. The plots were fitted using *Y*=100{−2[4−3(*X*/100)^2^]^0.5^+5}/3 (equation A), where *X* and *Y* are % e.e. of **1a** and % d.e. of **1a**_2_, respectively (for details, see Supplementary Methods). (**c**) ^1^H NMR spectra of carboxylic acid CO_2_H and amide NH proton resonances of **2a** (0.50 mM) with different % e.e. in CDCl_3_ at 25 °C. Full-scale spectra are shown in Supplementary Fig. 18b. (**d**) Partial ^1^H NMR spectra (0.50 mM) of (*S*,*S*)-**2a**, (*S*,*S*)-**2c**, an equimolar mixture (1.0 mM) of (*S*,*S*)-**2a** and (*S*,*S*)-**2c**, and an equimolar mixture (1.0 mM) of (*R*,*R*)-**2a** and (*S*,*S*)-**2c** in CDCl_3_ at 25 °C. Red circles denote the signals because of the duplex (*S*,*S*)-**2a**·(*S*,*S*)-**2c**. Full-scale spectra are shown in Supplementary Fig. 20.

We next investigated the chiral self-sorting behaviour of *rac*-**1a** and *rac*-**2a** using ^1^H NMR in CDCl_3_ at 25 °C. The ^1^H NMR spectrum of *rac*-**1a** showed two sets of signals with an integral ratio of 2:1 (0% enantiomeric excess (e.e.), Fig. 3a), which did not coalesce even at 50 °C (Supplementary Fig. 14), whereas a single set of signals was observed in DMSO (Supplementary Fig. 15), in which the duplexes undergo the anticipated dissociation into the single strands. The NOESY spectrum of *rac*-**1a** in CDCl_3_ also exhibited interstrand NOEs similar to those for the enantiopure (*R*,*R*)-**1a** without chemical exchange cross-peaks between the two species except for the labile CO_2_H signals (Supplementary Figs 16 and 17), suggesting that the exchange rate between the species is slower than the present NMR timescale. The major set of signals of *rac*-**1a** is identical to the signals of the homochiral duplexes ((*R*,*R*)-**1a**)_2_ and ((*S*,*S*)-**1a**)_2_, and the minor set of the signals decreased with an increase in the % e.e. of **1a** (Fig. 3a). Therefore, the minor signals are unambiguously assigned to the heterochiral duplex (*R*,*R*)-**1a**·(*S*,*S*)-**1a**.

The diastereomeric excess (d.e.) values (%) of **1a**_2_ (homochiral duplex versus heterochiral duplex) were then plotted versus the % e.e. of **1a**, which matched well with the simulated curve using the equation (*Y*=100{−2[4−3(*X*/100)^2^]^0.5^+5}/3) (inset in Fig. 3b; equation A) obtained by a modification of the reported equation (Fig. 3b)^55^,^56^ (for details, see Supplementary Methods), suggesting no preference in the diastereomeric duplex formation, thus forming the homochiral and heterochiral **1a**_2_ duplexes in a 2:1 molar ratio in CDCl_3_ at 25 °C. As anticipated, however, the diastereomeric duplex formation is sensitive to temperature and its molar ratio changed on heating or cooling (Supplementary Fig. 14d), being attributed to a subtle change in the relative interstrand hydrogen-bonding strength between the homo- and heterochiral **1a**_2_, which was in accordance with the observed differences in their amide NH temperature coefficients (Supplementary Fig. 14c).

In sharp contrast, *rac*-**2a** was completely chiral self-sorted to form only the homochiral duplexes ((*R*,*R*)-**2a**)_2_ and ((*S*,*S*)-**2a**)_2_, giving one set of ^1^H NMR signals in CDCl_3_ independent of the % e.e. of **2a** (Fig. 3c) and temperature from −20 to 50 °C (Supplementary Fig. 19). This was also supported by the following mixing experiments of (*S*,*S*)- or (*R*,*R*)-**2a** with its trimethylsilyl (TMS)-deprotected analogue (*S*,*S*)-**2c**; an equimolar mixture of (*S*,*S*)-**2a** and (*S*,*S*)-**2c** in CDCl_3_ gave a new set of signals corresponding to the heteroduplex (*S*,*S*)-(**2a**)·(*S*,*S*)-**2c**, whereas no trace amount of such a distinguishable heteroduplex derived from (*R*,*R*)-**2a** and (*S*,*S*)-**2c** was observed for an equimolar mixture of (*R*,*R*)-**2a** and (*S*,*S*)-**2c** (Fig. 3d), indicating that *rac*-**2a** completely self-sorts to form only the homochiral duplexes ((*R*,*R*)-**2a**)_2_ and ((*S*,*S*)-**2a**). In addition, these observations exclude the possibility of the fast exchange between the hetero- and homochiral duplexes of *rac*-**2a** on the present NMR timescale.

The difference in the chiral self-sorting behaviour between **1a** and **2a** may be due to the difference in the twist angles between the two rigid diphenylethynylene groups connecting to the chiral amide linkers with a different sequence, in which the average twist angle in ((*S*,*S*)-**2a′**)_2_ (*ca.* 48°) is much larger than that in ((*S*,*S*)-**1a′**)_2_ (*ca.* 18°) in the solid state (Fig. 2c,d). The density functional theory (DFT) calculations revealed that the homochiral duplex ((*S*,*S*)-**1a**)_2_ is only 17.0 kJ mol^−1^ more stable than the heterochiral duplex (*R*,*R*)-**1a**·(*S*,*S*)-**1a**, whereas, for **2a**, the homochiral duplex formation ((*S*,*S*)-**2a**)_2_ is much more favourable than the heterochiral duplex formation (*R*,*R*)-**2a**·(*S*,*S*)-**2a** by 51.7 kJ mol^−1^ (Supplementary Fig. 21a–d), being reasonably consistent with the previously discussed experimental results. The observed large energy difference (Δ*E*) between the homochiral and heterochiral duplexes for **2a**_2_ (−51.7 kJ mol^−1^) as compared with that for **1a**_2_ (−17.0 kJ mol^−1^) is attributed to a distorted structure of the heterochiral homoduplex (*R*,*R*)-**2a**·(*S*,*S*)-**2a** that lacks one interstrand hydrogen bond between the amide NH and carboxy C=O groups (Supplementary Fig. 21d), while all of the possible interstrand hydrogen bonds are retained in both the homo- and heterochiral **1a**_2_ during the calculations because of the perpendicular orientation of the strands as seen in the solid state (Fig. 2c), resulting in a similar duplex structure (Supplementary Fig. 21a,b). Therefore, predominant homochiral self-sorting could not take place for *rac*-**1a** (Fig. 3a).

**Figure 4:**
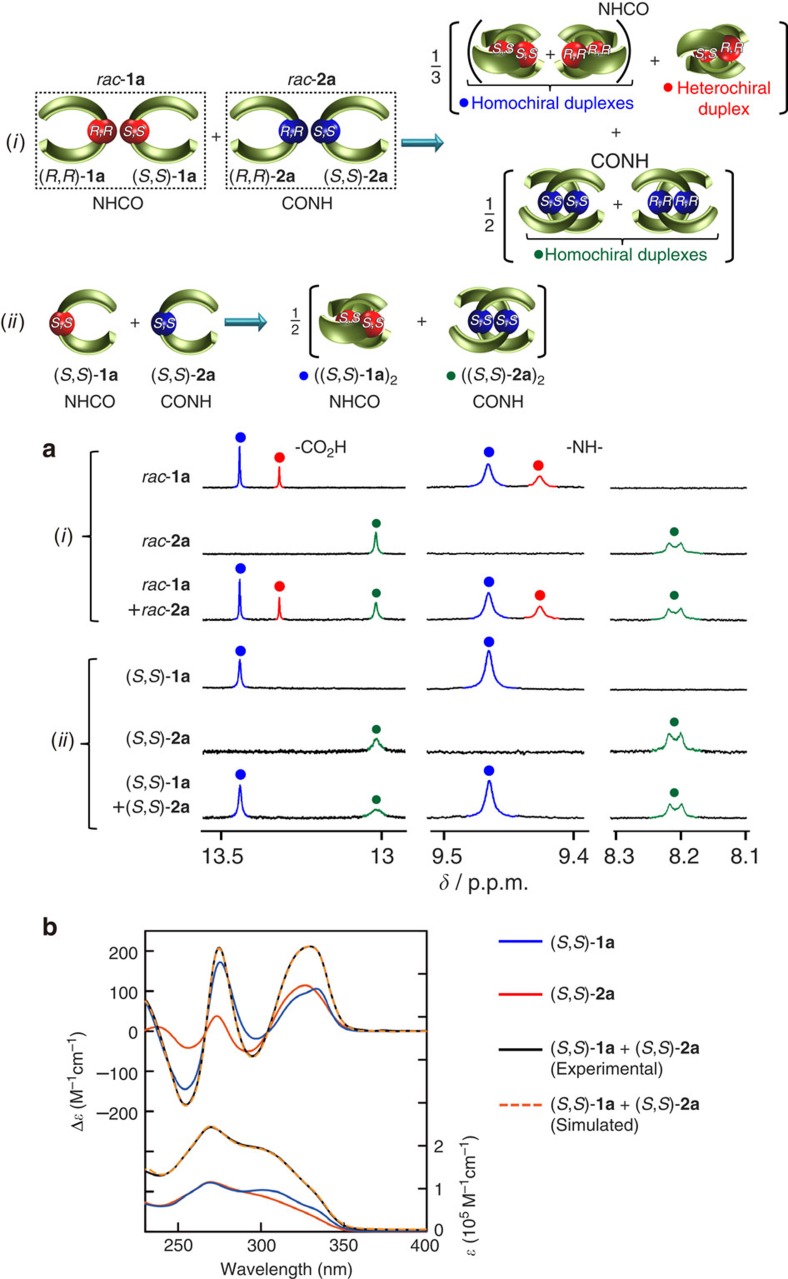
Sequence-selective chiral self-sorting behaviour of *rac*-1a and *rac*-2a. (**a**) ^1^H NMR spectra of carboxylic acid CO_2_H and amide NH proton resonances of *rac*-**1a** (1.0 mM), *rac*-**2a** (1.0 mM), an equimolar mixture (1.0 mM) of *rac*-**1a** and *rac*-**2a** (*S,S*)-**1a** (0.50 mM), (*S,S*)-**2a** (0.50 mM) and an equimolar mixture (1.0 mM) of (*S*,*S*)-**1a** and (*S*,*S*)-**2a** in CDCl_3_ at 25 °C. Full-scale spectra are shown in Supplementary Fig. 22. (**b**) CD and absorption spectra of (*S*,*S*)-**1a** (0.50 mM), (*S*,*S*)-**2a** (0.50 mM) and those observed and simulated for the mixture of an equimolar amount of (*S*,*S*)-**1a** and (*S*,*S*)-**2a** (0.50 mM) in CDCl_3_ at 25 °C.

Of further interest is that an equimolar mixture of *rac*-**1a** and *rac*-**2a** showed complete self-sorting in terms of their linker amide sequences, giving only the enantiomeric pairs of the **1a**_2_ and **2a**_2_ duplexes as explicitly observed in their ^1^H NMR spectra in CDCl_3_ (Fig. 4a). Each duplex of **1a** and **2a** is unable to exchange, that is further supported by the ^1^H NMR and CD measurements of an equimolar mixture of (*S*,*S*)-**1a** and (*S*,*S*)-**2a** in CDCl_3_ (Fig. 4a,b); the ^1^H NMR spectrum of an equimolar mixture of (*S*,*S*)-**1a** and (*S*,*S*)-**2a** in CDCl_3_ displayed only two sets of the signals corresponding to ((*S*,*S*)-**1a**)_2_ and ((*S*,*S*)-**2a**)_2_ (Fig. 4a). Moreover, the mixture showed a CD spectrum precisely identical to the simulated one (Fig. 4b), indicating that the complete sequence-selective chiral self-sorting took place within mixtures of *rac*-**1a** and *rac*-**2a**. The DFT calculations demonstrate the important role of the interstrand hydrogen bonds; the energy-minimized duplex structure of (*S*,*S*)-**1a**·(*S*,*S*)-**2a** with the different linker amide sequences has a mismatched arrangement of the interstrand hydrogen bonds (Supplementary Fig. 21e), and is 11.1 and 32.6 kJ mol^−1^ less stable than those of the chiral self-sorted homoduplexes ((*S*,*S*)-**2a**)_2_ and ((*S*,*S*)-**1a**)_2_, respectively (Supplementary Fig. 21a,c). On the basis of the total energies of the calculated structures (Supplementary Fig. 21), the stabilities of the duplexes decrease in the following order: ((*S*,*S*)-**1a**)_2_>(*R*,*R*)-**1a**·(*S*,*S*)-**1a**>((*S*,*S*)-**2a**)_2_>(*S*,*S*)-**1a**·(*S*,*S*)-**2a**>(*R*,*R*)-**2a**·(*S*,*S*)-**2a**, which are in good agreement with the experimental results, and the energetically disfavoured duplexes (*S*,*S*)-**1a**·(*S*,*S*)-**2a** and (*R*,*R*)-**2a**·(*S*,*S*)-**2a** were not detected at all under the present experimental conditions.

### Chirality- and sequence-selective heteroduplex formation

With all the above results taken together with our previous findings of the complementary double-helix formations through amidinium–carboxylate salt bridges^44^,^47^,^48^,^49^,^50^, we envisaged that the optically active amidine dimers linked through the chiral or *meso* amide linkers with a different sequence (NHCO-*c*Hex (**L1**) or CONH-*c*Hex (**L2**)) (**3a–e** and **4a–c**, Fig. 1b) could selectively recognize the carboxylic acid dimers (**1a** and **2a**) according to the linker chirality and sequences, thereby leading to a unprecedented diastereo- and sequence-selective complementary duplex formation.

On mixing *rac*-**2a** and (*R*,*R*,*R*,*R*,*R*,*R*)-**3a** composed of the dimeric (*R*,*R*,*R*,*R*) amidine linked through the (*R*,*R*)-**L2** linker in a 2:1 molar ratio in CDCl_3_, the ^1^H NMR spectrum immediately changed to that consisting of complementary diastereomeric duplexes of **3a**·(*R*,*R*)-**2a** and **3a**·(*S*,*S*)-**2a** together with the remaining **2a** (Supplementary Fig. 25). The nonequivalent N–H proton signals appeared at a low magnetic field (13–13.5 p.p.m.), suggesting the preferred-handed duplex formation stabilized by salt bridges^44^,^47^,^48^,^49^,^50^. Because the chain exchange rate between the complementary dimer strands is slower than the NMR timescale^49^, the diastereoselectivity between (*R*,*R*,*R*,*R*,*R*,*R*)-**3a** and *rac*-**2a** was estimated to be d.e.=58% ((*R*,*R*)-**2a**-rich, run 1, Table 1) from their integral ratio, indicating the homochiral (*R*,*R*) selectivity with respect to their *c*Hex linker chirality. This diastereoselectivity was further confirmed by CD; the observed CD spectrum is almost identical to the simulated CD (Supplementary Fig. 26).

**Figure 5:**
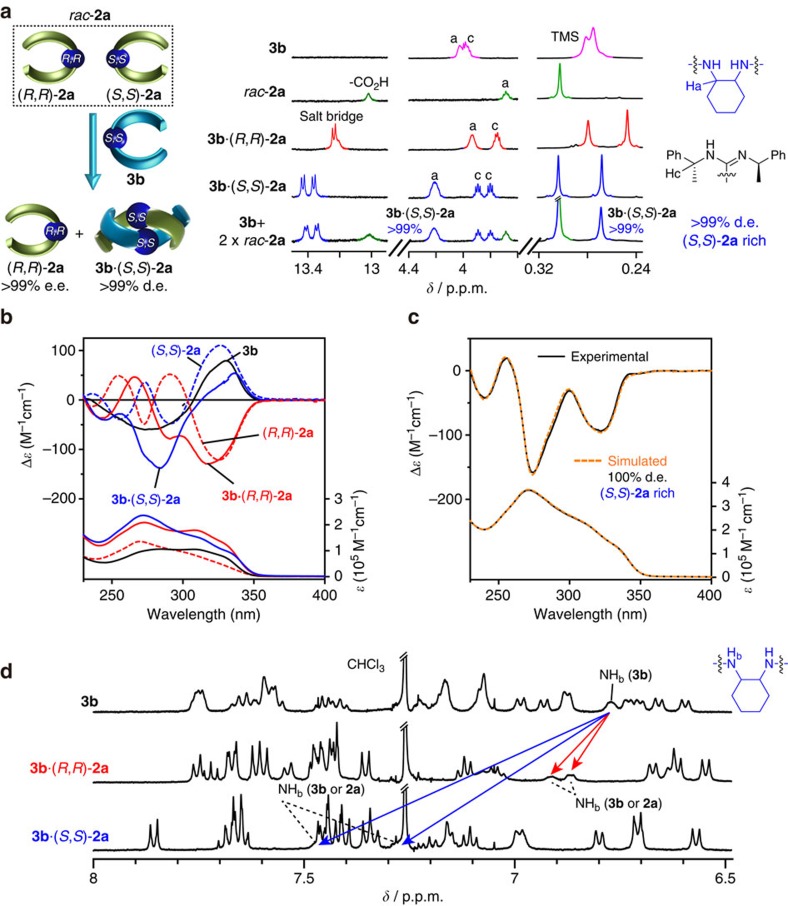
Diastereoselective complementary duplex formation. (**a**) Partial ^1^H NMR spectra of **3b** (0.50 mM), *rac*-**2a** (0.50 mM), **3b**·(*R*,*R*)-**2a** (0.50 mM), **3b**·(*S*,*S*)-**2a** (0.50 mM) and a mixture of **3b** (0.50 mM) and two equivalents of *rac*-**2a** (1.0 mM) in CDCl_3_ at 25 °C. Full-scale spectra are shown in Supplementary Fig. 27. (**b**) CD (upper) and absorption (bottom) spectra (0.50 mM) of **3b**, (*R*,*R*)-**2a**, (*S*,*S*)-**2a**, **3b**·(*R*,*R*)-**2a** and **3b**·(*S*,*S*)-**2a** in CDCl_3_ at ambient temperature. (**c**) Experimental and simulated CD (d.e.=100%) and absorption spectra for a mixture of **3a** (0.50 mM) and two equivalents of *rac*-**2a** (1.0 mM) in CDCl_3_ at ambient temperature. For the simulated CD and absorption spectra, see Supplementary Methods. (**d**) Partial ^1^H NMR spectra (0.5 mM) of **3b**, **3b**·(*R*,*R*)-**2a** and **3b**·(*S*,*S*)-**2a** in CDCl_3_ at 25 °C. The linker amide NH resonances were assigned using ^1^H 2D NMR spectroscopy (Supplementary Fig. 62).

Interestingly, the amidine dimer (*R*,*R*,*S*,*S*,*R*,*R*)-**3b** bearing the opposite (*S*,*S*)-**L2** linker showed a perfect diastereoselectivity towards *rac*-**2a**, producing the complementary duplex of **3b**·(*S*,*S*)-**2a** with d.e.>99% as well as (*R*,*R*)-**2a** (e.e.>99%) remaining as a homochiral duplex, as evidenced by the ^1^H NMR and identical experimental and simulated CD spectra (run 2, Table 1, Fig. 5a–c and Supplementary Figs 27 and 28). It should be noted that highly disfavoured **3b**·(*R*,*R*)-**2a** was hardly formed even in the presence of a large excess of (*R*,*R*)-**2a** (12 equivalents), indicating that the binding affinity of (*S*,*S*)-**2a** to **3b** is at least 10^5^ times higher than that of (*R*,*R*)-**2a** (Supplementary Fig. 29). This surprisingly high diastereoselectivity^57^,^58^ of **3b** towards *rac*-**2a** enabled the separation of *rac*-**2a** into the (*S*,*S*)- and (*R*,*R*)-**2a** enantiomers by facile silica gel column chromatography, giving the corresponding optically pure enantiomers in 85% and 63% yield, respectively (Supplementary Fig. 30). (*R*,*R*,*S*,*S*,*R*,*R*)-**3b** could be recovered for further separation of *rac*-**2a**. Moreover, the resolved optically pure (*R*,*R*)-**2a** (e.e.>99%) could be also used to separate *rac*-**3b** in principle. More practically, the chemical bonding of (*R*,*R*,*S*,*S*,*R*,*R*)-**3b** to chromatographic supports including silica gel will enable more efficient and preparative separation of *rac*-**2a** as well as other enantiomers as a novel chiral stationary phase (CSP) for chromatographic enantioseparation. The complementary 1:1 duplex formation of **3b**·(*S*,*S*)-**2a** was further evidenced using an electron-spray ionization mass spectroscopic (ESI-MS) measurement and vapour pressure osmometry experiment (Supplementary Figs 31–33).

More interestingly, (*R*,*R*)-**3c** and (*R*,*R*)-**3d**, in which the chiral amidine residues were replaced by achiral isopropyl (**3c**) and cyclohexyl (**3d**) substituents, respectively, also performed a perfect diastereoselectivity towards *rac*-**2a** to form duplexes only with (*R*,*R*)-**2a** (d.e.>99%) having the same linker chirality, which also resulted in the optically pure (*S,S*)-**2a** (e.e.>99%) as the remaining homochiral duplex (runs 3, 4, Table 1 and Supplementary Figs 34–37), suggesting that the diastereoselective duplex formation is governed by the linker chirality, and the chiral amidine residues may be no longer required. In other words, an anticipated complementary double-stranded helix formation stabilized by chiral amidinium–carboxylate salt bridges that would occur on both sides of the linker may not be prerequisite in order to achieve diastereoselective duplex formations. Therefore, the amidine dimer having the chiral amidine residues linked through achiral *meso*-linker (*R*,*R*,*meso*,*R*,*R*)-**3e** completely lost its diastereoselectivity (d.e.=0%; run 5, Table 1 and Supplementary Figs 38 and 39).

Various attempts to obtain crystals of the complementary duplex dimers suitable for an X-ray analysis produced only amorphous solids. Therefore, the energy-minimized structures of the duplexes of (*R*,*R*,*S*,*S*,*R*,*R*)-**3b**·(*S*,*S*)-**2a** and (*R*,*R*,*S*,*S*,*R*,*R*)-**3b**·(*R*,*R*)-**2a** were constructed using the semiempirical molecular orbital calculations followed by the DFT calculations based on an analogous crystal structure^47^. The initial model structures of (*R*,*R*,*S*,*S*,*R*,*R*)-**3b**·(*S*,*S*)-**2a** and its diastereomer (*R*,*R*,*S*,*S*,*R*,*R*)-**3b**·(*R*,*R*)-**2a** with a right-handed twisted conformation, in which the pendant 1-octynyl groups are replaced by hydrogen atoms, were constructed so as to satisfy the following experimental results: (1) as shown in their ^1^H NMR spectra (Fig. 5d and Supplementary Fig. 62), all of the amide NH resonances of (*R*,*R*,*S*,*S*,*R*,*R*)-**3b**·(*S*,*S*)-**2a** showed significant downfield shifts compared with those of the amidine strand (*R*,*R*,*S*,*S*,*R*,*R*)-**3b** (Δ*δ*=0.53 or 0.73 p.p.m.; Table 2, entry 2), indicating that all of the amide protons participate in hydrogen bonds. In contrast, the amide NH resonances of (*R*,*R*,*S*,*S*,*R*,*R*)-**3b**·(*R*,*R*)-**2a** were slightly shifted downfield (Δ*δ*=0.12 or 0.09 p.p.m.), suggesting weak hydrogen bonds. (2) An analogous complementary dimeric duplex composed of the identical (*R*,*R*)-amidine and carboxylic acid dimer strands linked by diacetylene residues bound together through salt bridges was determined to have a right-handed helical structure using the single-crystal X-ray analysis^47^.

**Figure 6:**
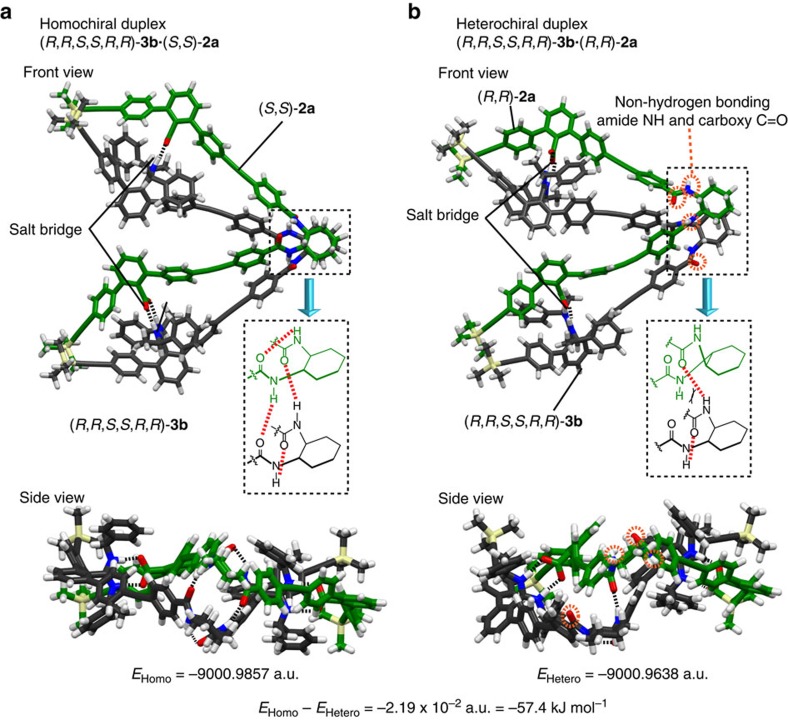
Structures of complementary duplexes. Capped-stick drawings of the structures for the homochiral duplex (*R*,*R*,*S*,*S*,*R*,*R*)-**3b**·(*S*,*S*)-**2a** (**a**) and the heterochiral duplex (*R*,*R*,*S*,*S*,*R*,*R*)-**3b**·(*R*,*R*)-**2a** (**b**) with respect to the *c*Hex linker chirality optimized by DFT calculations. DFT-calculated energies are also shown in the bottom.

The resultant energy-minimized structures of (*R*,*R*,*S*,*S*,*R*,*R*)-**3b**·(*S*,*S*)-**2a** and (*R*,*R*,*S*,*S*,*R*,*R*)-**3b**·(*R*,*R*)-**2a** with their total energies are depicted in Fig. 6a,b, respectively, which revealed that they take a largely bent-shaped, right-handed double-helix-like structure, in which (*R*,*R*,*S*,*S*,*R*,*R*)-**3b**·(*S*,*S*)-**2a** is 57.4 kJ mol^−1^ more stable than the other. All of the linker amide protons in **3b**·(*S*,*S*)-**2a** form inter- or intramolecular hydrogen bonds with the amide C=O groups (average NH···O distance=2.14 Å; Fig. 6a), whereas only two of the amide protons of **3b**·(*R*,*R*)-**2a** participate in such hydrogen bonds with a longer average NH···O distance (2.30 Å; Fig. 6b). The observed difference in the hydrogen-bonding networks between **3b**·(*S*,*S*)-**2a** and **3b**·(*R*,*R*)-**2a** was supported by their ^1^H NMR spectra (Fig. 5d and Supplementary Fig. 62). Therefore, the calculated structures reasonably explain the present unexpectedly high diastereoselective duplex formation between **3b** and (*S*,*S*)-**2a**. The 2D NOESY spectra of **3b**·(*S*,*S*)-**2a** in CDCl_3_ showed interstrand NOE cross-peaks including those between the terminal TMS protons and the phenyl moieties of the amidine residues along with the interstrand aromatic protons, while interstrand NOEs between the linker *c*Hex protons were not identified because of the same amide linker sequence (NHCO-*c*Hex) whose chemical shifts were quite similar to each other (Supplementary Figs 68–70). On the other hand, an interstrand NOE was clearly observed between the linker *c*Hex protons of **4b**·(*R*,*R*)-**2a** because of the different linker amide sequences (Supplementary Figs 71 and 72). Considering all the results including the 2D NOESY, ESI-MS, vapour pressure osmometry, salt-bridge formations and large downfield shifts of the linker amide NH resonances of **3b**·(*S*,*S*)-**2a**, the calculated structure (Fig. 6a) is most likely retained in solution.

The amidine dimers **3a**–**3d** also formed a duplex in a diastereoselective manner towards *rac*-**1a** with the linker amide sequence (NHCO-*c*Hex) different from that of *rac*-**2a** and **3a**–**3d** (CONH-*c*Hex; runs 6–9, Table 1 and Supplementary Figs 40–47). However, their diastereoselectivities significantly decreased with the opposite heterochiral selectivity with respect to the *c*Hex linker chirality, for example, (*R*,*R*)-**3c** preferentially formed a duplex with (*S*,*S*)-**1a** in 64% d.e., which is in significant contrast to the homochiral selectivity observed between **3a**–**3d** and *rac*-**2a** (runs 1–4, Table 1). Again, the linker chirality plays a critical role so that (*R*,*R*,*meso*,*R*,*R*)-**3e** showed no diastereoselectivity (run 10, Table 1 and Supplementary Figs 48 and 49). The reason for this heterochiral preference between **3a-d** and *rac*-**1a** is not totally understood, but may be due to the difference in the interstrand hydrogen-bonding networks that could be more efficiently formed between the heterochiral linker amide residues than between the homochiral counterparts. This speculation is supported by the fact that the amide NH resonances of, for instance, the heterochiral (*R*,*R*,*S*,*S*,*R*,*R*)-**3b**·(*R*,*R*)-**1a** showed higher downfield shifts (Δ*δ*=0.56 p.p.m.) as compared with those of the homochiral (*R*,*R*,*S*,*S*,*R*,*R*)-**3b**·(*S*,*S*)-**1a** (Δ*δ*=0.03 p.p.m.), respectively (Supplementary Fig. 64 and Table 2, entry 1).

We also investigated the diastereoselective duplex formations using a series of amidine dimers (**4a**–**4c**) with the NHCO-*c*Hex linker amide sequence towards *rac*-**1a** and *rac*-**2a**. In all the duplex formations, moderate diastereoselectivities (64–80% d.e.) were observed except for that between **4b** and *rac*-**2a** (34% d.e.; runs 11–16, Table 1 and Supplementary Figs 50–61), which were in contrast to the perfect diastereoselectivities achieved between **3b**–**3d** and *rac*-**2a**. As for the selectivities with regard to the *c*Hex amide linker chirality, there is the same tendency; **4a**–**4c** favourably formed duplexes with **1a** and **2a** with the same and opposite configurations, respectively. As a typical example, (*R*,*R*)-**4c** preferentially formed a duplex with (*R*,*R*)-**1a**, but with (*S*,*S*)-**2a** in 70 and 80% d.e., respectively. The observed diastereoselectivities also rely on the difference in the interstrand hydrogen-bond strengths between the diastereomeric duplexes as revealed by more downfield shifts of the amide NH protons for the major duplex diastereoselectively formed during the complexations (Supplementary Figs 65–67 and Table 2, entries 3 and 4).

**Figure 7:**
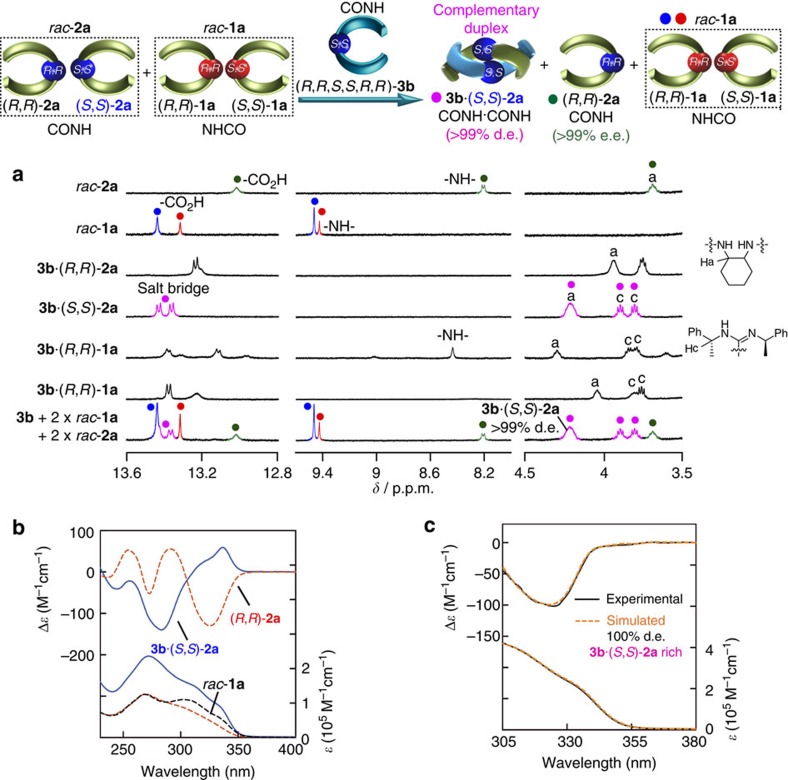
Chirality- and sequence-selective complementary duplex formation. (**a**) Partial ^1^H NMR spectra of *rac*-**2a** (0.50 mM), *rac*-**1a** (0.50 mM), **3b**·(*R*,*R*)-**2a** (0.50 mM), **3b**·(*S*,*S*)-**2a** (0.50 mM), **3b**·(*R*,*R*)-**1a** (0.50 mM), **3b**·(*S*,*S*)-**1a** (0.50 mM) and a mixture of **3b** (0.50 mM) and two equivalents of *rac*-**2a** (1.0 mM) and *rac*-**1a** (1.0 mM) in CDCl_3_ at 25 °C. Full-scale spectra are shown in Supplementary Fig. 73. (**b**) CD (upper) and absorption (bottom) spectra (0.50 mM) of **3b**·(*S*,*S*)-**2a**, (*R*,*R*)-**2a** and *rac*-**1a** in CDCl_3_ under ambient temperature. (**c**) Experimental and simulated CD (d.e.=100%) and absorption spectra for a mixture of **3b** (0.50 mM) and two equivalents of each of *rac*-**1a** (1.0 mM) and *rac*-**2a** (1.0 mM) in CDCl_3_ under ambient temperature.

On the basis of these results, we anticipated that particular chiral amidine dimers would simultaneously recognize the chirality and sequence within a mixture of complementary carboxylic acid dimers via specific duplex formations. In fact, the mixing of two equivalents of each of *rac*-**1a** and *rac*-**2a** with (*R*,*R*,*S*,*S*,*R*,*R*)-**3b** in CDCl_3_ resulted in the formation of only the (*R*,*R*,*S*,*S*,*R*,*R*)-**3b**·(*S*,*S*)-**2a** duplex (d.e.>99%), while no duplex formation was observed towards *rac*-**1a**. As a result, an optically pure (*R*,*R*)-**2a** (e.e.>99%) quantitatively remained as a homochiral duplex together with free *rac*-**1a** (Fig. 7); thus, the perfect sequence- (CONH-*c*Hex or NHCO-*c*Hex) and chirality- ((*R*,*R*) or (*S*,*S*)) selective duplex formation was achieved as unambiguously evidenced using their ^1^H NMR analysis (Fig. 7a) and the observed and simulated CD spectra (Fig. 7b,c). The linker chirality rather than the amidine chirality also plays a vital role in the sequence- and chirality-selective complementary duplex formation; therefore, (*R*,*R*)-**3d** composed of achiral amidine residues formed a duplex only with (*R*,*R*)-**2a** with a complete diastereoselectivity (d.e.>99%) and sequence specificity in a mixture of *rac*-**1a** and *rac*-**2a** (Supplementary Figs 74 and 75).

## Discussion

We demonstrate here an unprecedented successive chiral self-sorting during the unique homo- and subsequent heteroduplex formations through interstrand multihydrogen bonds that take place in a perfect sequence-selective way accompanied with an extraordinary high diastereoselectivity in the latter case, which enables the separation of the racemic strands into the enantiomers. The present chirality- and sequence-selective successive self-sorting is most likely achieved because of the rigid chiral geometries generated by the chiral amide-linked dimeric strands that arrange functional groups in a suitable way for specific duplex formations.

In our previous studies, we showed that a carboxylic acid dimer such as **CC** formed an intertwined homodouble helix via interstrand association of the carboxylic acids^51^, which further formed a double-stranded helix (**AA·CC**) with the complementary amidine dimer such as **AA** (Fig. 1a)^44^,^47^,^48^,^49^. The helical handedness of the complementary double helix is fully controlled by the chirality introduced on the amidine residues^49^. On the basis of the present studies, however, it appears that the chirality and sequence (NHCO-*c*Hex or CONH-*c*Hex) of the amide linkers of dimeric carboxylic acid and amidine strands are of primary importance and dictate the overall chirality- and sequence-selective self-assemblies of the dimer strands, resulting from the unique multihydrogen-bonding networks formed between the linker amide residues. Importantly, the amidine chirality is not almost involved in the present diastereoselective complementary duplex formations that mostly rely on the sequence and chirality of the amide linkers of the amidine dimers relative to those of the carboxylic acid dimers.

The dimers of amidines and carboxylic acids possess reactive trimethylsilylethynyl groups at the ends, which allows longer oligomers joined by chiral amide linkers with specific sequence and chirality to be synthesized, which would provide a unique strategy for asymmetric template synthesis and an artificial replication system^59^,^60^ based on chirality- and sequence-selective multihydrogen-bond-assisted duplex formations, and also provide a clue towards a better understanding of the biological chiral self-sorting process.

## Methods

### General procedures for the complementary duplex formations

A typical procedure for the diastereoselective duplex formations between amidine and carboxylic acid dimers is described below. Stock solutions of (*R*,*R*,*R*,*R*,*R*,*R*)-**3a** (2.0 mM; solution I) and *rac*-**2a** (2.0 mM; solution II) were prepared in dry CDCl_3_. Aliquots of I (0.40 μmol, 200 μl), II (0.80 μmol, 400 μl) and dry CDCl_3_ (200 μl) were added to an NMR tube, and its ^1^H NMR spectrum was measured at 25 °C to determine the % d.e. of the **3a**·**2a** duplexes (Supplementary Fig. 25). The solution was also used for measuring the CD (Supplementary Fig. 26). In a similar way, other diastereoselective duplex formations were preformed and their % d.e. values were estimated (Table 1).

### Optical resolution of *rac*-2a via the heteoduplex formation

(*R*,*R*,*S*,*S*,*R*,*R*)-**3b** (5.44 mg, 3.58 μmol) and two equivalents of *rac*-**2a** (9.46 mg, 7.16 μmol) were dissolved in CHCl_3_ (2 ml), which produced an equimolar mixture of **3b**·(*S*,*S*)-**2a** and (*R*,*R*)-**2a** judging from its ^1^H NMR spectrum (Fig. 5a) and were separated into the first and second fractions, respectively, using flash column chromatography (SiO_2_, 2 cm (i.d.) × 12 cm; eluent; CHCl_3_/MeOH=1/0–50/1, v/v). The % d.e. of the **3b**·(*S*,*S*)-**2a** duplex obtained (6.3 mg) was >99% as estimated using its CD and ^1^H NMR spectra (Supplementary Fig. 30a,c)), while the second fraction mainly contained (*R*,*R*)-**2a** along with a small amount of **3b**·(*S*,*S*)-**2a** and an unknown compound probably generated during the column chromatography. Thus, the second fraction was purified again using flush column chromatography, yielding **3b**·(*S*,*S*)-**2a** (2.3 mg, >99% d.e., total yield; 8.6 mg, 85%) and (*R*,*R*)-**2a** containing a small amount of the unknown compound. Further purification using recycle size-exclusion chromatography (eluent; CHCl_3_) afforded pure (*R*,*R*)-**2a** (2.99 mg) in 63% yield; its e.e. value was estimated to be >99% on the basis of the CD and ^1^H NMR measurements (Supplementary Fig. 30b,c).

## Additional information

**Accession codes:** The X-ray crystallographic coordinates for structures ((*S*,*S*)-**1a′** and (*S*,*S*)-**2a′**) reported in this Article have been deposited at the Cambridge Crystallographic Data Centre (CCDC), under deposition number CCDC 1036590 and 1036591. These data can be obtained free of charge from The Cambridge Crystallographic Data Centre via www.ccdc.cam.ac.uk/data_request/cif.

**How to cite this article:** Makiguchi, W. *et al*. Chirality- and sequence-selective successive self-sorting via specific homo- and complementary-duplex formations. *Nat. Commun.* 6:7236 doi: 10.1038/ncomms8236 (2015).

## Supplementary Material

Supplementary Information Supplementary Figures 1-117, Supplementary Tables 1-2, Supplementary Methods and Supplementary References

Supplementary Data 1 cif file for x-ray crystal structure of (S,S)-1a'

Supplementary Data 2 cif file for x-ray crystal structure of (S,S)-2a'

## Figures and Tables

**Table 1:** Results of diastereoselective duplex formations between chiral amidine dimers (3a-4c) and racemic carboxylic acid dimers (*rac*-1a and *rac*-2a).

**Amidine dimer (amide sequence)**	***rac*****-2a (CONH-*****c*****Hex)**			***rac*****-1a (NHCO-*****c*****Hex)**		
	**Run**	d.e. (%)*	**Suppl.**† **Fig. no.**	**Run**	**d.e.** (%)*	**Suppl.**† **Fig. no.**
(*R,R,**R**,**R**,R,R*)-**3a** (CONH-*c*Hex)	1	58 (*R*,*R*)	25	6	14 (*S*,*S*)	40
(*R,R,**S**,**S**,R,R*)-**3b** (CONH-*c*Hex)	2	>99 (*S*,*S*)	27	7	64 (*R*,*R*)	42
(***R**,**R***)-**3c** (CONH-*c*Hex)	3	>99 (*R*,*R*)	34	8	64 (*S*,*S*)	44
(***R**,**R***)-**3d** (CONH-*c*Hex)	4	>99 (*R*,*R*)	36	9	68 (*S*,*S*)	46
(*R,R,meso,R,R*)-**3e** (CONH-*c*Hex)	5	0	38	10	0	48
(*R,R,**R**,**R**,R,R*)-**4a** (NHCO-*c*Hex)	11	70 (*S*,*S*)	50	14	64 (*R*,*R*)	56
(*R,R,**S**,**S**,R,R*)-**4b** (NHCO-*c*Hex)	12	34 (*R*,*R*)	52	15	74 (*S*,*S*)	58
(***R**,**R***)-**4c** (NHCO-*c*Hex)	13	80 (*S*,*S*)	54	16	70 (*R*,*R*)	60

^*^Estimated using ^1^H NMR (CDCl_3_, 25 °C).

^†^Supplementary Fig. no.

**Table 2:** Relationships between the diastereoselective complementary duplex formations and downfield chemical shifts (Δ*δ*) of the linker amide NH resonances of the amidine strands of **3b·1a–4b·2a** duplexes from the monomeric amidine strands (**3b** and **4b**).

**Entry**	**Combination**	**Duplex**	**Δ*****d***_**NH**_ **(p.p.m.)**	**d.e. (%)**	**Supplementary Fig no.**
1	(*R*,*R*,***S***,***S***,*R*,*R*)-**3b** (CONH-*c*Hex) and **1a** (NHCO-*c*Hex)	**3b**·(*R*,*R*)-**1a**favoured	0.56	64 ((*R*,*R*)-**1a**-rich)	64
		**3b**·(*S*,*S*)-**1a**disfavoured	0.03		
2	(*R*,*R*,***S***,***S***,*R*,*R*)-**3b** (CONH-*c*Hex) and **2a** (CONH-*c*Hex)	**3b**·(*R*,*R*)-**2a**disfavoured	0.12 or 0.09*	>99 ((*S*,*S*)-**2a**-rich)	62
		**3b**·(*S*,*S*)-**2a**favoured	0.73 or 0.53*		
3	(*R*,*R*,***S***,***S***,*R*,*R*)-**4b** (NHCO-*c*Hex) and **1a** (NHCO-*c*Hex)	**4b**·(*R*,*R*)-**1a**disfavoured	0.52	74 ((*S*,*S*)-**1a**-rich)	67
		**4b**·(*S*,*S*)-**1a**favoured	1.16 or 1.08*		
4	(*R*,*R*,***S***,***S***,*R*,*R*)-**4b** (NHCO-*c*Hex) and **2a** (CONH-*c*Hex)	**4b**·(*R*,*R*)-**2a**favoured	0.90	34 ((*R*,*R*)-**2a**-rich)	66
		**4b**·(*S*,*S*)-**2a**disfavoured	0.59		

*c*Hex, (*R*,*R*)-*trans*-1,2-cyclohexane.

^*^The amide NH resonances of the amidine strands of duplexes could not be distinguished from those of the carboxylic acid strands because of overlapping with the phenyl and aliphatic proton signals. For the assignments of the amide NH proton signals, see Supplementary Figs 62–67.
