# Supplementary material for: Chirality- and sequence-selective successive self-sorting via specific homo- and complementary-duplex formations
Source: Nat Commun. 2015 Jun 8;6:7236. doi: 10.1038/ncomms8236 (PMC4468858; doi:10.1038/ncomms8236)
Supplement: Supplementary Information — Supplementary Figures 1-117, Supplementary Tables 1-2, Supplementary Methods and Supplementary References [file ncomms8236-s1.pdf]

1. Supplementary Figures

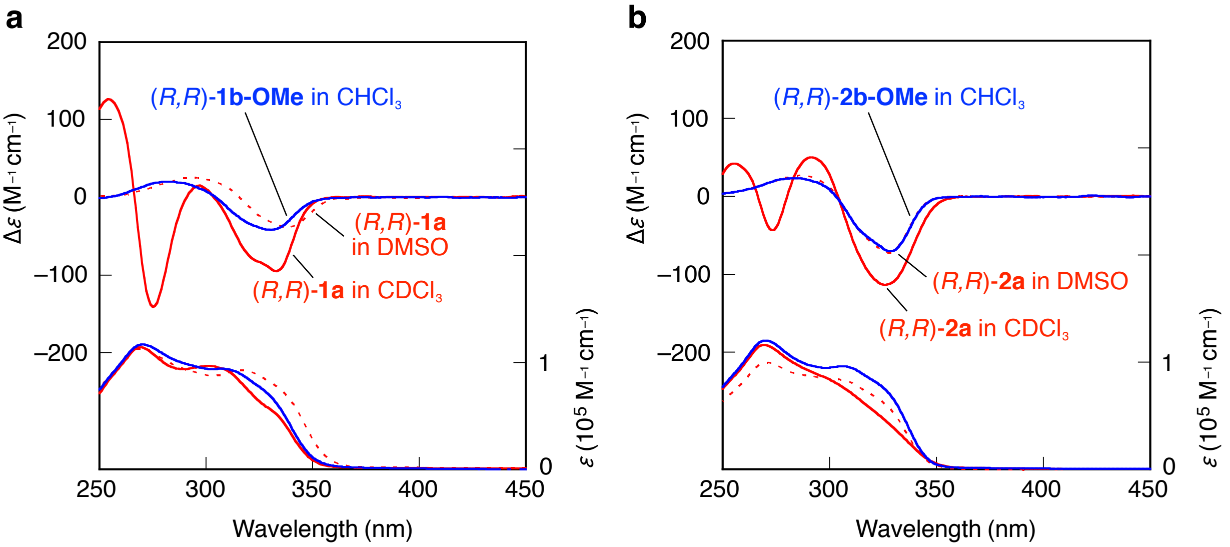

Supplementary Figure 1 | CD and absorption spectra of (*R*,*R*)-1a, -2a, -1b-OMe and -2b-OMe in $CDCl_3$ and DMSO. (**a**) CD and absorption spectra of (*R*,*R*)-**1a** (0.50 mM, red dashed lines) in DMSO, (*R*,*R*)-**1a** (red solid lines) in $CDCl_3$, and (*R*,*R*)-**1b-OMe** (0.50 mM, blue) in $CHCl_3$ at ambient temperature. (**b**) CD and absorption spectra of (*R*,*R*)-**2a** (0.50 mM, red dashed lines) in DMSO, (*R*,*R*)-**2a** in $CDCl_3$ (red solid lines), and (*R*,*R*)-**2b-OMe** (0.50 mM, blue) in $CHCl_3$ at ambient temperature. It should be noted that the split-type Cotton effects were still observed for (*R*,*R*)-**1a** and -**2a** even in the monomeric state in DMSO because of the rigid chiral cyclohexyl skeleton with a twisted orientation of the two identical π-conjugated chromophores[1].

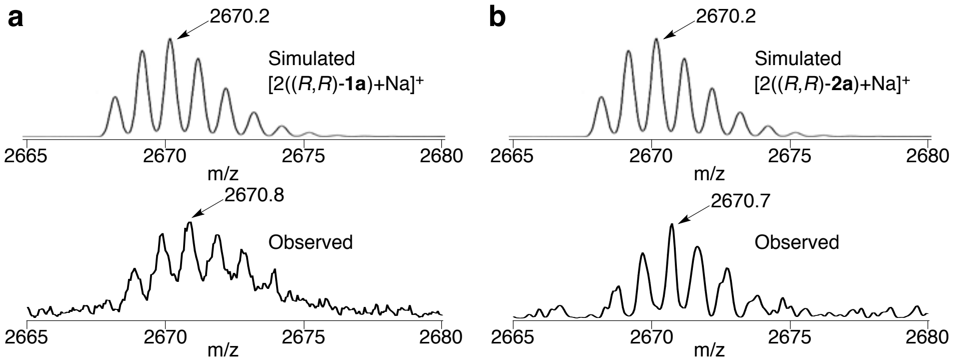

Supplementary Figure 2 | MALDI-TOF-MS spectra of (*R*,*R*)-1a and (*R*,*R*)-2a. Partial MALDI-TOF-MS spectra (positive mode; matrix: THAP) of (**a**) [(*R*,*R*)-**1a**]$_2$ and (**b**) [(*R*,*R*)-**2a**]$_2$.

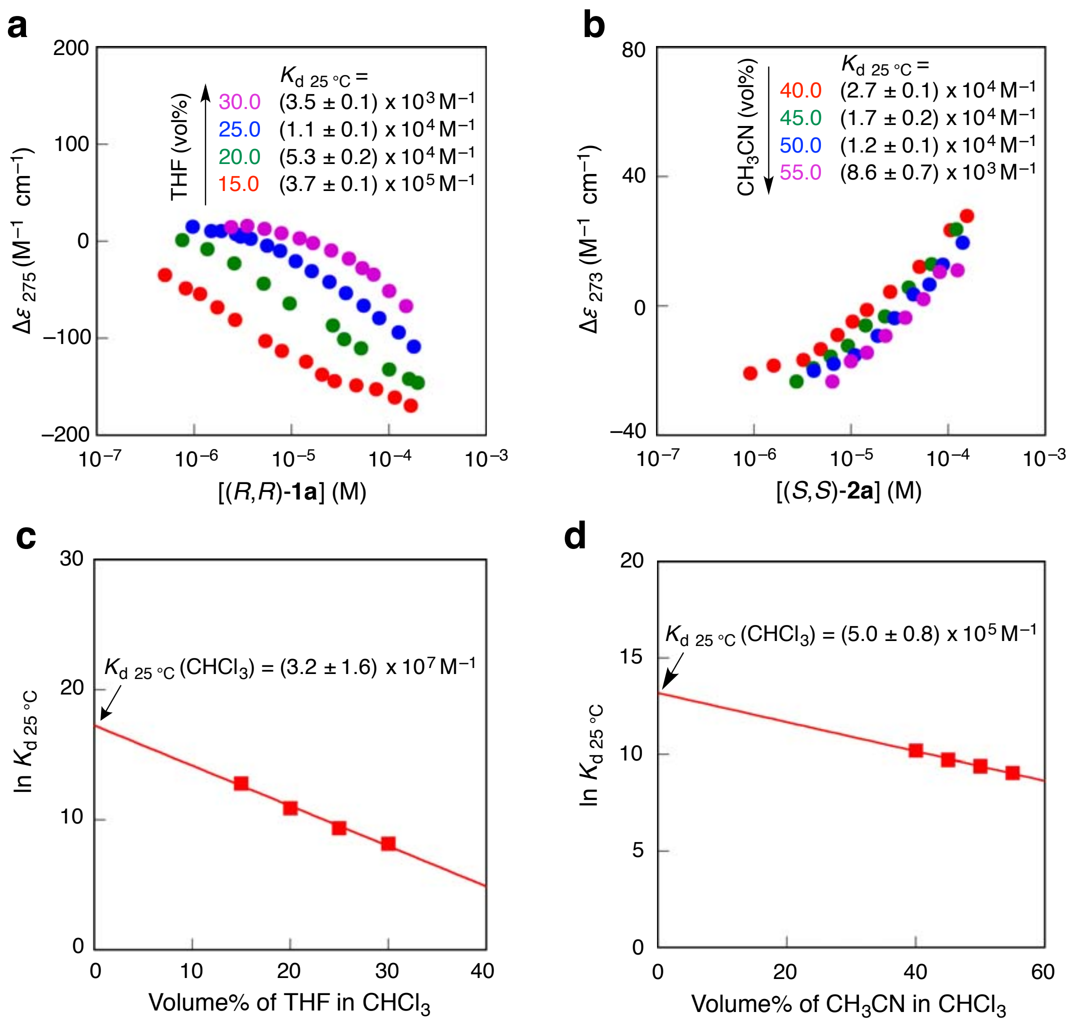

Supplementary Figure 3 | Determination of $K_{d\ 25\ °C}$ for (*R*,*R*)-1a and (*S*,*S*)-2a in $CHCl_3$. Plots of the $\Delta\varepsilon_{275}$ values versus the logarithm of the concentrations of (*R*,*R*)-**1a** (**a**) and (*S*,*S*)-**2a** (**b**) in various $CHCl_3$/THF and $CHCl_3$/CH_3CN mixtures, respectively. Plots of ln $K_{d\ 25\ °C}$ of (*R*,*R*)-**1a** (**c**) and (*S*,*S*)-**2a** (**d**) versus vol% of THF and CH_3CN in $CHCl_3$, respectively.

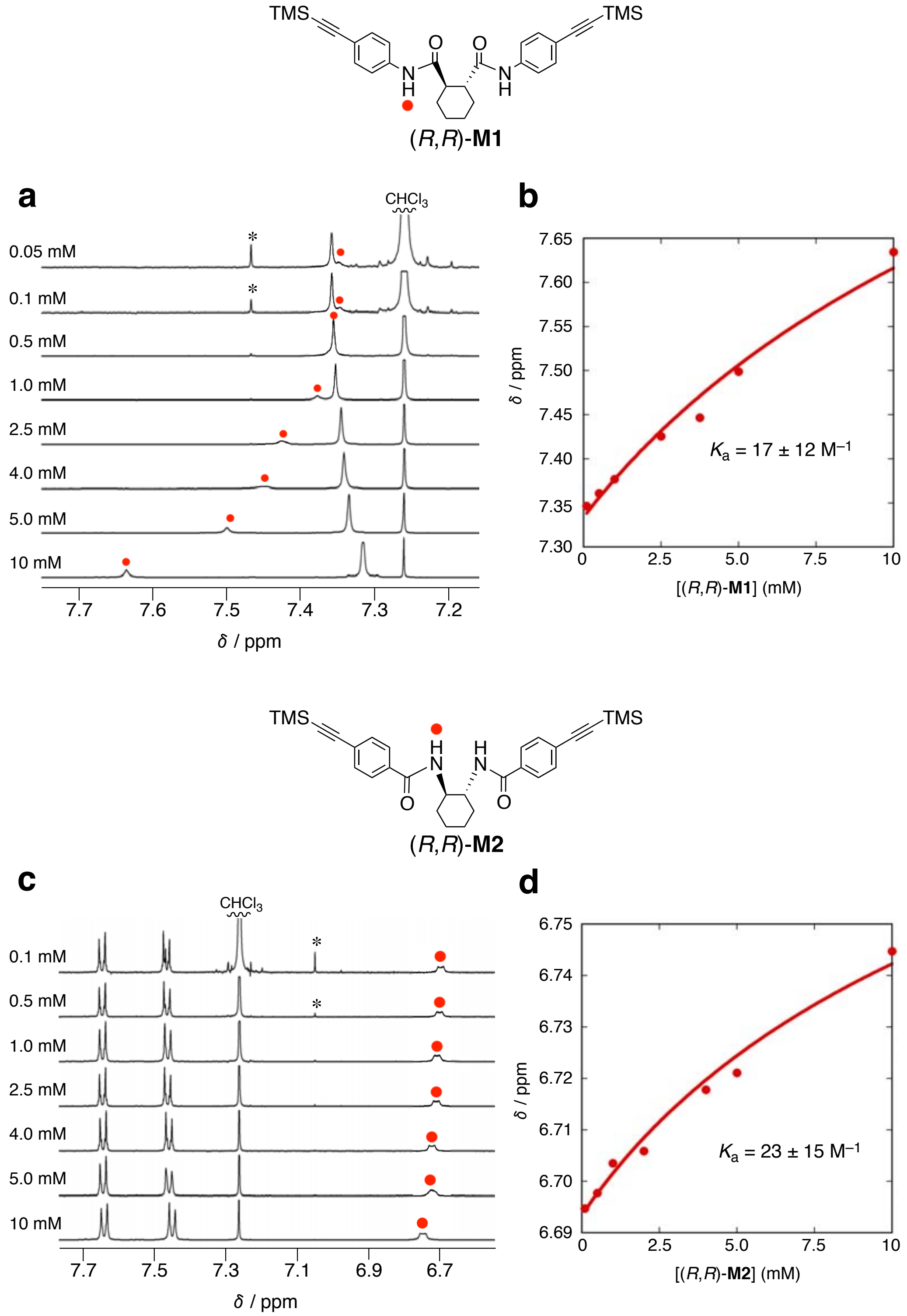

Supplementary Figure 4 | Determination of K_a for (*R*,*R*)-M1 and (*R*,*R*)-M2 in $CDCl_3$. Partial concentration-dependent ^{1}H NMR spectra (500 MHz) of (*R*,*R*)-**M1** (**a**) and (*R*,*R*)-**M2** (**c**) in $CDCl_3$ at 25 °C. ✻ denotes the satellite peaks of the solvent. Plots of the NH chemical shifts versus the concentration of (*R*,*R*)-**M1** (**b**) and (*R*,*R*)-**M2** (**d**) in $CDCl_3$. The curves in the plots were obtained by the curve-fitting method, giving the self-association constants (K_a) of (*R*,*R*)-**M1** **and** (*R*,*R*)-**M2** to be 17 ± 12 M^{-1} and 23 ± 15 M^{-1}, respectively.

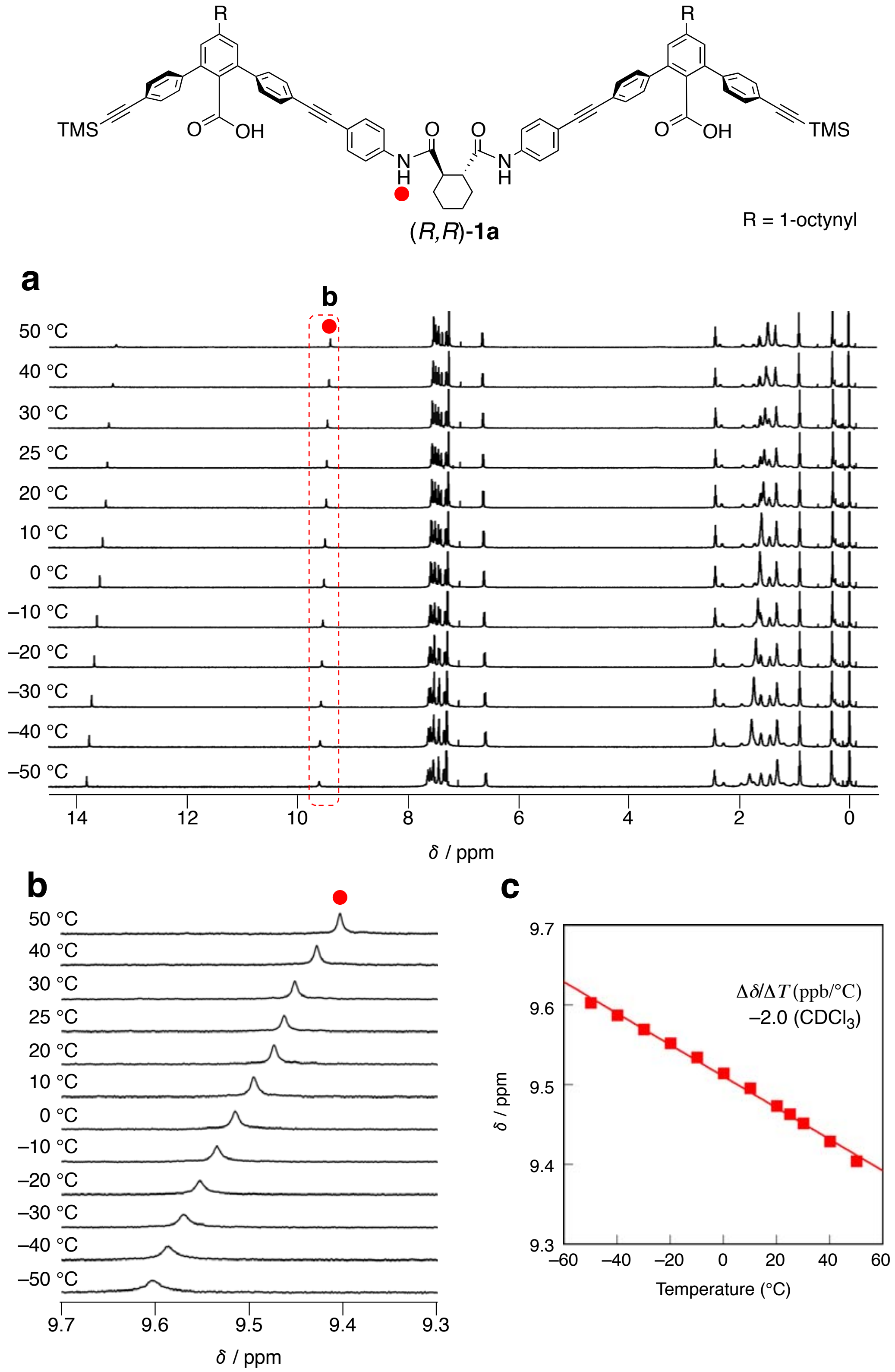

Supplementary Figure 5 | Variable-temperature (VT) ¹H NMR spectra of (*R*,*R*)-1a in CDCl$_3$. Full (**a**) and partial (**b**) variable-temperature ^{1}H NMR spectra (500 MHz, 1.0 mM) of (*R*,*R*)-**1a** in CDCl$_3$ from –50 °C to 50 °C. (**c**) Plots of NH chemical shifts of (*R*,*R*)-**1a** in the ^{1}H NMR spectra versus temperature in CDCl$_3$.

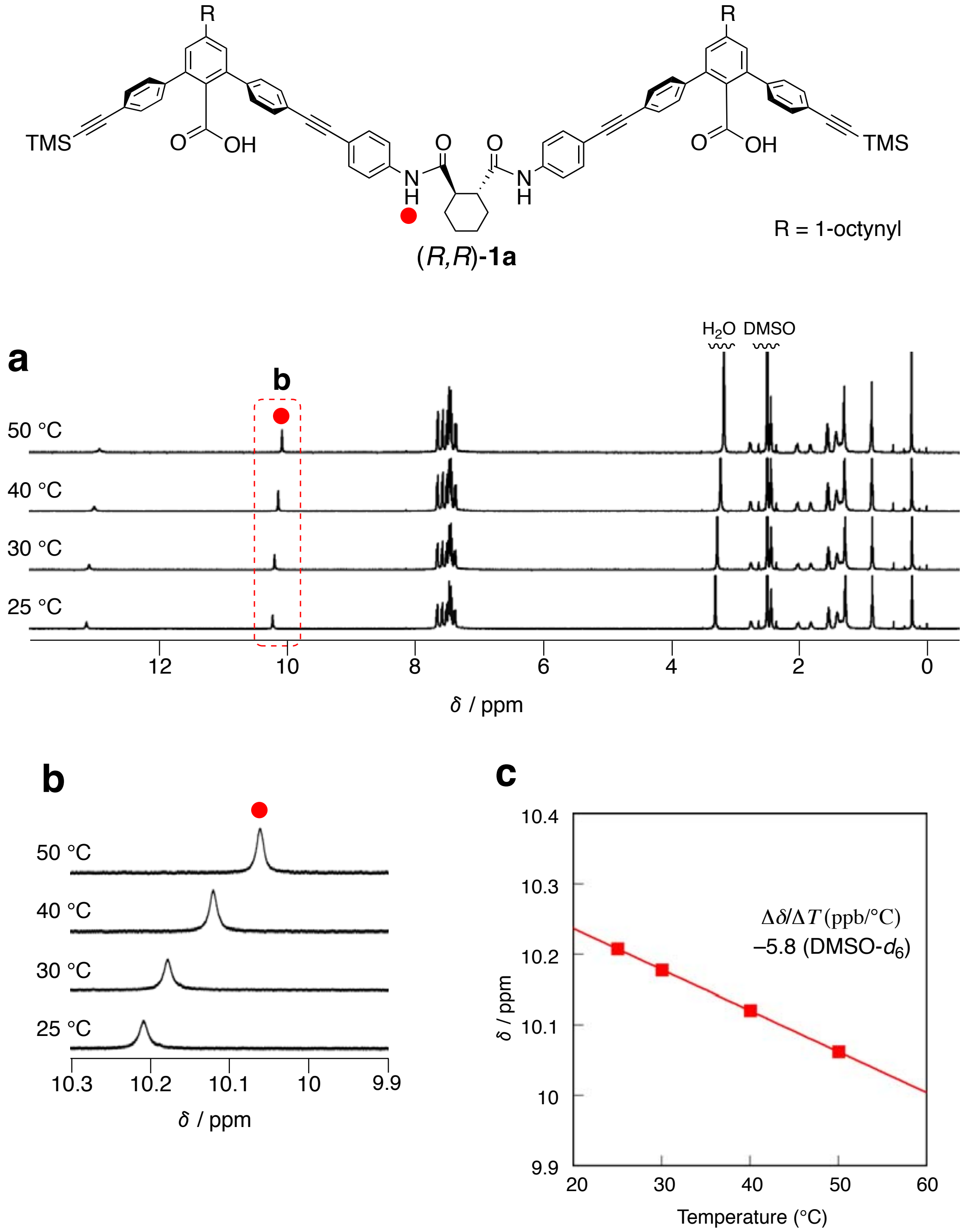

Supplementary Figure 6 | VT ^{1}H NMR spectra of (*R*,*R*)-1a in DMSO-d_6. Full (**a**) and partial (**b**) variable-temperature ^{1}H NMR spectra (500 MHz, 1.0 mM) of (*R*,*R*)-**1a** in DMSO-d_6 from 25 °C to 50 °C. (**c**) Plots of NH chemical shifts of (*R*,*R*)-**1a** in the ^{1}H NMR spectra versus temperature in DMSO-d_6.

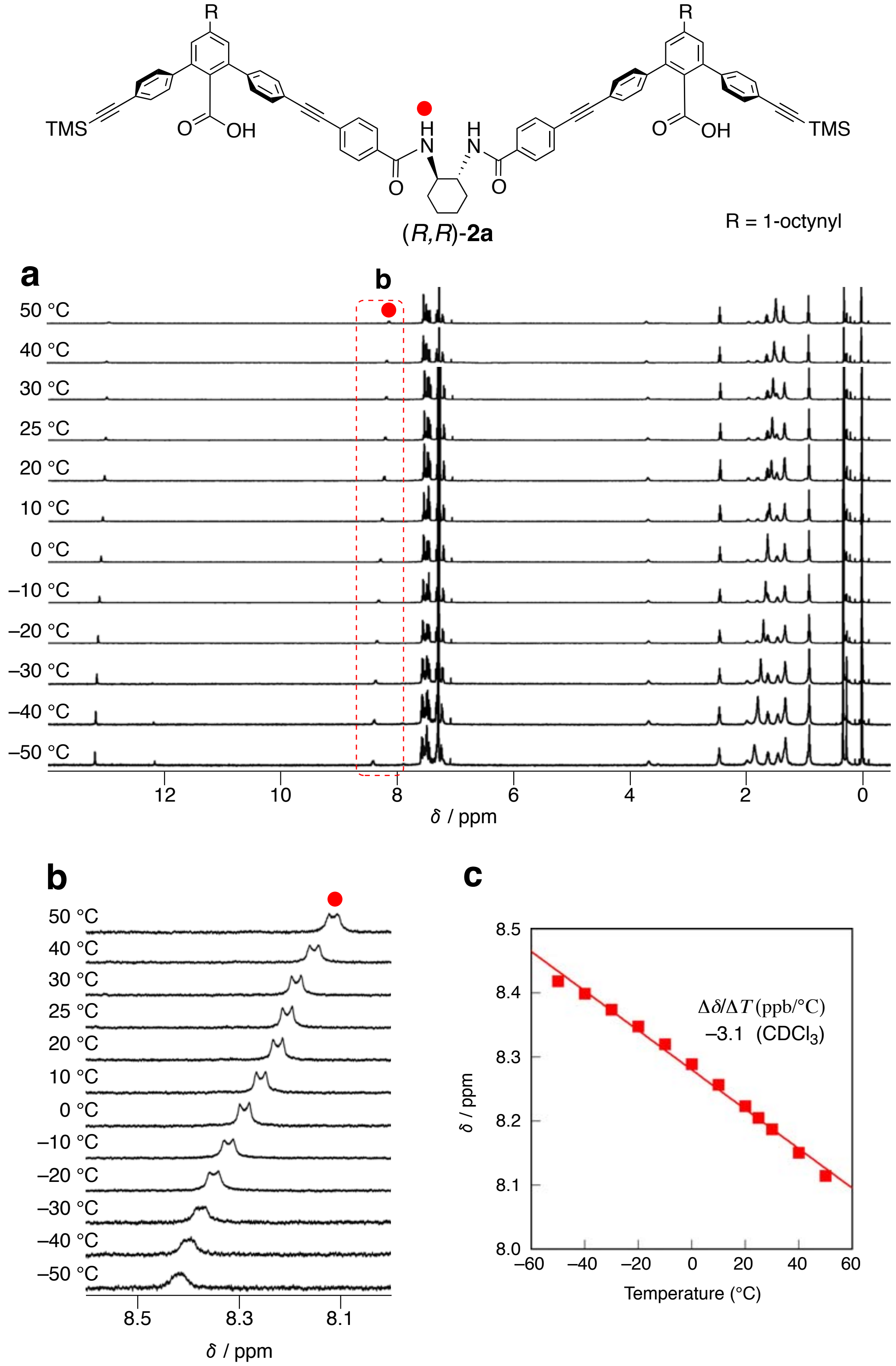

Supplementary Figure 7 | VT ^{1}H NMR spectra of (*R*,*R*)-2a in $CDCl_3$. Full (**a**) and partial (**b**) variable-temperature ^{1}H NMR spectra (500 MHz, 1.0 mM) of (*R*,*R*)-**2a** in $CDCl_3$ from –50 °C to 50 °C. (**c**) Plots of NH chemical shifts of (*R*,*R*)-**2a** in the ^{1}H NMR spectra versus temperature in $CDCl_3$.

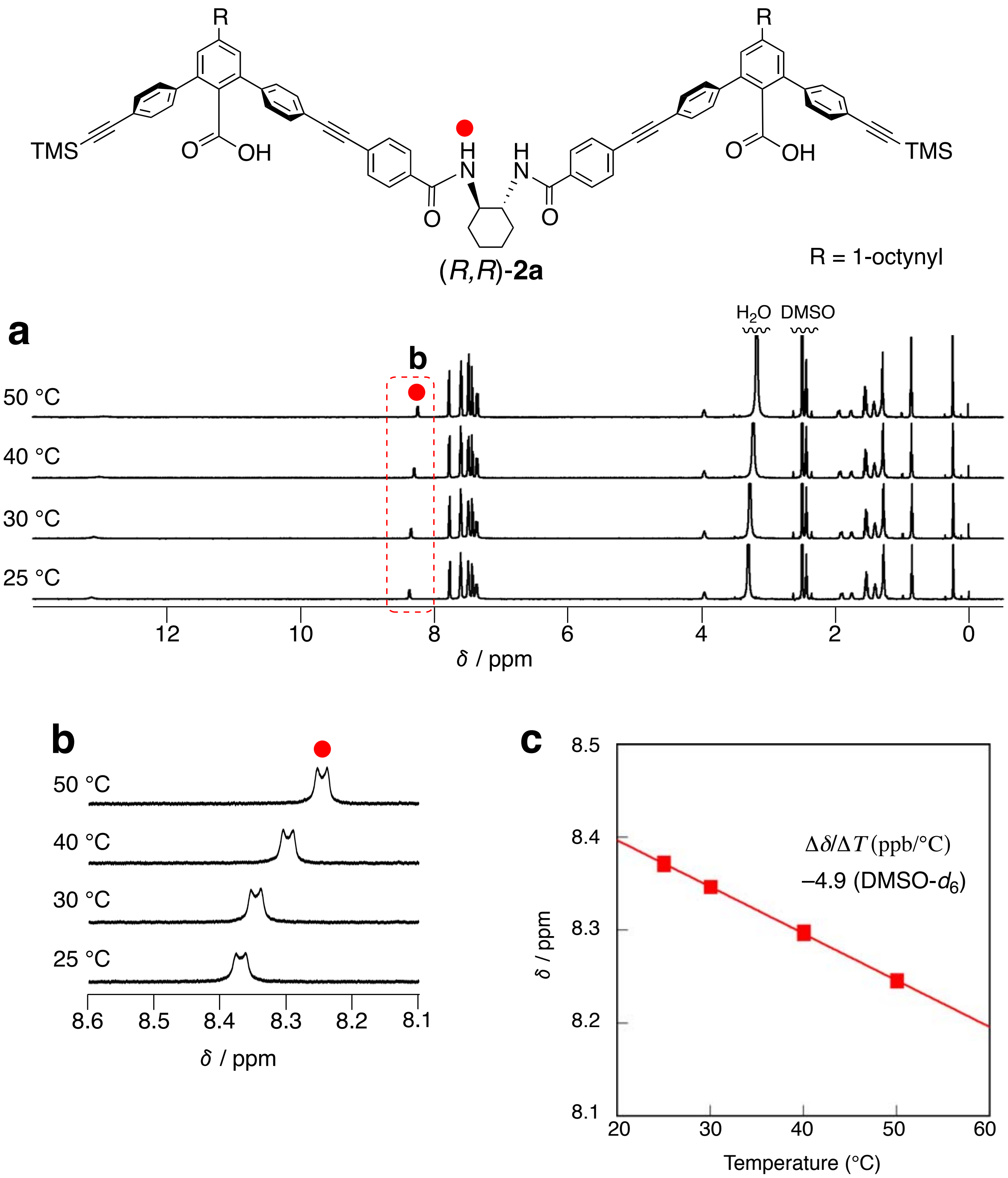

Supplementary Figure 8 | VT ^{1}H NMR spectra of (*R*,*R*)-2a in DMSO-*d*$_6$. Full (**a**) and partial (**b**) variable-temperature ^{1}H NMR spectra (500 MHz, 1.0 mM) of (*R*,*R*)-**2a** in DMSO-*d*$_6$ from 25 °C to 50 °C. (**c**) Plots of NH chemical shifts of (*R*,*R*)-**2a** in the ^{1}H NMR spectra versus temperature in DMSO-*d*$_6$.

a : (*R*,*R*)-1a

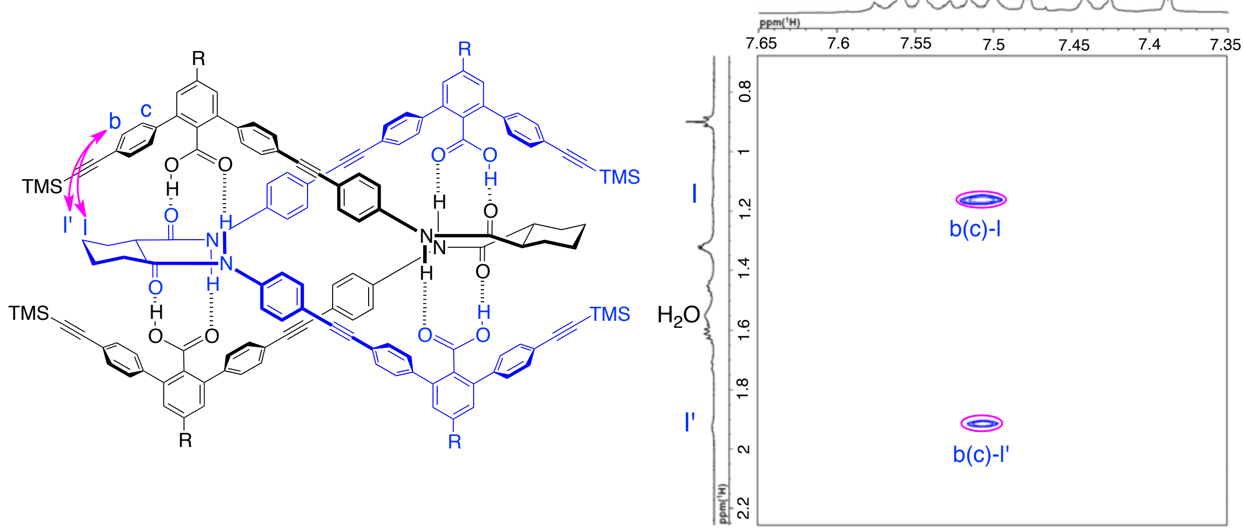

b : (*S*,*S*)-2a

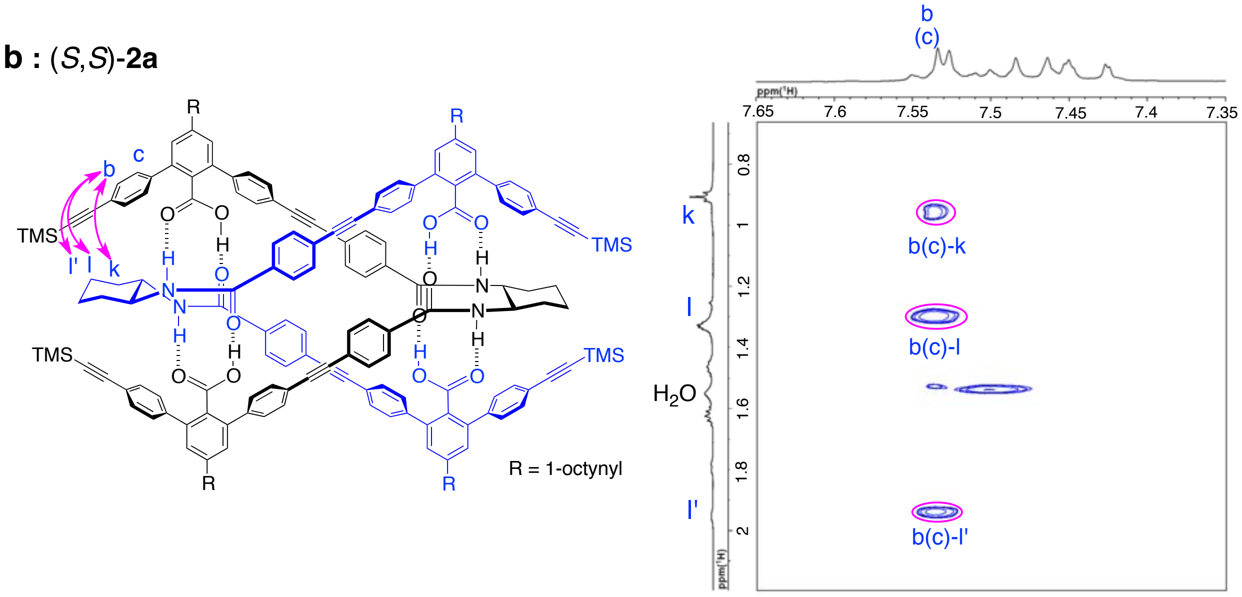

Supplementary Figure 9 | NOESY spectra of (*R*,*R*)-1a and (*S*,*S*)-2a in $CDCl_3$. Partial NOESY (500 MHz, $CDCl_3$, 25 °C, mixing time = 500 ms) spectra of (**a**) (*R*,*R*)-**1a** (2.0 mM) and (**b**) (*S*,*S*)-**2a** (2.0 mM). The proton resonances were assigned by the combination of *g*COSY and NOESY measurements (Supplementary Figs 10-13).

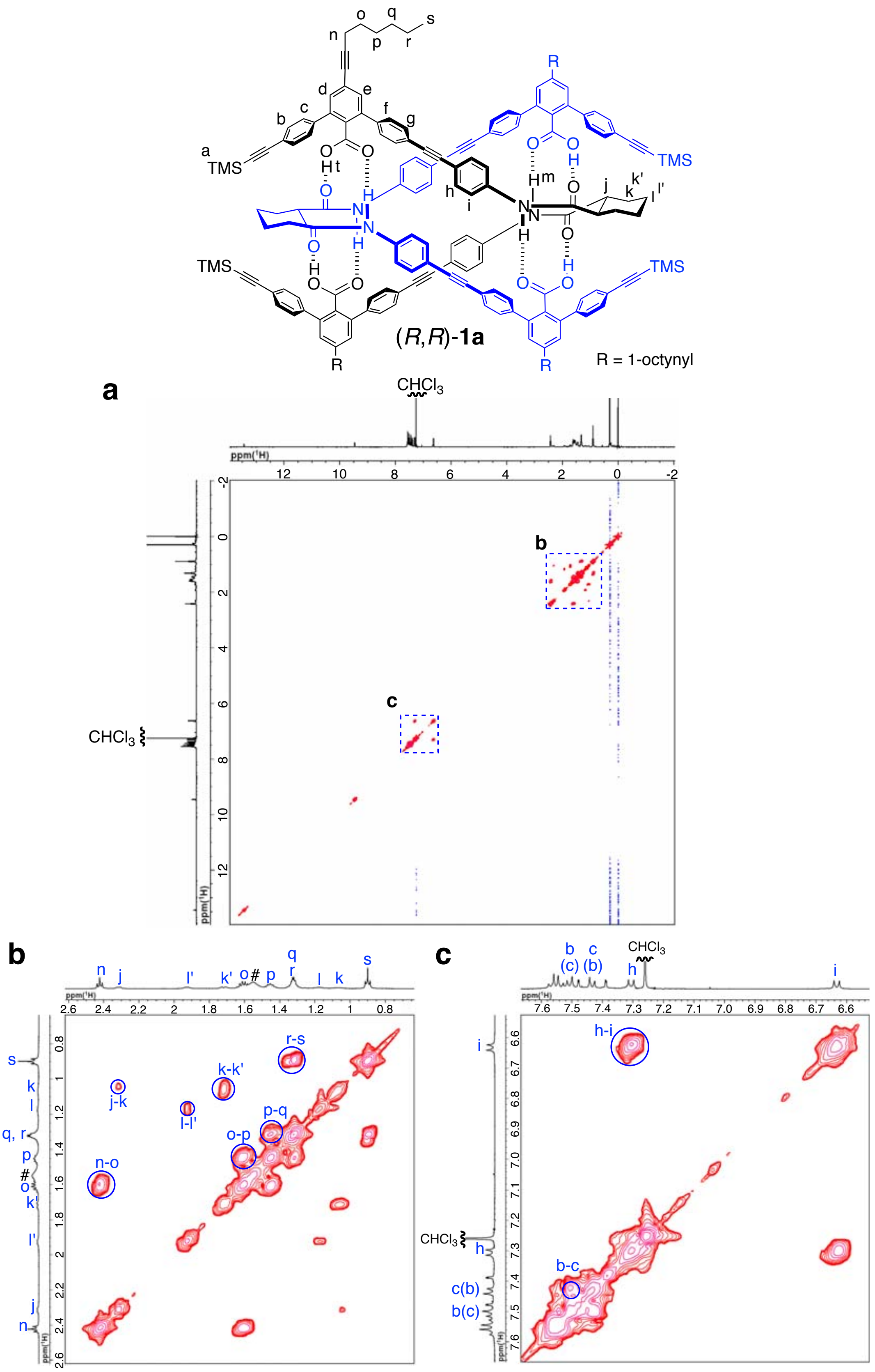

Supplementary Figure 10 | *g*COSY spectra of (*R*,*R*)-1a in $CDCl_3$. Full (a) and partial (b and c) *g*COSY (500 MHz, $CDCl_3$, 25 °C) spectra of (*R*,*R*)-**1a** (2.0 mM). # denotes the protons from H_2O.

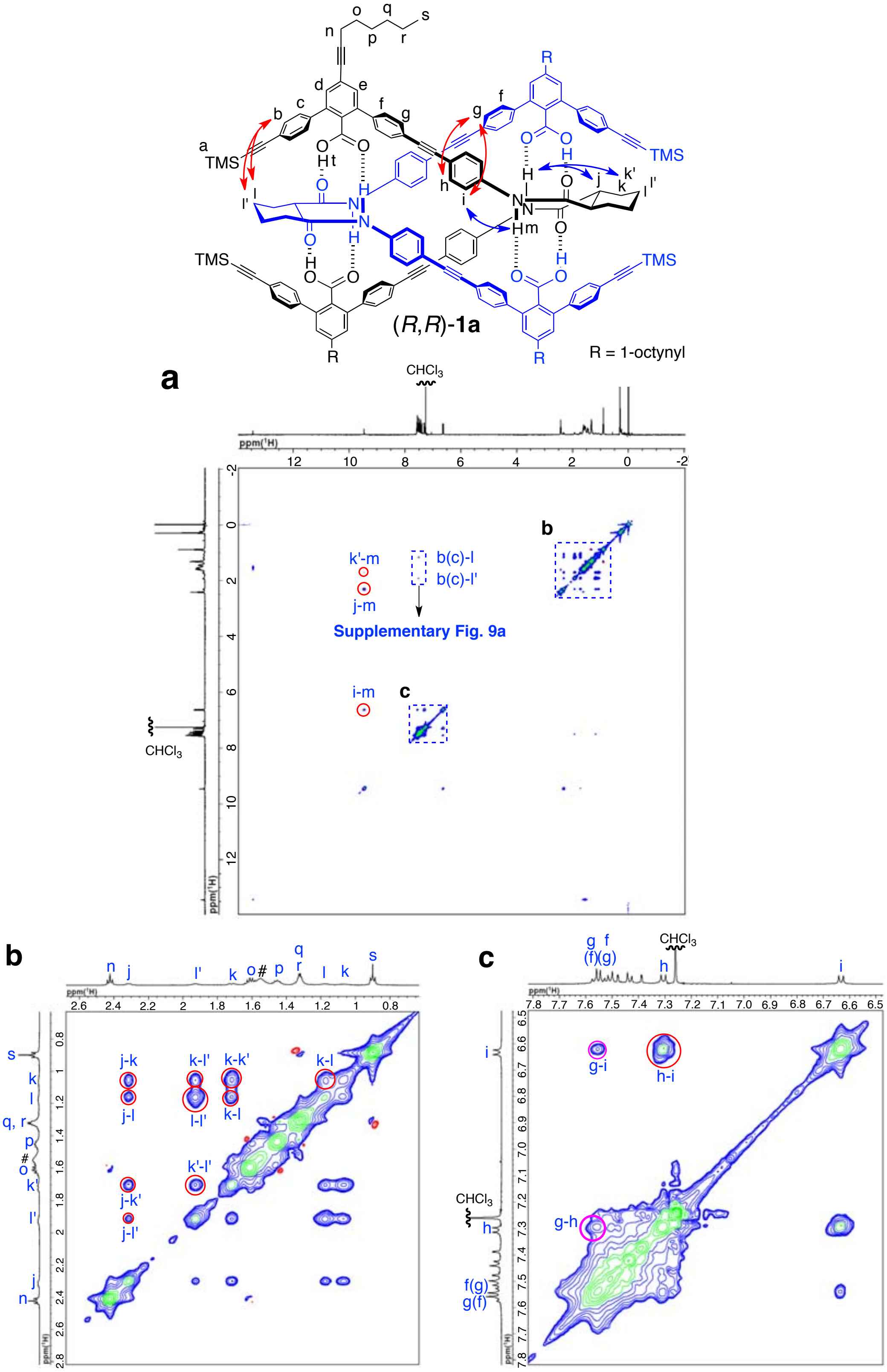

Supplementary Figure 11 | NOESY spectra of (*R*,*R*)-1a in CDCl$_3$. Full (**a**) and partial (**b** and **c**) NOESY (500 MHz, CDCl$_3$, 25 °C, mixing time = 500 ms) spectra of (*R*,*R*)-**1a** (2.0 mM). # denotes the protons from H_2O.

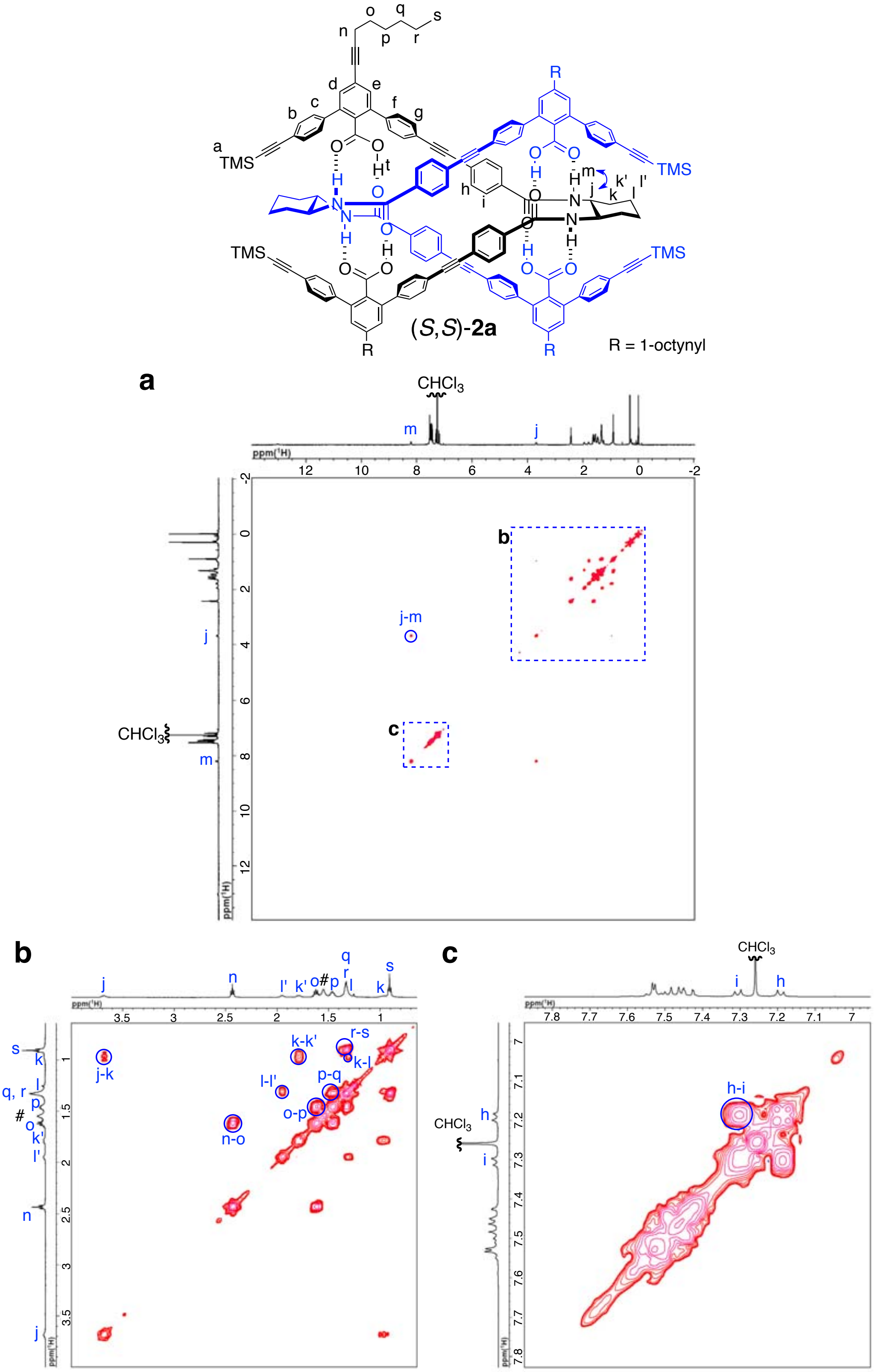

Supplementary Figure 12 | *g*COSY spectra of (*S*,*S*)-2a in $CDCl_3$. Full (**a**) and partial (**b** and **c**) *g*COSY (500 MHz, $CDCl_3$, 25 °C) spectrum of (*S*,*S*)-**2a** (2.0 mM). # denotes the protons from H_2O.

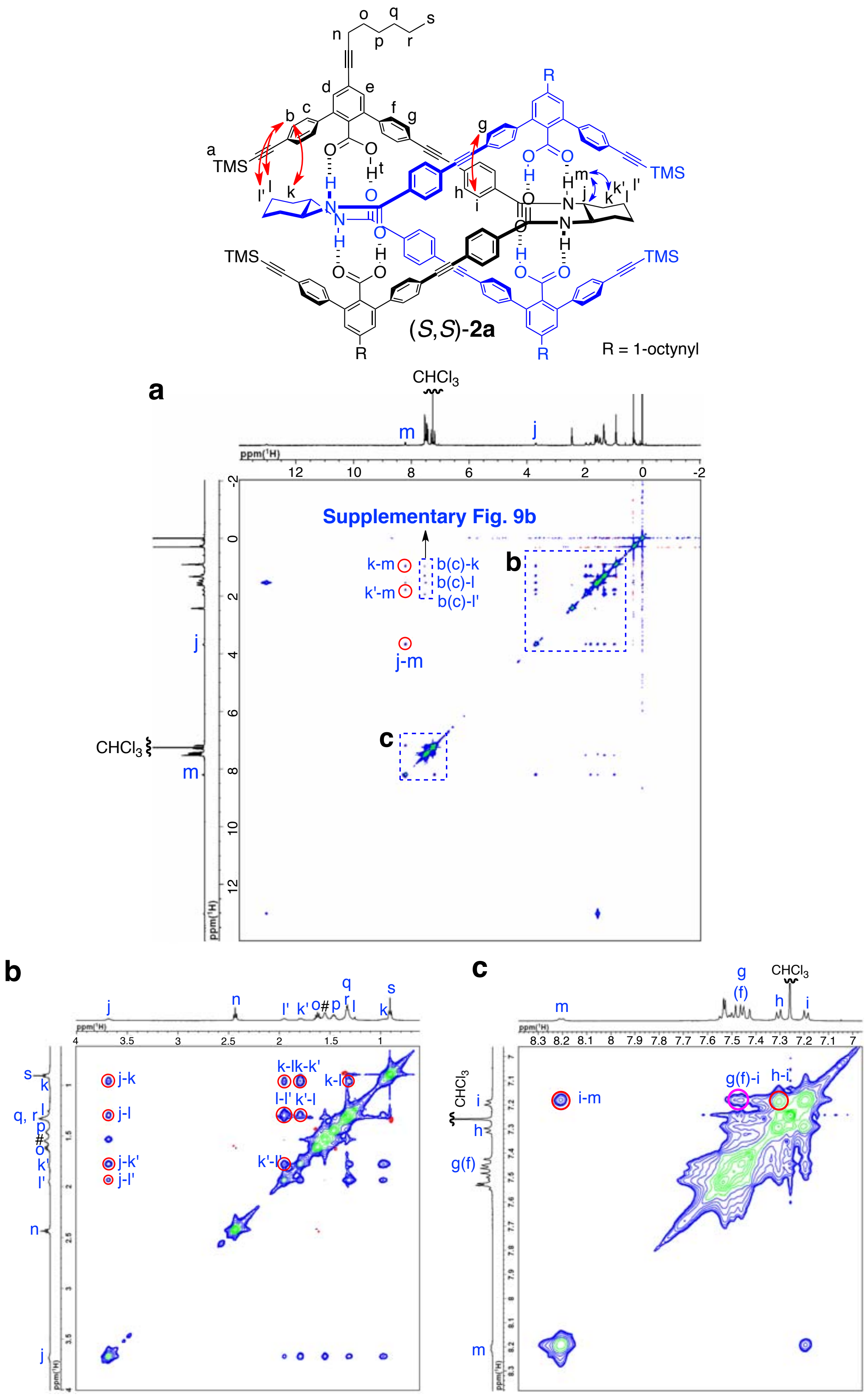

Supplementary Figure 13 | NOESY spectra of (*S*,*S*)-2a in $CDCl_3$. Full (**a**) and partial (**b** and **c**) NOESY (500 MHz, $CDCl_3$, 25 °C, mixing time = 500 ms) spectrum of (*S*,*S*)-**2a** (2.0 mM). # denotes the protons from H_2O.

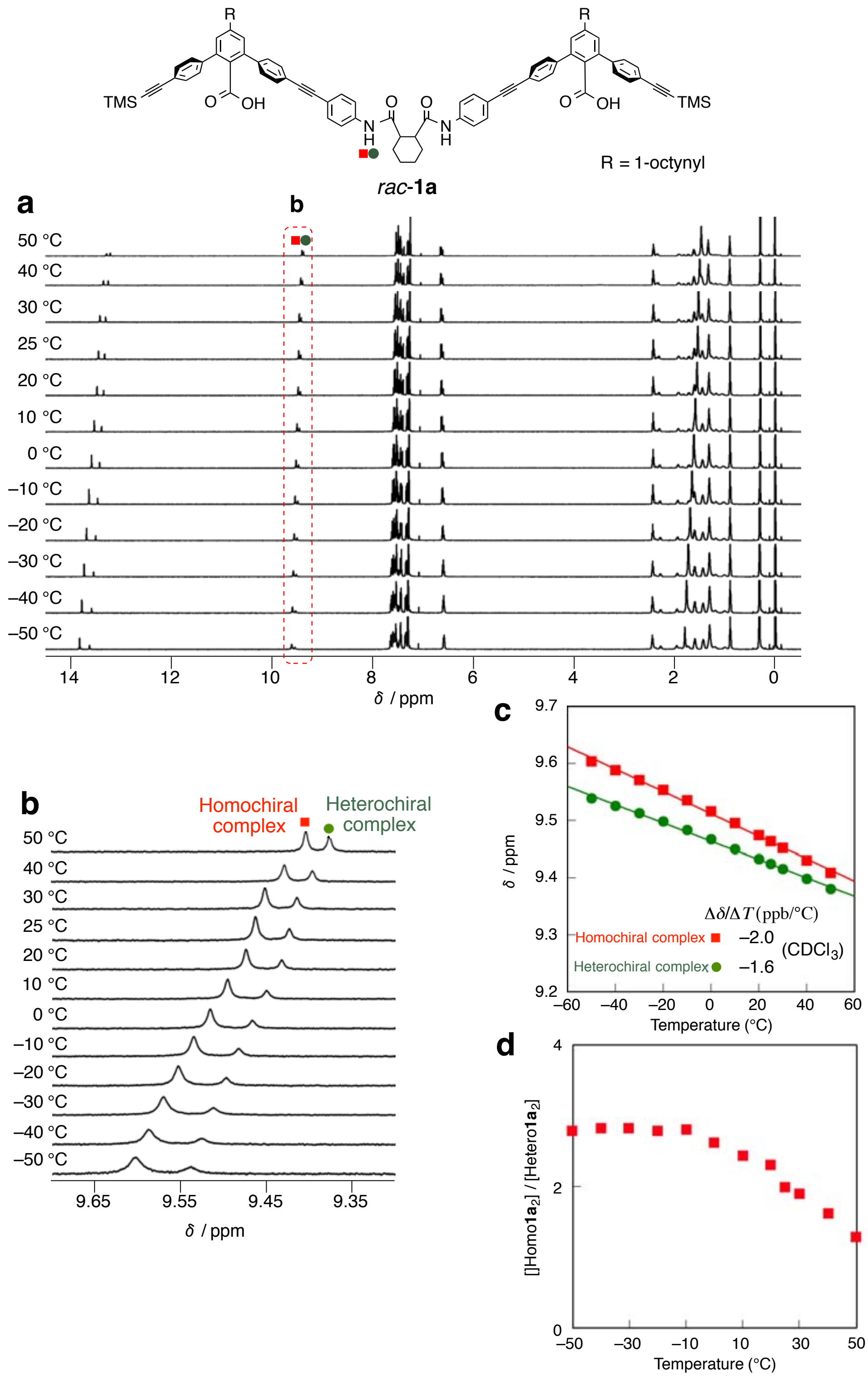

Supplementary Figure 14 | VT ^{1}H NMR spectra of *rac*-1a in $CDCl_3$. Full (**a**) and partial (**b**) variable-temperature ^{1}H NMR spectra (1.0 mM) of *rac*-**1a** in $CDCl_3$ from –50 °C to 50 °C. (**c**) Plots of NH chemical shifts of *rac*-**1a** in the ^{1}H NMR spectra versus temperature in $CDCl_3$. (**d**) Plots of ([homo**1a**$_2$]/[hetero**1a**$_2$]) versus temperature of *rac*-**1a** in $CDCl_3$.

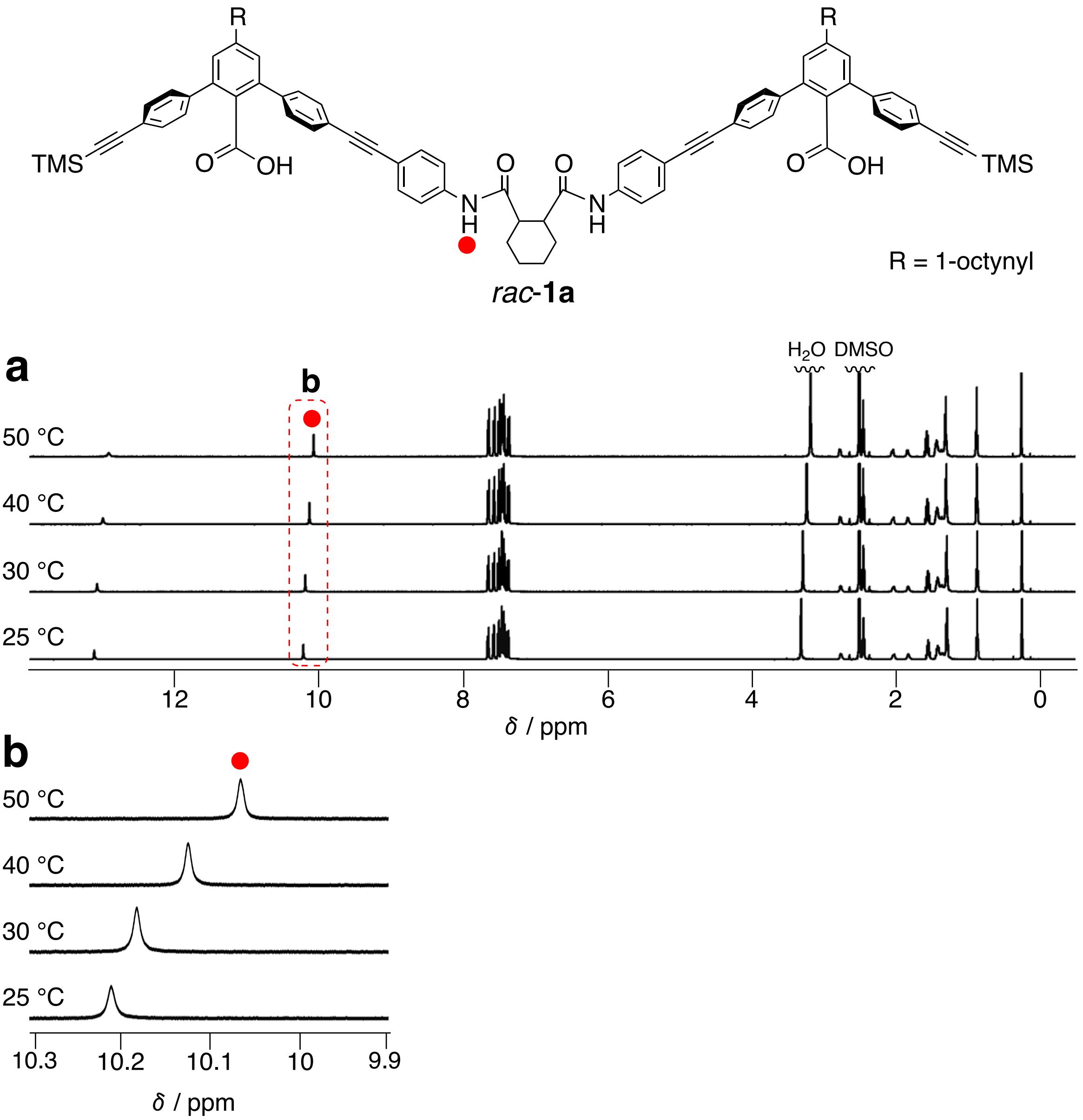

Supplementary Figure 15 | VT ^{1}H NMR spectra of *rac*-1a in DMSO-d_6. Full (**a**) and partial (**b**) variable-temperature ^{1}H NMR spectra (1.0 mM) of *rac*-**1a** in DMSO-d_6 from 25 °C to 50 °C.

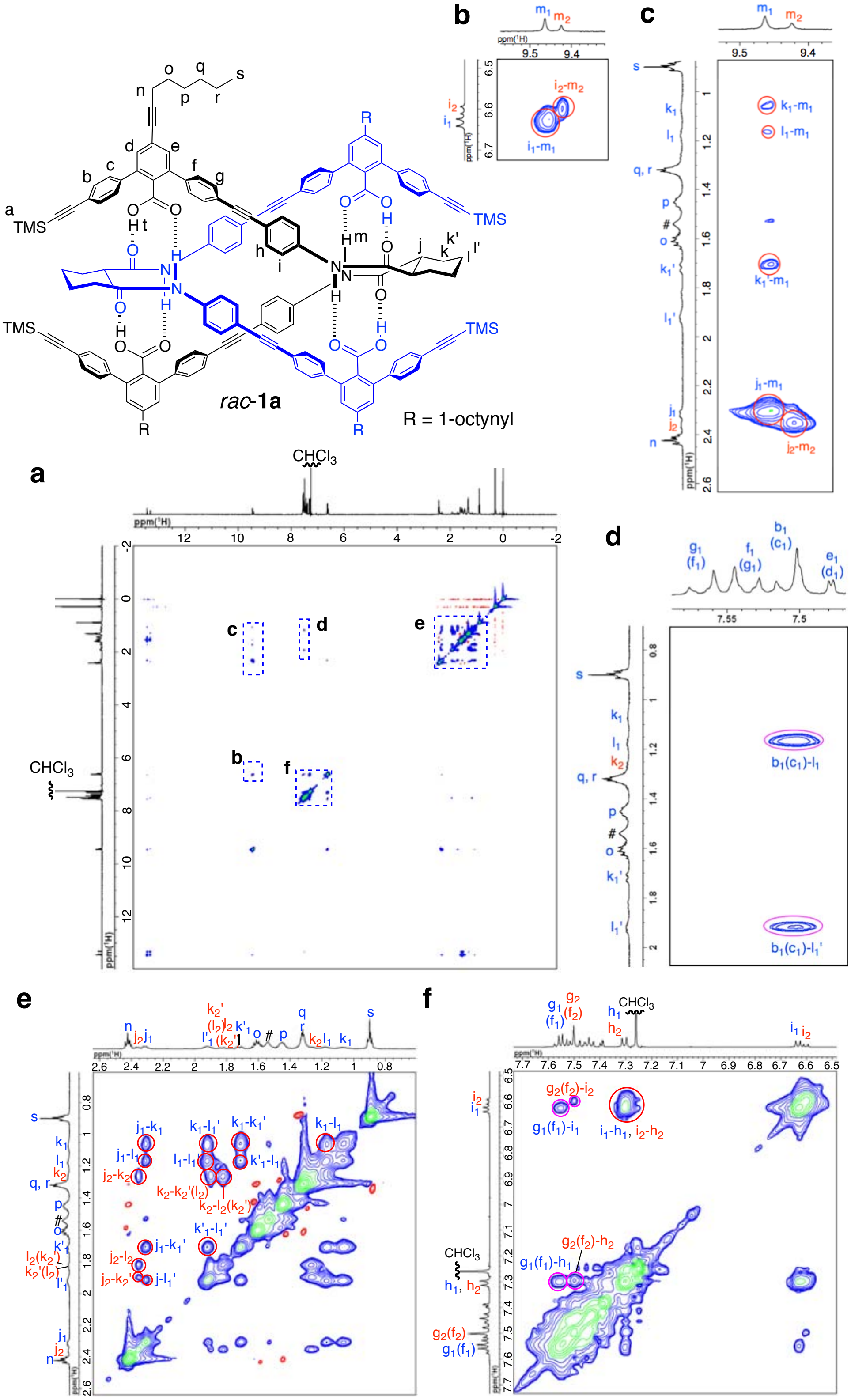

Supplementary Figure 16 | NOESY spectra of *rac*-1a in $CDCl_3$. Full (**a**) and partial (**b**, **c**, **d**, **e**, and **f**) NOESY (500 MHz, $CDCl_3$, 25 °C, mixing time = 500 ms) spectrum of *rac*-**1a** (2.0 mM), where the subscripts 1 and 2 denote the protons corresponding to the homochiral and heterochiral complexes, respectively. # denotes the protons from H_2O.

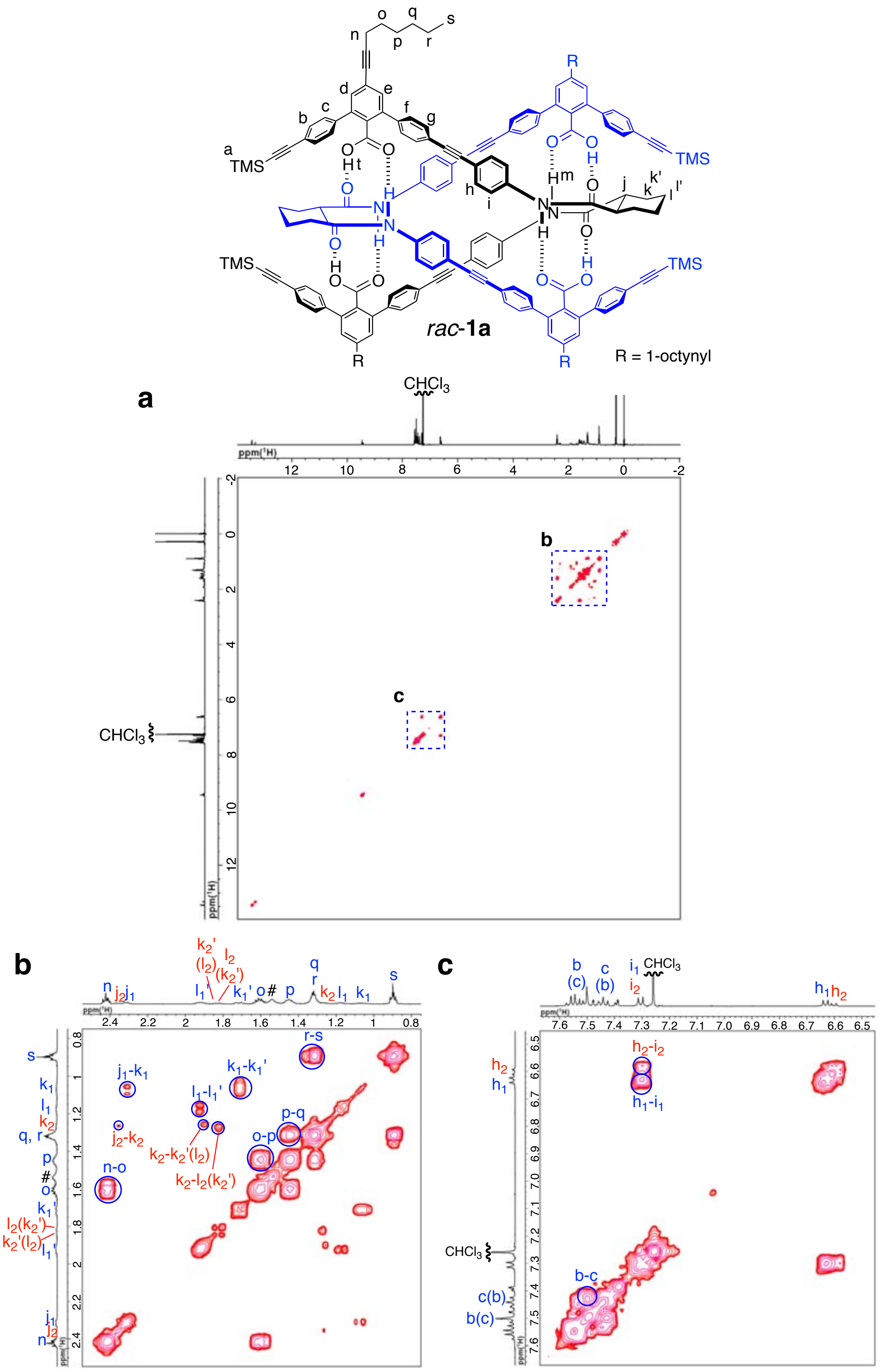

Supplementary Figure 17 | *g*COSY spectra of *rac*-1a in $CDCl_3$. Full (**a**) and partial (**b** and **c**) *g*COSY (500 MHz, $CDCl_3$, 25 °C) spectrum of *rac*-**1a** (2.0 mM), where the subscripts 1 and 2 denote the protons corresponding to the homochiral and heterochiral complexes, respectively. # denotes the protons from H_2O.

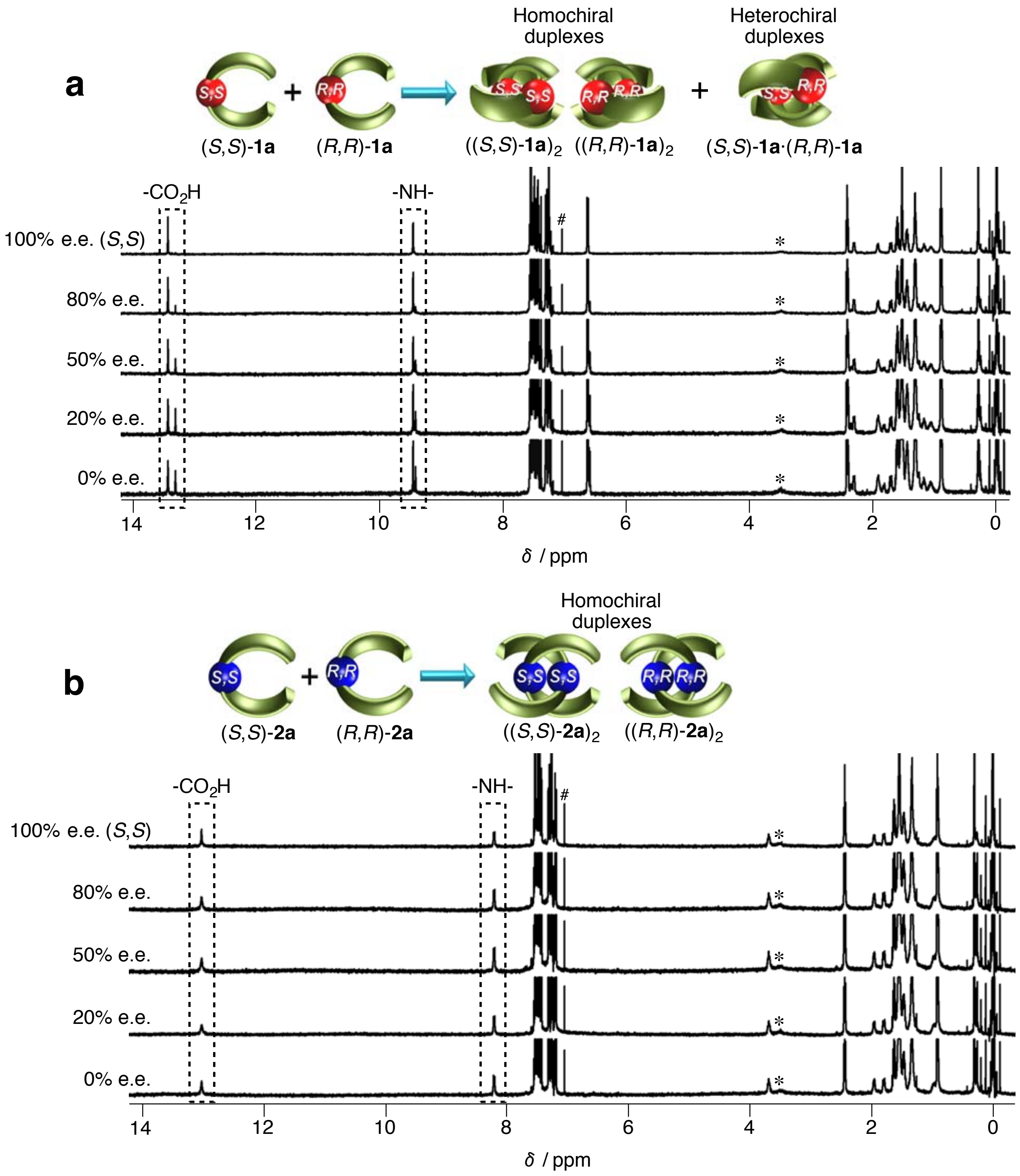

Supplementary Figure 18 | ^{1}H NMR spectra of 1a and 2a with different % e.e. in $CDCl_3$. Full ^{1}H NMR spectra of **1a** (**a**) and **2a** (**b**) (0.50 mM) with different % e.e. in $CDCl_3$ at 25 °C (see Fig. 3a and c for expanded spectra of CO_2H and amide NH proton resonance regions, respectively). The asterisks denote impurities contained in the solvent $CDCl_3$.

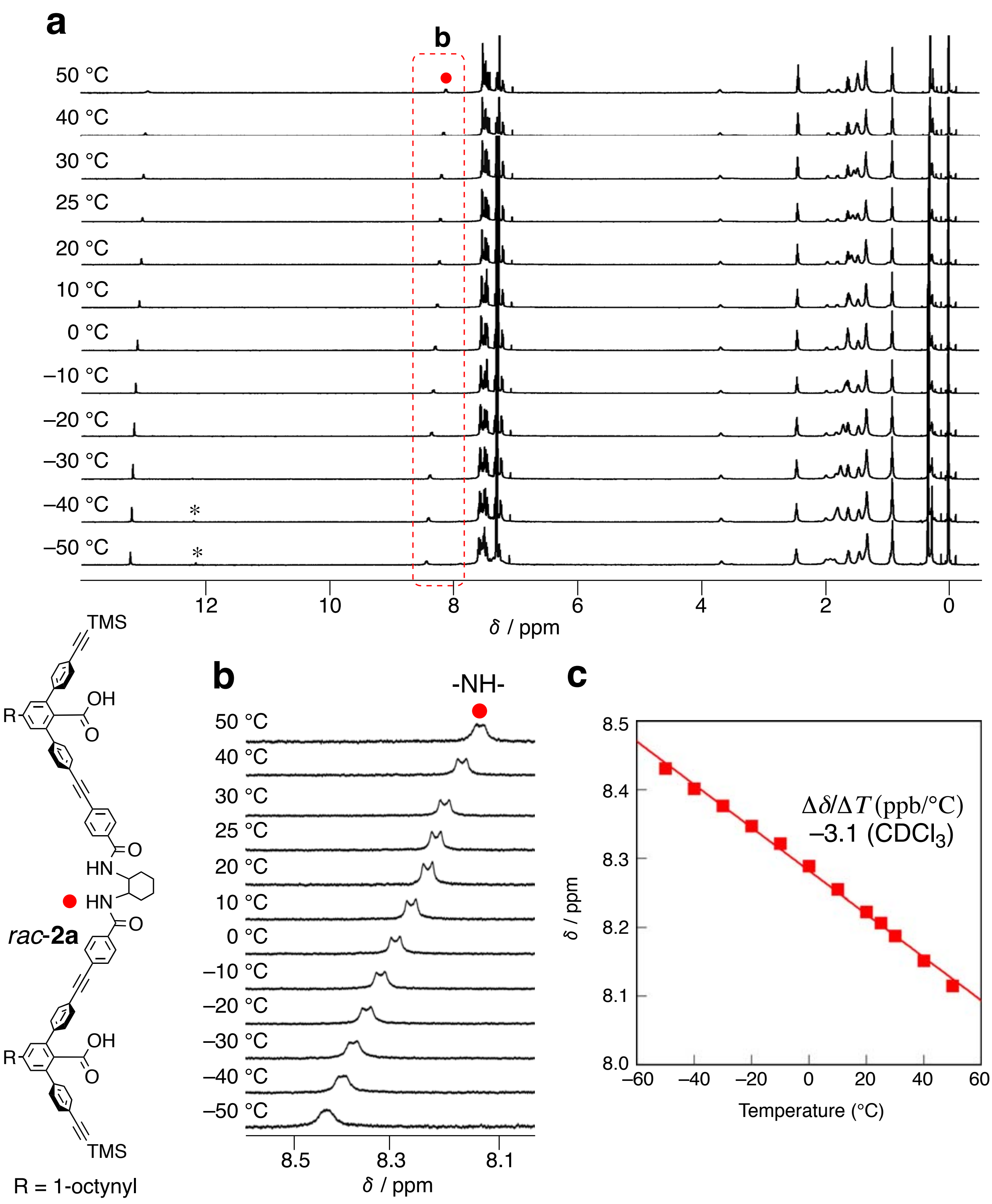

Supplementary Figure 19 | VT ^{1}H NMR spectra of *rac*-2a in $CDCl_3$. Full (**a**) and partial (**b**) variable-temperature ^{1}H NMR spectra (1.0 mM) of *rac*-**2a** in $CDCl_3$ from –50 °C to 50 °C. (**c**) Plots of NH chemical shifts of *rac*-**2a** in the ^{1}H NMR spectra versus temperature in $CDCl_3$. A very small peak marked by * appeared at low temperatures (below –30 °C), which was also observed for enantiopure (*R*,*R*)-**2a** at below –30 °C (Supplementary Fig. 7a). These minor peaks are most likely due to a minor conformer resulting from slow rotation of phenyl rings.

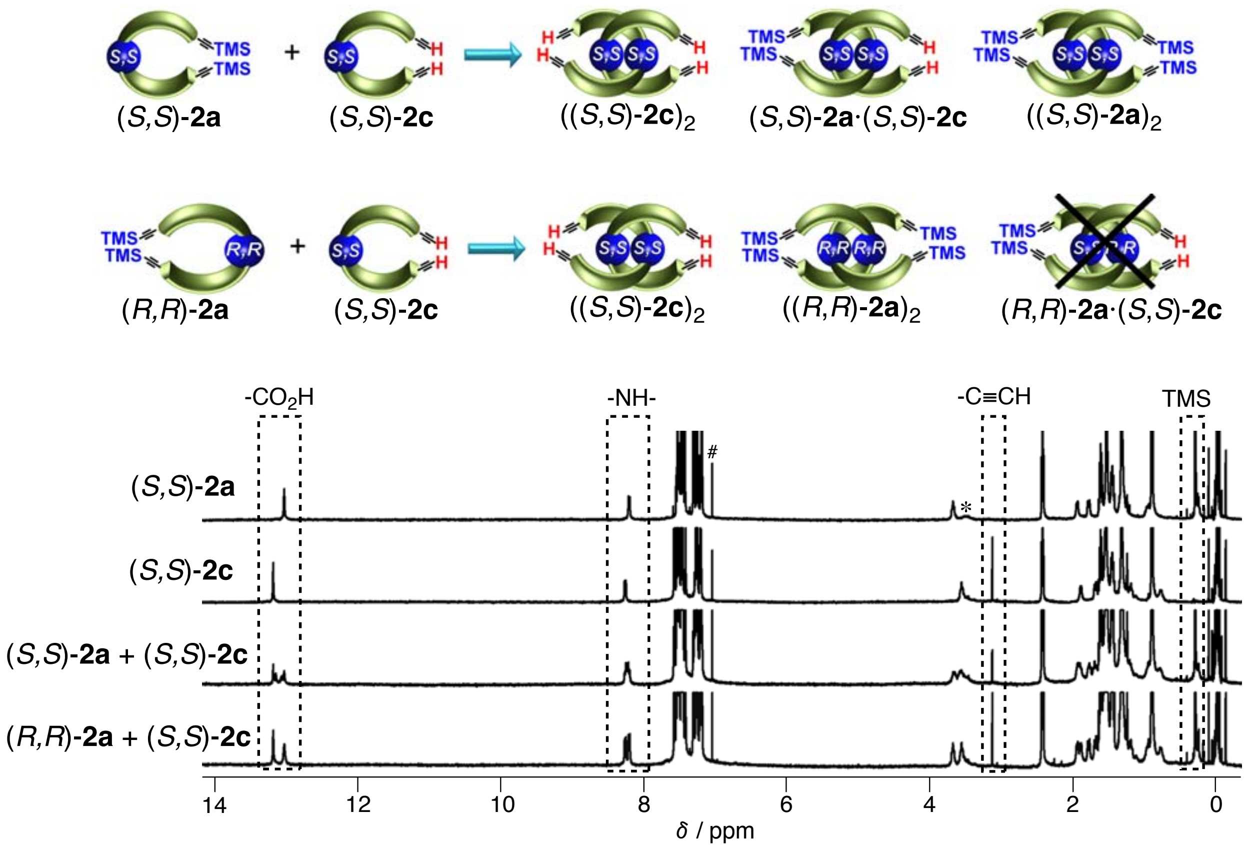

Supplementary Figure 20 | Chiral self-sorting behavior of *rac*-1a and *rac*-2a. Full ^{1}H NMR spectra (0.50 mM) of (*S*,*S*)-**2a**, (*S*,*S*)-**2c**, an equimolar mixture (1.0 mM) of (*S*,*S*)-**2a** and (*S*,*S*)-**2c**, and an equimolar mixture (1.0 mM) of (*R*,*R*)-**2a** and (*S*,*S*)-**2c** in $CDCl_3$ at 25 °C (see Fig. 3d for expanded spectra of CO_2H, amide NH, C≡CH, and TMS proton resonance regions, respectively). The asterisks denote impurities contained in the solvent $CDCl_3$.

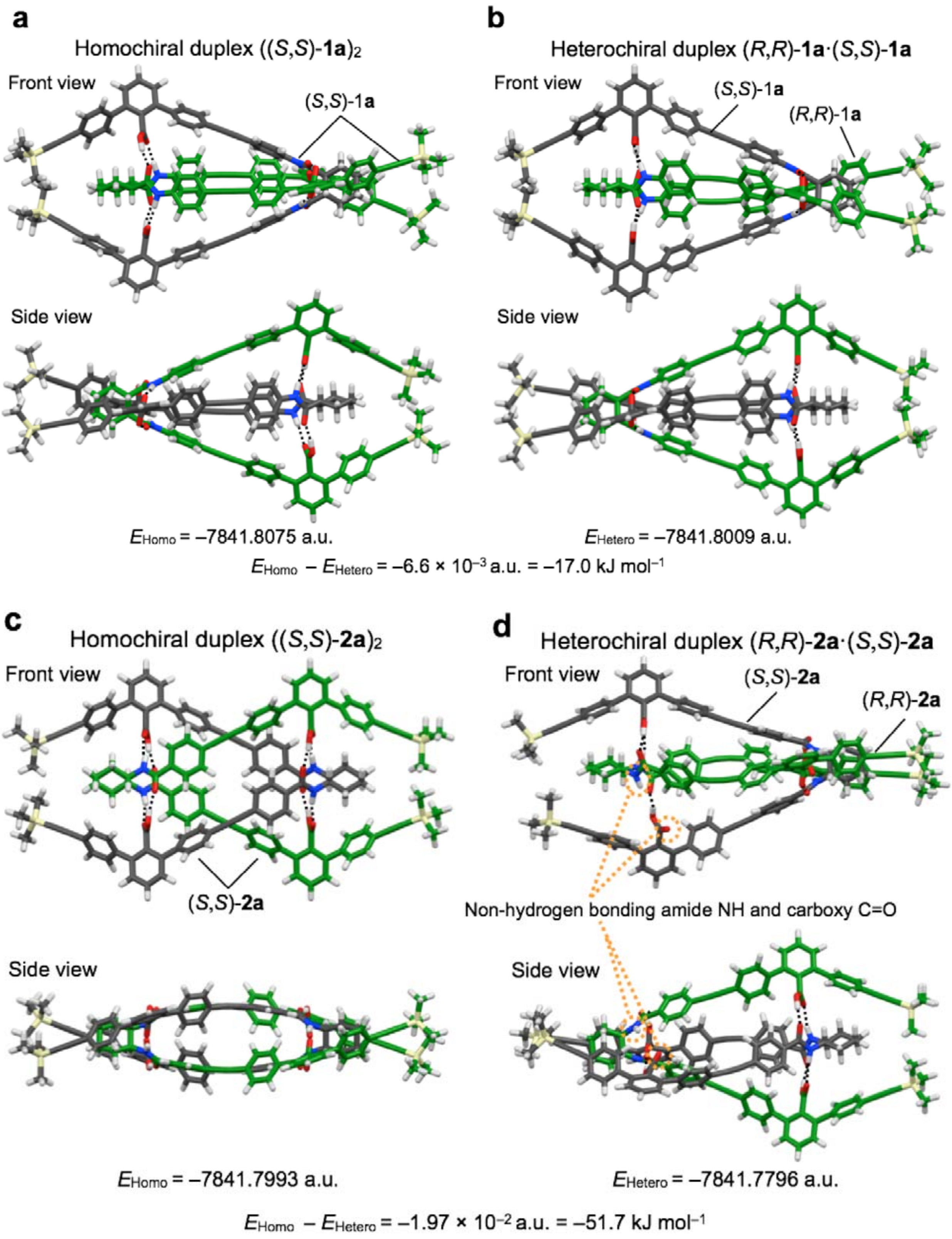

(Supplementary Fig. 21 to be continued)

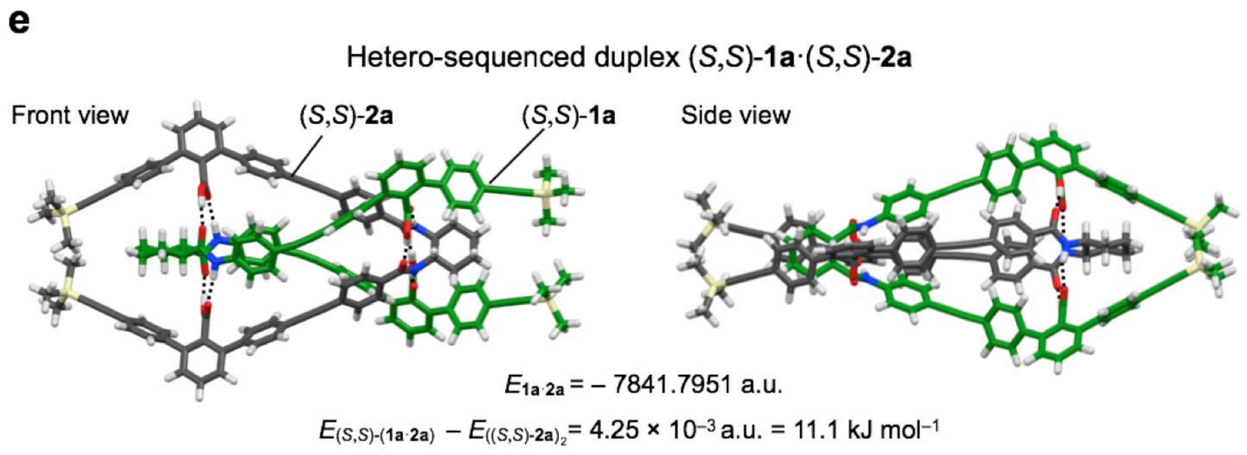

Supplementary Figure 21 | Theoretical studies on the structures of the homochiral duplexes ((*S*,*S*)-1a)$_2$ and ((*S*,*S*)-2a)$_2$, heterochiral duplexes (*S*,*S*)-1a•(*R*,*R*)-1a and (*S*,*S*)-2a•(*R*,*R*)-2a, and hetero-sequenced duplex (*S*,*S*)-1a•(*S*,*S*)-2a. Capped-stick drawings of the structures for the homochiral duplexes ((*S*,*S*)-**1a**)$_2$ (**a**) and ((*S*,*S*)-**2a**)$_2$ (**c**), heterochiral duplexes (*R*,*R*)-**1a**·(*S*,*S*)-**1a** (**b**), (*R*,*R*)-**2a**·(*S*,*S*)-**2a** (**d**), and hetero-sequenced duplex (*S*,*S*)-**1a**·(*S*,*S*)-**2a** (**e**) optimized by DFT calculations. DFT calculated energies are also shown in the bottom.

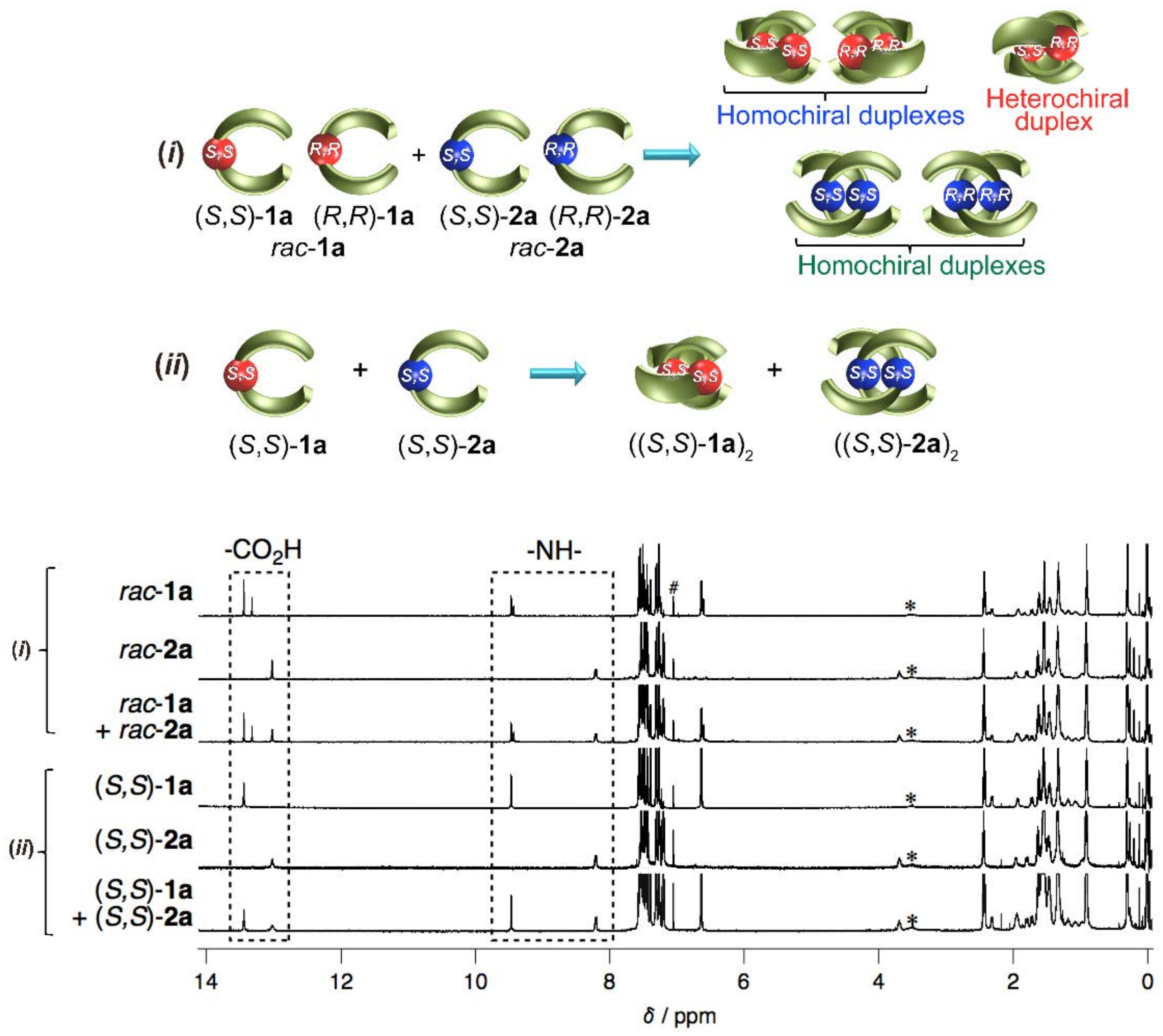

Supplementary Figure 22 | Sequence-selective chiral self-sorting behavior of *rac*-1a and *rac*-2a. Full ^{1}H NMR spectra of *rac*-**1a** (1.0 mM), *rac*-**2a** (1.0 mM), an equimolar mixture (1.0 mM) of *rac*-**1a** and *rac*-**2a**, (*S*,*S*)-**1a** (0.50 mM), (*S*,*S*)-**2a** (0.50 mM), and an equimolar mixture (1.0 mM) of (*S*,*S*)-**1a** and (*S*,*S*)-**2a** in $CDCl_3$ at 25 °C (see Fig. 4a for expanded CO_2H and NH amide proton resonance regions). The asterisk denotes impurities contained in the solvent $CDCl_3$.

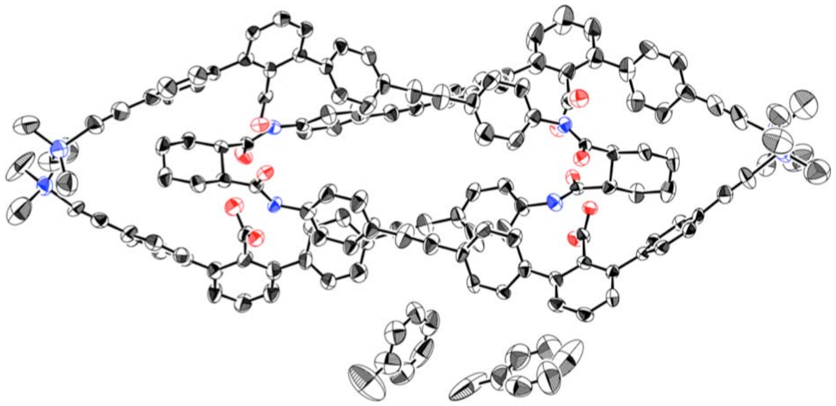

Supplementary Figure 23 | The crystal structure of (*S*,*S*)-1a'. ORTEP drawing of the crystal structure of (*S*,*S*)-**1a'** with thermal ellipsoids at 50% probability.

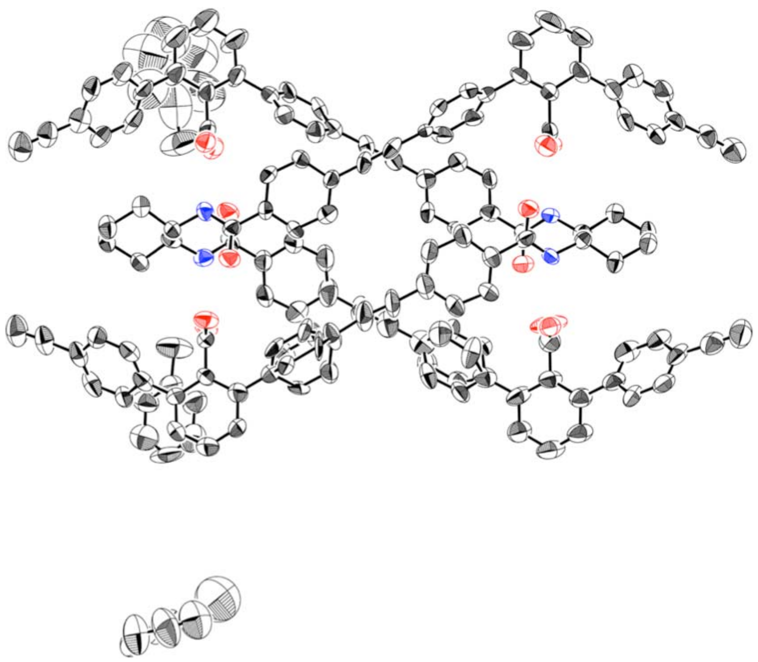

Supplementary Figure 24 | The crystal structure of (*S*,*S*)-2a'. ORTEP drawing of the crystal structure of (*S*,*S*)-**2a**' with thermal ellipsoids at 50% probability.

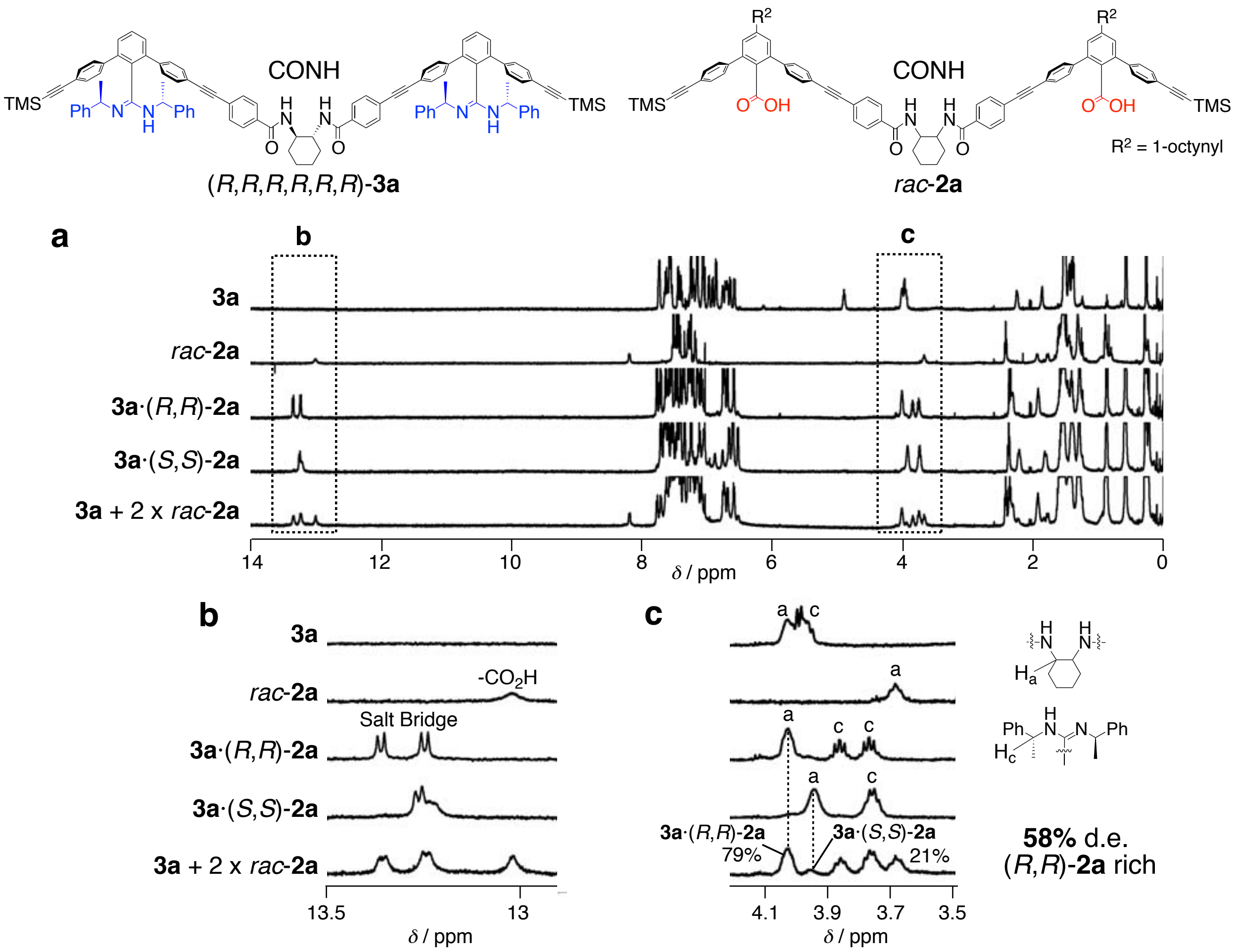

Supplementary Figure 25 | Diastereoselective duplex formation between $3a_{CONH}$ and *rac*-$2a_{CONH}$ (run 1, Table 1). Full (**a**) and partial (**b** and **c**) ^{1}H NMR spectra of **3a** (0.50 mM), *rac*-**2a** (0.50 mM), **3a**·(*R*,*R*)-**2a** (0.50 mM), **3a**·(*S*,*S*)-**2a** (0.50 mM), and a mixture of **3a** (0.50 mM) and *rac*-**2a** (1.0 mM) in $CDCl_3$ at 25 °C.

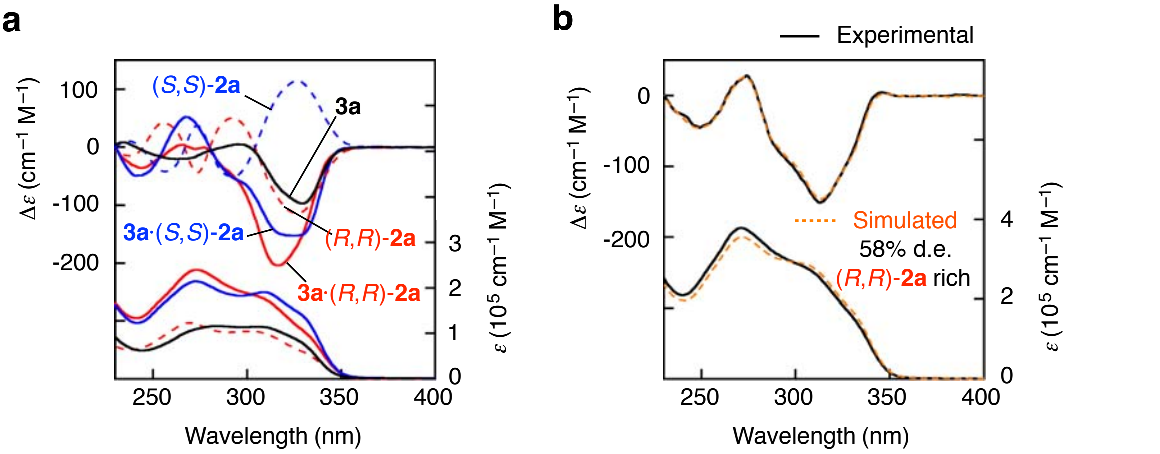

Supplementary Figure 26 | Diastereoselective duplex formation between $3a_{CONH}$ and *rac*-$2a_{CONH}$. (**a**) CD and absorption spectra (0.50 mM) of **3a**, (*R*,*R*)-**2a**, (*S*,*S*)-**2a**, **3a**·(*R*,*R*)-**2a**, and **3a**·(*S*,*S*)-**2a** in $CDCl_3$ at ambient temperature. (**b**) Experimental and simulated CD (d.e. = 58%) and absorption spectra for a mixture of **3a** (0.50 mM) and *rac*-**2a** (1.0 mM) in $CDCl_3$ at ambient temperature.

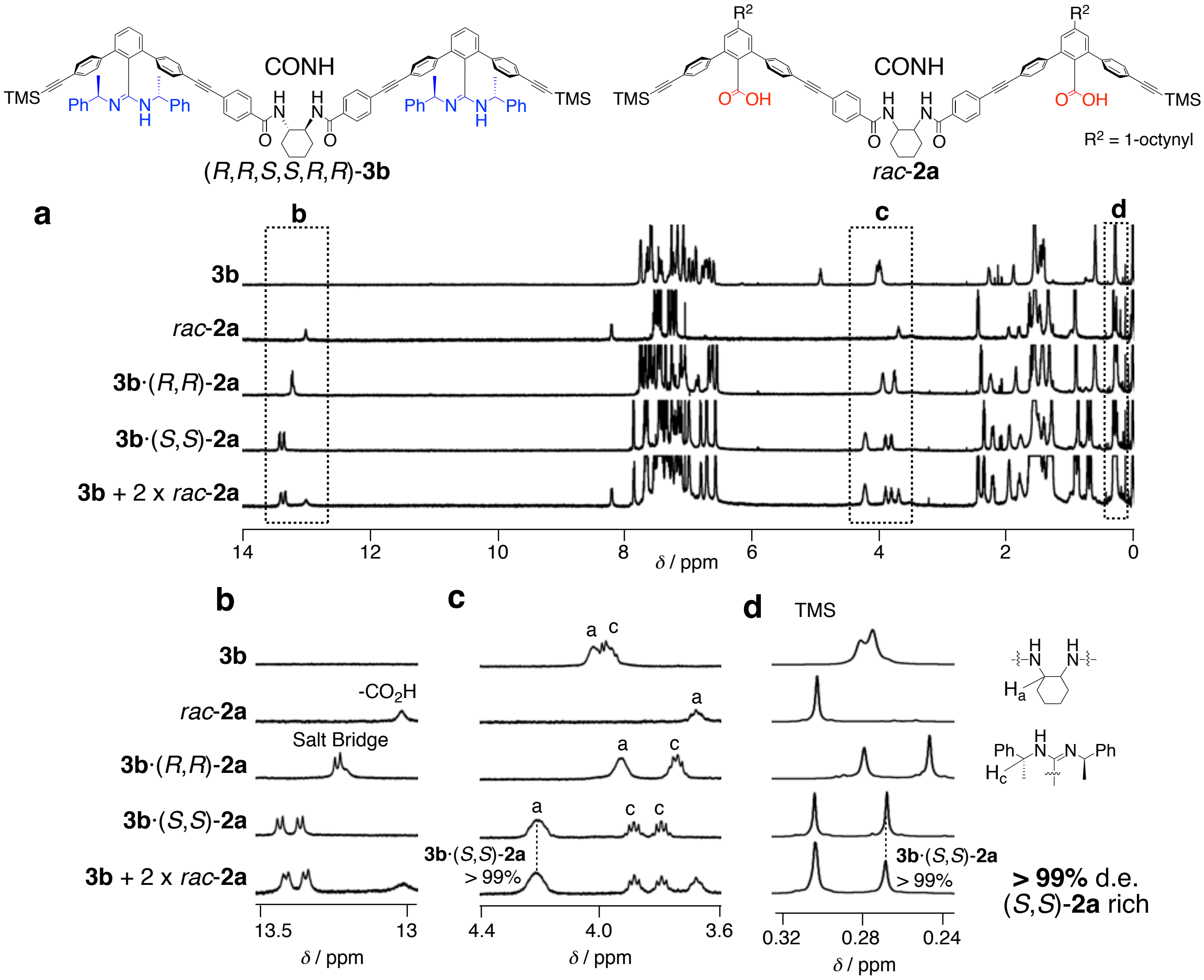

Supplementary Figure 27 | Diastereoselective duplex formation between $\mathbf{3b}_{\mathrm{CONH}}$ and *rac*-$\mathbf{2a}_{\mathrm{CONH}}$ (run 2, Table 1). Full (**a**) and partial (**b** and **c**) ^{1}H NMR spectra of **3b** (0.50 mM), *rac*-**2a** (0.50 mM), **3b**·(*R*,*R*)-**2a** (0.50 mM), **3b**·(*S*,*S*)-**2a** (0.50 mM), and a mixture of **3b** (0.50 mM) and *rac*-**2a** (1.0 mM) in $CDCl_3$ at 25 °C (see also Fig. 5a).

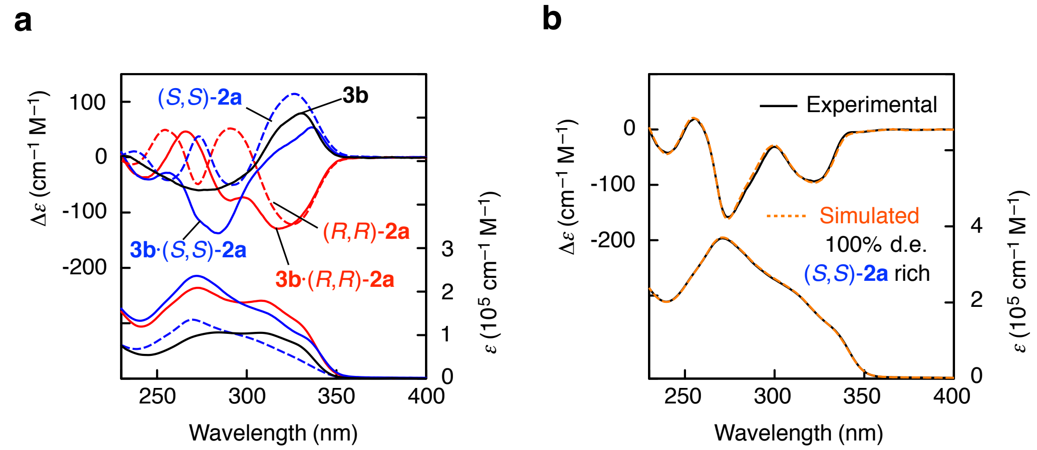

Supplementary Figure 28 | Diastereoselective duplex formation between $\mathbf{3b}_{\mathrm{CONH}}$ and *rac*-$\mathbf{2a}_{\mathrm{CONH}}$. (**a**) CD and absorption spectra (0.50 mM) of **3b**, (*R*,*R*)-**2a**, (*S*,*S*)-**2a**, **3b**·(*R*,*R*)-**2a**, and **3b**·(*S*,*S*)-**2a** in $CDCl_3$ at ambient temperature. (**b**) Experimental and simulated CD (d.e. = 100%) and absorption spectra for a mixture of **3b** (0.50 mM) and *rac*-**2a** (1.0 mM) in $CDCl_3$ at ambient temperature.

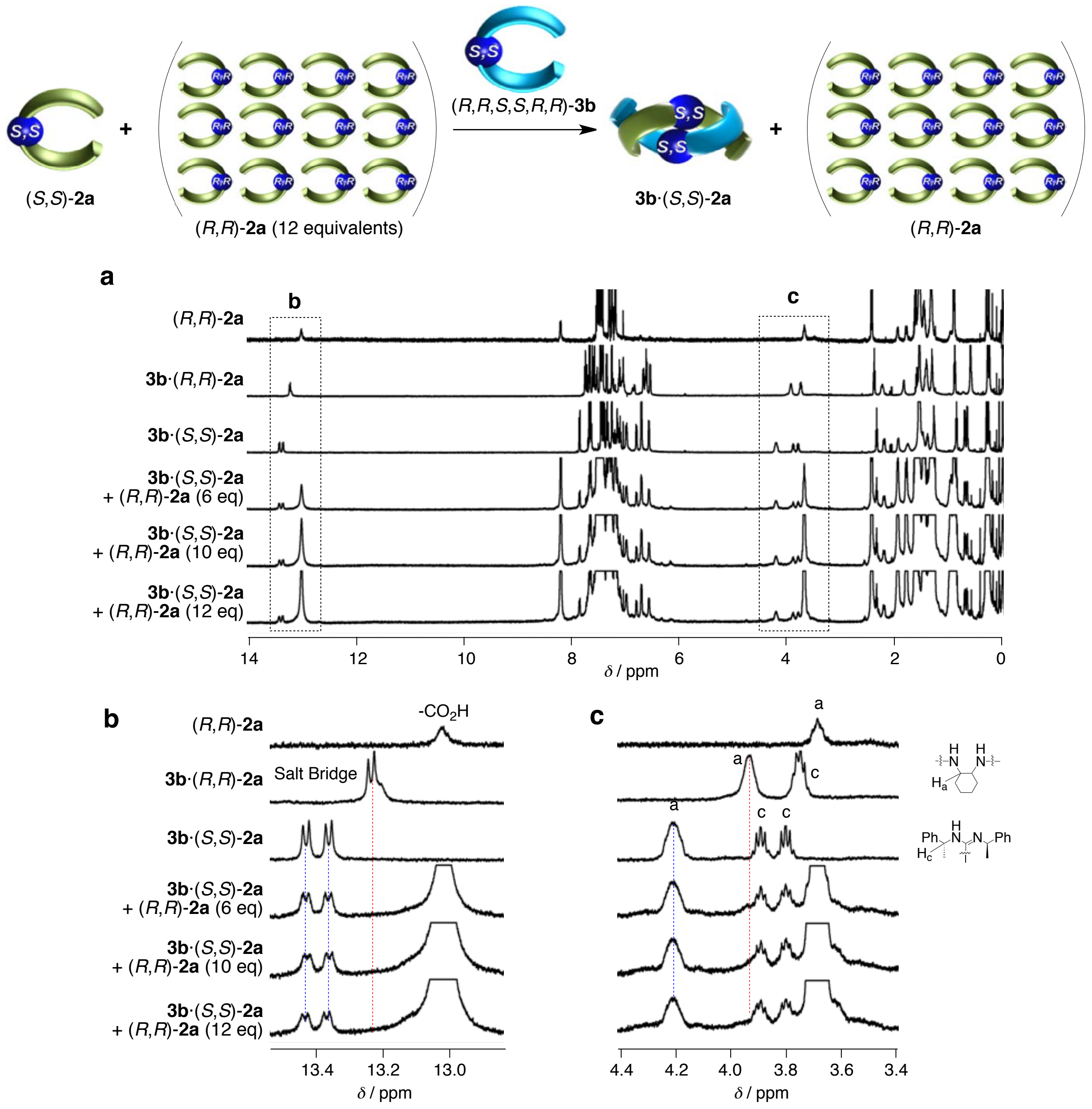

Supplementary Figure 29 | Diastereoselective duplex formation between $\mathbf{3b}_{\mathrm{CONH}}$ and (*S*,*S*)-$\mathbf{2a}_{\mathrm{CONH}}$ in the presence of a large excess (*R*,*R*)-$\mathbf{2a}_{\mathrm{CONH}}$. Full (**a**) and partial (**b**) ^{1}H NMR spectra of (*R*,*R*)-**2a** (0.50 mM), **3b**·(*R*,*R*)-**2a** (0.50 mM), **3b**·(*S*,*S*)-**2a** (0.50 mM), and mixtures of **3b** (0.50 mM) and (*S*,*S*)-**2a** (0.50 mM) in the presence of an excess amount of (*R*,*R*)-**2a** (3.0, 5.0, and 6.0 mM, respectively) in $CDCl_3$ at 25 °C. (*R*,*R*,*S*,*S*,*R*,*R*)-**3b** formed a duplex in an extremely high diastereoselectivity with (*S*,*S*)-**2a** even in the presence of 12 equivalents of (*R*,*R*)-**2a**.

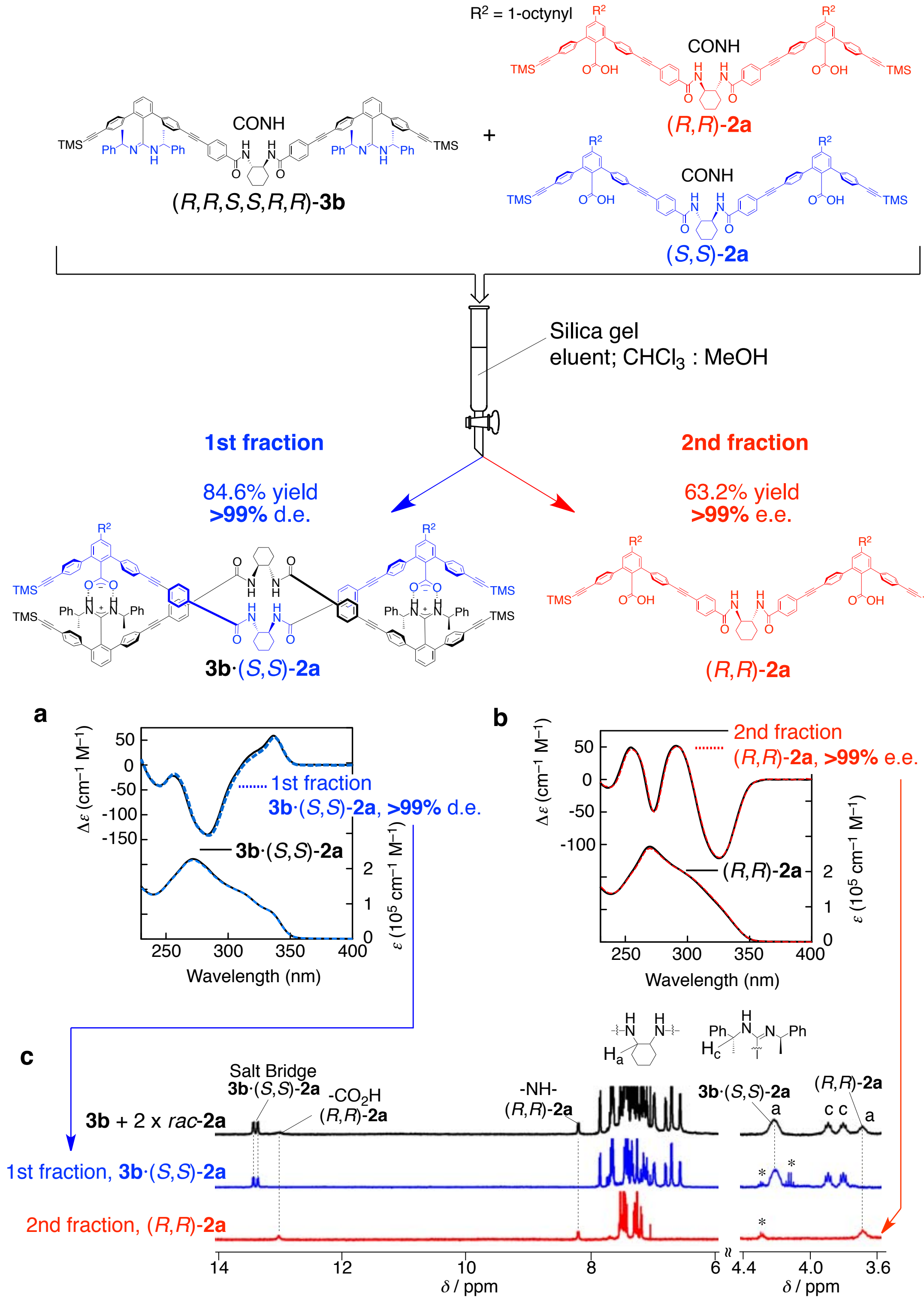

Supplementary Figure 30 | Optical resolution of *rac*-2a via complementary duplex formation. CD and absorption spectra of (**a**) **3b**·(*S*,*S*)-**2a** (1st fraction, 0.44 mM) separated from a mixture of **3b** and 2 equivalents of *rac*-**2a** and (**b**) (*R*,*R*)-**2a** (2nd fraction, 0.48 mM) separated from a mixture of **3b** and 2 equivalents of *rac*-**2a** in $CDCl_3$ at ambient temperature. CD and absorption spectra of diastereopure **3b**·(*S*,*S*)-**2a** (0.50 mM) and enantiopure (*R*,*R*)-**2a** (0.50 mM) separately prepared are also shown. (**c**) ^{1}H NMR spectra of (top) a mixture of **3b** (0.50 mM) and *rac*-**2a** (1.0 mM), (middle) **3b**·(*S*,*S*)-**2a** (1st fraction), and (bottom) (*R*,*R*)-**2a** (2nd fraction) in $CDCl_3$ at 25 °C. * denotes signals from the impurities contained in eluents ($CHCl_3$/MeOH) used for column chromatography.

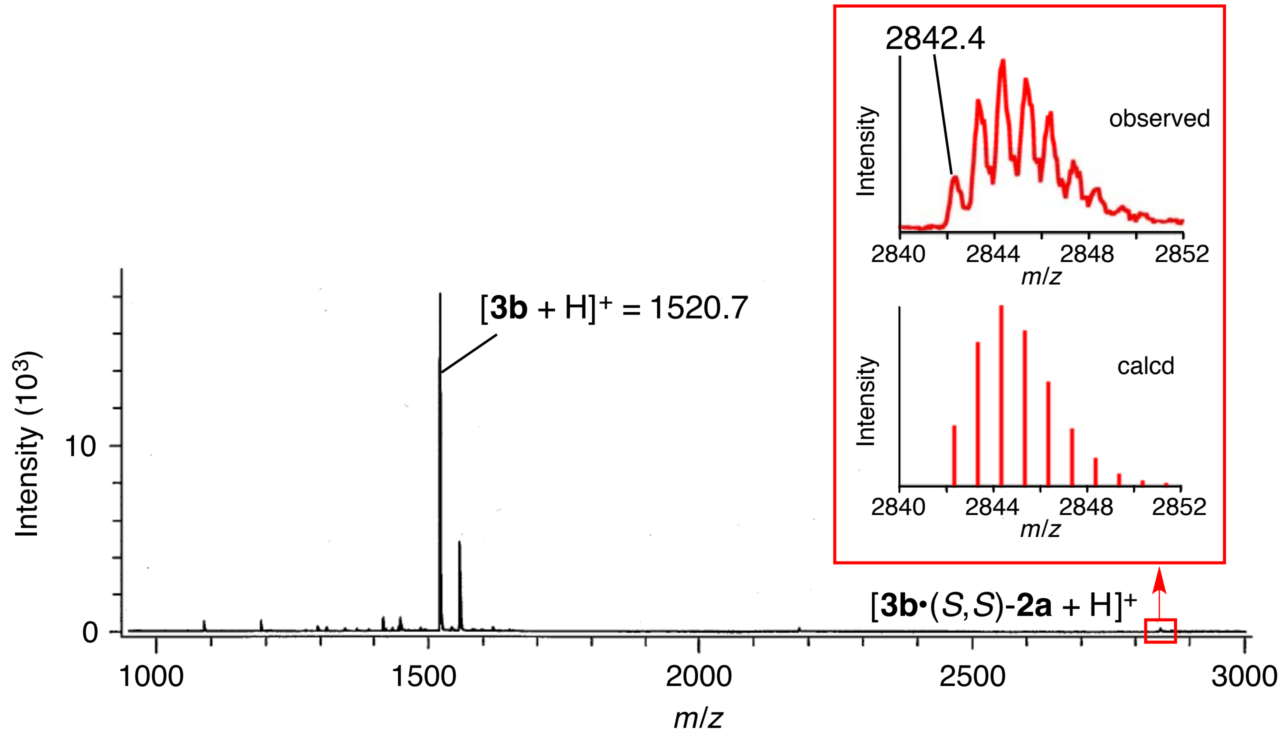

Supplementary Figure 31 | ESI-MS spectrum of duplex (*R*,*R*,*S*,*S*,*R*,*R*)-3b·(*S*,*S*)-2a. Positive mode ESI-MS ($CHCl_3$/MeOH = 1/1 (*v*/*v*) as eluent) spectrum of **3b**·(*S*,*S*)-**2a**.

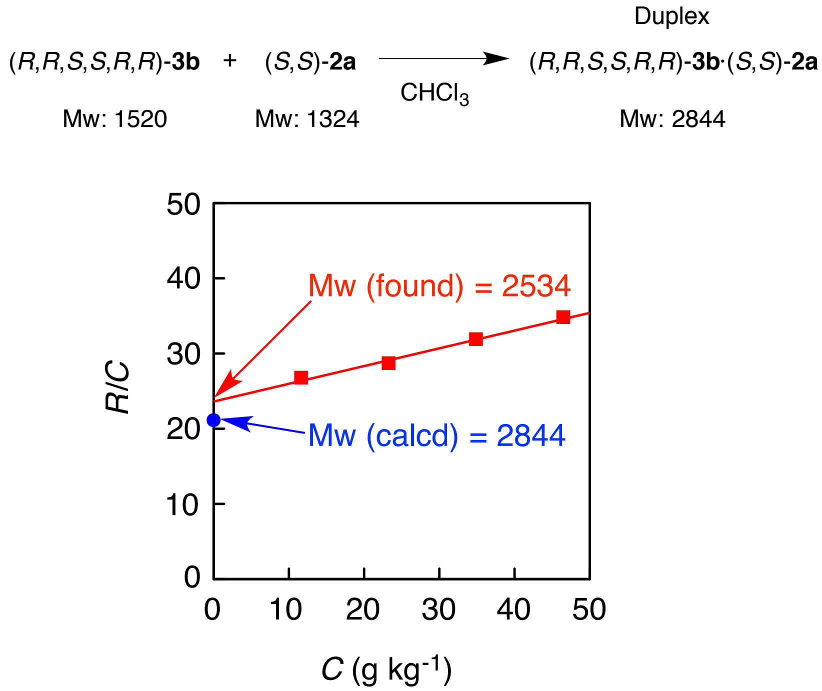

Supplementary Figure 32 | VPO measurement of duplex (*R*,*R*,*S*,*S*,*R*,*R*)-3b·(*S*,*S*)-2a. Plots of *R*/*C* versus concentration of the duplex ((*R*,*R*,*S*,*S*,*R*,*R*)-**3b**·(*S*,*S*)-**2a**) in $CHCl_3$ at 40 °C (*R*: VPO response, arbitrary unit; *C*: initial concentration of the duplex). [Duplex] = 4.05, 8.10, 12.2, and 16.2 mmol kg^{-1}.

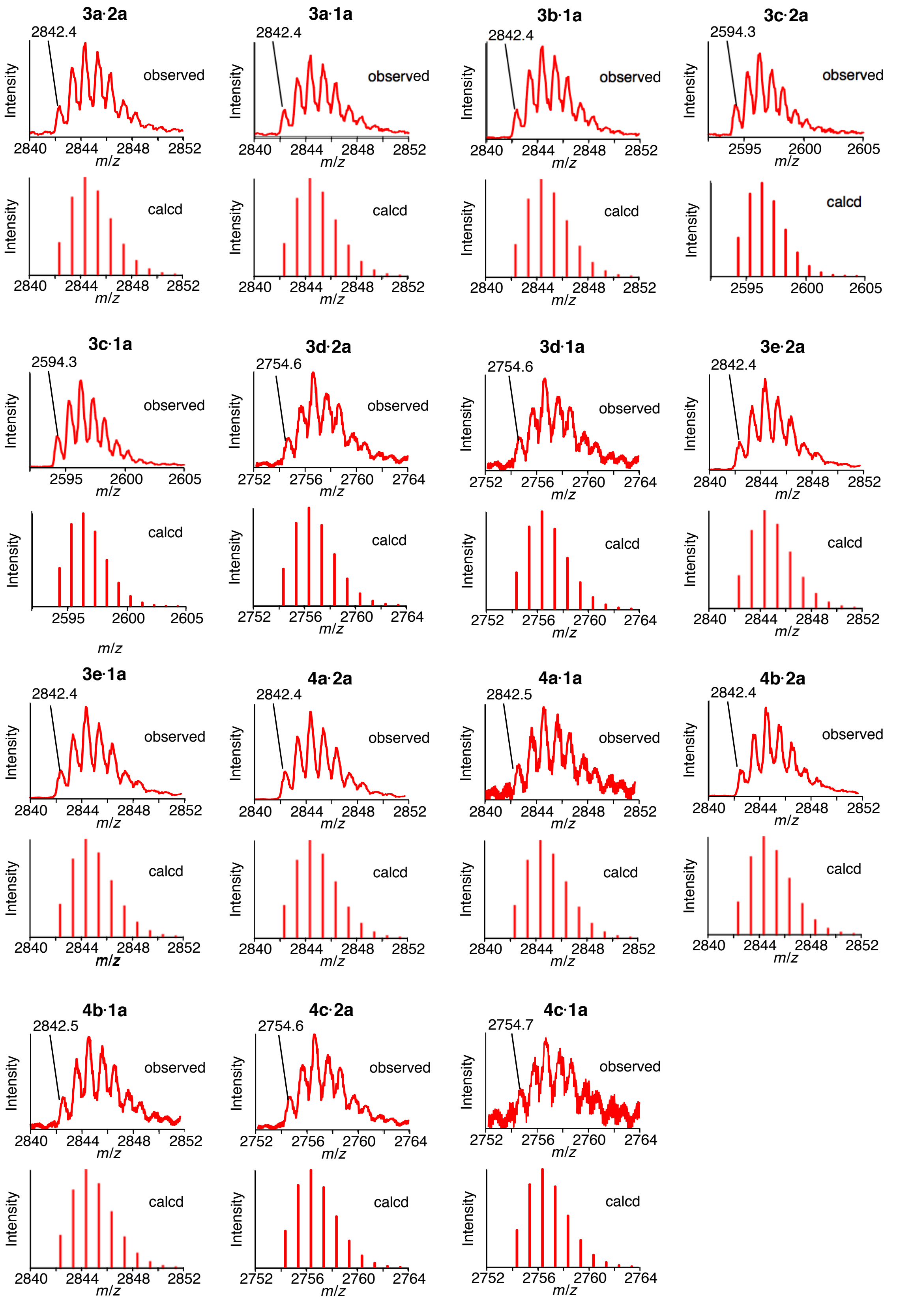

Supplementary Figure 33 | ESI-MS spectra of complementary duplexes. Positive mode ESI-MS ($CHCl_3$/MeOH = 1/1 (*v*/*v*) as eluent) spectra of **3a·2a**, **3a·1a**, **3b·1a**, **3c·2a**, **3c·1a**, **3d·2a**, **3d·1a**, **3e·2a**, **3e·1a**, **4a·2a**, **4a·1a**, **4b·2a**, **4b·1a**, **4c·2a**, and **4c·1a**. The ESI-MS spectrum of **3b**·(*S*,*S*)-**2a** is shown in Supplementary Fig. 31.

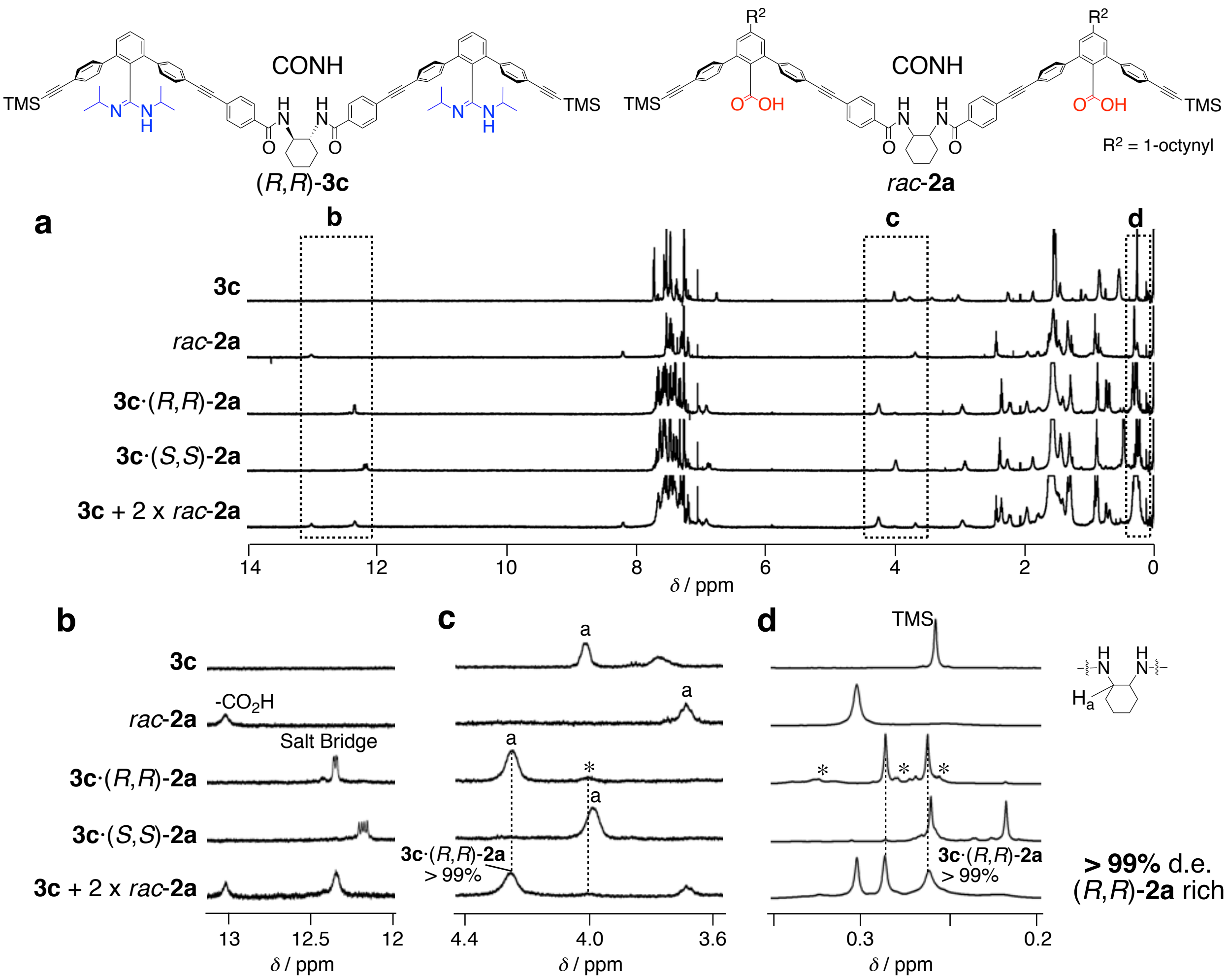

Supplementary Figure 34 | Diastereoselective duplex formation between 3c$_{\mathbf{CONH}}$ and *rac*-2a$_{\mathbf{CONH}}$ (run 3, Table 1). Full (**a**) and partial (**b** and **c**) ^{1}H NMR spectra of **3c** (0.50 mM), *rac*-**2a** (0.50 mM), **3c**·(*R*,*R*)-**2a** (0.50 mM), **3c**·(*S*,*S*)-**2a** (0.50 mM), and a mixture of **3c** (0.50 mM) and *rac*-**2a** (1.0 mM) in $CDCl_3$ at 25 °C. ∗ denotes peaks due to an unknown complex probably formed between **3c** and (*R*,*R*)-**2a** (7.3%).

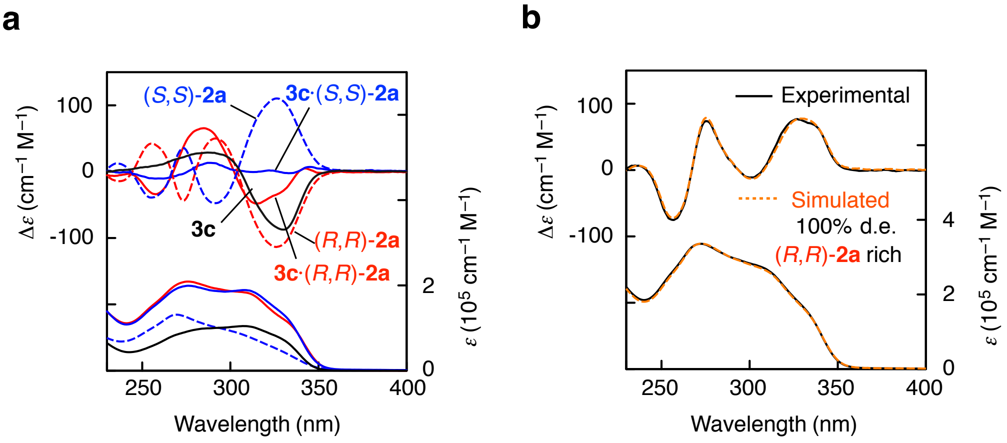

Supplementary Figure 35. | Diastereoselective duplex formation between 3c$_{\mathbf{CONH}}$ and *rac*-2a$_{\mathbf{CONH}}$. (**a**) CD and absorption spectra (0.50 mM) of **3c**, (*R*,*R*)-**2a**, (*S*,*S*)-**2a**, **3c**·(*R*,*R*)-**2a**, and **3c**·(*S*,*S*)-**2a** in $CDCl_3$ at ambient temperature. (**b**) Experimental and simulated CD (d.e. = 100%) and absorption spectra for a mixture of **3c** (0.50 mM) and *rac*-**2a** (1.0 mM) in $CDCl_3$ at ambient temperature.

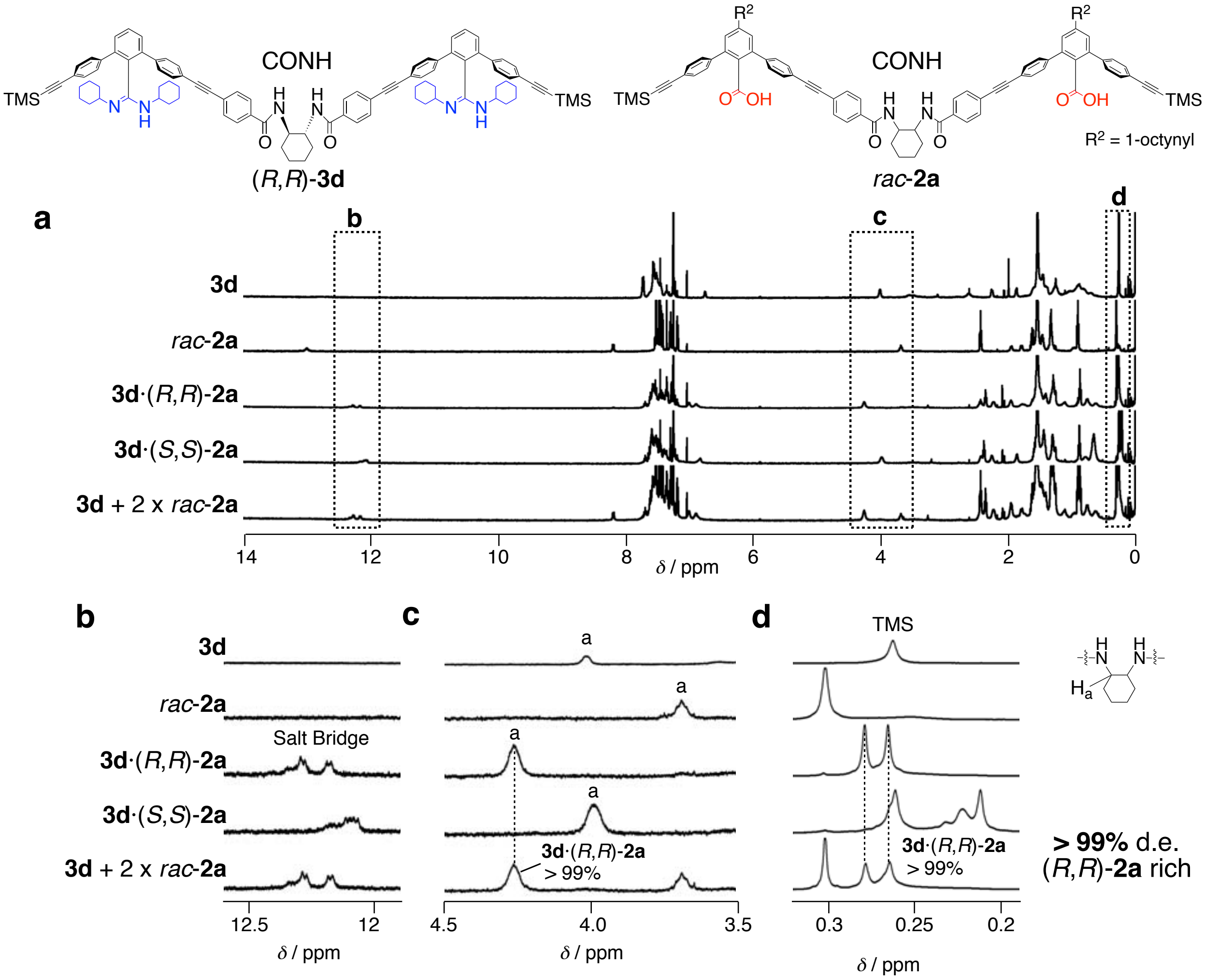

Supplementary Figure 36 | Diastereoselective duplex formation between 3d$_{CONH}$ and *rac*-2a$_{CONH}$ (run 4, Table 1). Full (**a**) and partial (**b** and **c**) ^{1}H NMR spectra of **3d** (0.50 mM), *rac*-**2a** (0.50 mM), **3d**·(*R*,*R*)-**2a** (0.50 mM), **3d**·(*S*,*S*)-**2a** (0.50 mM), and a mixture of **3d** (0.50 mM) and *rac*-**2a** (1.0 mM) in $CDCl_3$ at 25 °C.

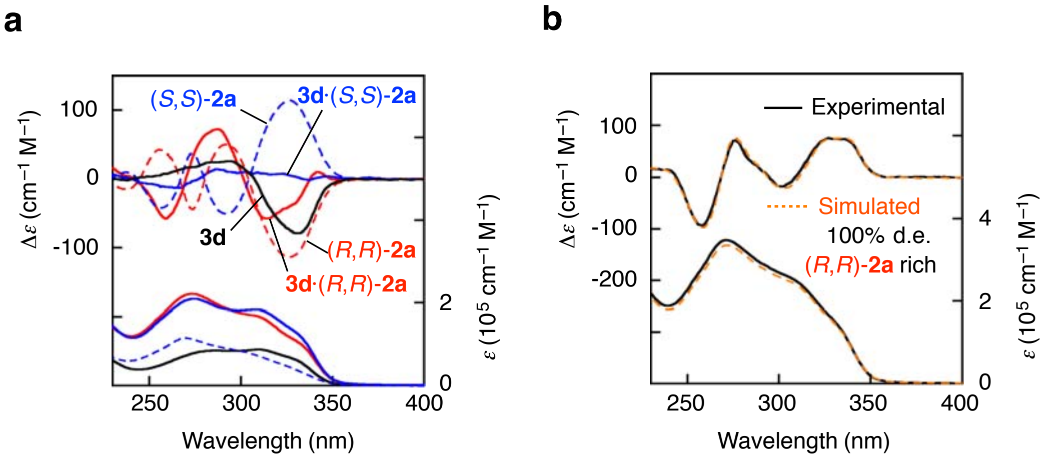

Supplementary Figure 37 | Diastereoselective duplex formation between 3d$_{CONH}$ and *rac*-2a$_{CONH}$. (**a**) CD and absorption spectra (0.50 mM) of **3d**, (*R*,*R*)-**2a**, (*S*,*S*)-**2a**, **3d**·(*R*,*R*)-**2a**, and **3d**·(*S*,*S*)-**2a** in $CDCl_3$ at ambient temperature. (**b**) Experimental and simulated CD (d.e. = 100%) and absorption spectra for a mixture of **3d** (0.50 mM) and *rac*-**2a** (1.0 mM) in $CDCl_3$ at ambient temperature.

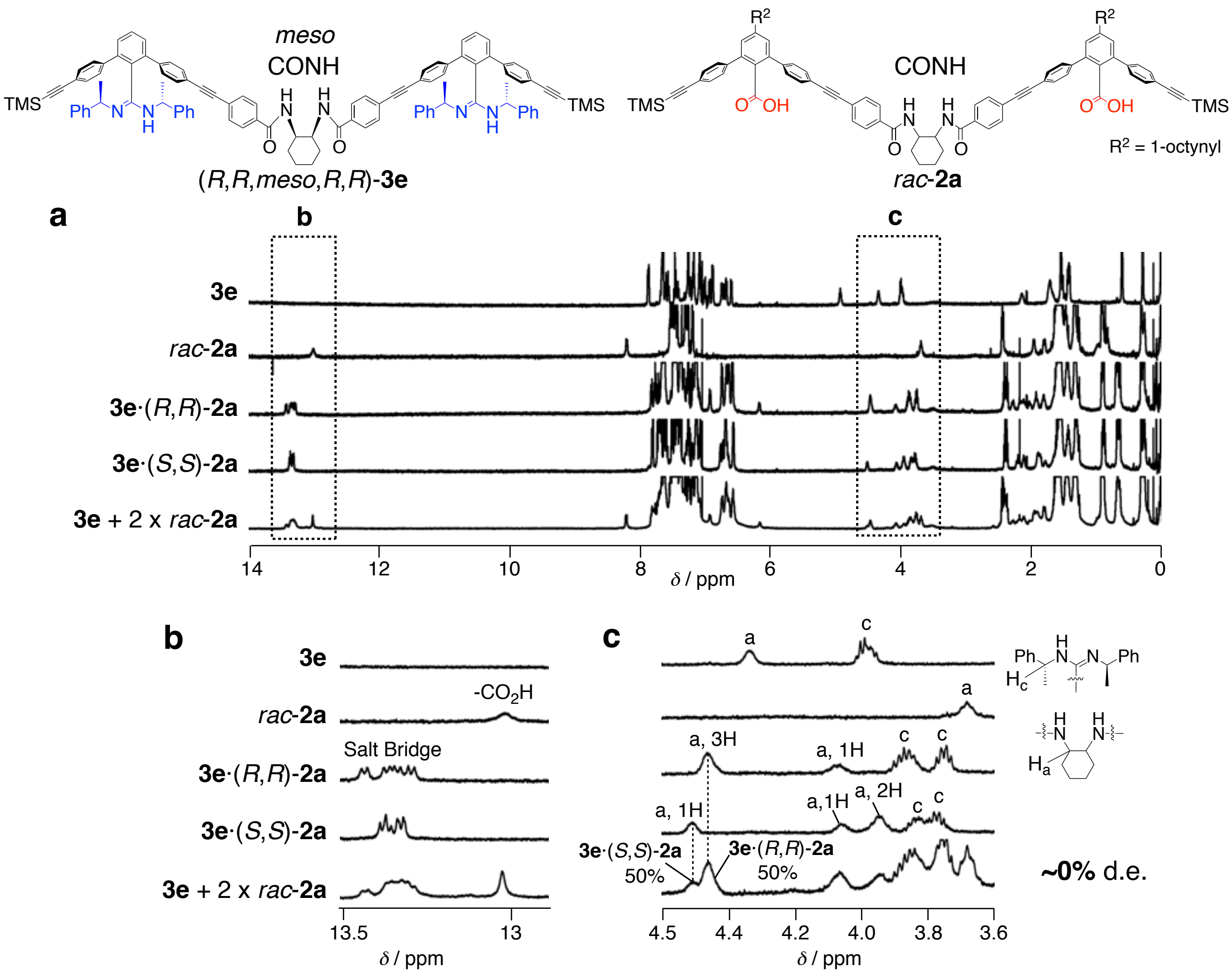

Supplementary Figure 38 | Diastereoselective duplex formation between $\mathbf{3e}_{\mathbf{CONH}}$ and *rac*-$\mathbf{2a}_{\mathbf{CONH}}$ (run 5, Table 1). Full (**a**) and partial (**b** and **c**) ^{1}H NMR spectra of **3e** (0.50 mM), *rac*-**2a** (0.50 mM), **3e**·(*R*,*R*)-**2a** (0.50 mM), **3e**·(*S*,*S*)-**2a** (0.50 mM), and a mixture of **3e** (0.50 mM) and *rac*-**2a** (1.0 mM) in $CDCl_3$ at 25 °C.

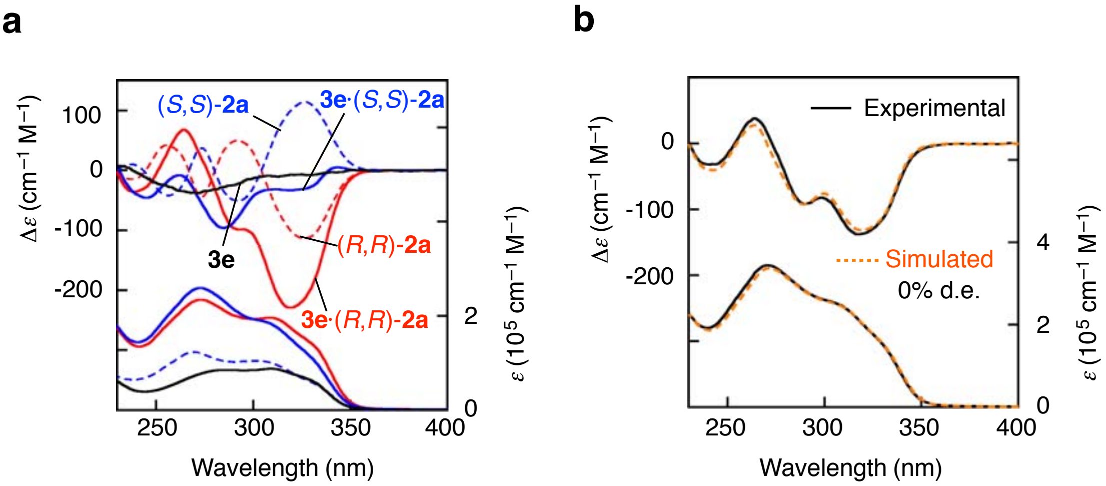

Supplementary Figure 39 | Diastereoselective duplex formation between $\mathbf{3e}_{\mathbf{CONH}}$ and *rac*-$\mathbf{2a}_{\mathbf{CONH}}$. (**a**) CD and absorption spectra (0.50 mM) of **3e**, (*R*,*R*)-**2a**, (*S*,*S*)-**2a**, **3e**·(*R*,*R*)-**2a**, and **3e**·(*S*,*S*)-**2a** in $CDCl_3$ at ambient temperature. (**b**) Experimental and simulated CD (d.e. = 0%) and absorption spectra for a mixture of **3e** (0.50 mM) and *rac*-**2a** (1.0 mM) in $CDCl_3$ at ambient temperature.

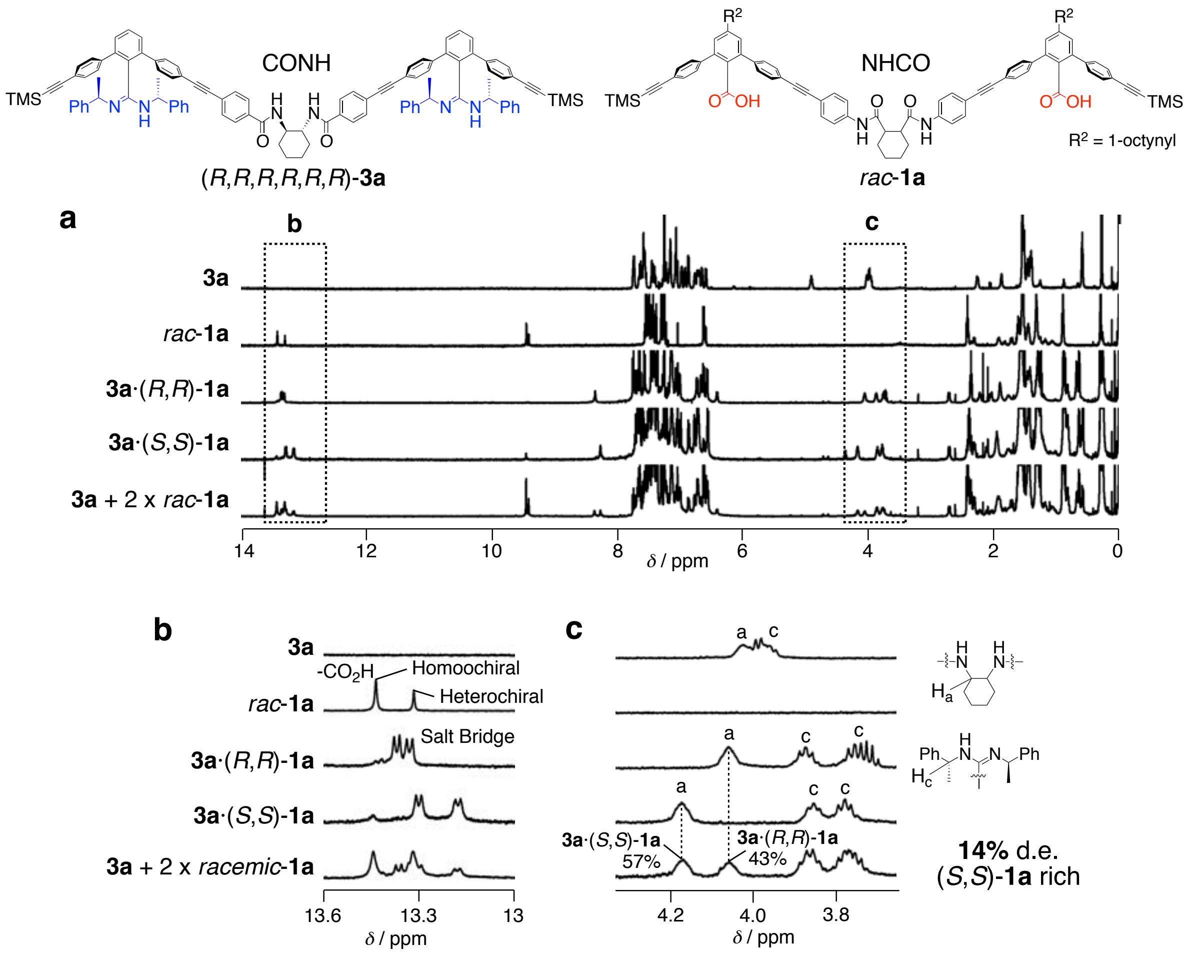

Supplementary Figure 40 | Diastereoselective duplex formation between 3a$_{CONH}$ and *rac*-1a$_{NHCO}$ (run 6, Table 1). Full (**a**) and partial (**b** and **c**) ^{1}H NMR spectra of **3a** (0.50 mM), *rac*-**1a** (0.50 mM), **3a**·(*R*,*R*)-**1a** (0.50 mM), **3a**·(*S*,*S*)-**1a** (0.50 mM), and a mixture of **3a** (0.50 mM) and *rac*-**1a** (1.0 mM) in CDCl$_3$ at 25 °C.

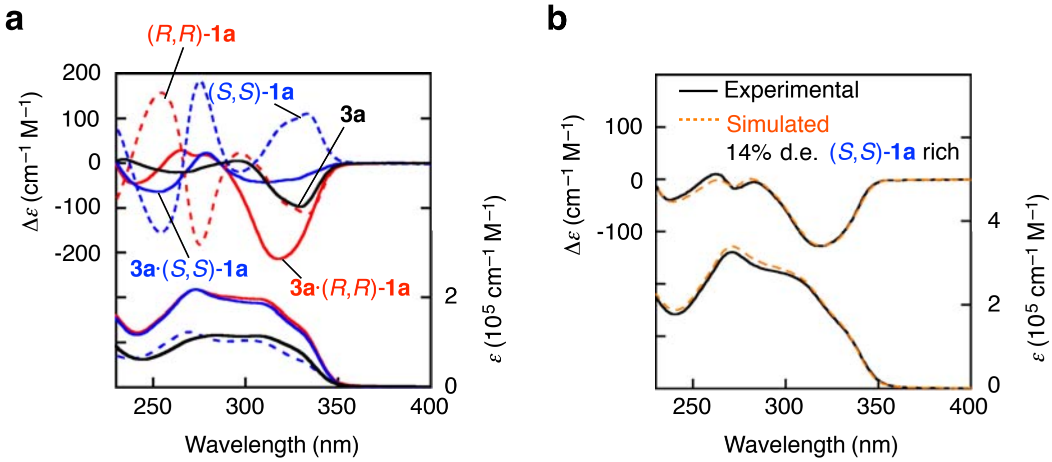

Supplementary Figure 41 | Diastereoselective Duplex Formation between 3a$_{CONH}$ and *rac*-1a$_{NHCO}$. (**a**) CD and absorption spectra (0.50 mM) of **3a**, (*R*,*R*)-**1a**, (*S*,*S*)-**1a**, **3a**·(*R*,*R*)-**1a**, and **3a**·(*S*,*S*)-**1a** in CDCl$_3$ at ambient temperature. (**b**) Experimental and simulated CD (d.e. = 14%) and absorption spectra for a mixture of **3a** (0.50 mM) and *rac*-**1a** (1.0 mM) in CDCl$_3$ at ambient temperature.

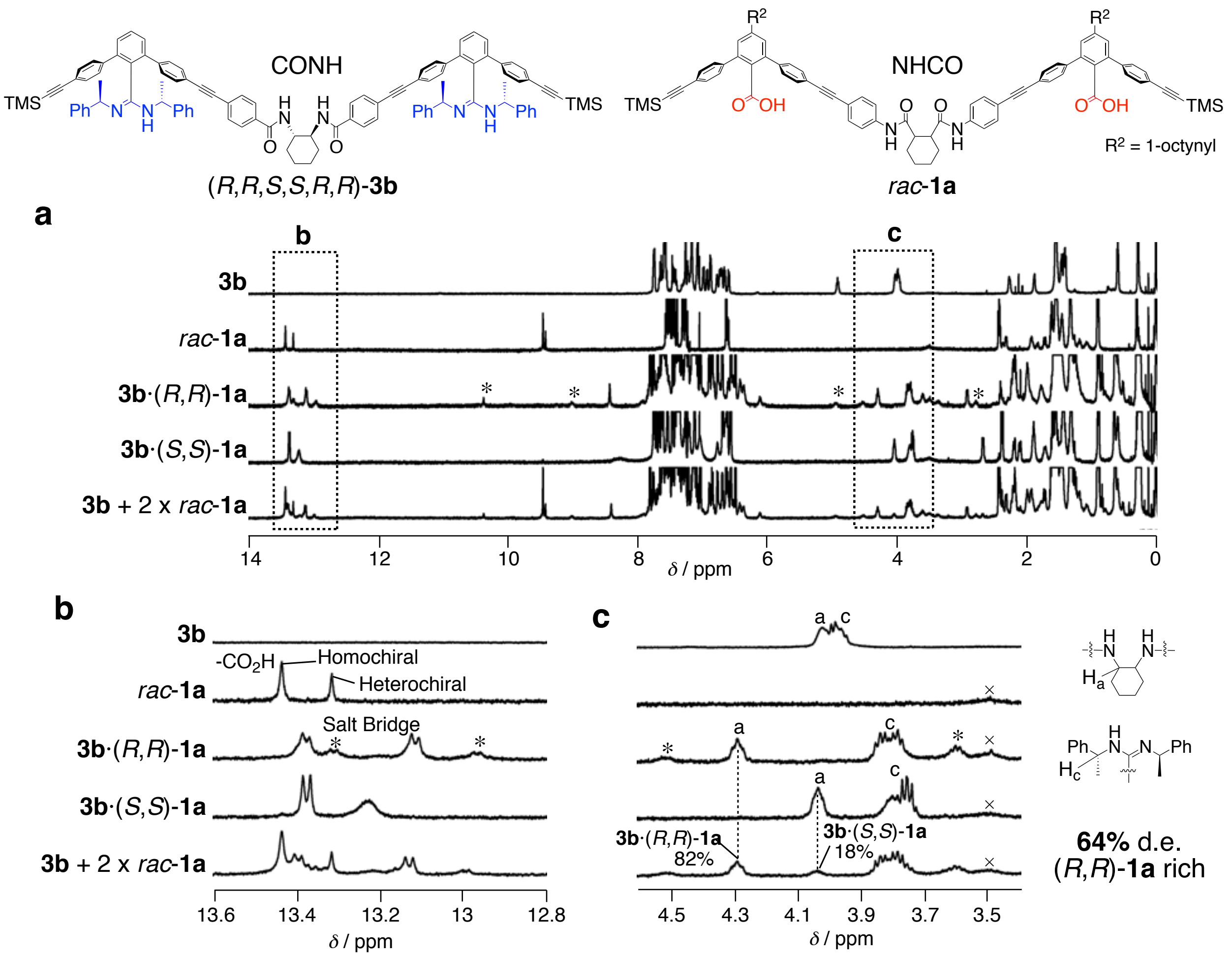

Supplementary Figure 42 | Diastereoselective duplex formation between $\mathbf{3b_{CONH}}$ and *rac*-$\mathbf{1a_{NHCO}}$ (run 7, Table 1). Full (**a**) and partial (**b** and **c**) ^{1}H NMR spectra of **3b** (0.50 mM), *rac*-**1a** (0.50 mM), **3b**·(*R*,*R*)-**1a** (0.50 mM), **3b**·(*S*,*S*)-**1a** (0.50 mM), and a mixture of **3b** (0.50 mM) and *rac*-**1a** (1.0 mM) in $CDCl_3$ at 25 °C. ∗ denotes peaks due to an unknown complex probably formed between **3b** and (*R*,*R*)-**1a** (14%). × denotes signals from the impurities contained in $CDCl_3$.

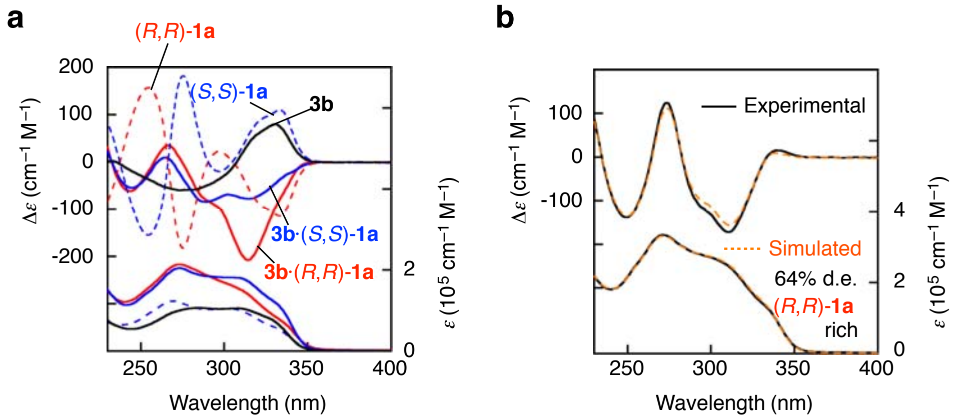

Supplementary Figure 43 | Diastereoselective duplex formation between $\mathbf{3b_{CONH}}$ and *rac*-$\mathbf{1a_{NHCO}}$. (**a**) CD and absorption spectra (0.50 mM) of **3b**, (*R*,*R*)-**1a**, (*S*,*S*)-**1a**, **3b**·(*R*,*R*)-**1a**, and **3b**·(*S*,*S*)-**1a** in $CDCl_3$ at ambient temperature. (**b**) Experimental and simulated CD (d.e. = 64%) and absorption spectra for a mixture of **3b** (0.50 mM) and *rac*-**1a** (1.0 mM) in $CDCl_3$ at ambient temperature.

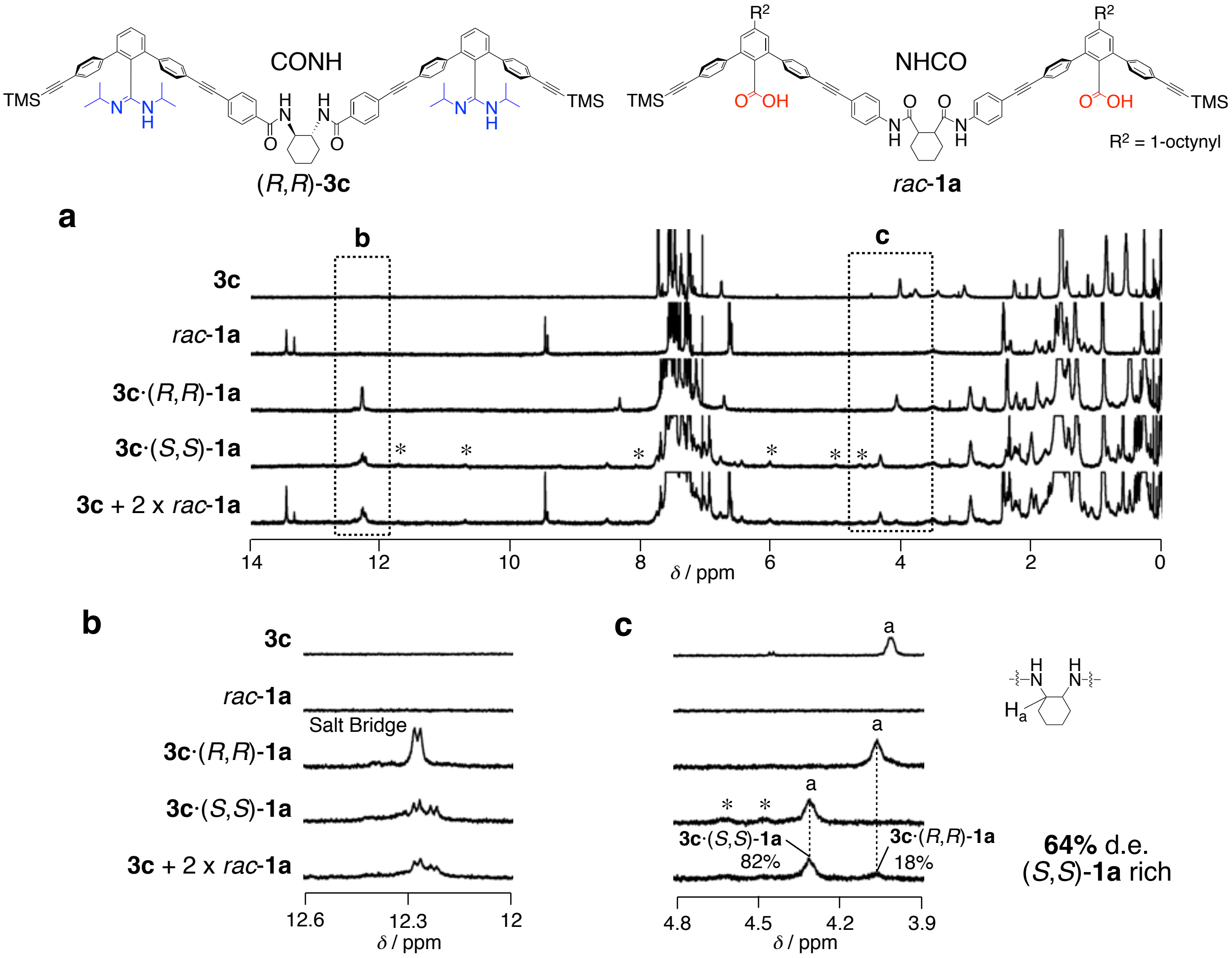

Supplementary Figure 44 | Diastereoselective duplex formation between 3c$_{\text{CONH}}$ and *rac*-1a$_{\text{NHCO}}$ (run 8, Table 1). Full (**a**) and partial (**b** and **c**) ^{1}H NMR spectra of **3c** (0.50 mM), *rac*-**1a** (0.50 mM), **3c**·(*R*,*R*)-**1a** (0.50 mM), **3c**·(*S*,*S*)-**1a** (0.50 mM), and a mixture of **3c** (0.50 mM) and *rac*-**1a** (1.0 mM) in $CDCl_3$ at 25 °C. ∗ denotes peaks due to an unknown complex probably formed between **3c** and (*S*,*S*)-**1a** (16%).

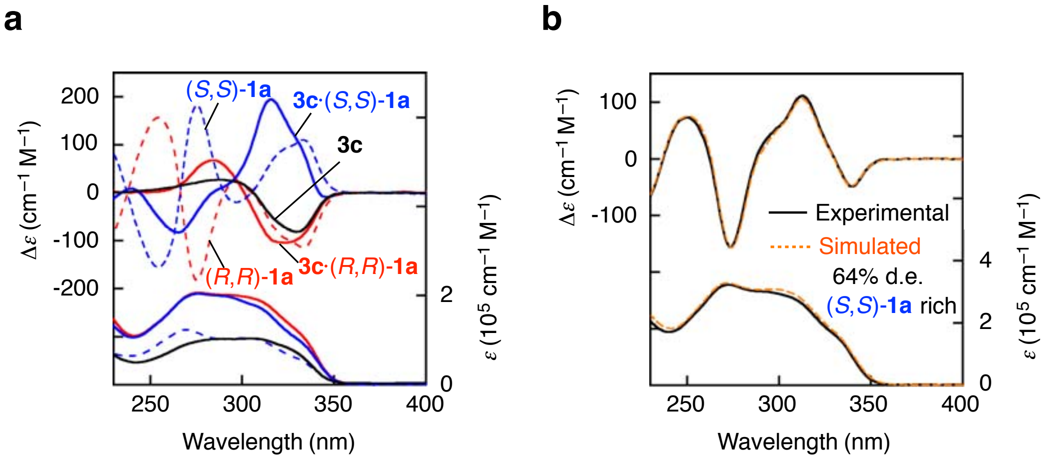

Supplementary Figure 45 | Diastereoselective duplex formation between 3c$_{\text{CONH}}$ and *rac*-1a$_{\text{NHCO}}$. (**a**) CD and absorption spectra (0.50 mM) of **3c**, (*R*,*R*)-**1a**, (*S*,*S*)-**1a**, **3c**·(*R*,*R*)-**1a**, and **3c**·(*S*,*S*)-**1a** in $CDCl_3$ at ambient temperature. (**b**) Experimental and simulated CD (d.e. = 64%) and absorption spectra for a mixture of **3c** (0.50 mM) and *rac*-**1a** (1.0 mM) in $CDCl_3$ at ambient temperature.

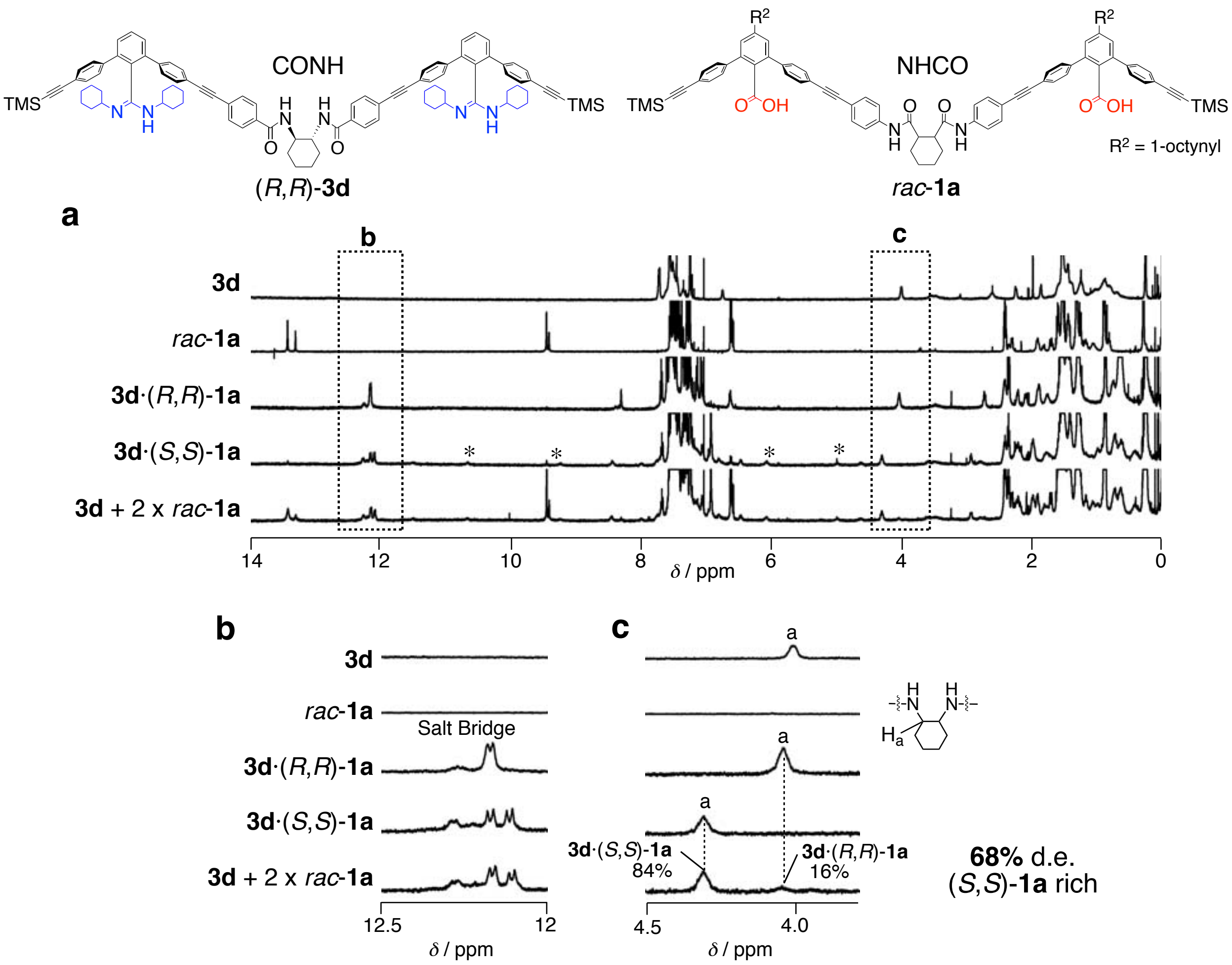

Supplementary Figure 46 | Diastereoselective duplex formation between 3d$_{\mathbf{CONH}}$ and *rac*-1a$_{\mathbf{NHCO}}$ (run 9, Table 1). Full (**a**) and partial (**b** and **c**) ^{1}H NMR spectra of **3d** (0.50 mM), *rac*-**1a** (0.50 mM), **3d**·(*R*,*R*)-**1a** (0.50 mM), **3d**·(*S*,*S*)-**1a** (0.50 mM), and a mixture of **3d** (0.50 mM) and *rac*-**1a** (1.0 mM) in $CDCl_3$ at 25 °C. ∗ denotes peaks due to an unknown complex probably formed between **3b** and (*S*,*S*)-**1a** (13%).

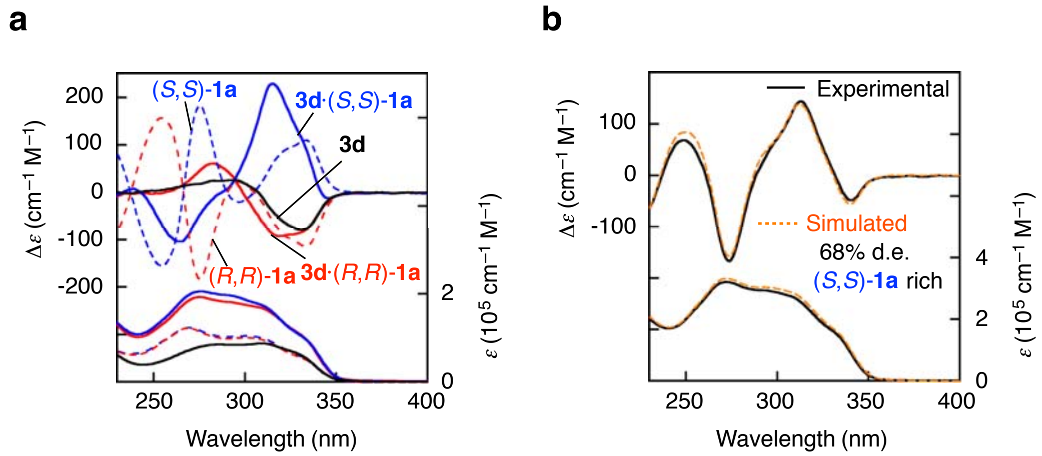

Supplementary Figure 47 | Diastereoselective duplex formation between 3d$_{\mathbf{CONH}}$ and *rac*-1a$_{\mathbf{NHCO}}$. (**a**) CD and absorption spectra (0.50 mM) of **3d**, (*R*,*R*)-**1a**, (*S*,*S*)-**1a**, **3d**·(*R*,*R*)-**1a**, and **3d**·(*S*,*S*)-**1a** in $CDCl_3$ at ambient temperature. (**b**) Experimental and simulated CD (d.e. = 68%) and absorption spectra for a mixture of **3d** (0.50 mM) and *rac*-**1a** (1.0 mM) in $CDCl_3$ at ambient temperature.

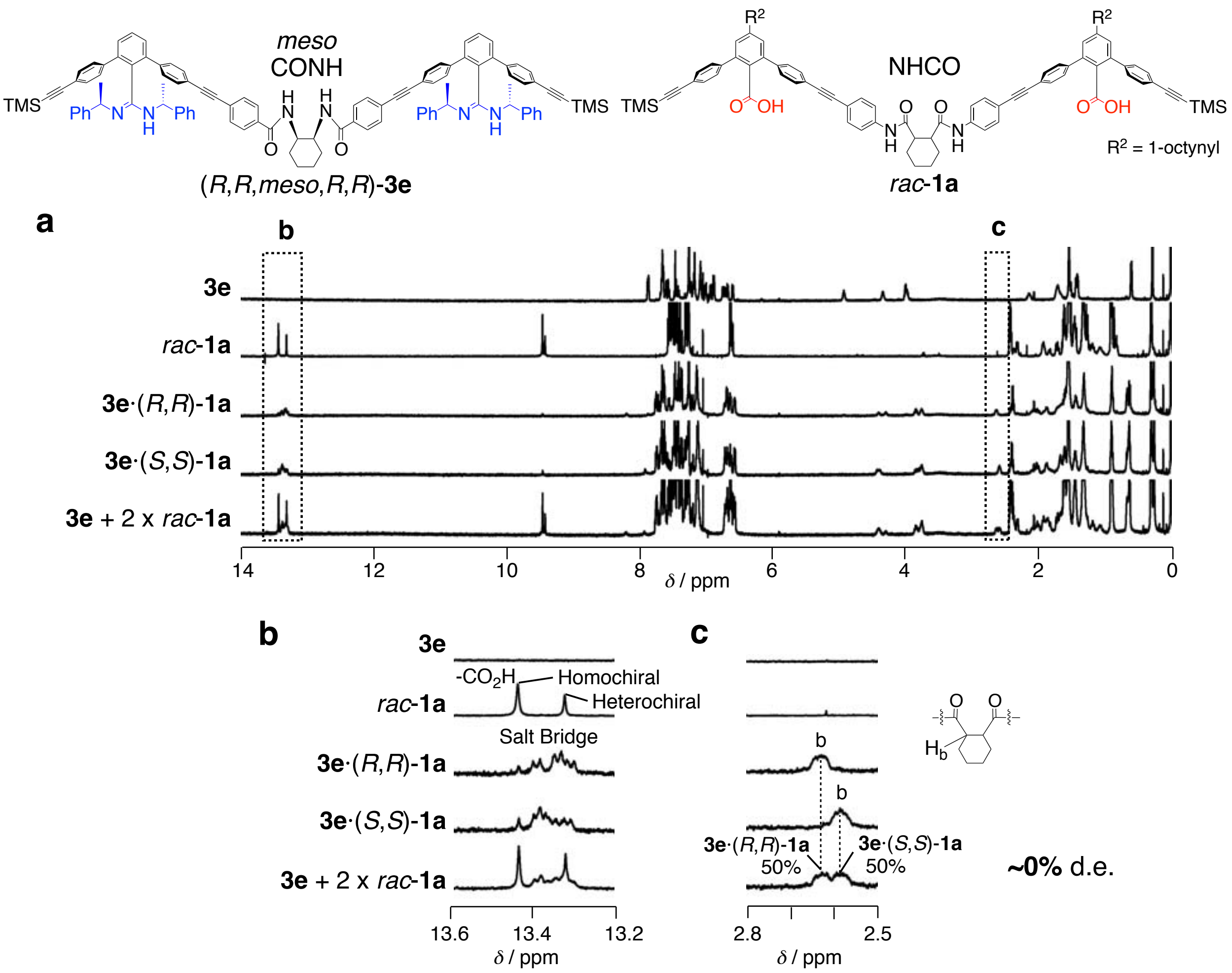

Supplementary Figure 48 | Diastereoselective duplex formation between $\mathbf{3e}_{\mathrm{CONH}}$ and *rac*-$\mathbf{1a}_{\mathrm{NHCO}}$ (run 10, Table 1). Full (**a**) and partial (**b** and **c**) ^{1}H NMR spectra of **3e** (0.50 mM), *rac*-**1a** (0.50 mM), **3e**·(*R*,*R*)-**1a** (0.50 mM), **3e**·(*S*,*S*)-**1a** (0.50 mM), and a mixture of **3e** (0.50 mM) and *rac*-**1a** (1.0 mM) in $CDCl_3$ at 25 °C.

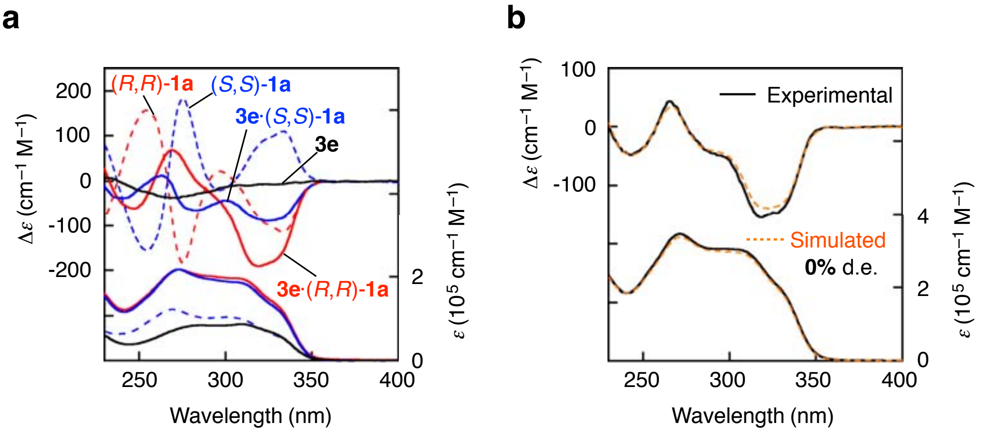

Supplementary Figure 49 | Diastereoselective duplex formation between $\mathbf{3e}_{\mathrm{CONH}}$ and *rac*-$\mathbf{1a}_{\mathrm{NHCO}}$. (**a**) CD and absorption spectra (0.50 mM) of **3e**, (*R*,*R*)-**1a**, (*S*,*S*)-**1a**, **3e**·(*R*,*R*)-**1a**, and **3e**·(*S*,*S*)-**1a** in $CDCl_3$ at ambient temperature. (**b**) Experimental and simulated CD (d.e. = 0%) and absorption spectra for a mixture of **3e** (0.50 mM) and *rac*-**1a** (1.0 mM) in $CDCl_3$ at ambient temperature.

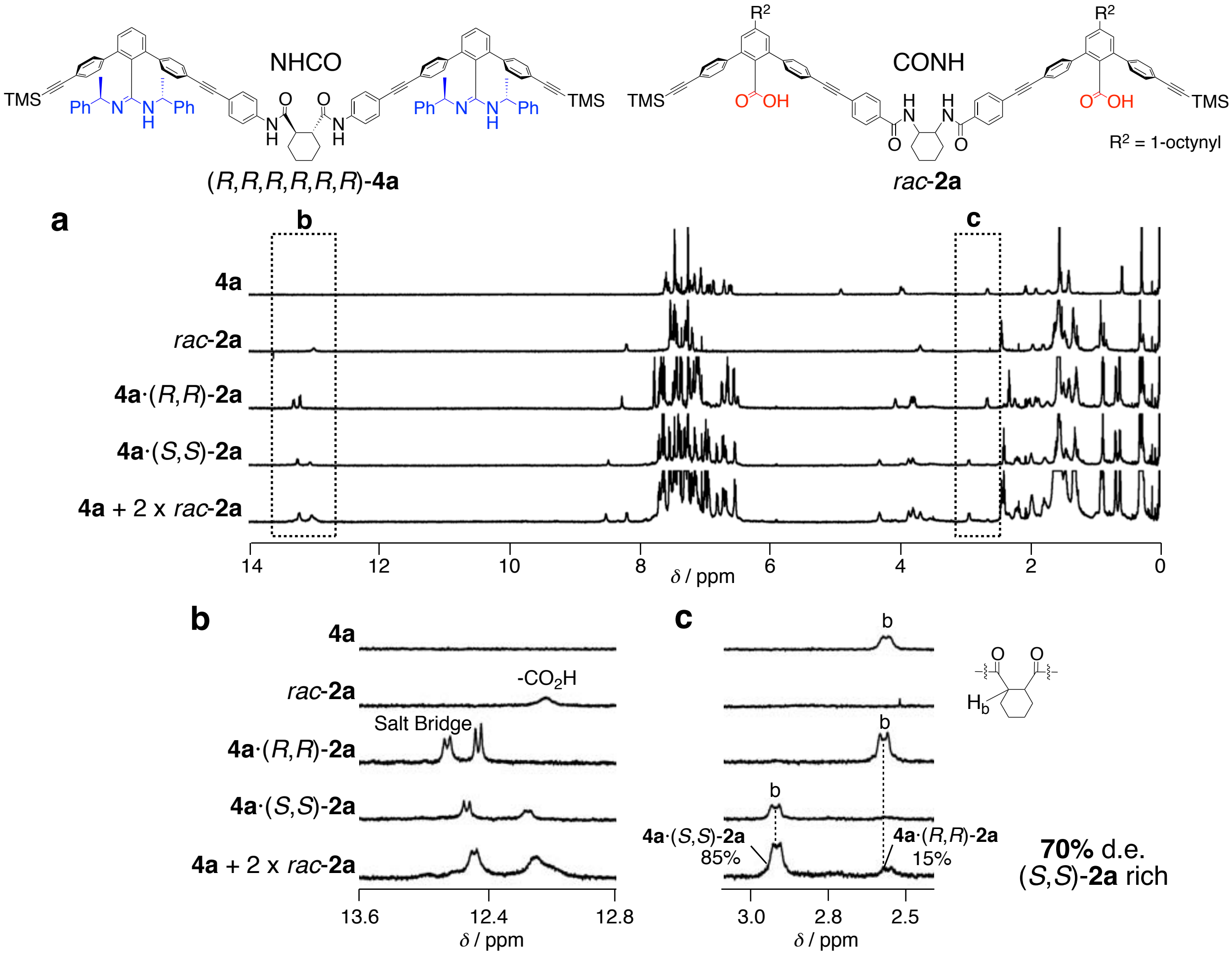

Supplementary Figure 50 | Diastereoselective duplex formation between $4a_{NHCO}$ and *rac*-$2a_{CONH}$ (run 11, Table 1). Full (**a**) and partial (**b** and **c**) ^{1}H NMR spectra of **4a** (0.50 mM), *rac*-**2a** (0.50 mM), **4a**·(*R*,*R*)-**2a** (0.50 mM), **4a**·(*S*,*S*)-**2a** (0.50 mM), and a mixture of **4a** (0.50 mM) and *rac*-**2a** (1.0 mM) in $CDCl_3$ at 25 °C.

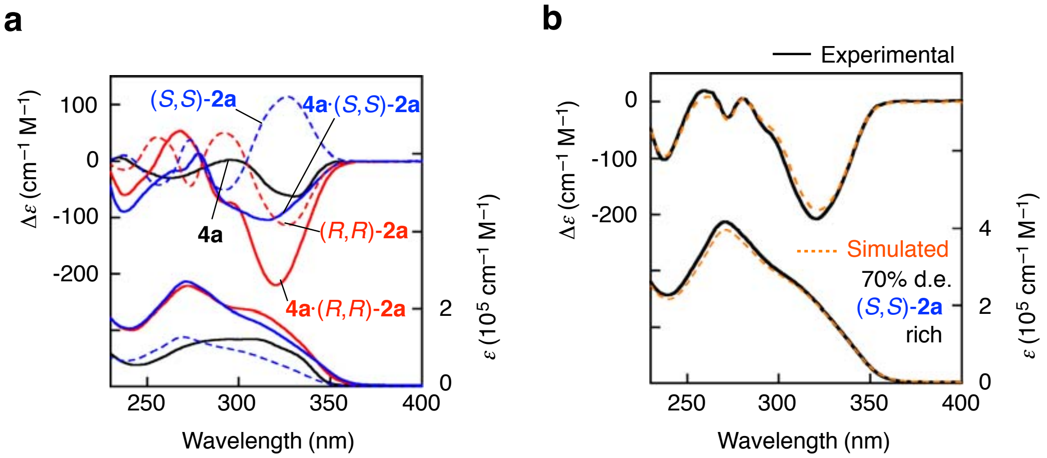

Supplementary Figure 51 | Diastereoselective duplex formation between $4a_{NHCO}$ and *rac*-$2a_{CONH}$. (**a**) CD and absorption spectra (0.50 mM) of **4a**, (*R*,*R*)-**2a**, (*S*,*S*)-**2a**, **4a**·(*R*,*R*)-**2a**, and **4a**·(*S*,*S*)-**2a** in $CDCl_3$ at ambient temperature. (**b**) Experimental and simulated CD (d.e. = 70%) and absorption spectra for a mixture of **4a** (0.50 mM) and *rac*-**2a** (1.0 mM) in $CDCl_3$ at ambient temperature.

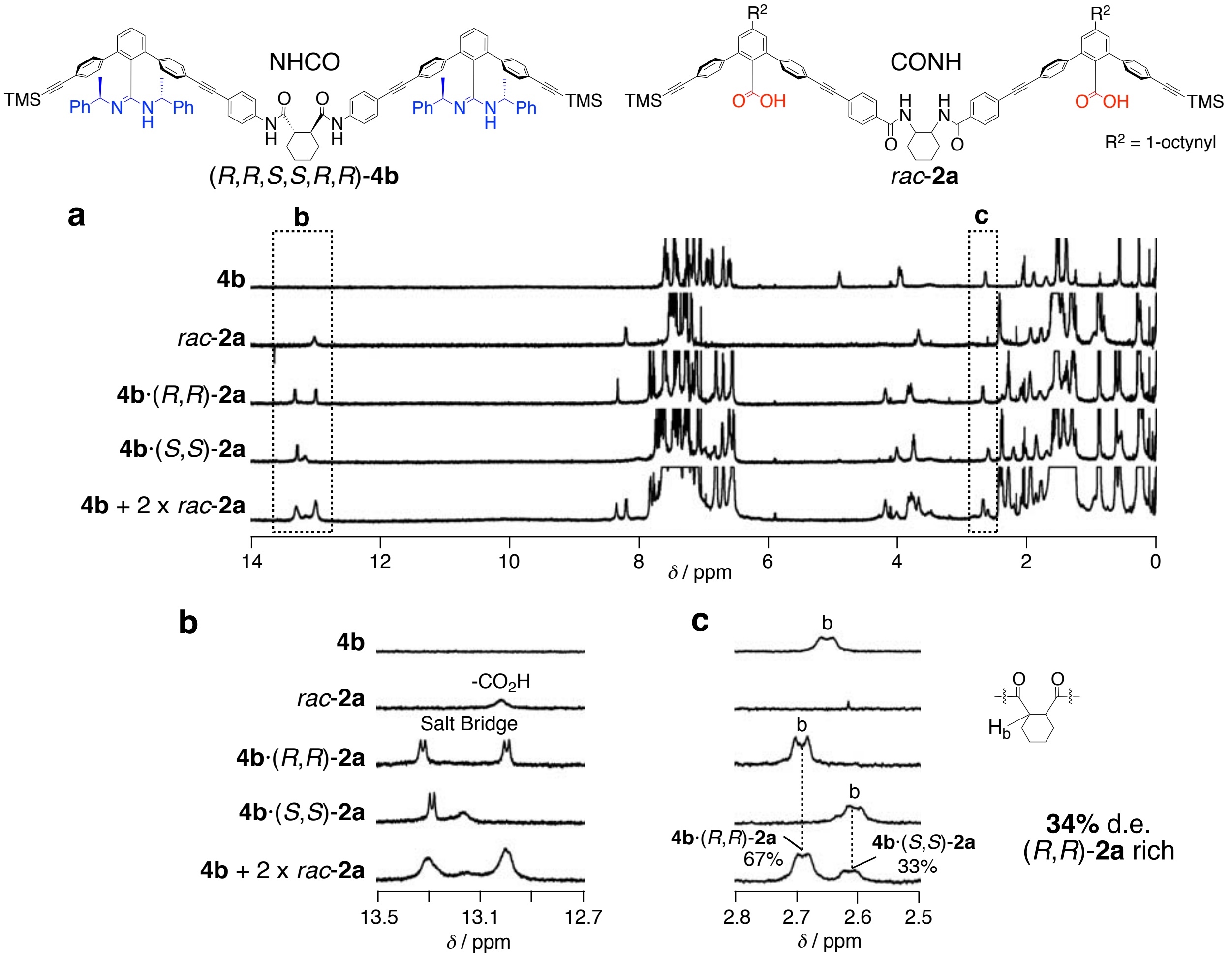

Supplementary Figure 52 | Diastereoselective duplex formation between $\mathbf{4b}_{\mathrm{NHCO}}$ and *rac*-$\mathbf{2a}_{\mathrm{CONH}}$ (run 12, Table 1). Full (**a**) and partial (**b** and **c**) ^{1}H NMR spectra of **4b** (0.50 mM), *rac*-**2a** (0.50 mM), **4b**·(*R*,*R*)-**2a** (0.50 mM), **4b**·(*S*,*S*)-**2a** (0.50 mM), and a mixture of **4b** (0.50 mM) and *rac*-**2a** (1.0 mM) in $CDCl_3$ at 25 °C.

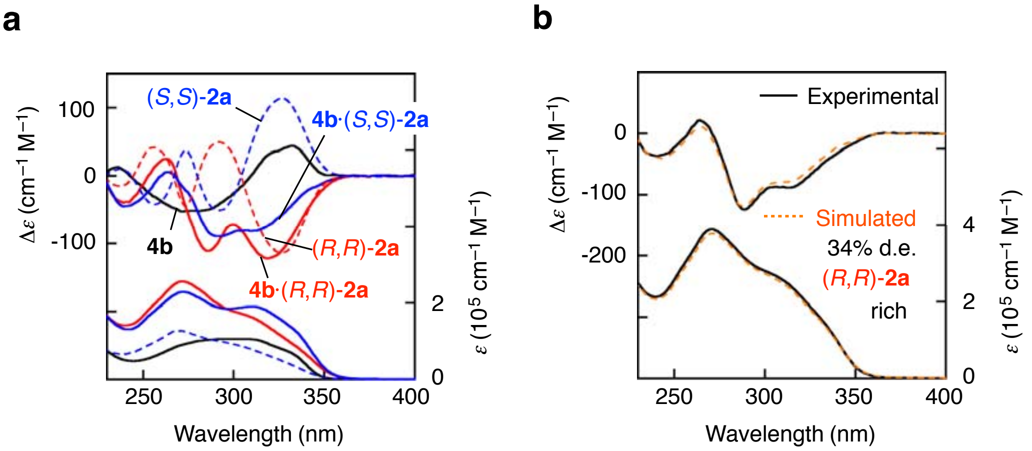

Supplementary Figure 53 | Diastereoselective duplex formation between $\mathbf{4b}_{\mathrm{NHCO}}$ and *rac*-$\mathbf{2a}_{\mathrm{CONH}}$. (**a**) CD and absorption spectra (0.50 mM) of **4b**, (*R*,*R*)-**2a**, (*S*,*S*)-**2a**, **4b**·(*R*,*R*)-**2a**, and **4b**·(*S*,*S*)-**2a** in $CDCl_3$ at ambient temperature. (**b**) Experimental and simulated CD (d.e. = 34%) and absorption spectra for a mixture of **4b** (0.50 mM) and *rac*-**2a** (1.0 mM) in $CDCl_3$ at ambient temperature.

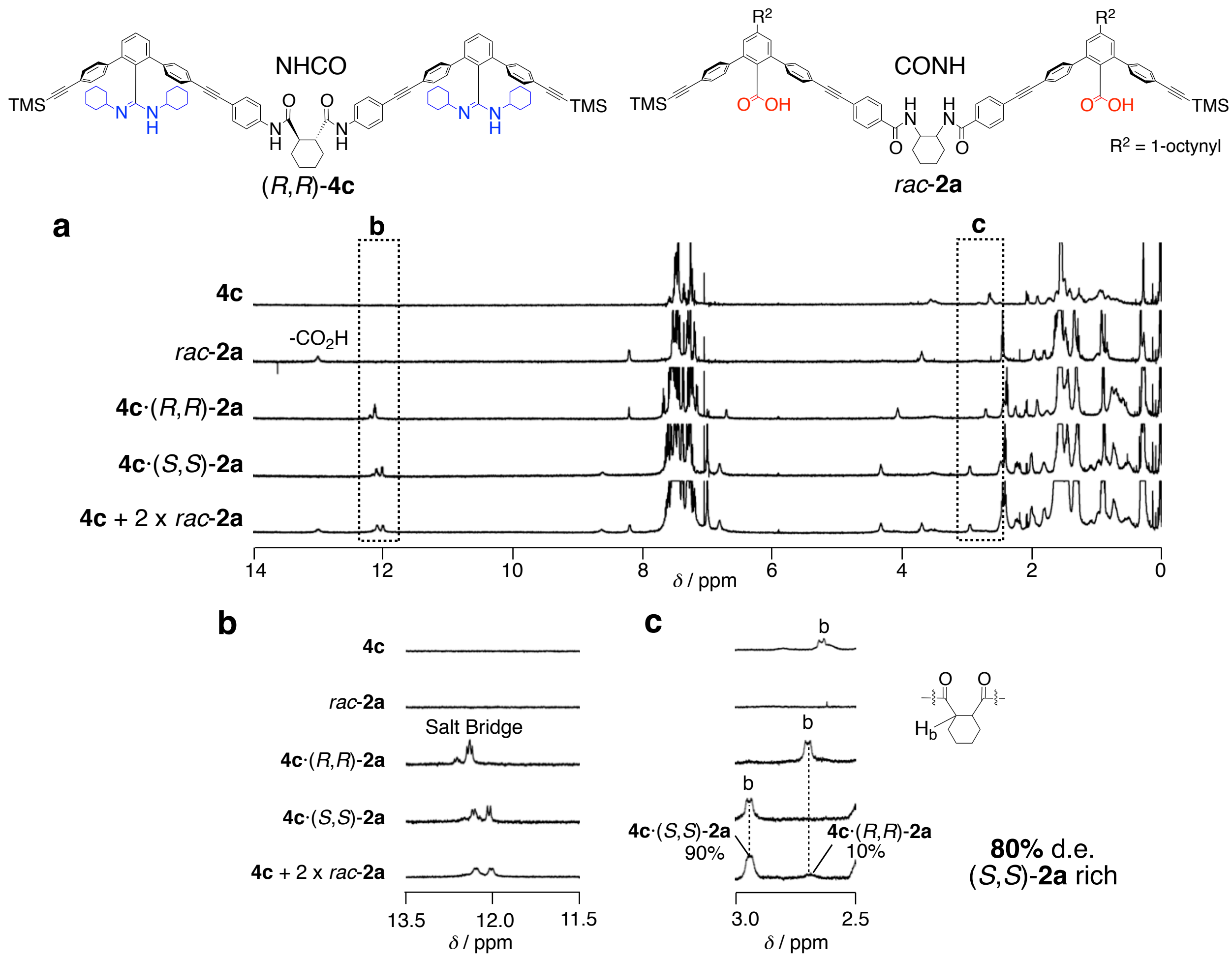

Supplementary Figure 54 | Diastereoselective duplex formation between 4c$_{NHCO}$ and *rac*-2a$_{CONH}$ (run 13, Table 1). Full (**a**) and partial (**b** and **c**) ^{1}H NMR spectra of **4c** (0.50 mM), *rac*-**2a** (0.50 mM), **4c**·(*R*,*R*)-**2a** (0.50 mM), **4c**·(*S*,*S*)-**2a** (0.50 mM), and a mixture of **4c** (0.50 mM) and *rac*-**2a** (1.0 mM) in $CDCl_3$ at 25 °C.

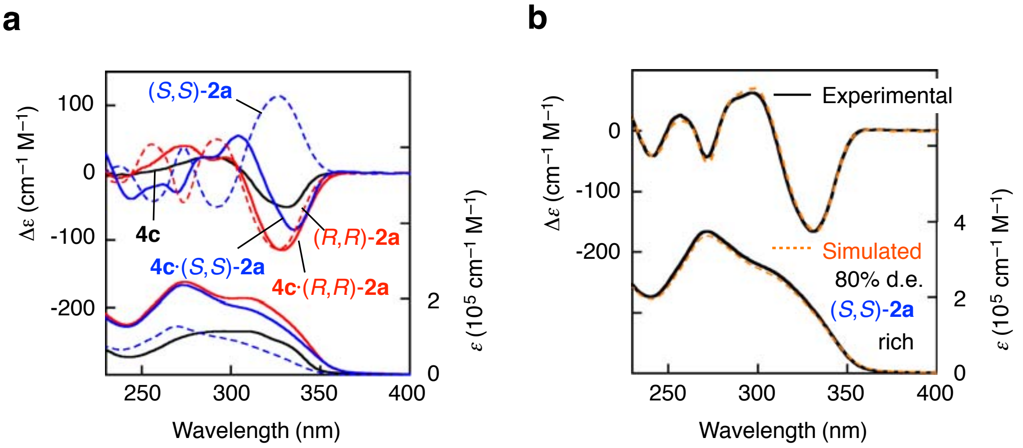

Supplementary Figure 55 | Diastereoselective duplex formation between 4c$_{NHCO}$ and *rac*-2a$_{CONH}$. (**a**) CD and absorption spectra (0.50 mM) of **4c**, (*R*,*R*)-**2a**, (*S*,*S*)-**2a**, **4c**·(*R*,*R*)-**2a**, and **4c**·(*S*,*S*)-**2a** in $CDCl_3$ at ambient temperature. (**b**) Experimental and simulated CD (d.e. = 80%) and absorption spectra for a mixture of **4c** (0.50 mM) and *rac*-**2a** (1.0 mM) in $CDCl_3$ at ambient temperature.

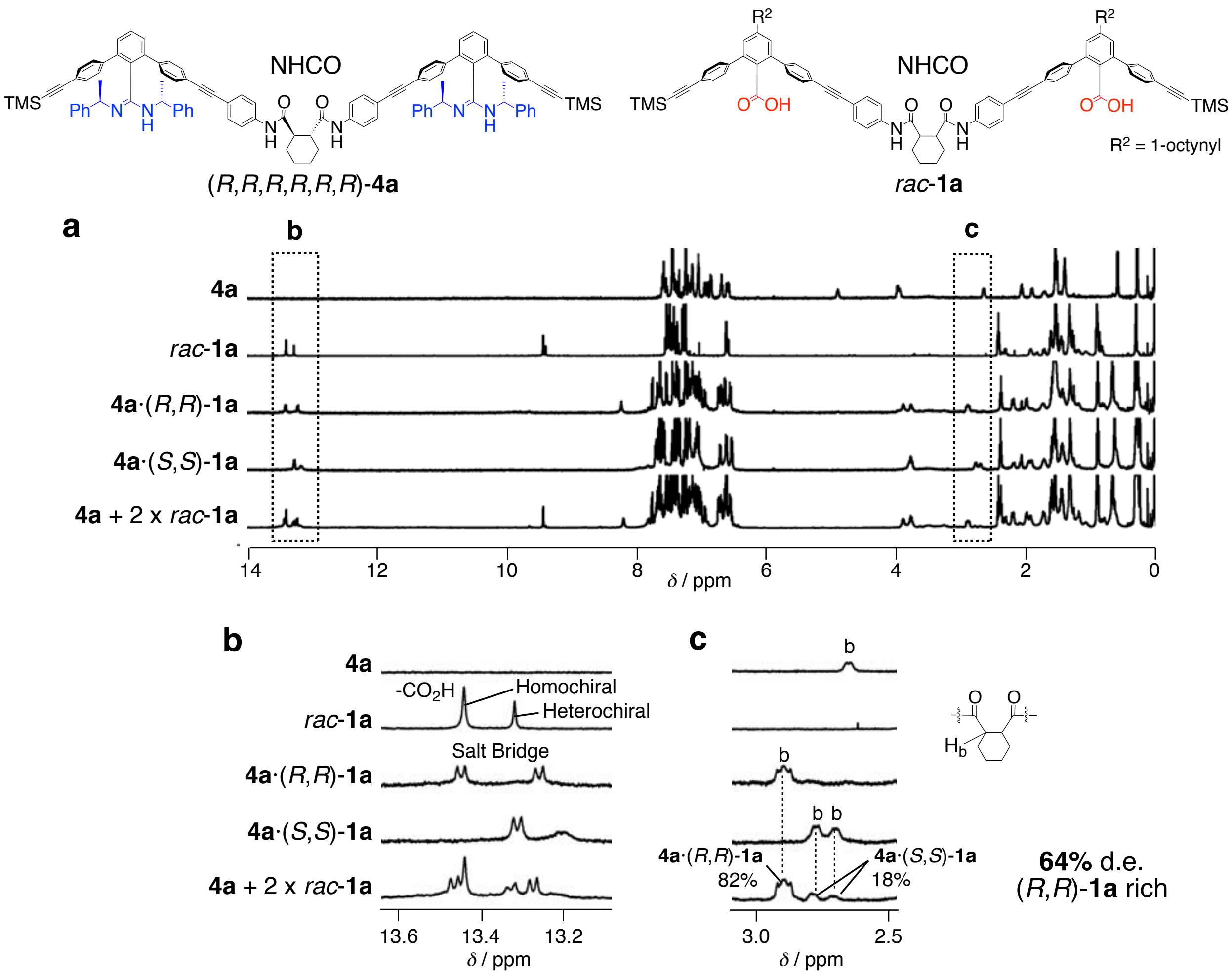

Supplementary Figure 56 | Diastereoselective duplex formation between 4a$_{NHCO}$ and *rac*-1a$_{NHCO}$ (run 14, Table 1). Full (**a**) and partial (**b** and **c**) ^{1}H NMR spectra of **4a** (0.50 mM), *rac*-**1a** (0.50 mM), **4a**·(*R*,*R*)-**1a** (0.50 mM), **4a**·(*S*,*S*)-**1a** (0.50 mM), and a mixture of **4a** (0.50 mM) and *rac*-**1a** (1.0 mM) in $CDCl_3$ at 25 °C.

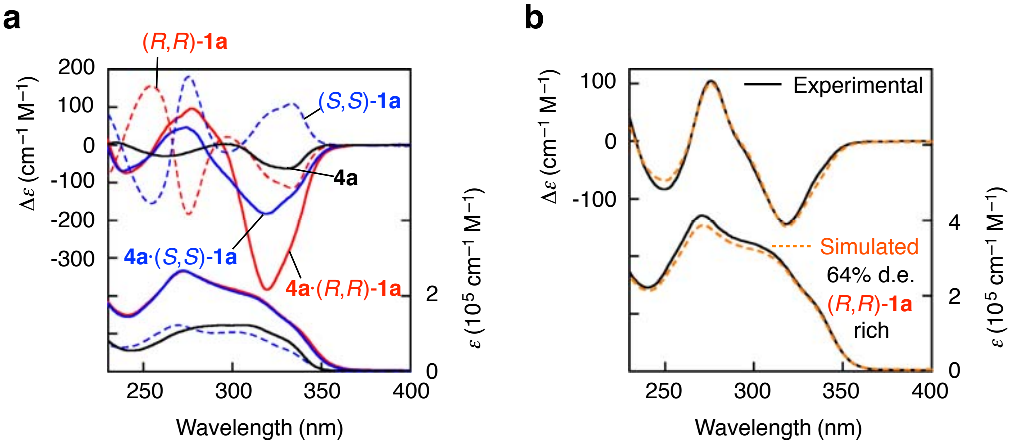

Supplementary Figure 57 | Diastereoselective duplex formation between 4a$_{NHCO}$ and *rac*-1a$_{NHCO}$. (**a**) CD and absorption spectra (0.50 mM) of **4a**, (*R*,*R*)-**1a**, (*S*,*S*)-**1a**, **4a**·(*R*,*R*)-**1a**, and **4a**·(*S*,*S*)-**1a** in $CDCl_3$ at ambient temperature. (**b**) Experimental and simulated CD (d.e. = 64%) and absorption spectra for a mixture of **4a** (0.50 mM) and *rac*-**1a** (1.0 mM) in $CDCl_3$ at ambient temperature.

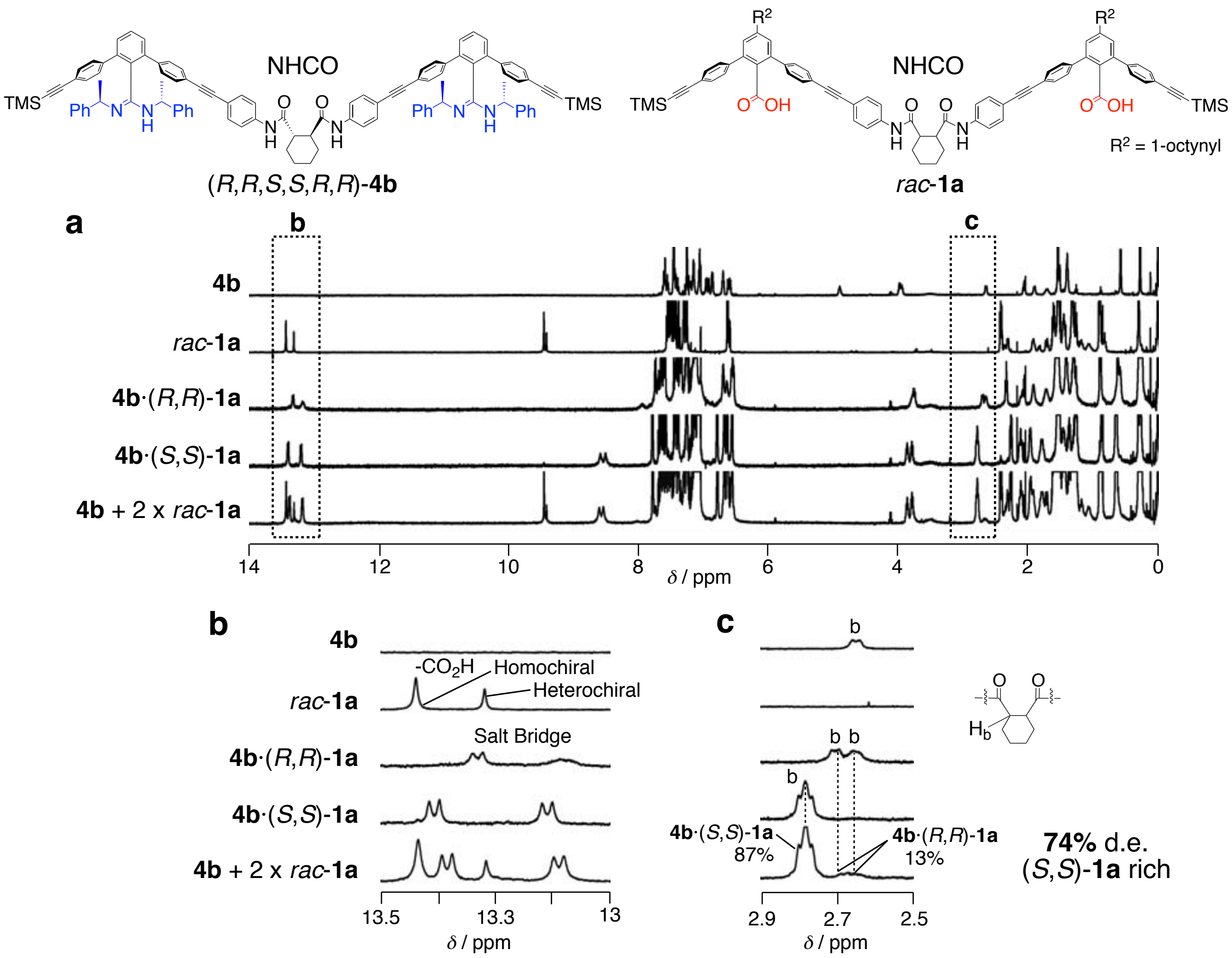

Supplementary Figure 58 | Diastereoselective duplex formation between $\mathbf{4b}_{\mathbf{NHCO}}$ and *rac*-$\mathbf{1a}_{\mathbf{NHCO}}$ (run 15, Table 1). Full (**a**) and partial (**b** and **c**) ^{1}H NMR spectra of **4b** (0.50 mM), *rac*-**1a** (0.50 mM), **4b**·(*R*,*R*)-**1a** (0.50 mM), **4b**·(*S*,*S*)-**1a** (0.50 mM), and a mixture of **4b** (0.50 mM) and *rac*-**1a** (1.0 mM) in $CDCl_3$ at 25 °C.

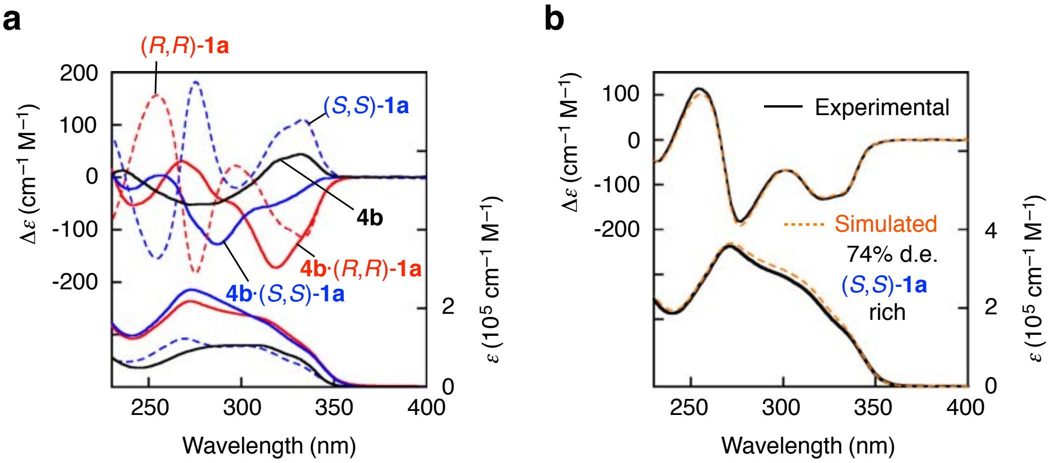

Supplementary Figure 59 | Diastereoselective duplex formation between $\mathbf{4b}_{\mathbf{NHCO}}$ and *rac*-$\mathbf{1a}_{\mathbf{NHCO}}$. (**a**) CD and absorption spectra (0.50 mM) of **4b**, (*R*,*R*)-**1a**, (*S*,*S*)-**1a**, **4b**·(*R*,*R*)-**1a**, and **4b**·(*S*,*S*)-**1a** in $CDCl_3$ at ambient temperature. (**b**) Experimental and simulated CD (d.e. = 74%) and absorption spectra for a mixture of **4b** (0.50 mM) and *rac*-**1a** (1.0 mM) in $CDCl_3$ at ambient temperature.

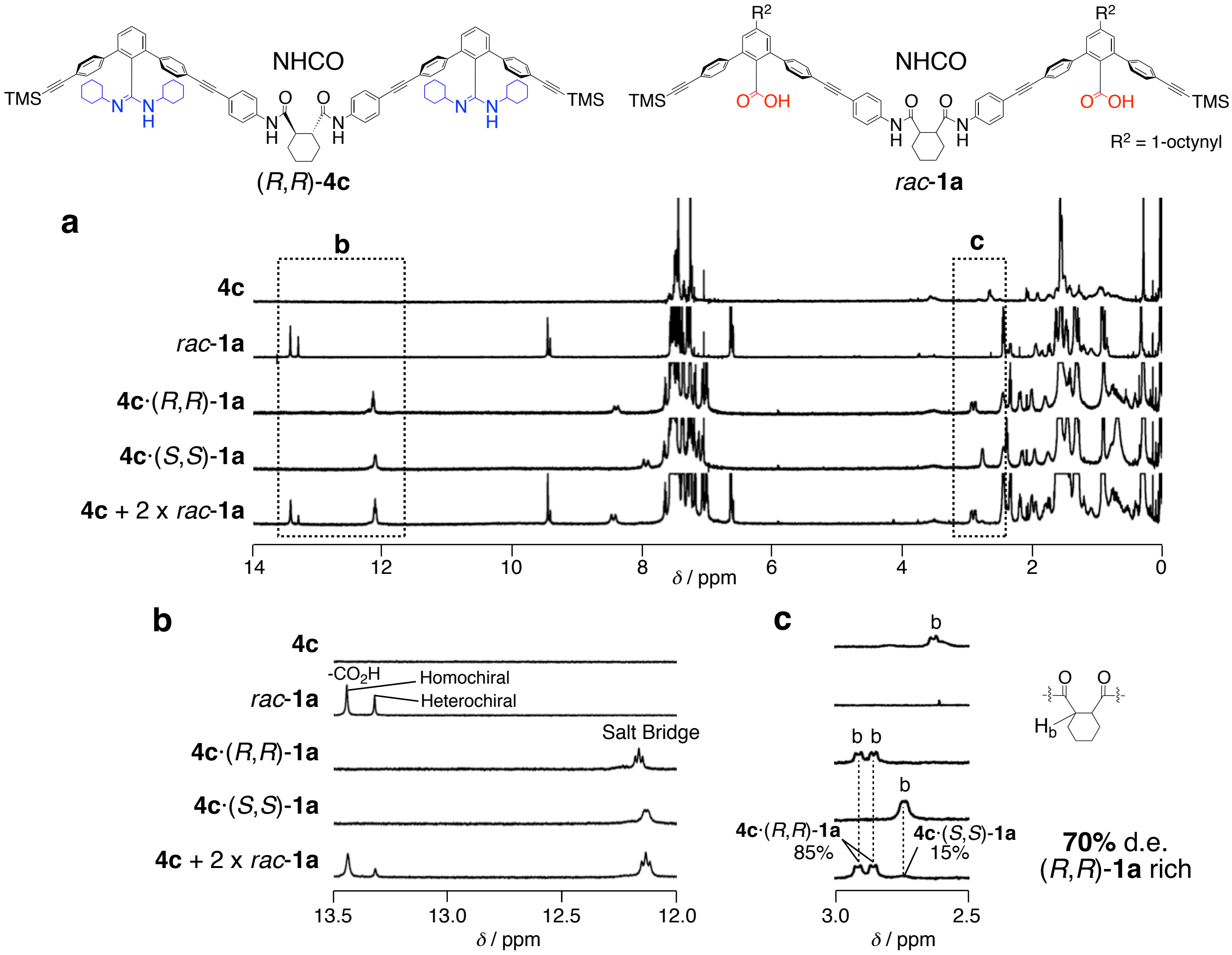

Supplementary Figure 60 | Diastereoselective duplex formation between 4c$_{NHCO}$ and *rac*-1a$_{NHCO}$ (run 16, Table 1). Full (**a**) and partial (**b** and **c**) ^{1}H NMR spectra of **4c** (0.50 mM), *rac*-**1a** (0.50 mM), **4c**·(*R*,*R*)-**1a** (0.50 mM), **4c**·(*S*,*S*)-**1a** (0.50 mM), and a mixture of **4c** (0.50 mM) and *rac*-**1a** (1.0 mM) in $CDCl_3$ at 25 °C.

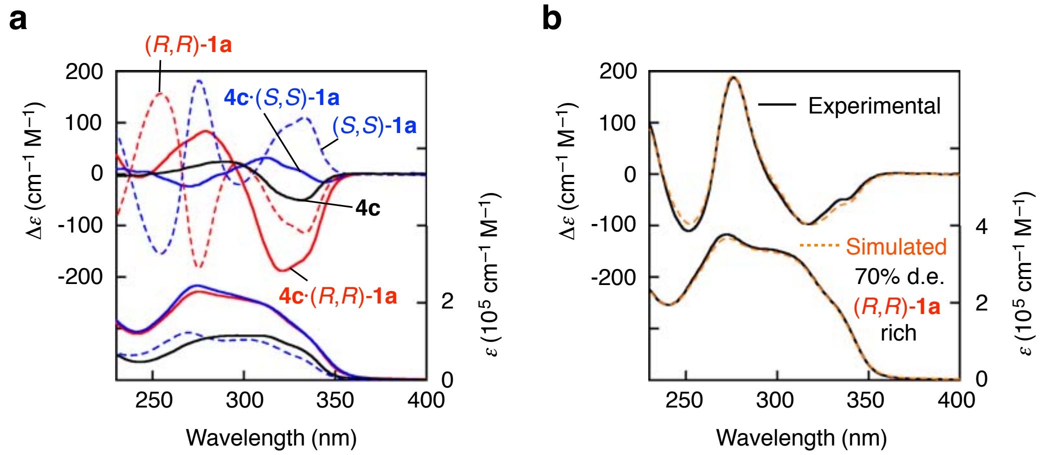

Supplementary Figure 61 | Diastereoselective duplex formation between 4c$_{NHCO}$ and *rac*-1a$_{NHCO}$. (**a**) CD and absorption spectra (0.50 mM) of **4c**, (*R*,*R*)-**1a**, (*S*,*S*)-**1a**, **4c**·(*R*,*R*)-**1a**, and **4c**·(*S*,*S*)-**1a** in $CDCl_3$ at ambient temperature. (**b**) Experimental and simulated CD (d.e. = 70%) and absorption spectra for a mixture of **4c** (0.50 mM) and *rac*-**1a** (1.0 mM) in $CDCl_3$ at ambient temperature.

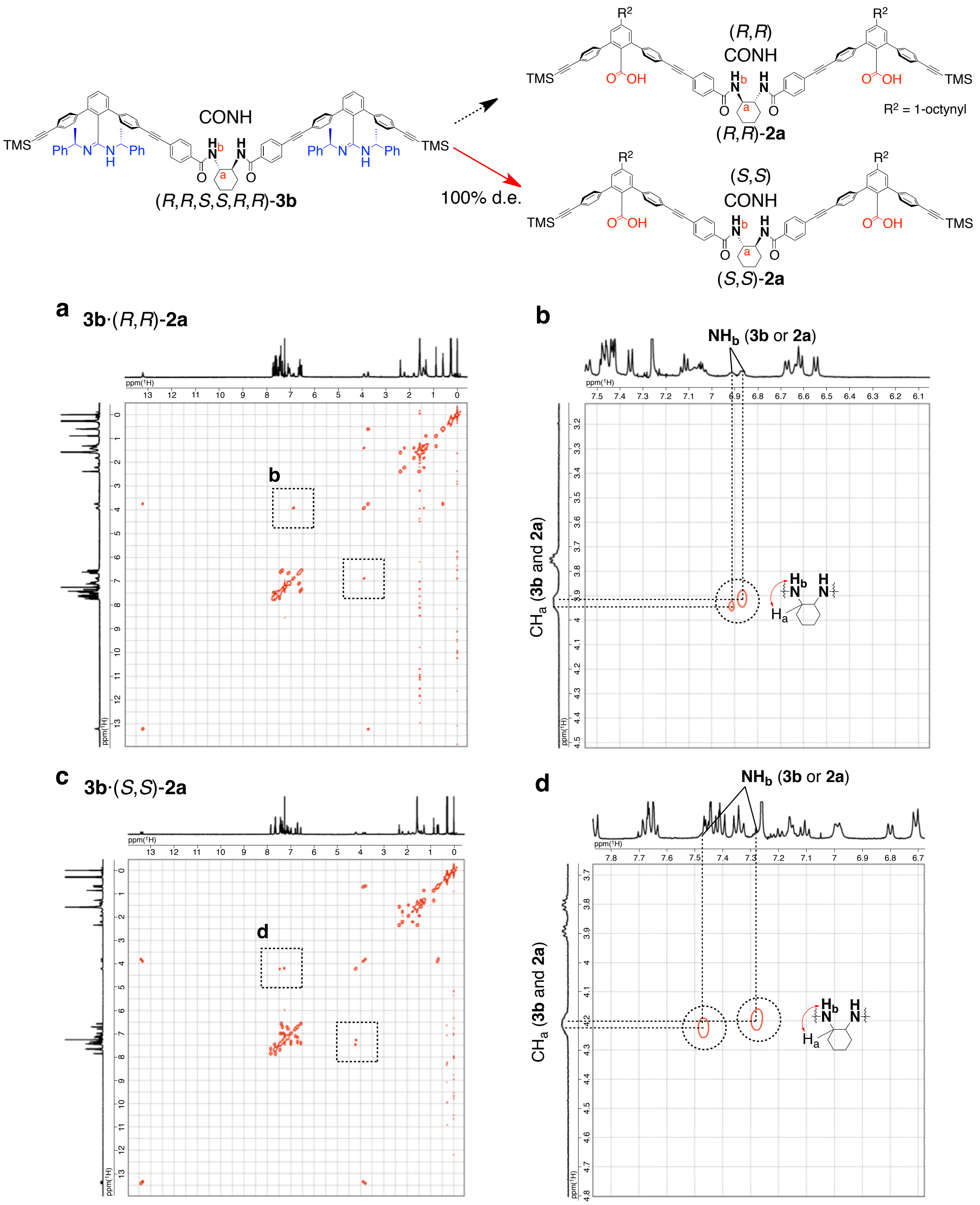

Supplementary Figure 62 | Amide NH proton assignments for 3b·(*R*,*R*)-2a (CONH·CONH) and 3b·(*S*,*S*)-2a (CONH·CONH). Full (**a** and **c**) and partial (**b** and **d**) *g*COSY spectra (1.0 mM) of **3b**·(*R*,*R*)-**2a** (**a**, **b**) and **3b**·(*S*,*S*)-**2a** (**c**, **d**) in $CDCl_3$ at 25 °C.

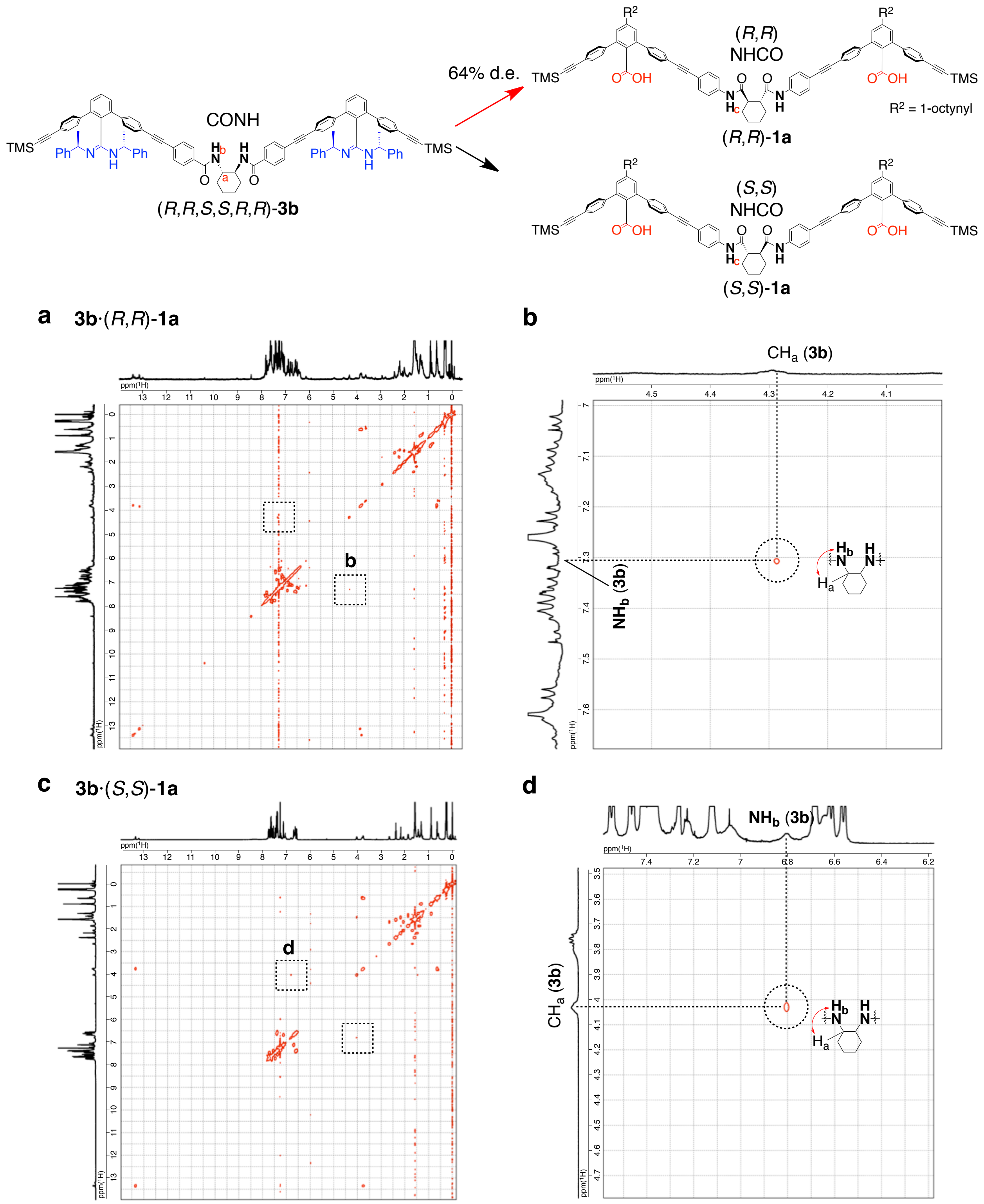

Supplementary Figure 63 | Amide NH proton assignments for 3b·(*R*,*R*)-1a (CONH·NHCO) and 3b·(*S*,*S*)-1a (CONH·NHCO). Full (**a** and **c**) and partial (**b** and **d**) *g*COSY spectra (1.0 mM) of **3b**·(*R*,*R*)-**1a** (**a**, **b**) and **3b**·(*S*,*S*)-**1a** (**c**, **d**) in $CDCl_3$ at 25 °C.

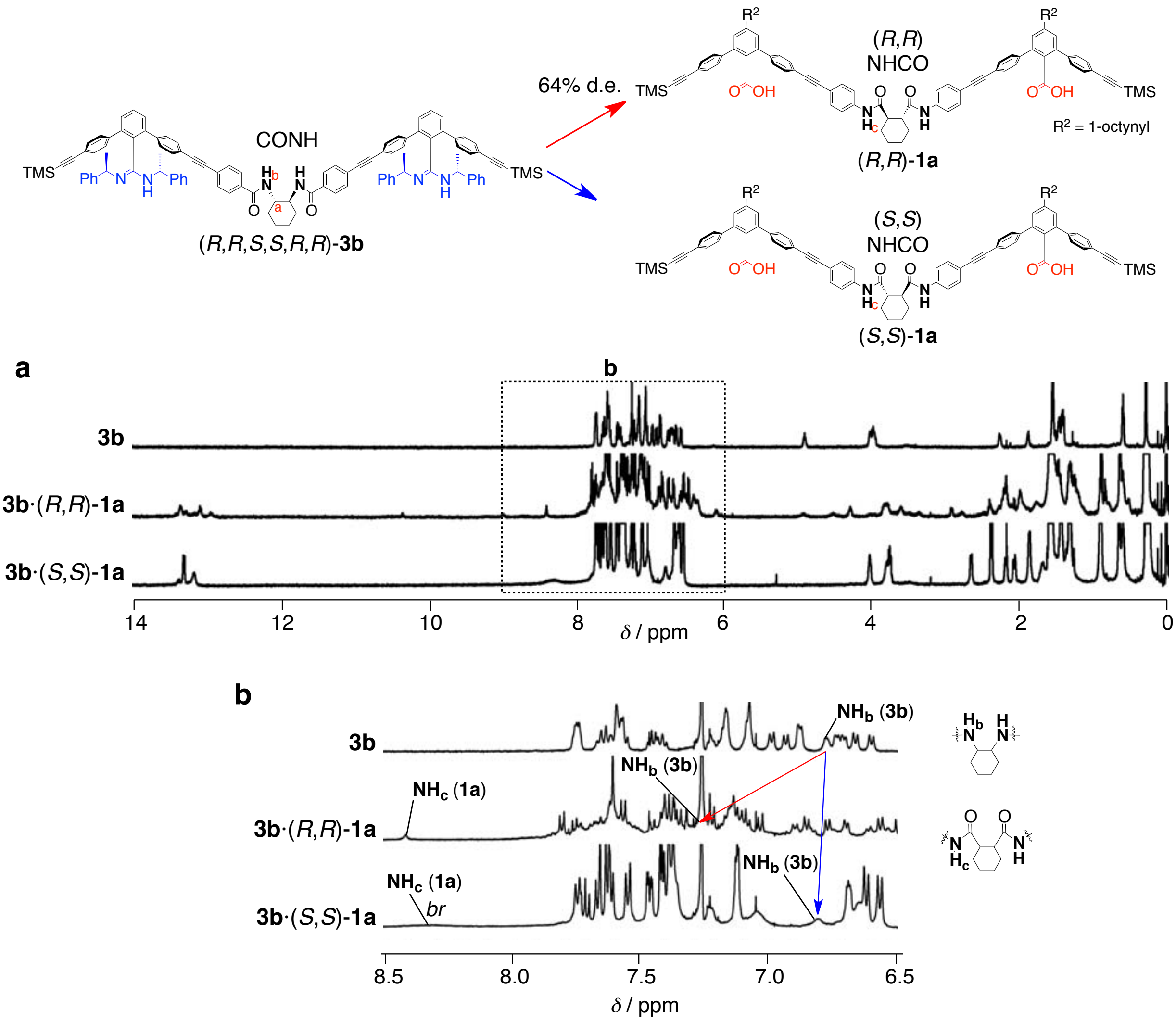

Supplementary Figure 64 | Amide NH proton assignments for 3b·(*R*,*R*)-1a (CONH·NHCO) and 3b·(*S*,*S*)-1a (CONH·NHCO). Full (**a**) and partial (**b**) ^{1}H NMR spectra (1.0 mM) of **3b**, **3b**·(*R*,*R*)-**1a**, and **3b**·(*S*,*S*)-**1a** in $CDCl_3$ at 25 °C. The signals for the NH_c protons of **1a** in **3b**·(*R*,*R*)-**1a** and **3b**·(*S*,*S*)-**1a** were identified upon the addition of CD_3OD. The NH_b proton assignments were done by *g*COSY measurements (see Supplementary Fig. 63).

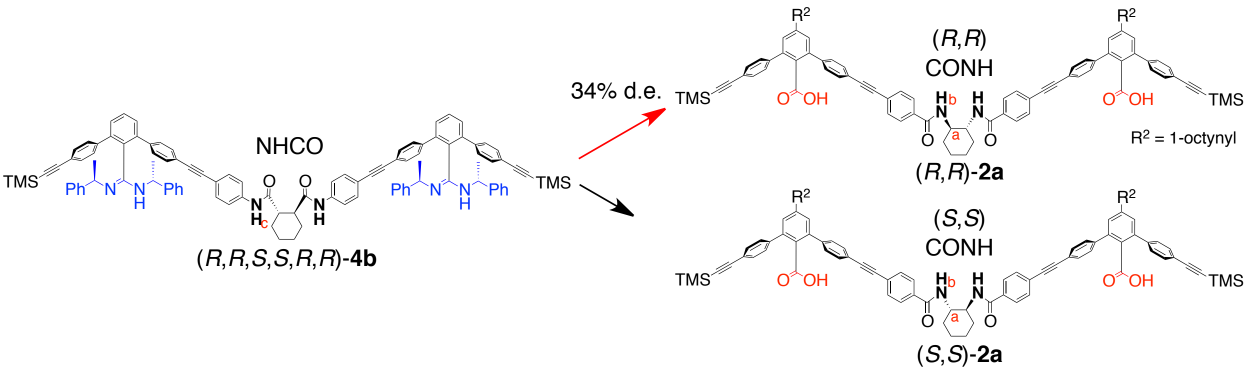

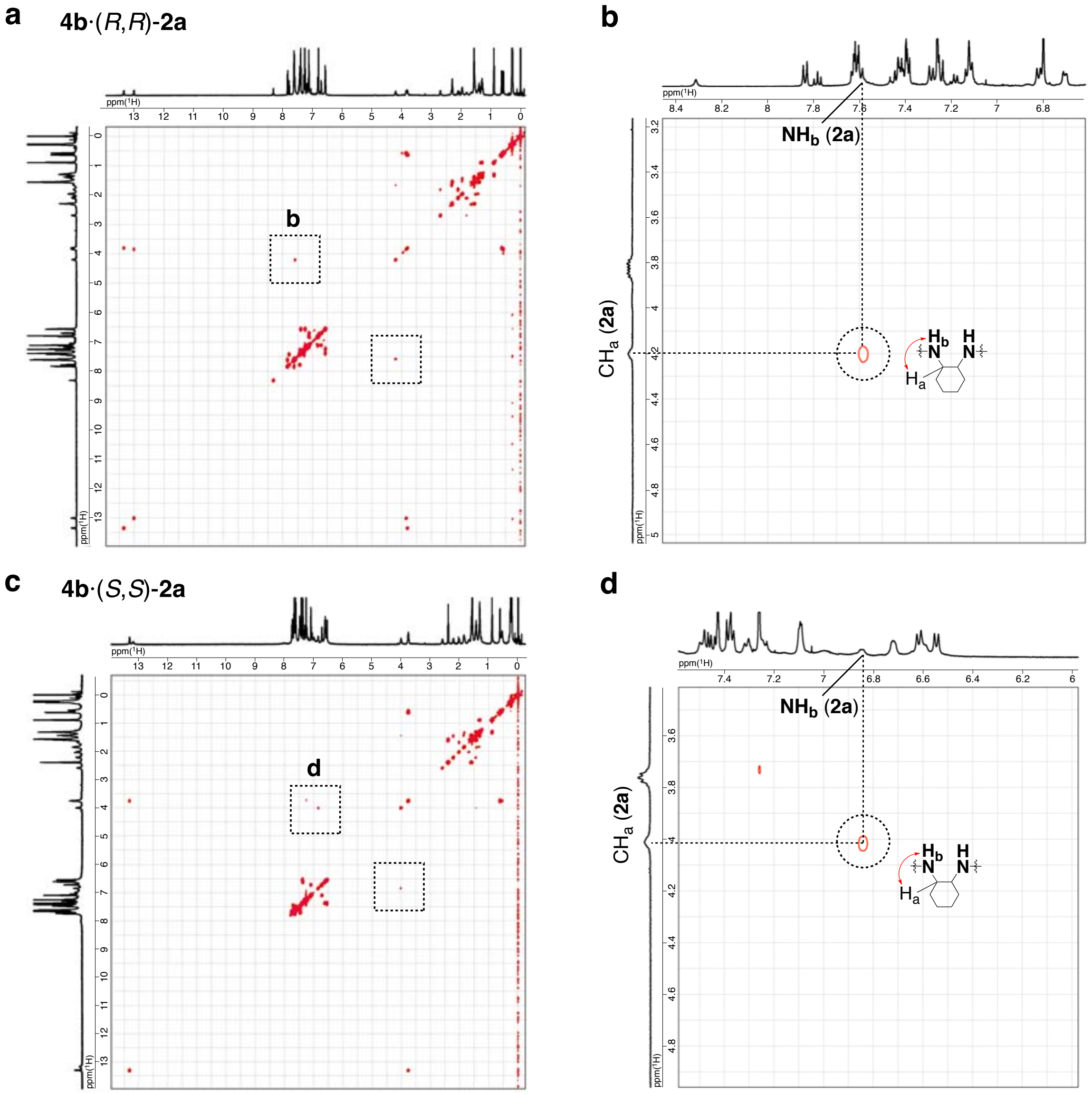

Supplementary Figure 65 | Amide NH proton assignments for 4b·(*R*,*R*)-2a (NHCO·CONH) and 4b·(*S*,*S*)-2a (NHCO·CONH). Full (**a** and **c**) and partial (**b** and **d**) *g*COSY NMR spectra (1.0 mM) of **4b**·(*R*,*R*)-**2a** (**a**, **b**) and **4b**·(*S*,*S*)-**2a** (**c**, **d**) in $CDCl_3$ at 25 °C.

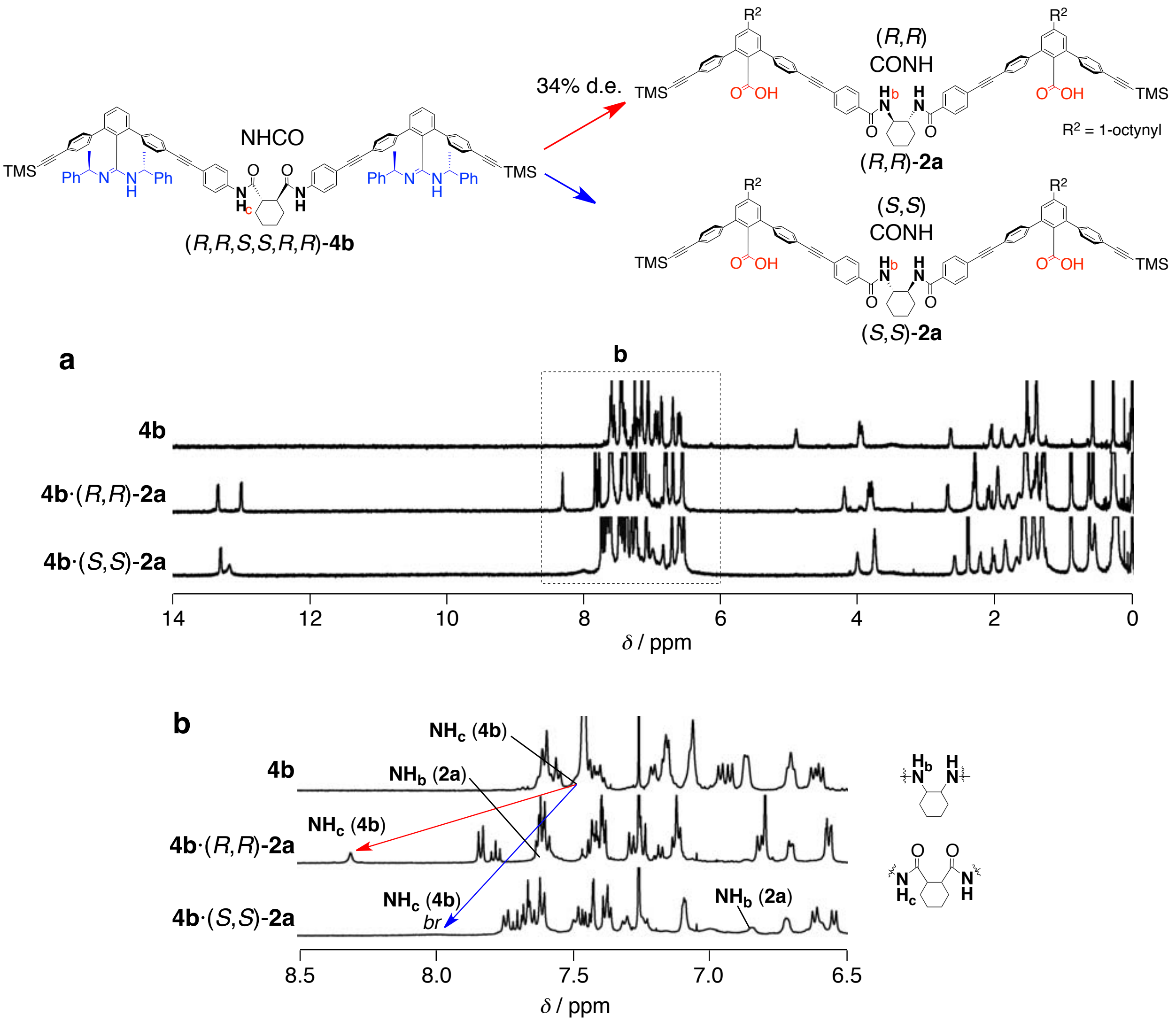

Supplementary Figure 66 | Amide NH proton assignments for 4b·(*R*,*R*)-2a (NHCO·CONH) and 4b·(*S*,*S*)-2a (NHCO·CONH). Full (**a**) and partial (**b**) ^{1}H NMR spectra (1.0 mM) of **4b**, **4b**·(*R*,*R*)-**2a**, and **4b**·(*S*,*S*)-**2a** in $CDCl_3$ at 25 °C. The signals for the NH_c protons of **4b** in **4b**·(*R*,*R*)-**2a** and **4b**·(*S*,*S*)-**2a** were identified upon the addition of CD_3OD. The NH_b proton assignments were done by *g*COSY measurements (see Supplementary Fig. 65).

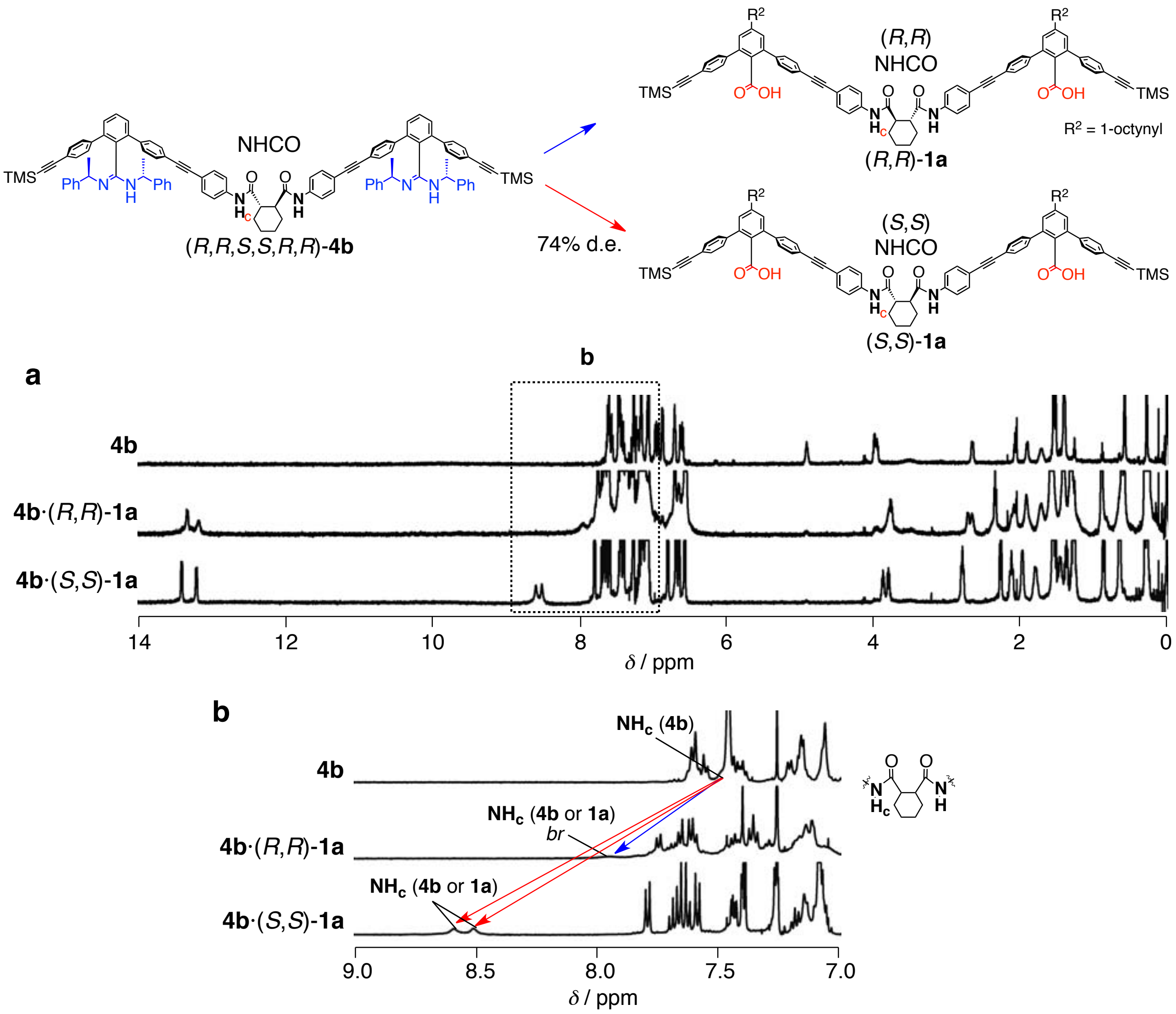

Supplementary Figure 67 | Amide NH proton assignments for 4b·(*R*,*R*)-1a (NHCO·NHCO) and 4b·(*S*,*S*)-1a (NHCO·NHCO). Full (**a**) and partial (**b**) ^{1}H NMR spectra (1.0 mM) of **4b**, **4b**·(*R*,*R*)-**1a**, and **4b**·(*S*,*S*)-**1a** in $CDCl_3$ at 25 °C. The signals for the NH_c protons of **4b** and **1a** in **4b**·(*R*,*R*)-**1a** and **4b**·(*S*,*S*)-**1a** were identified upon the addition of CD_3OD.

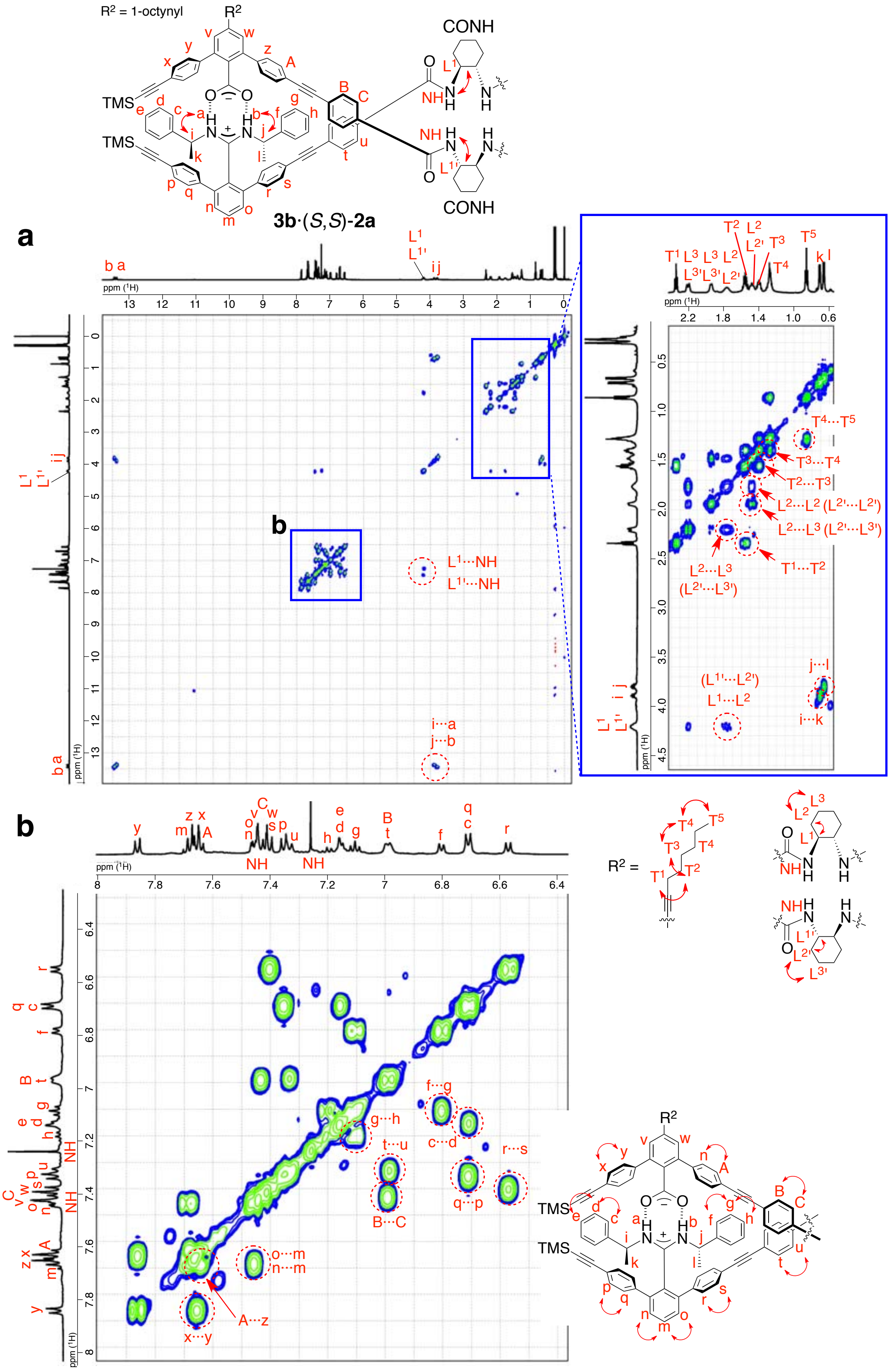

Supplementary Figure 68 | 2D NMR spectra of (*R*,*R*,*S*,*S*,*R*,*R*)-3b·(*S*,*S*)-2a with the same amide sequence (CONH·CONH). Full (**a**) and Partial (**b**) *g*COSY spectra of **3b**·(*S*,*S*)-**2a** (2.0 mM) in $CDCl_3$ at 25 °C.

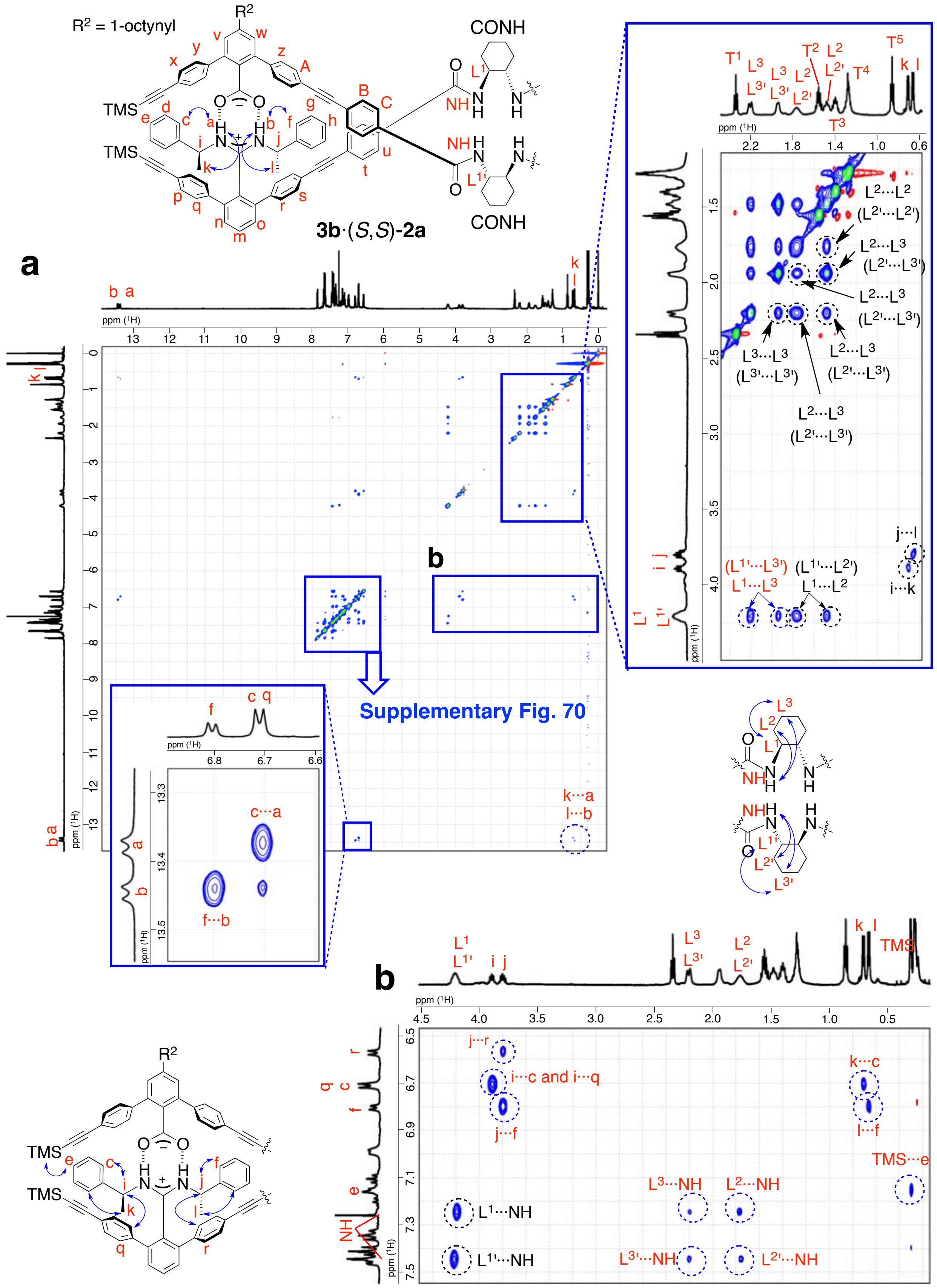

Supplementary Figure 69 | 2D NMR spectra of (*R*,*R*,*S*,*S*,*R*,*R*)-3b·(*S*,*S*)-2a with the same amide sequence (CONH·CONH). Full (**a**) and partial (**b**) NOESY spectra (mixing time = 500 ms) of **3b**·(*S*,*S*)-**2a** (2.0 mM) in $CDCl_3$ at 25 °C.

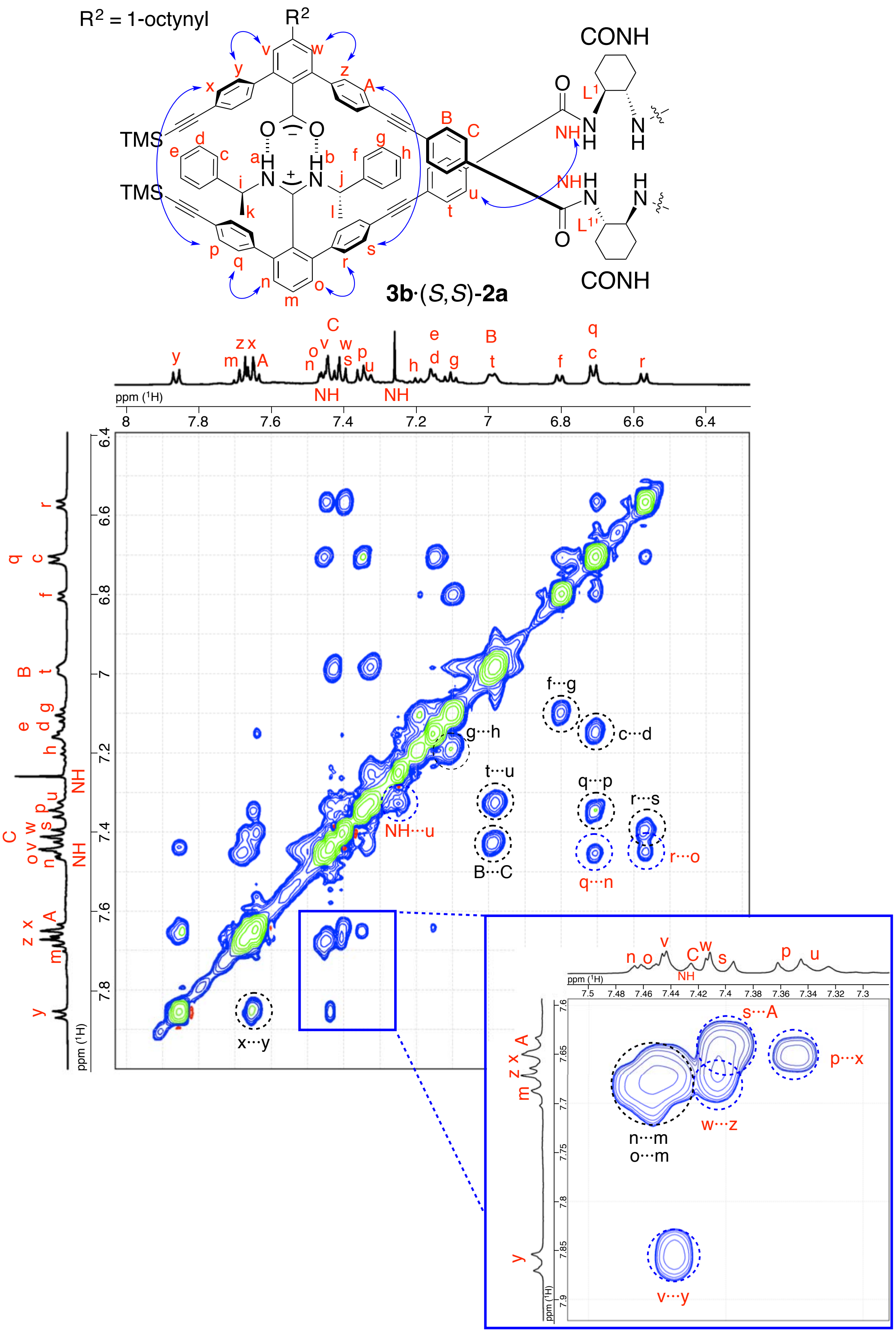

Supplementary Figure 70 | 2D NMR spectra of (*R*,*R*,*S*,*S*,*R*,*R*)-3b·(*S*,*S*)-2a with the same amide sequence (CONH·CONH). Partial NOESY spectra (mixing time = 500 ms) of **3b**·(*S*,*S*)-**2a** (2.0 mM) in $CDCl_3$ at 25 °C.

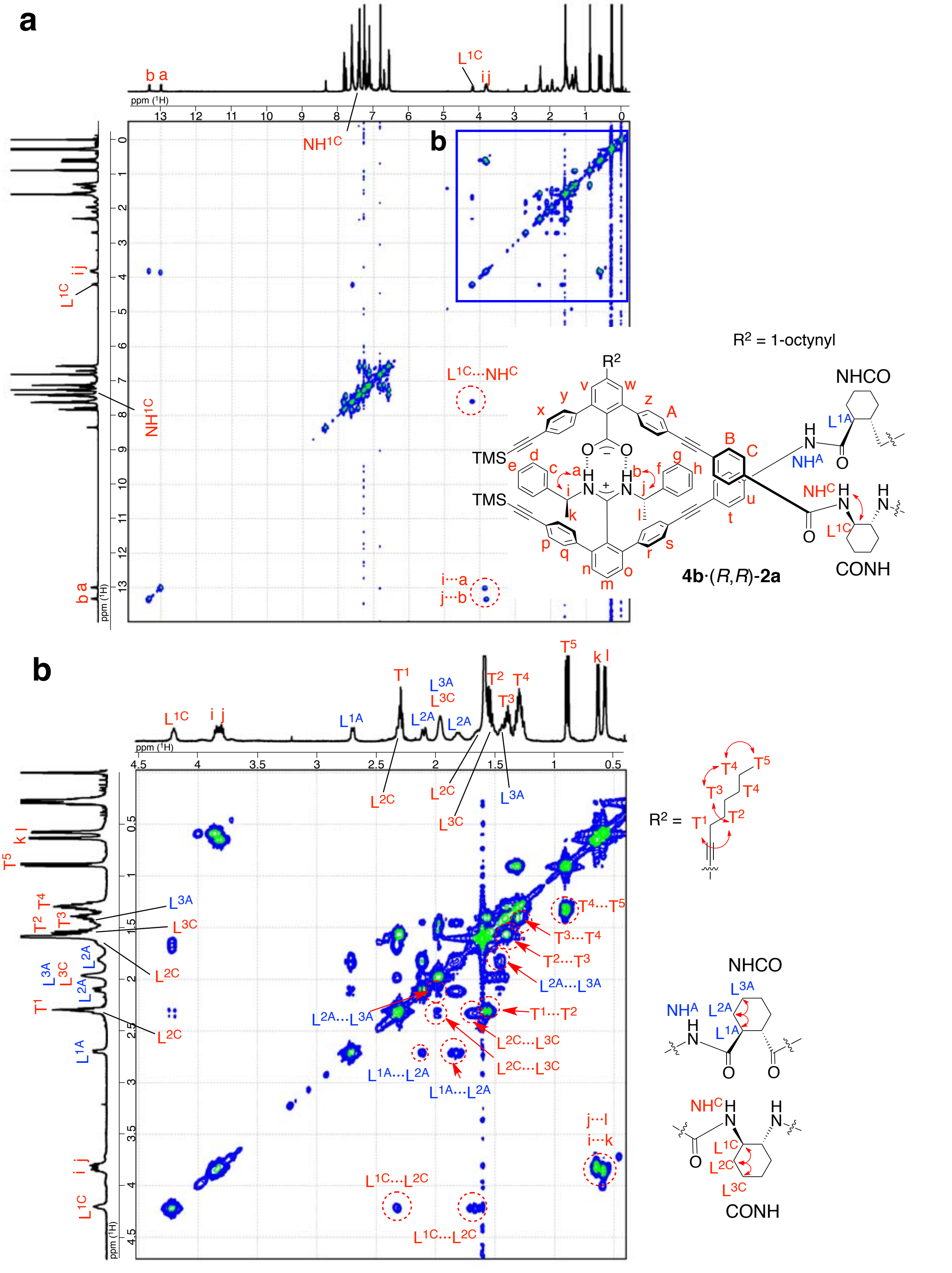

Supplementary Figure 71 | 2D NMR spectra of (*R*,*R*,*S*,*S*,*R*,*R*)-4b·(*R*,*R*)-2a with the different amide sequence (NHCO·CONH). Full (**a**) and Partial (**b**) *g*COSY spectrum of **4b**·(*R*,*R*)-**2a** (2.0 mM) in $CDCl_3$ at 25 °C.

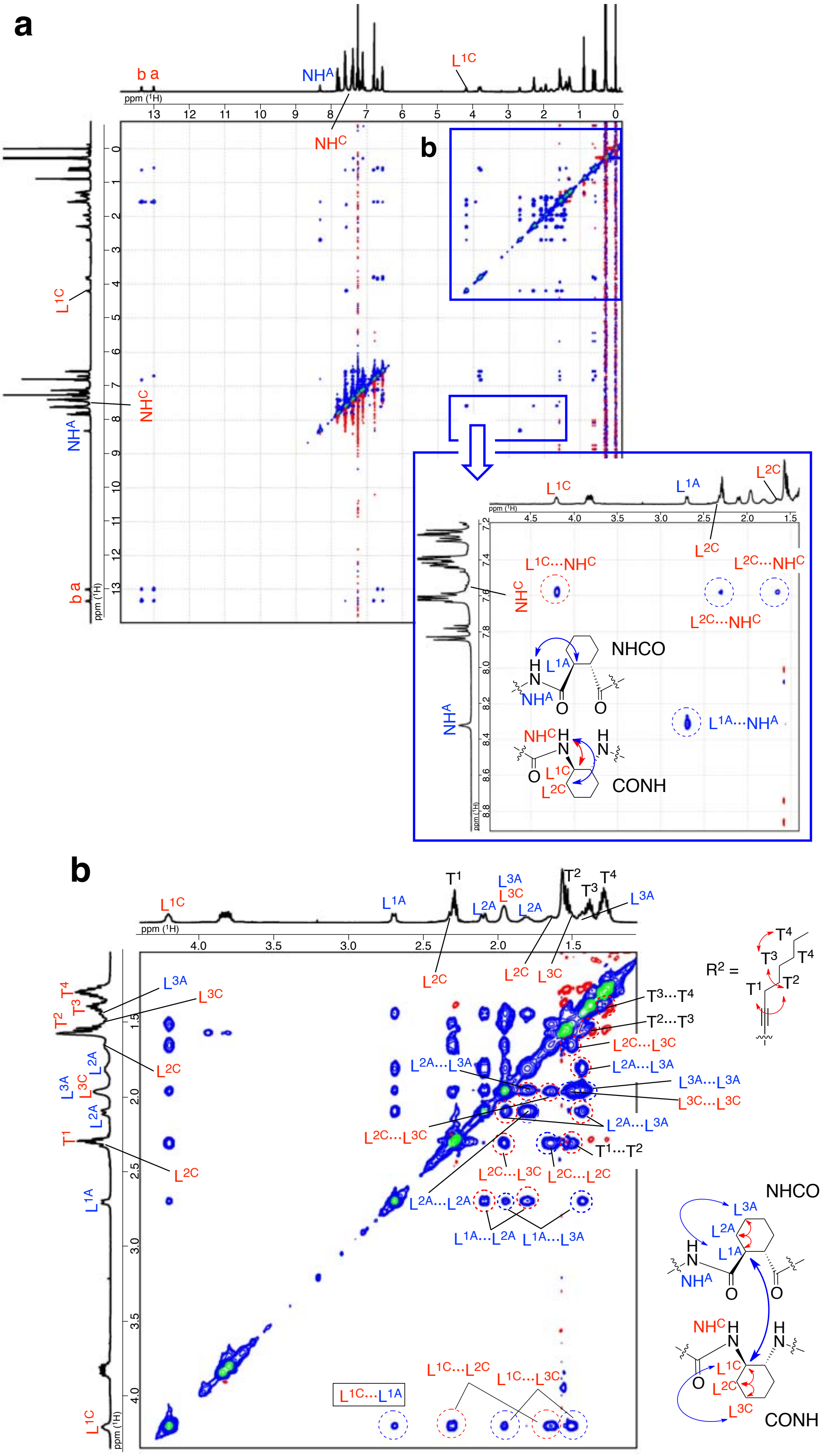

Supplementary Figure 72 | 2D NMR spectra of (*R*,*R*,*S*,*S*,*R*,*R*)-4b·(*R*,*R*)-2a with the different amide sequence (NHCO·CONH). Full (**a**) and partial (**b**) NOESY spectra (mixing time = 500 ms) of **4b**·(*R*,*R*)-**2a** (2.0 mM) in $CDCl_3$ at 25 °C.

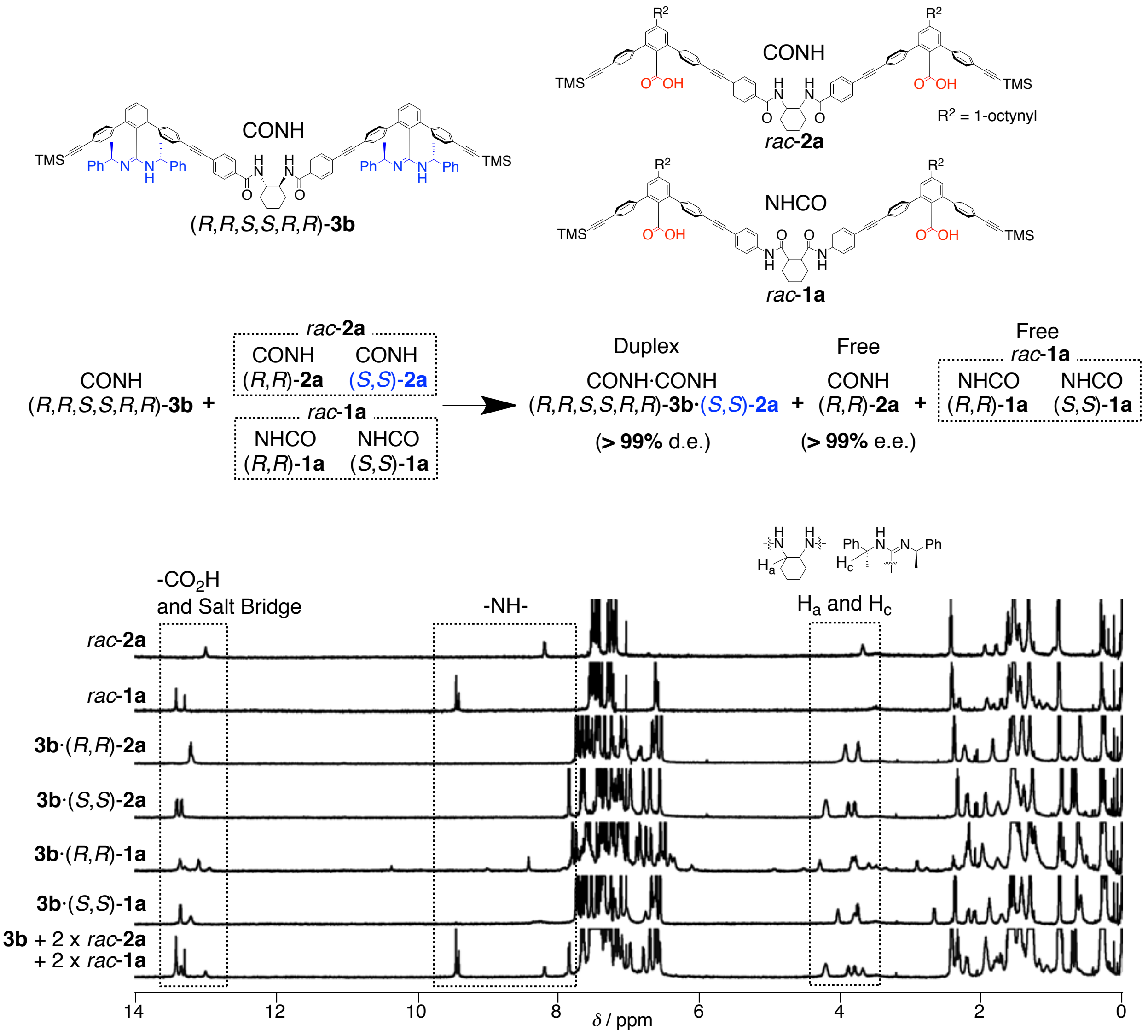

Supplementary Figure 73 | Diastereo- and sequence-selective duplex formation between $\mathbf{3b}_{\mathbf{CONH}}$ and a mixture of *rac*-$\mathbf{2a}_{\mathbf{CONH}}$ and *rac*-$\mathbf{1a}_{\mathbf{NHCO}}$. Full ^{1}H NMR spectra of *rac*-**2a** (0.50 mM), *rac*-**1a** (0.50 mM), **3b**·(*R*,*R*)-**2a** (0.50 mM), **3b**·(*S*,*S*)-**2a** (0.50 mM), **3b**·(*R*,*R*)-**1a** (0.50 mM), **3b**·(*S*,*S*)-**1a** (0.50 mM), and a mixture of **3b** (0.50 mM), *rac*-**2a** (1.0 mM), and *rac*-**1a** (1.0 mM) in $CDCl_3$ at 25 °C. For expanded ^{1}H NMR spectra indicated by the squares, see Fig. 7a.

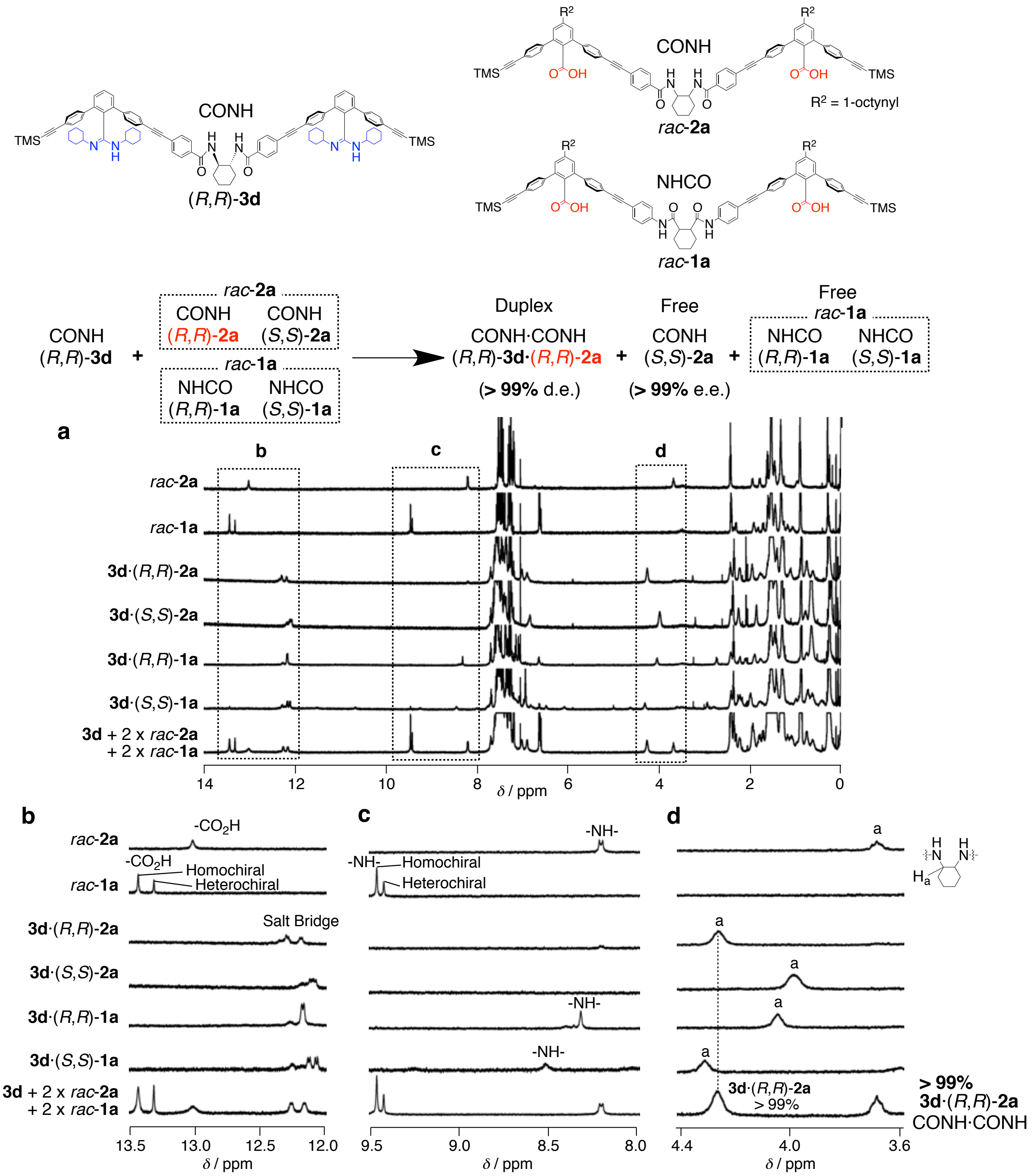

Supplementary Figure 74 | Diastereo- and sequence-selective duplex formation between 3d$_{CONH}$ and a mixture of *rac*-2a$_{CONH}$ and *rac*-1a$_{NHCO}$. Full (**a**) and partial (**b**, **c**, and **d**) ^{1}H NMR spectra of *rac*-**2a** (0.50 mM), *rac*-**1a** (0.50 mM), **3d**·(*R*,*R*)-**2a** (0.50 mM), **3d**·(*S*,*S*)-**2a** (0.50 mM), **3d**·(*R*,*R*)-**1a** (0.50 mM), **3d**·(*S*,*S*)-**1a** (0.50 mM), and a mixture of **3d** (0.50 mM), *rac*-**2a** (1.0 mM), and *rac*-**1a** (1.0 mM) in $CDCl_3$ at 25 °C.

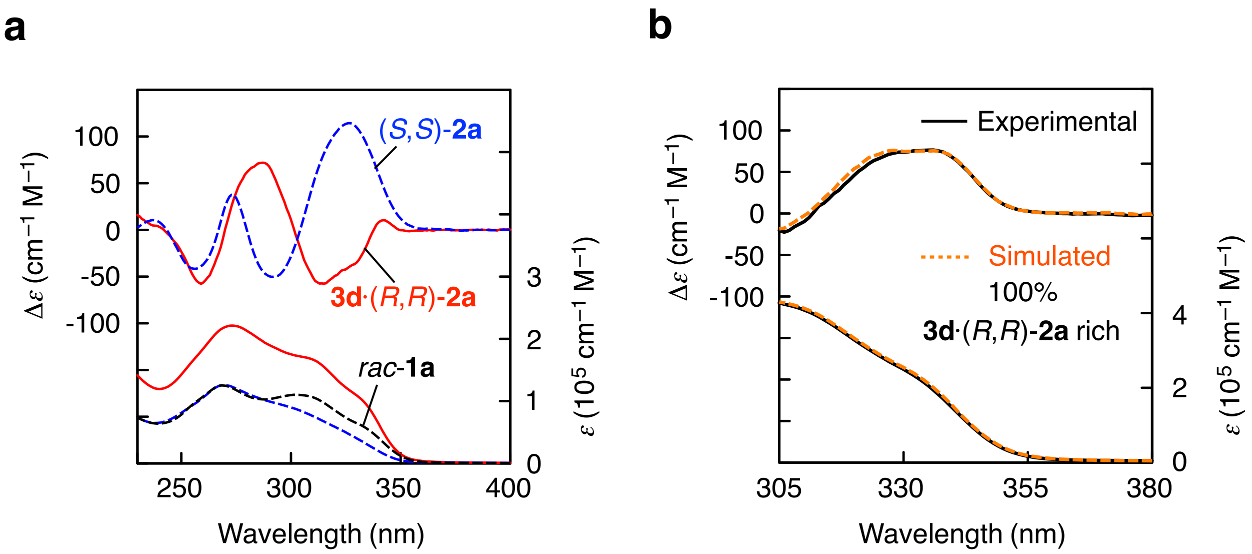

Supplementary Figure 75 | Diastereo- and sequence-selective duplex formation between 3d$_{\mathrm{CONH}}$ and a mixture of *rac*-2a$_{\mathrm{CONH}}$ and *rac*-1a$_{\mathrm{NHCO}}$. (**a**) CD and absorption spectra (0.50 mM) of **3d**·(*R*,*R*)-**2a**, (*S*,*S*)-**2a**, and *rac*-**1a** in $CDCl_3$ at ambient temperature. (**b**) Experimental and simulated CD and absorption spectra for a mixture of **3d** (0.50 mM), *rac*-**2a** (1.0 mM), and *rac*-**1a** (1.0 mM) in $CDCl_3$ at ambient temperature.

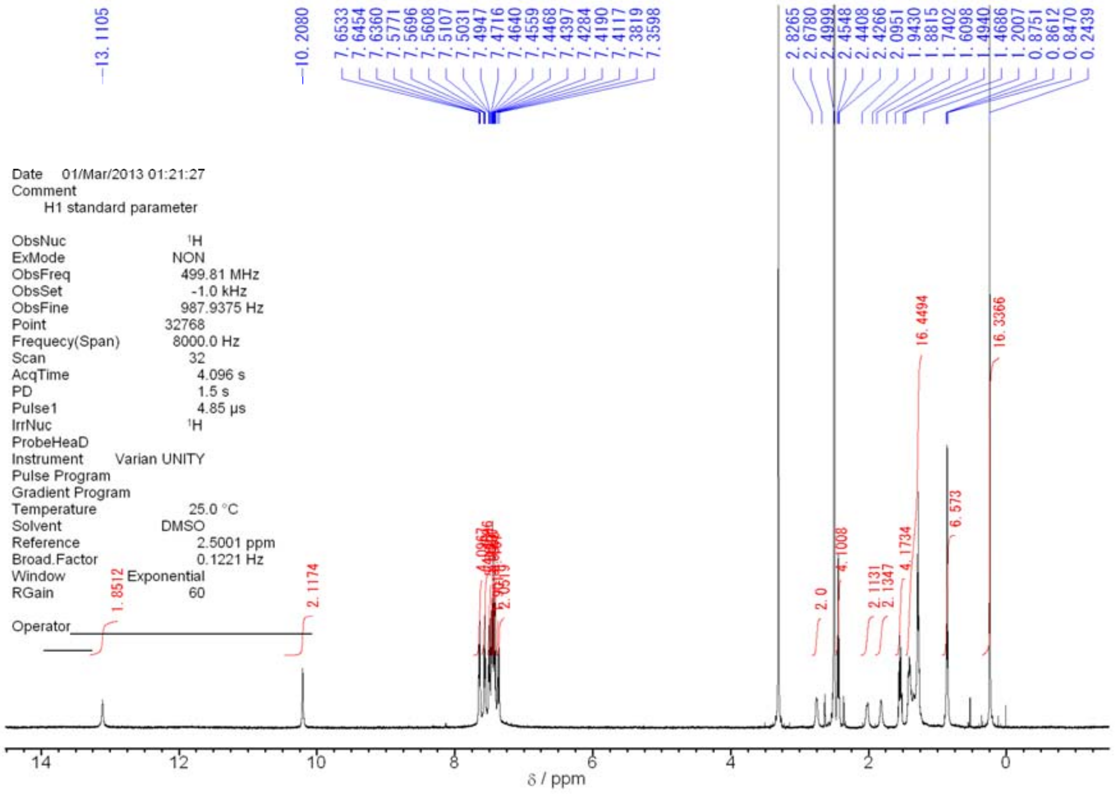

Supplementary Figure 76 | ^{1}H NMR (DMSO-d_6, 500 MHz) spectrum of (*R*,*R*)-**1a**.

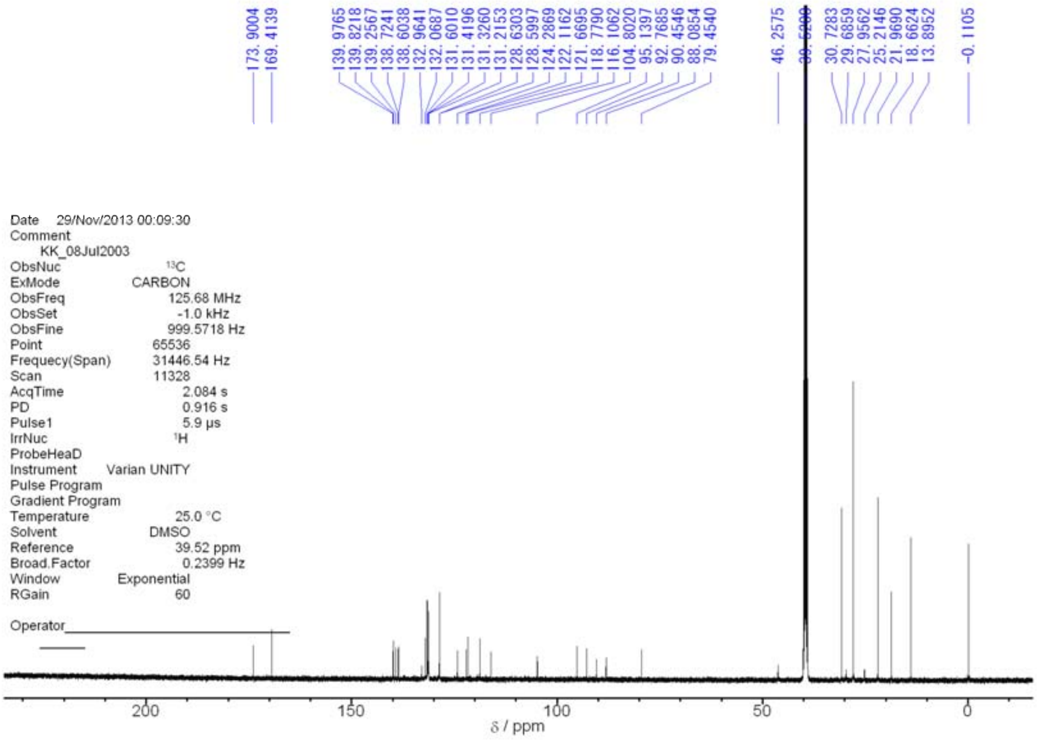

Supplementary Figure 77 | ^{13}C NMR (DMSO-d_6, 125 MHz) spectrum of (*R*,*R*)-**1a**.

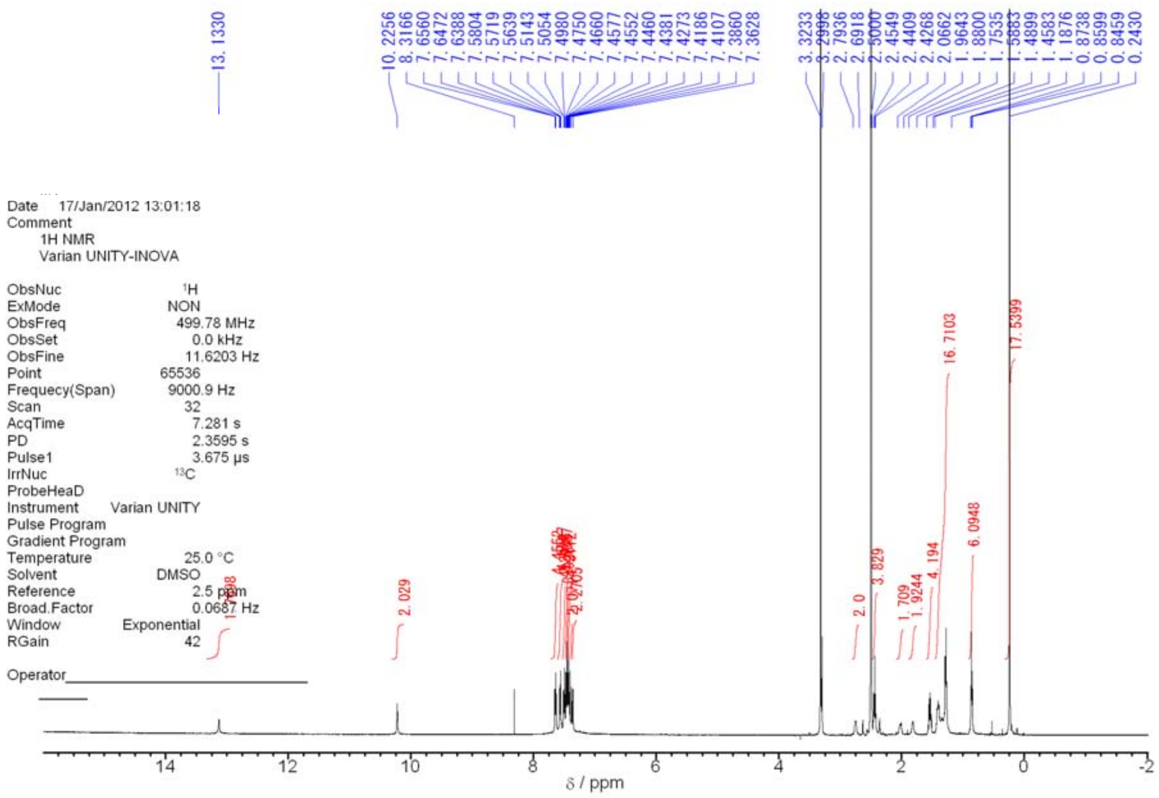

Supplementary Figure 78 | ^{1}H NMR (DMSO-d_6, 500 MHz) spectrum of (*S*,*S*)-**1a**.

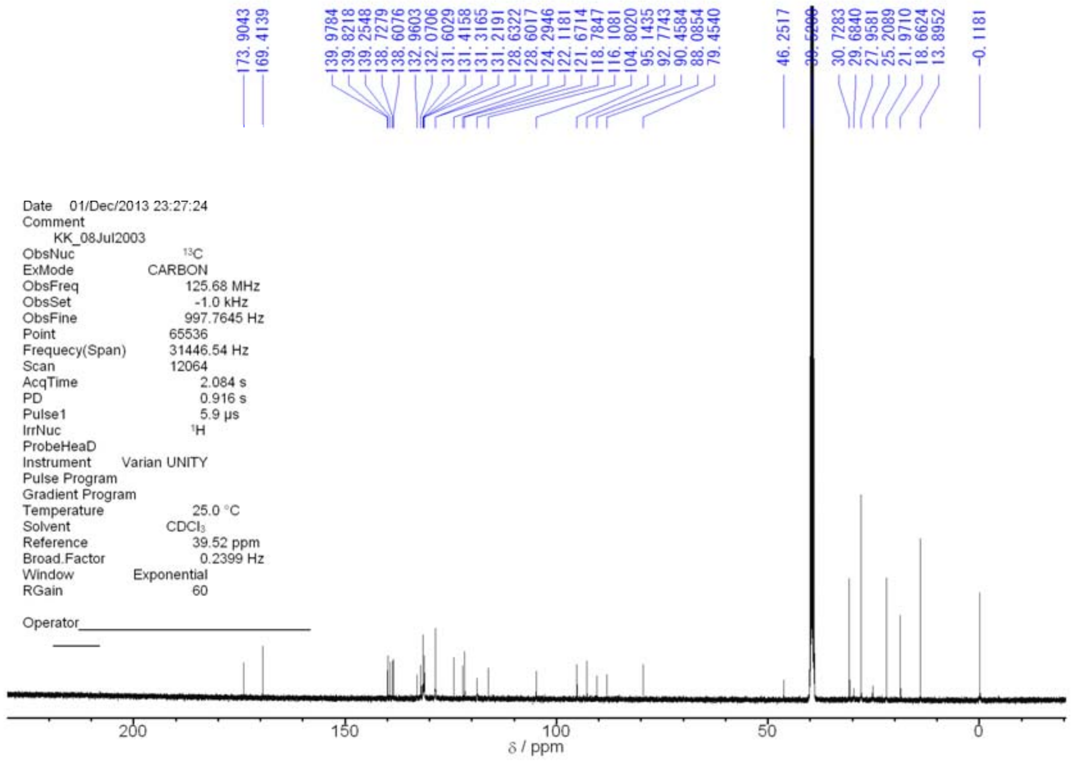

Supplementary Figure 79 | ^{13}C NMR (DMSO-d_6, 125 MHz) spectrum of (*S*,*S*)-**1a**.

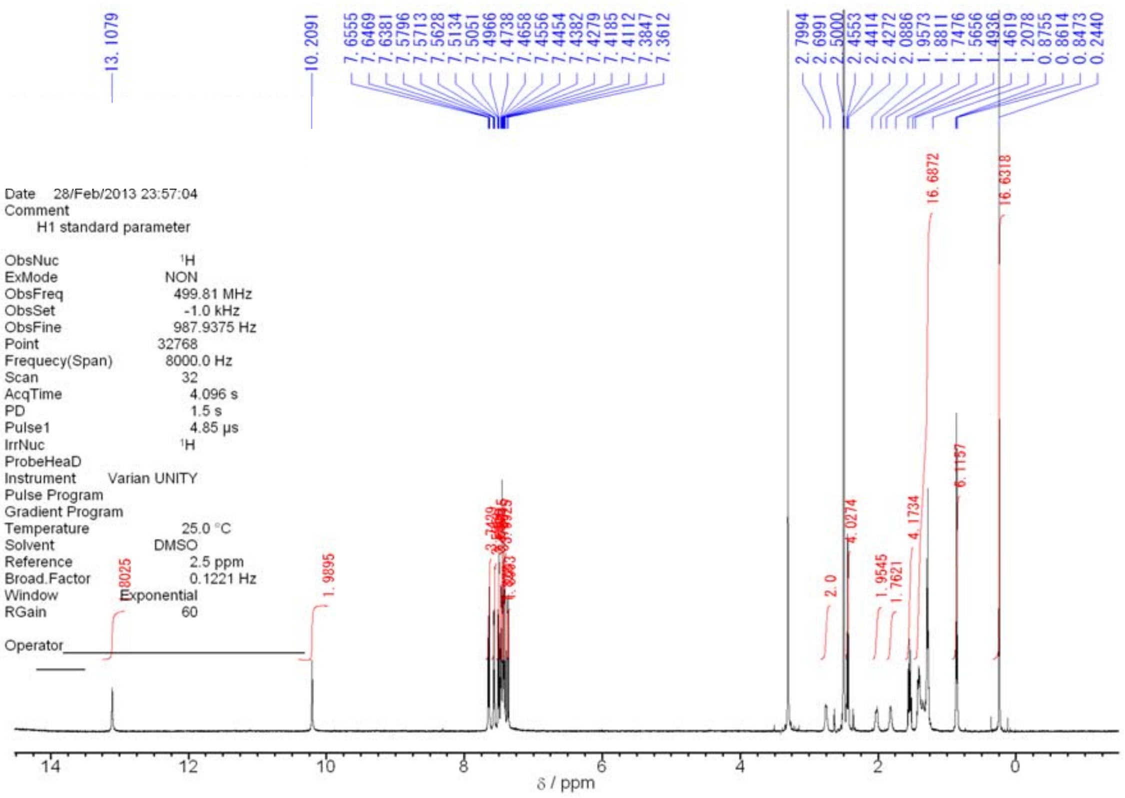

Supplementary Figure 80 | ^{1}H NMR (DMSO-d_6, 500 MHz) spectrum of *rac*-**1a**.

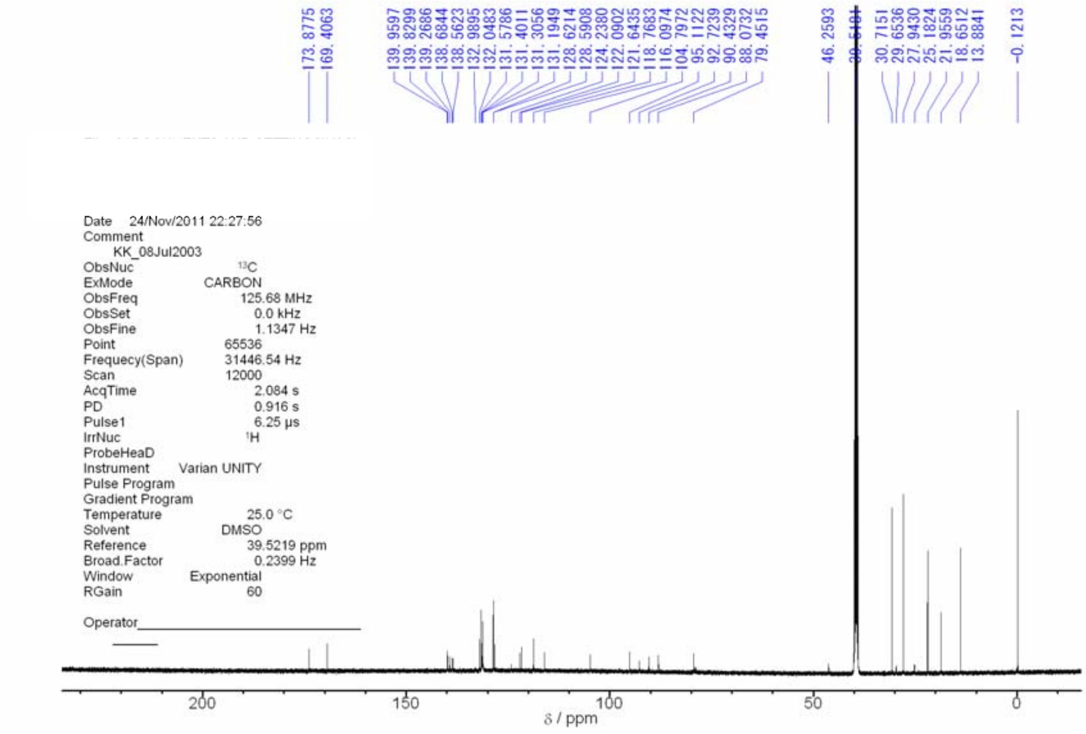

Supplementary Figure 81 | ^{13}C NMR (DMSO-d_6, 125 MHz) spectrum of *rac*-**1a**.

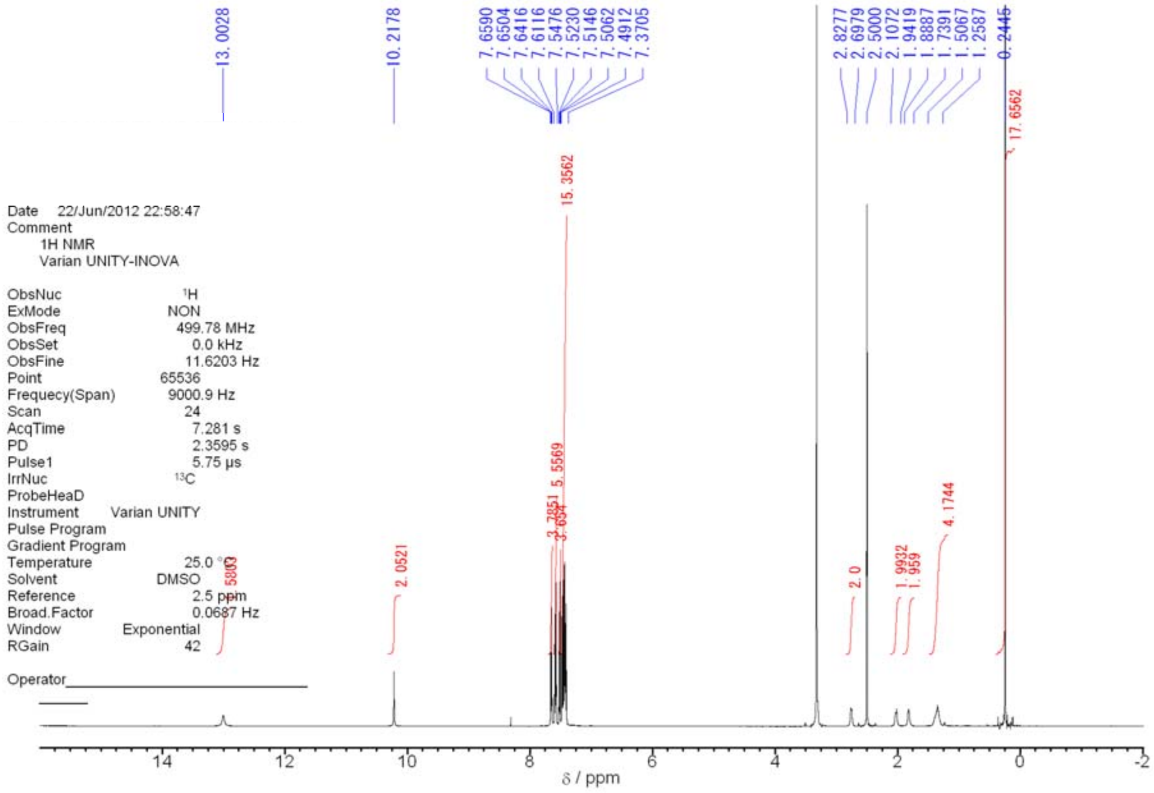

Supplementary Figure 82 | ^{1}H NMR (DMSO-d_6, 500 MHz) spectrum of (*S*,*S*)-**1a'**.

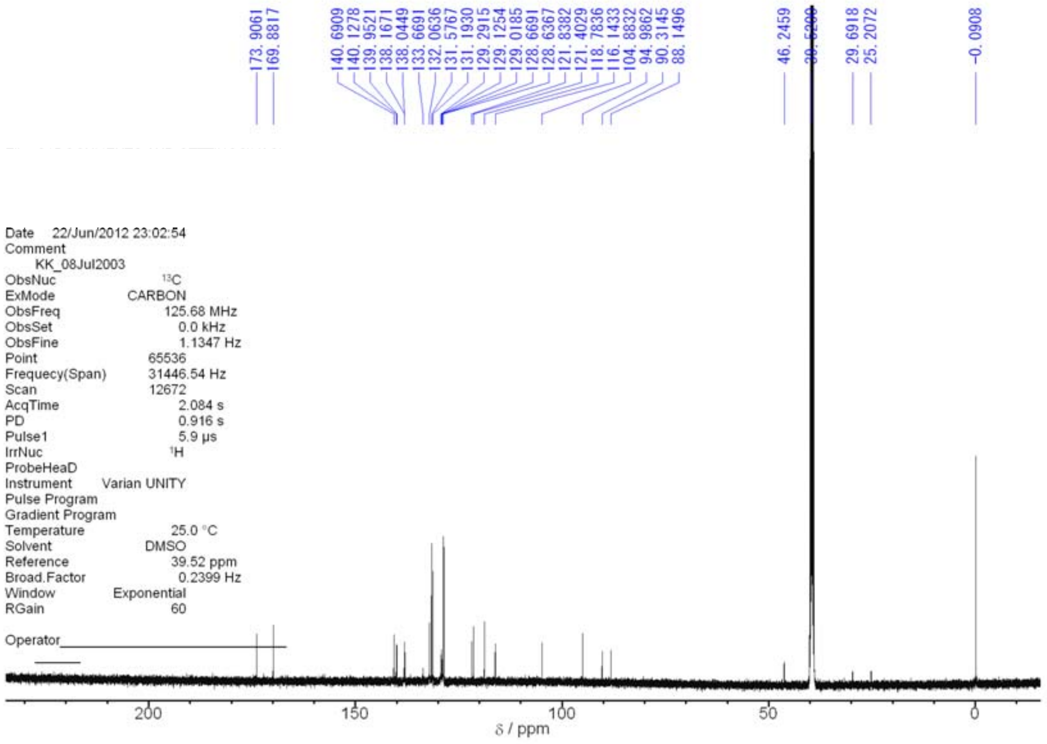

Supplementary Figure 83 | ^{13}C NMR (DMSO-d_6, 125 MHz) spectrum of (*S*,*S*)-**1a'**.

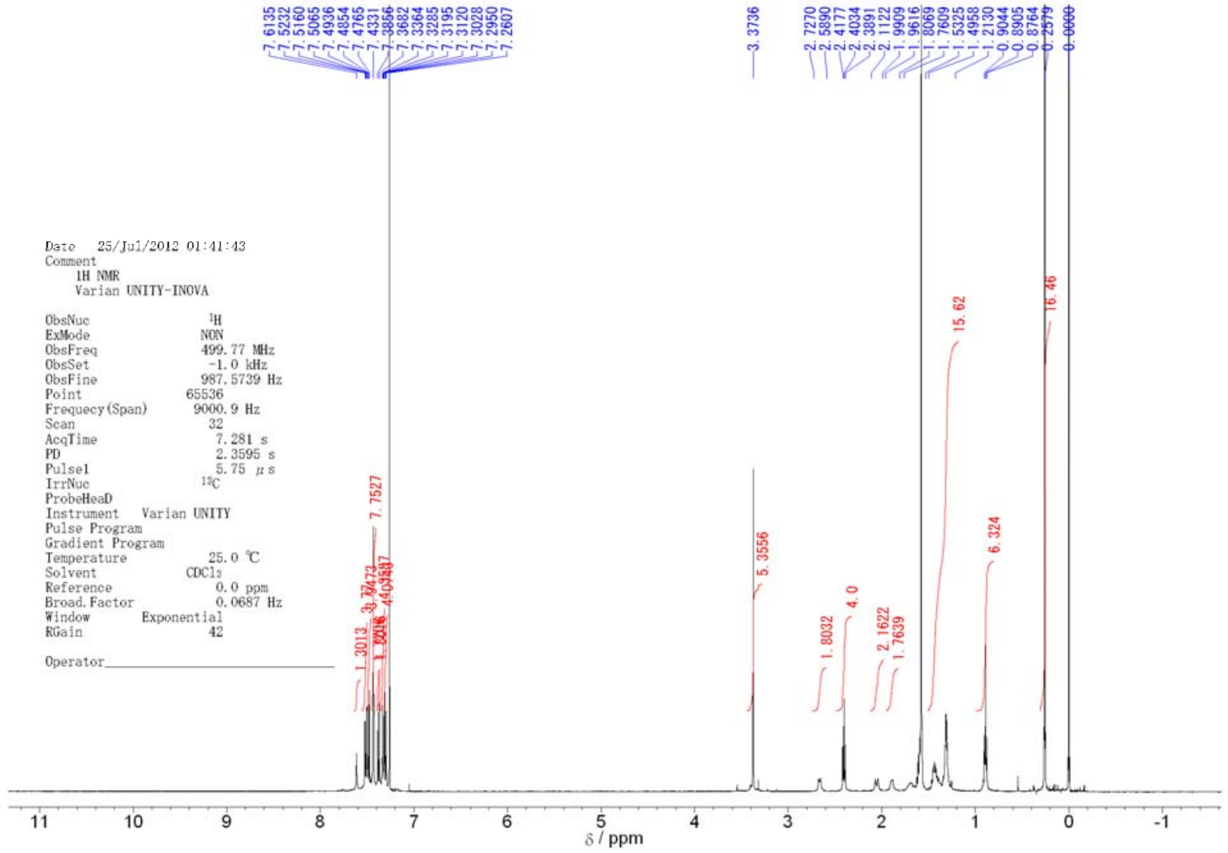

Supplementary Figure 84 | ^{1}H NMR ($CDCl_3$, 500 MHz) spectrum of (*R*,*R*)-**1b-OMe**.

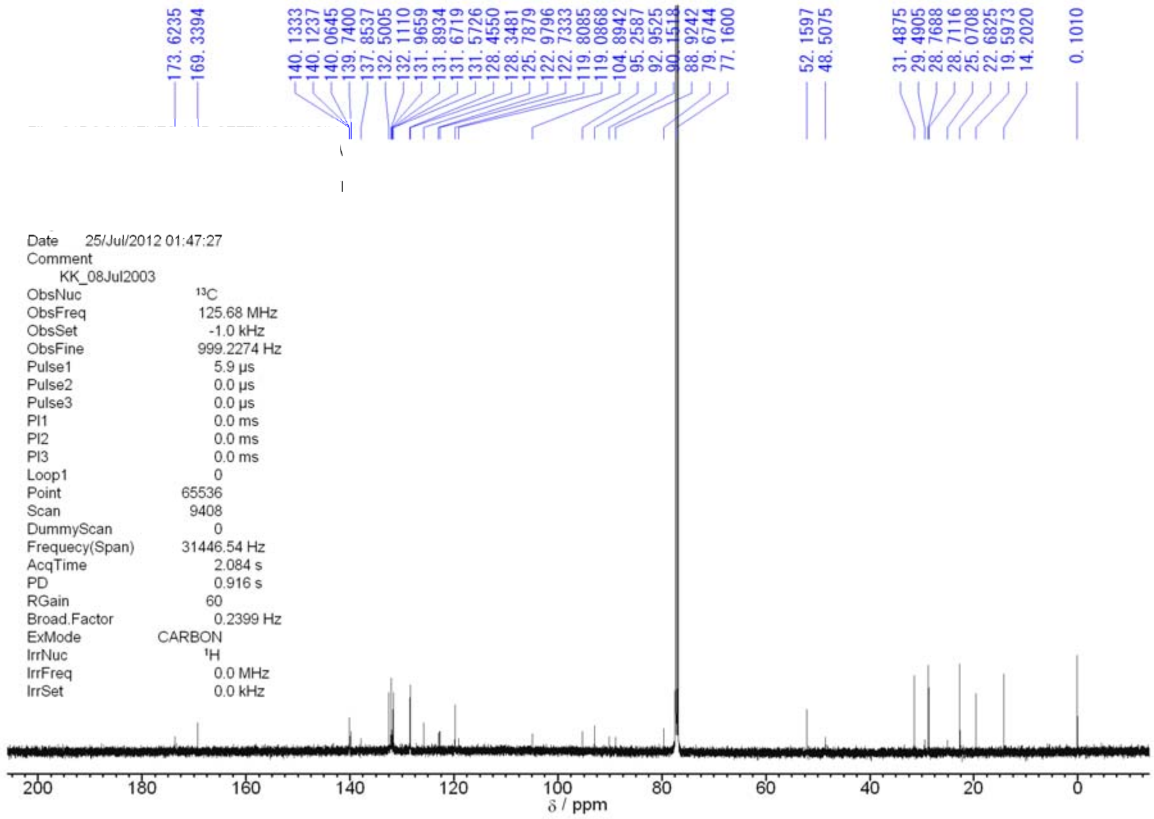

Supplementary Figure 85 | ^{13}C NMR ($CDCl_3$, 125 MHz) spectrum of (*R*,*R*)-**1b-OMe**.

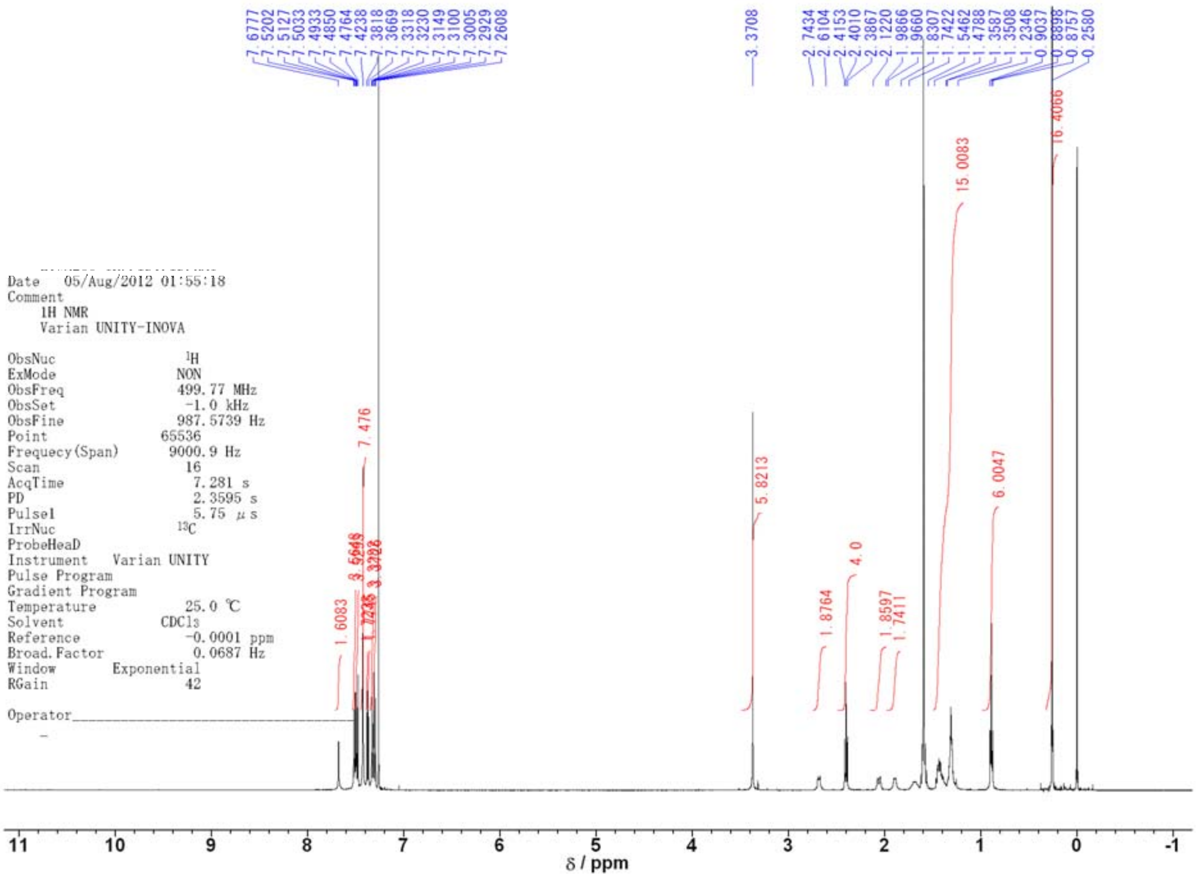

Supplementary Figure 86 | ^{1}H NMR ($CDCl_3$, 500 MHz) spectrum of (*S*,*S*)-**1b-OMe**.

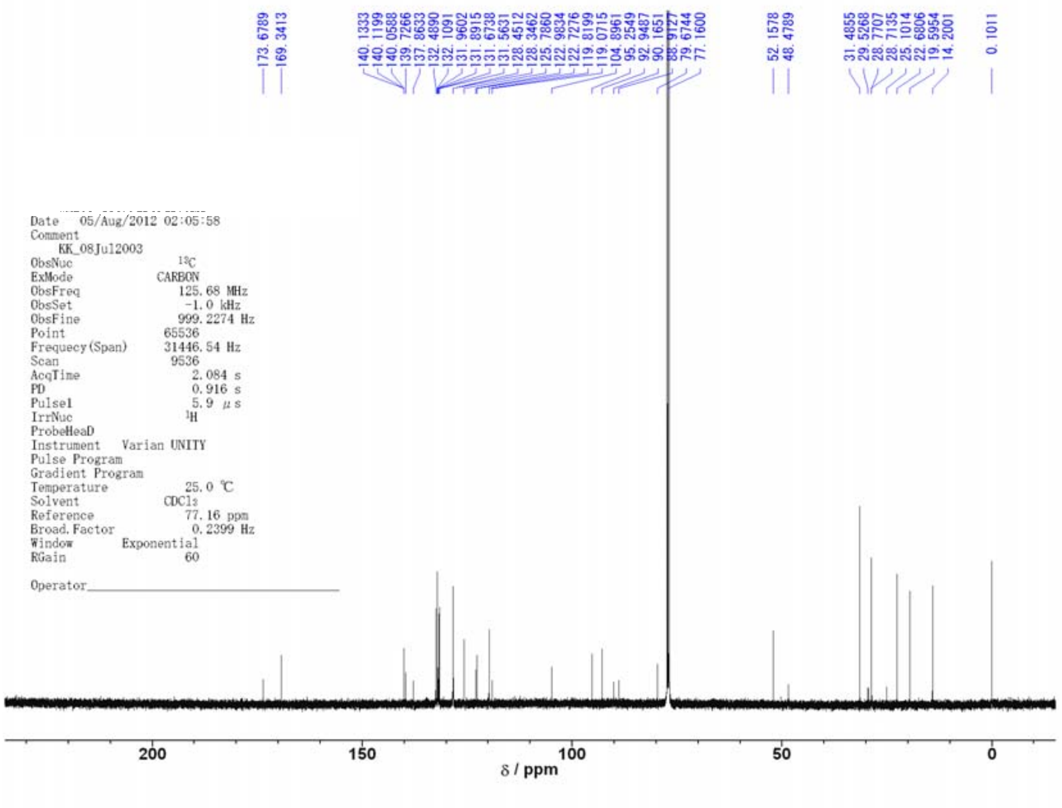

Supplementary Figure 87 | ^{13}C NMR ($CDCl_3$, 125 MHz) spectrum of (*S*,*S*)-**1b-OMe**.

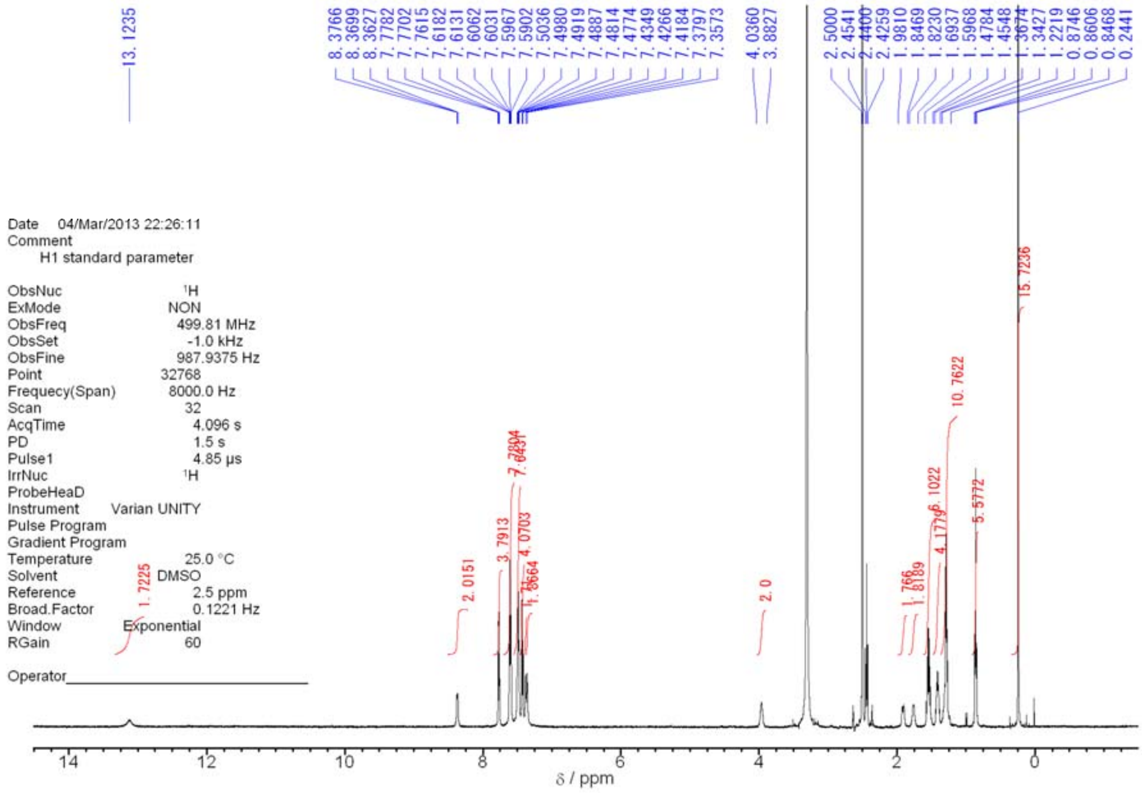

Supplementary Figure 88 | ^{1}H NMR (DMSO-d_6, 500 MHz) spectrum of (*R*,*R*)-**2a**.

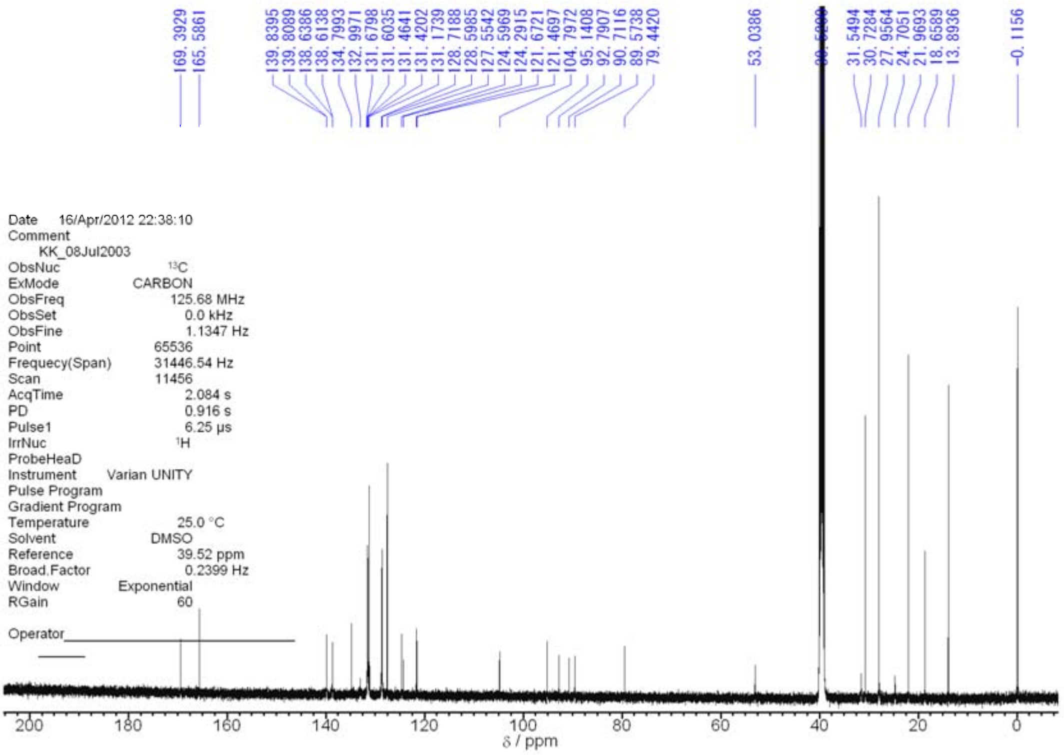

Supplementary Figure 89 | ^{13}C NMR (DMSO-d_6, 125 MHz) spectrum of (*R*,*R*)-**2a**.

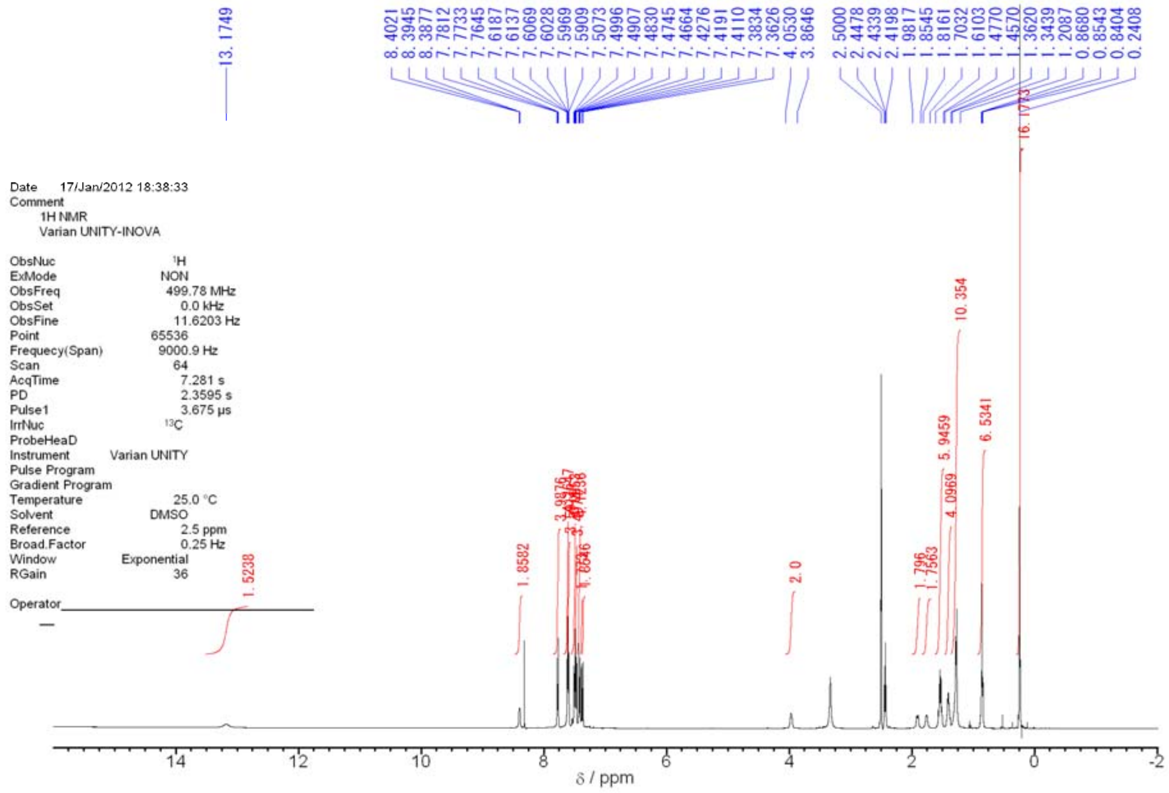

Supplementary Figure 90 | ^{1}H NMR (DMSO-d_6, 500 MHz) spectrum of (*S*,*S*)-**2a**.

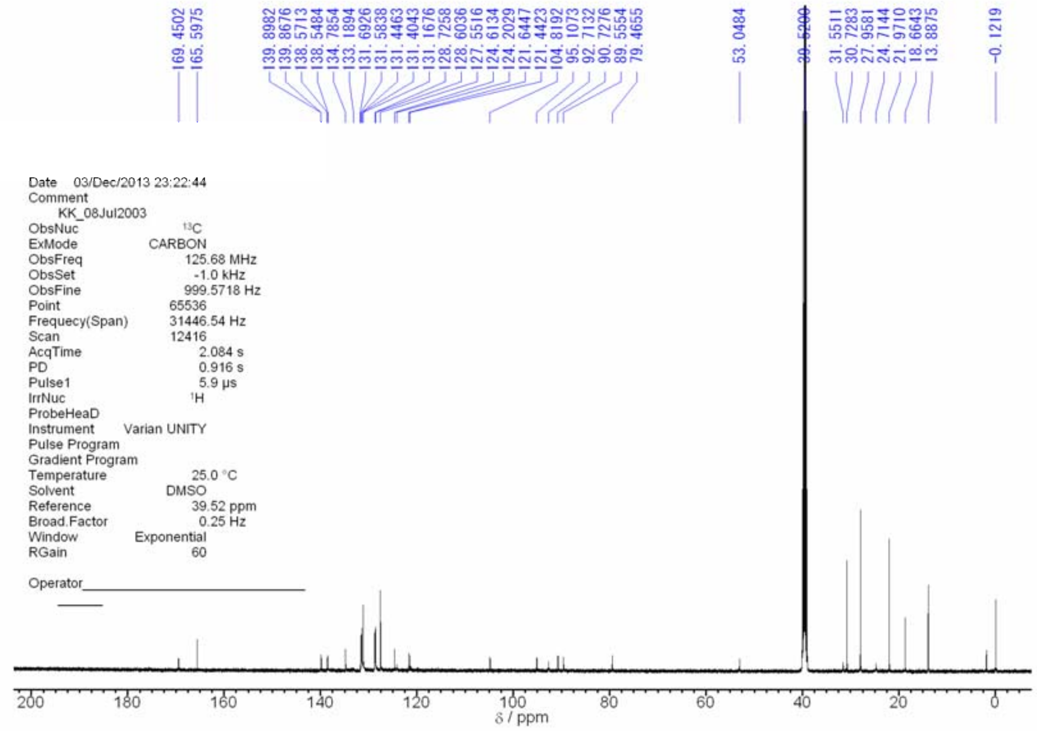

Supplementary Figure 91 | ^{13}C NMR (DMSO-d_6, 125 MHz) spectrum of (*S*,*S*)-**2a**.

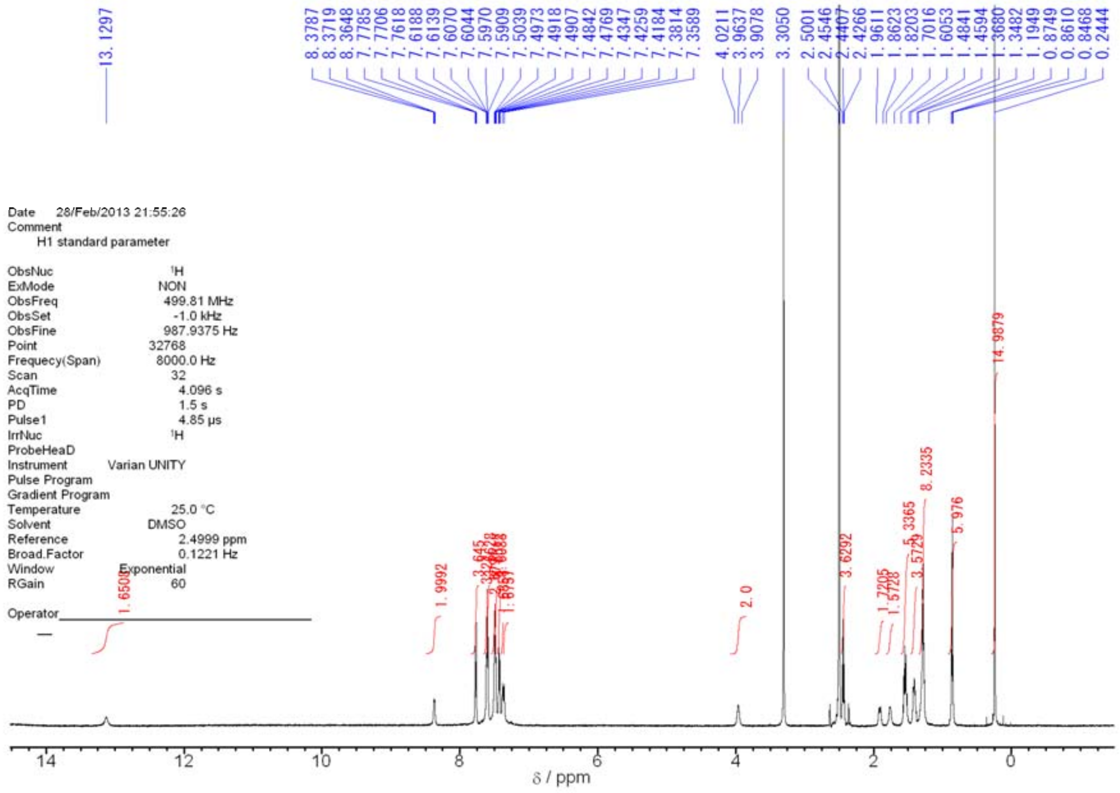

Supplementary Figure 92 | ¹H NMR (DMSO-d_6, 500 MHz) spectrum of *rac*-**2a**.

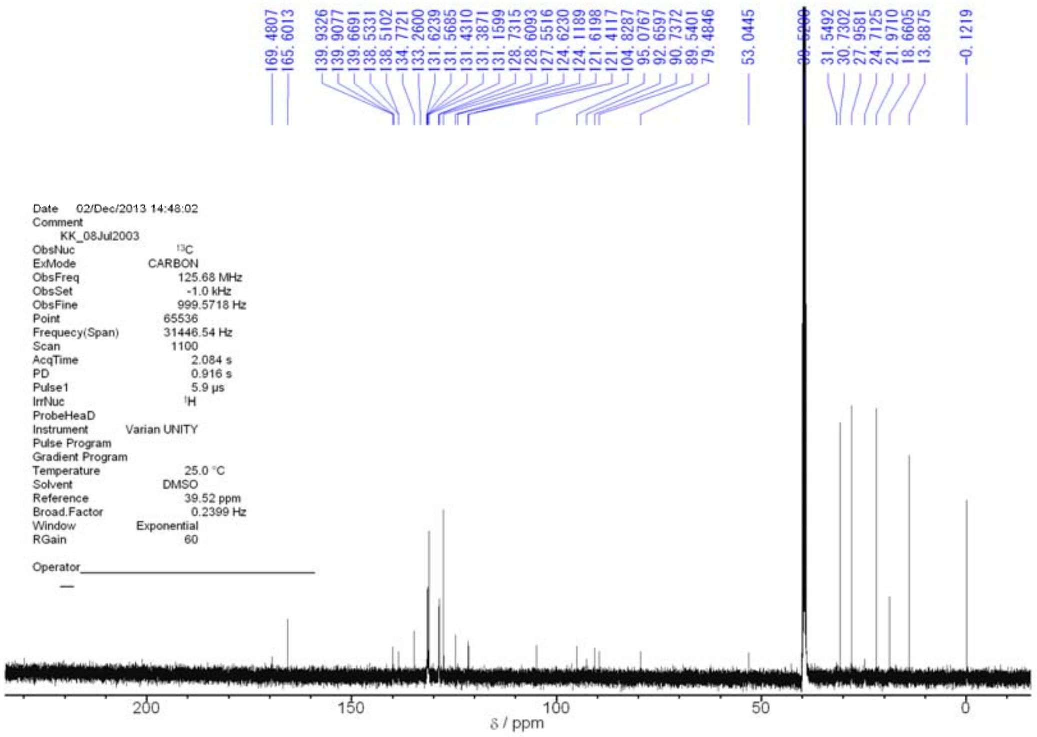

Supplementary Figure 93 | ¹³C NMR (DMSO-d_6, 125 MHz) spectrum of *rac*-**2a**.

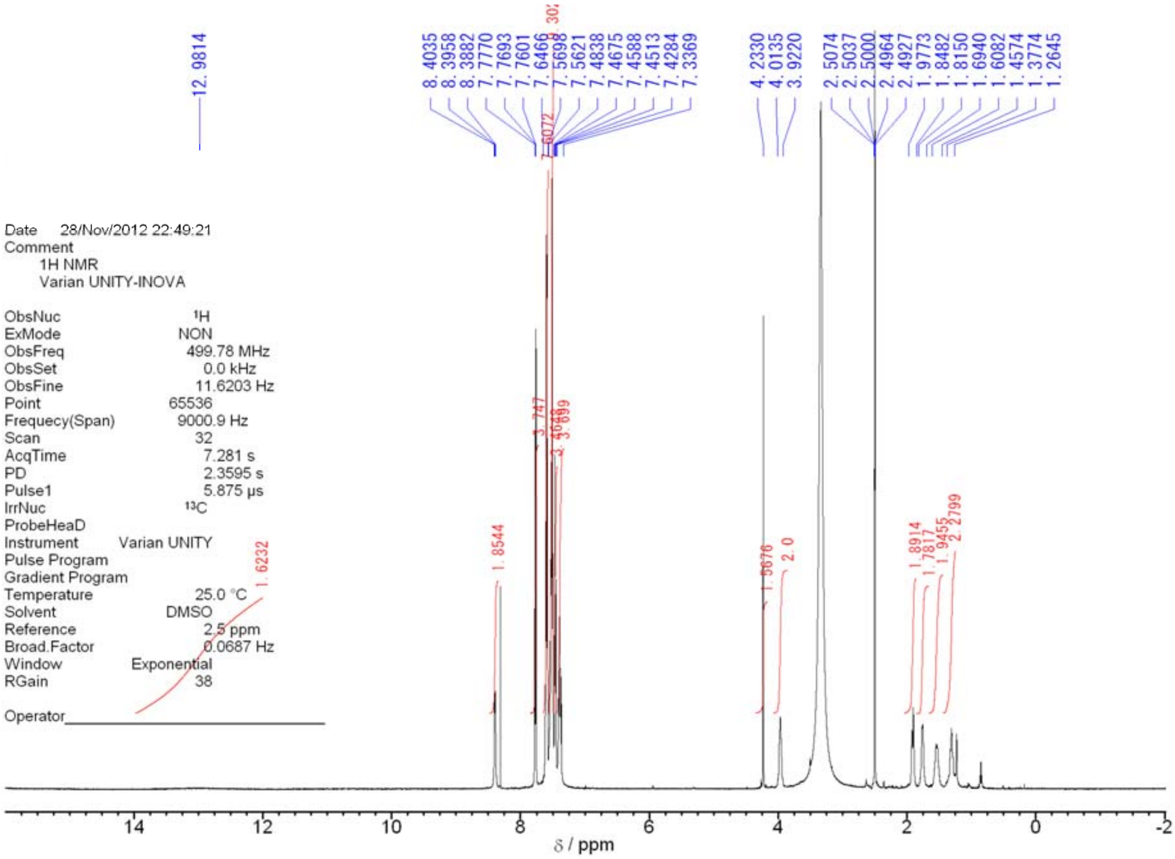

Supplementary Figure 94 | ^{1}H NMR (DMSO-d_6, 500 MHz) spectrum of (*S*,*S*)-**2a'**.

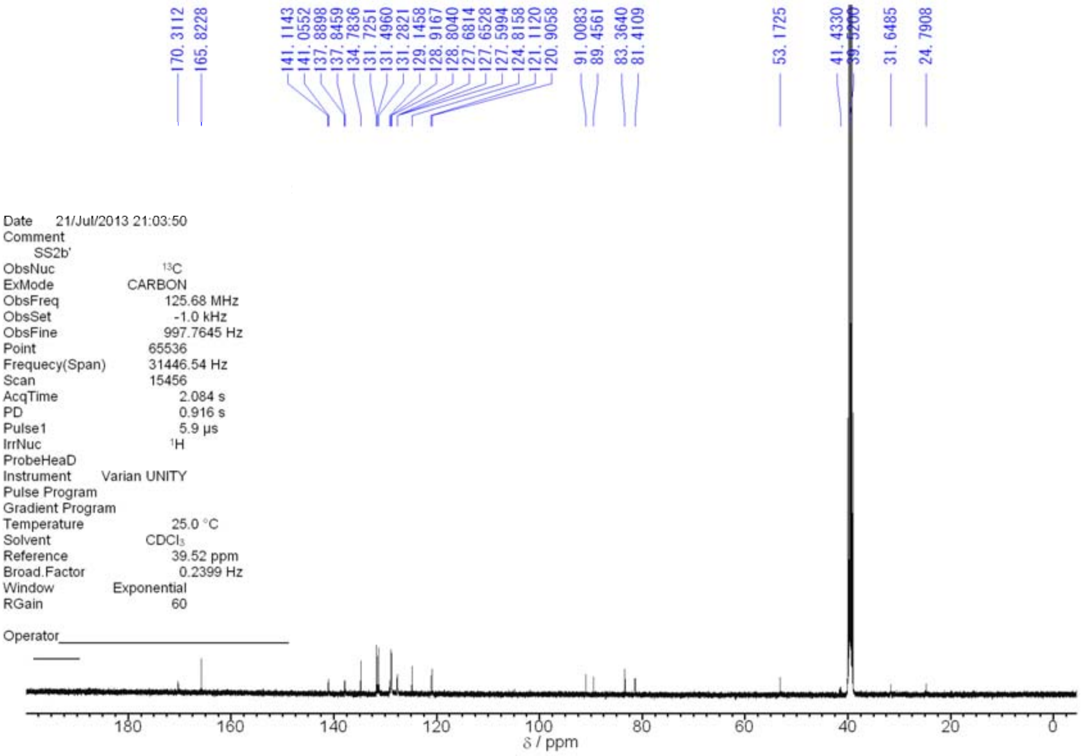

Supplementary Figure 95 | ^{13}C NMR (DMSO-d_6, 125 MHz) spectrum of (*S*,*S*)-**2a'**.

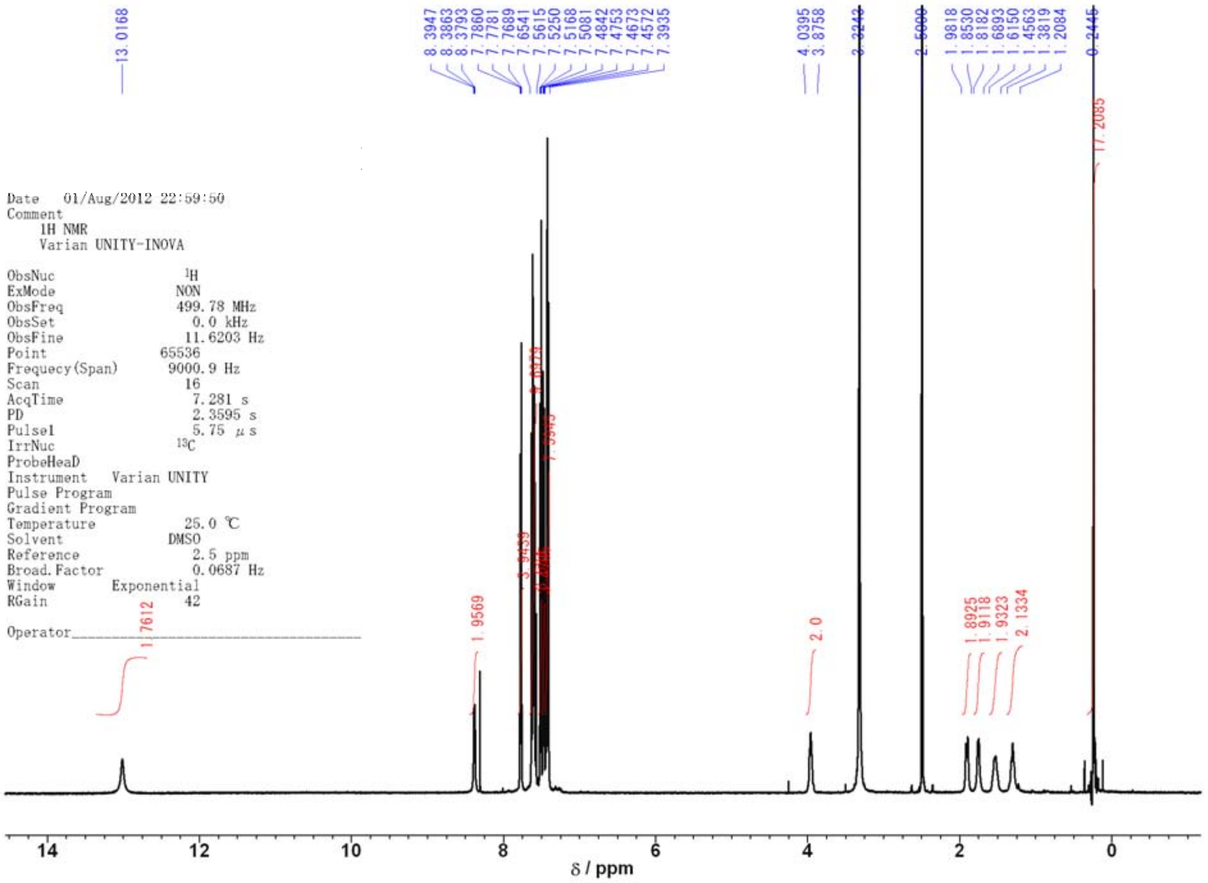

Supplementary Figure 96 | ^{1}H NMR (DMSO-d_6, 500 MHz) spectrum of (*S*,*S*)-**2a'-TMS**.

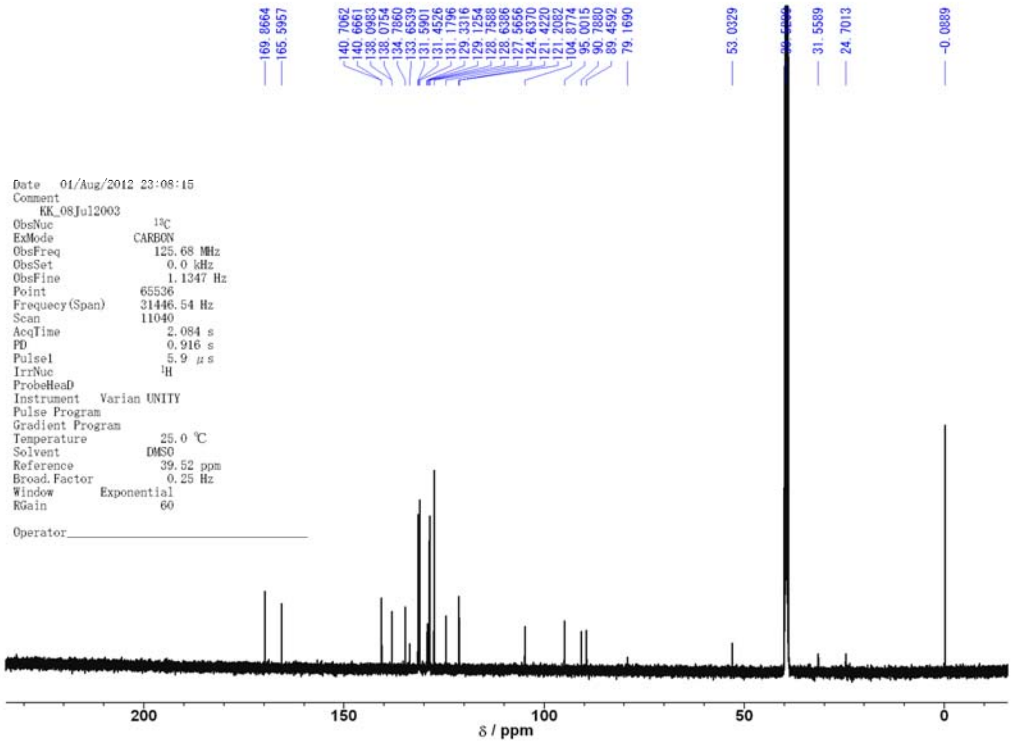

Supplementary Figure 97 | ^{13}C NMR (DMSO-d_6, 125 MHz) spectrum of (*S*,*S*)-**2a'-TMS**.

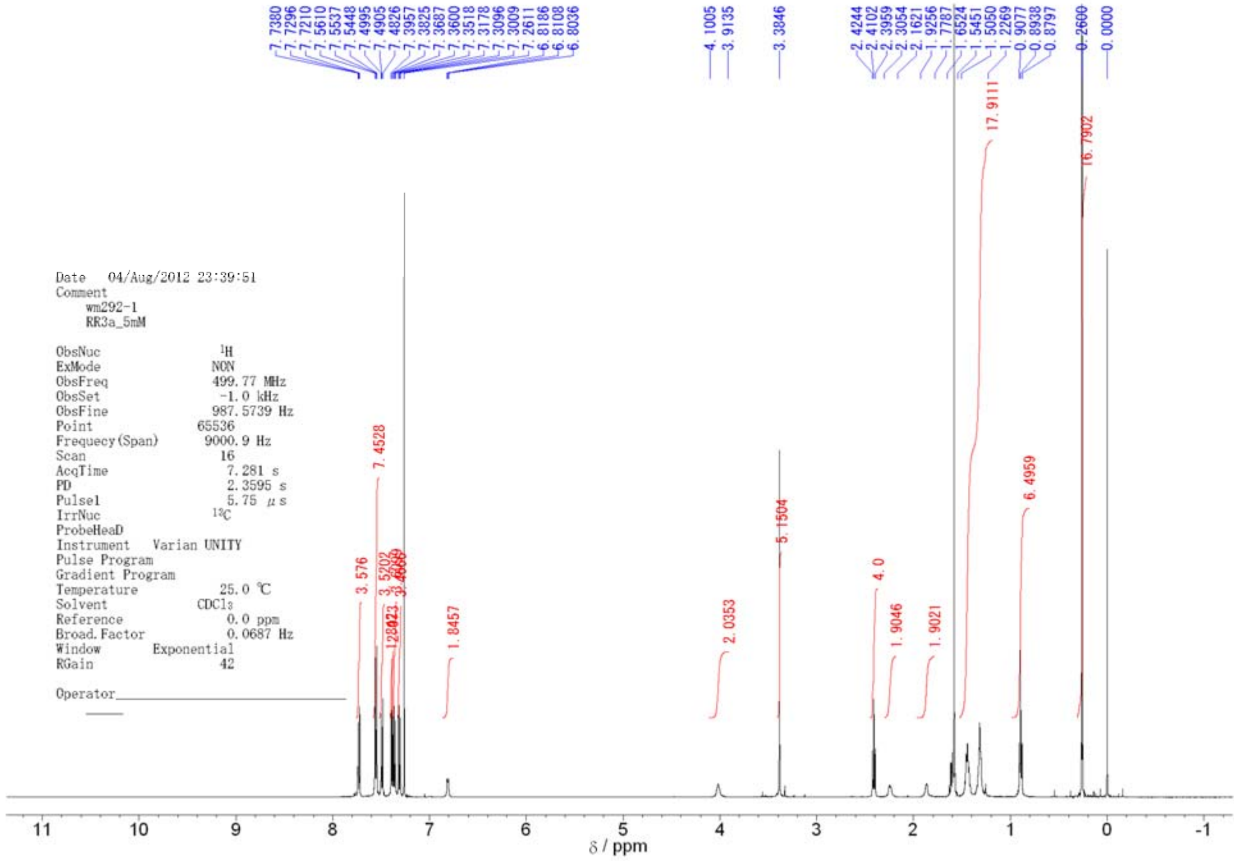

Supplementary Figure 98 | ^{1}H NMR ($CDCl_3$, 500 MHz) spectrum of (*R*,*R*)-**2b-OMe**.

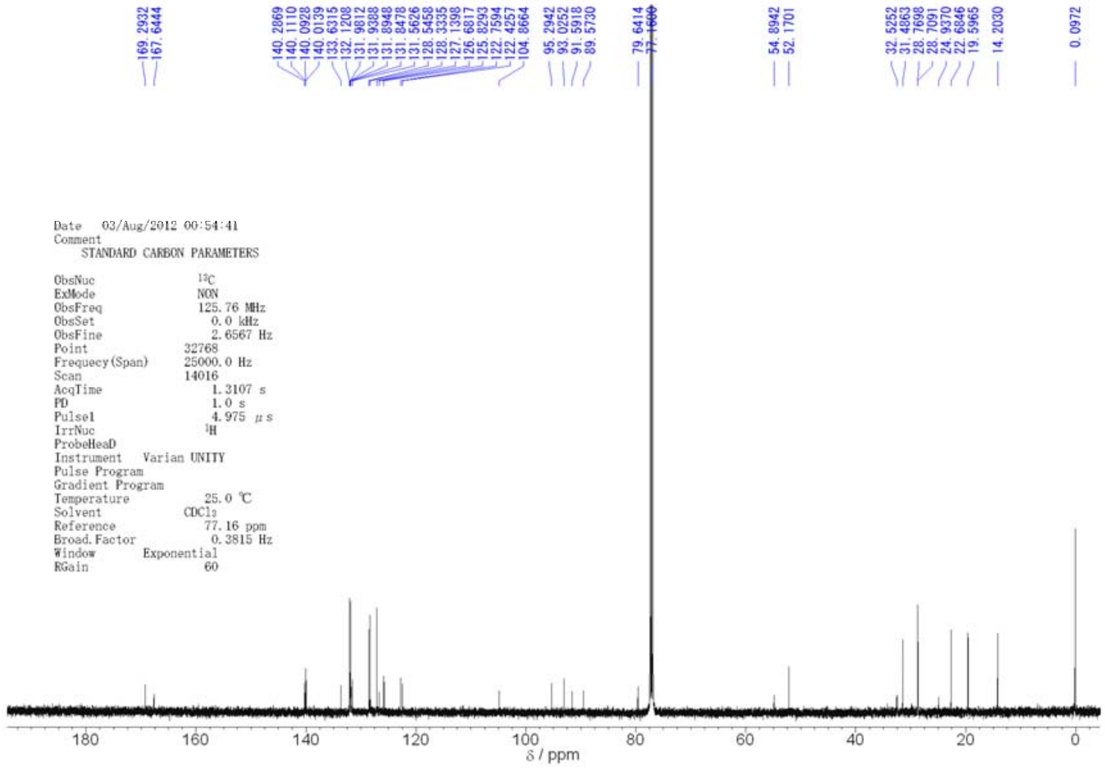

Supplementary Figure 99 | ^{13}C NMR ($CDCl_3$, 125 MHz) spectrum of (*R*,*R*)-**2b-OMe**.

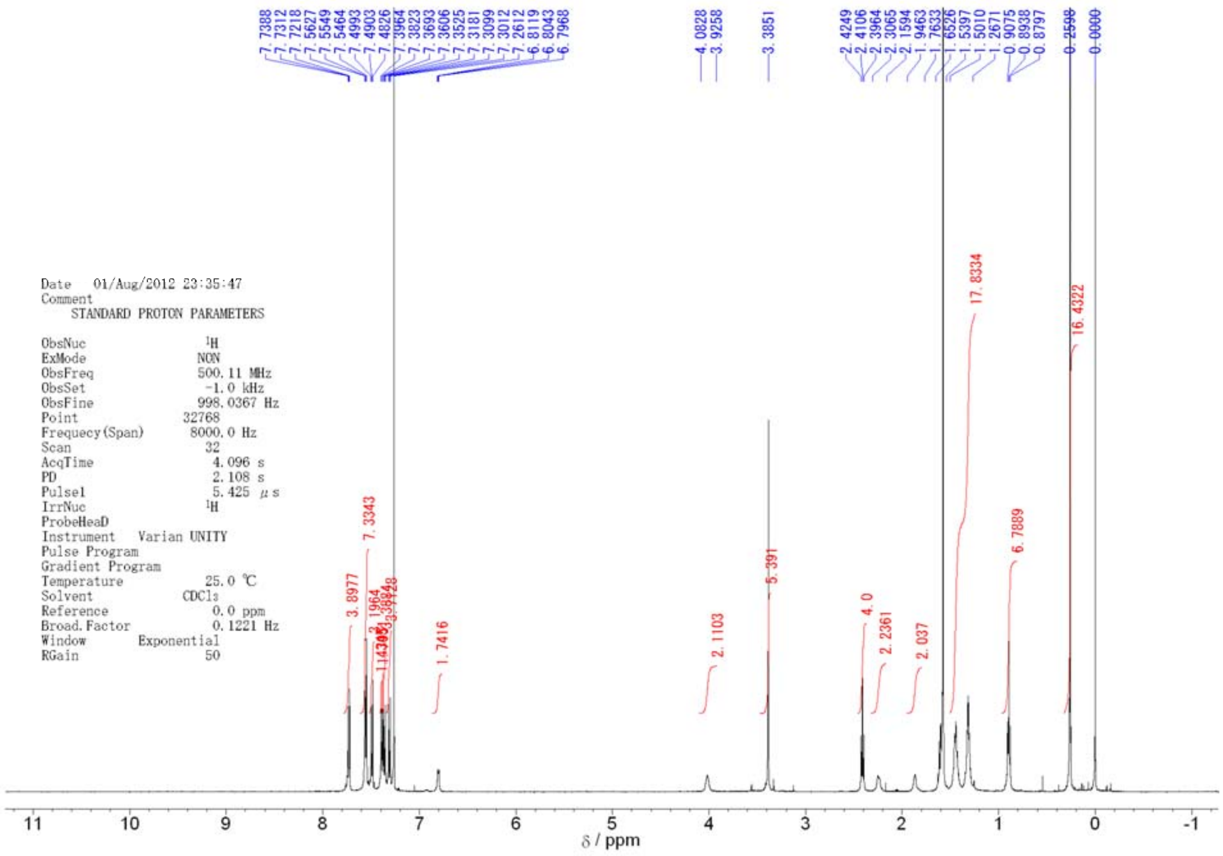

Supplementary Figure 100 | ^{1}H NMR ($CDCl_3$, 500 MHz) spectrum of (*S*,*S*)-**2b-OMe**.

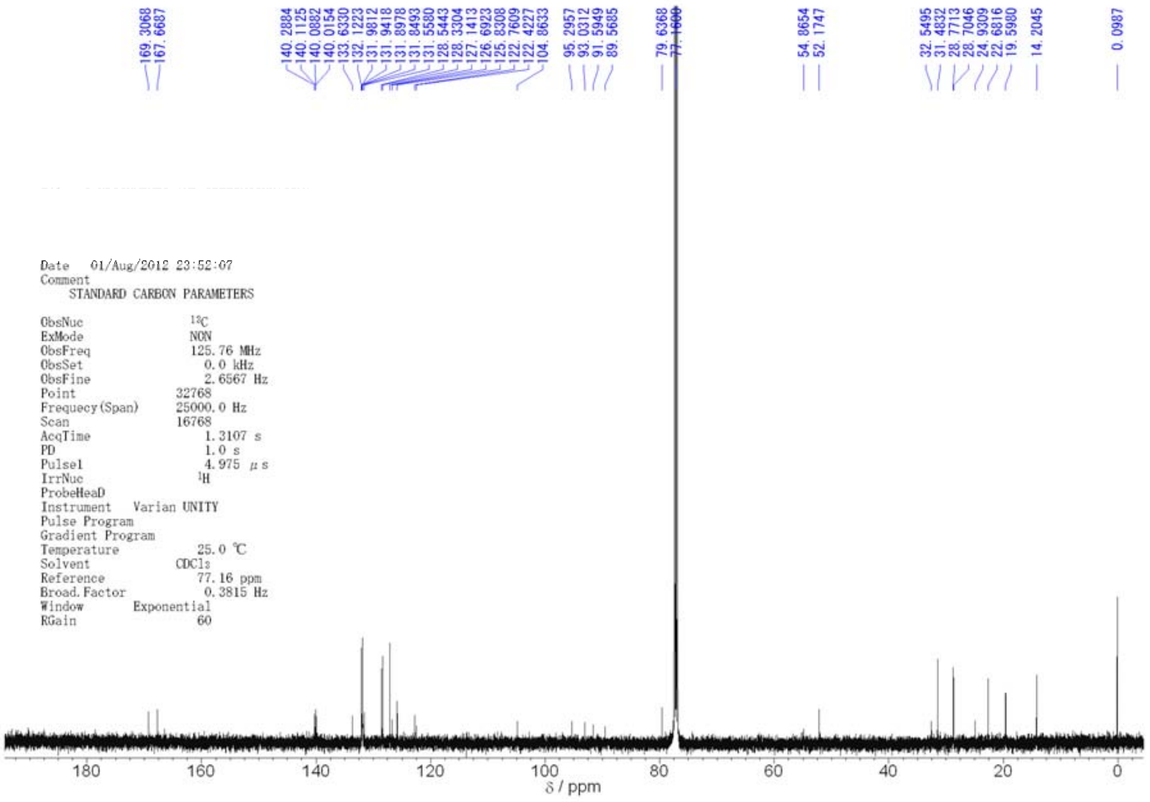

Supplementary Figure 101 | ^{13}C NMR ($CDCl_3$, 125 MHz) spectrum of (*S*,*S*)-**2b-OMe**.

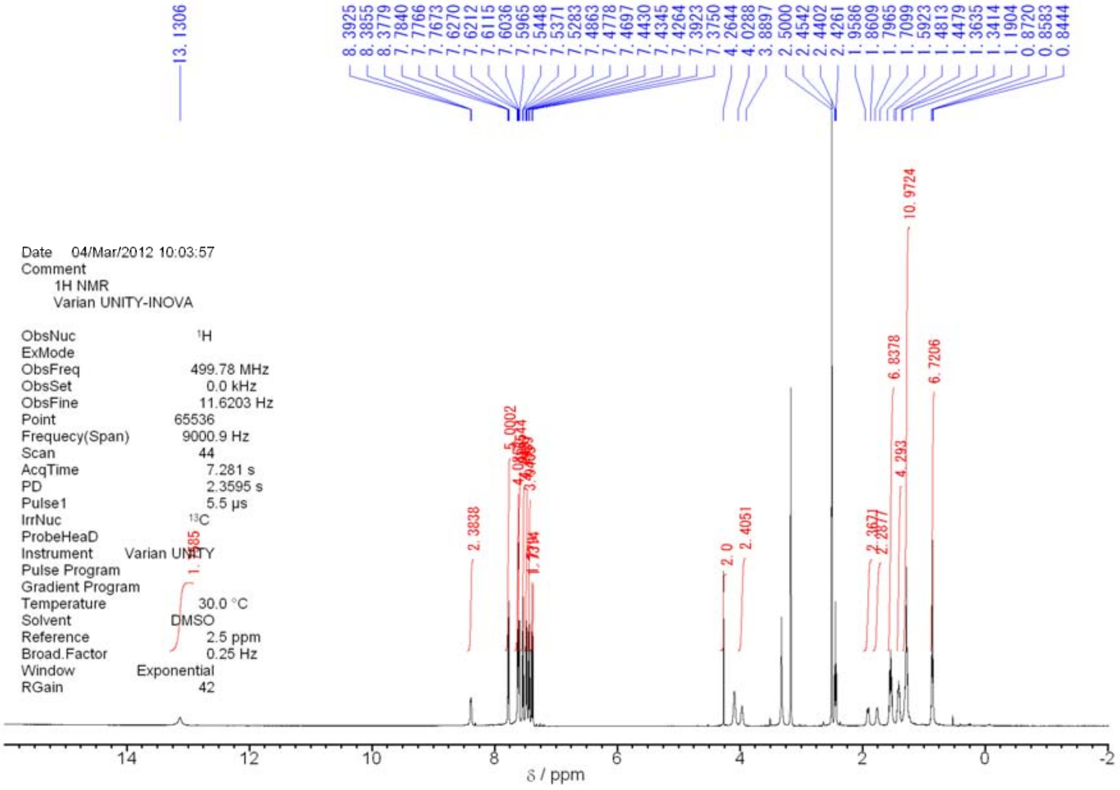

Supplementary Figure 102 | ^{1}H NMR (DMSO-d_6, 500 MHz) spectrum of (*S*,*S*)-**2c**.

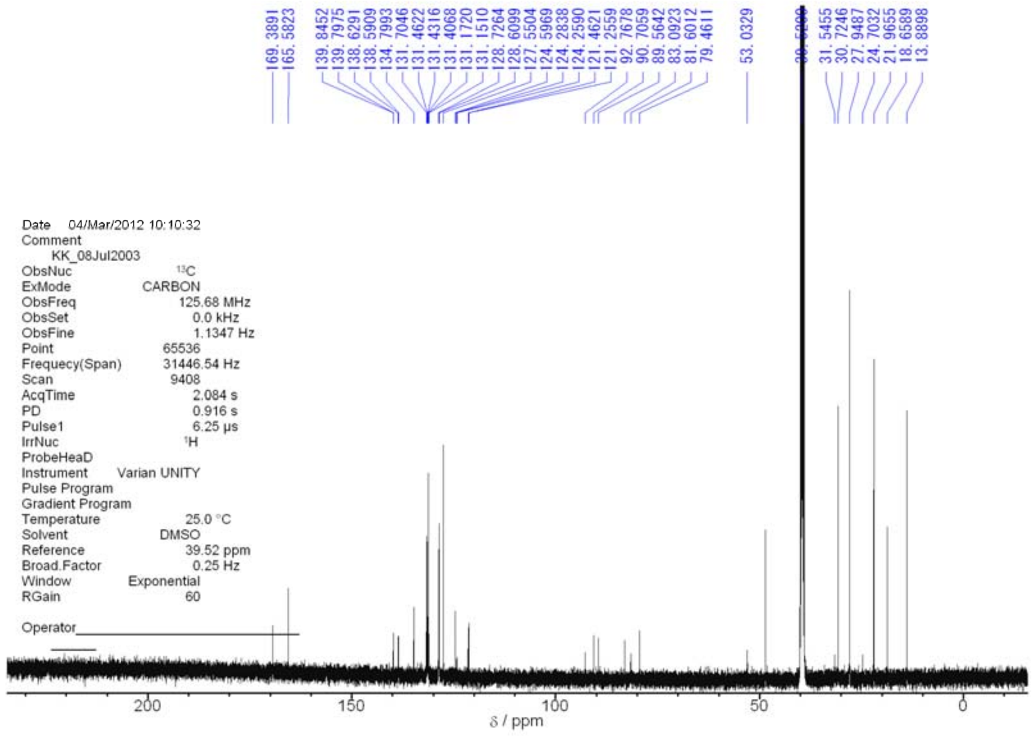

Supplementary Figure 103 | ^{13}C NMR (DMSO-d_6, 125 MHz) spectrum of (*S*,*S*)-**2c**.

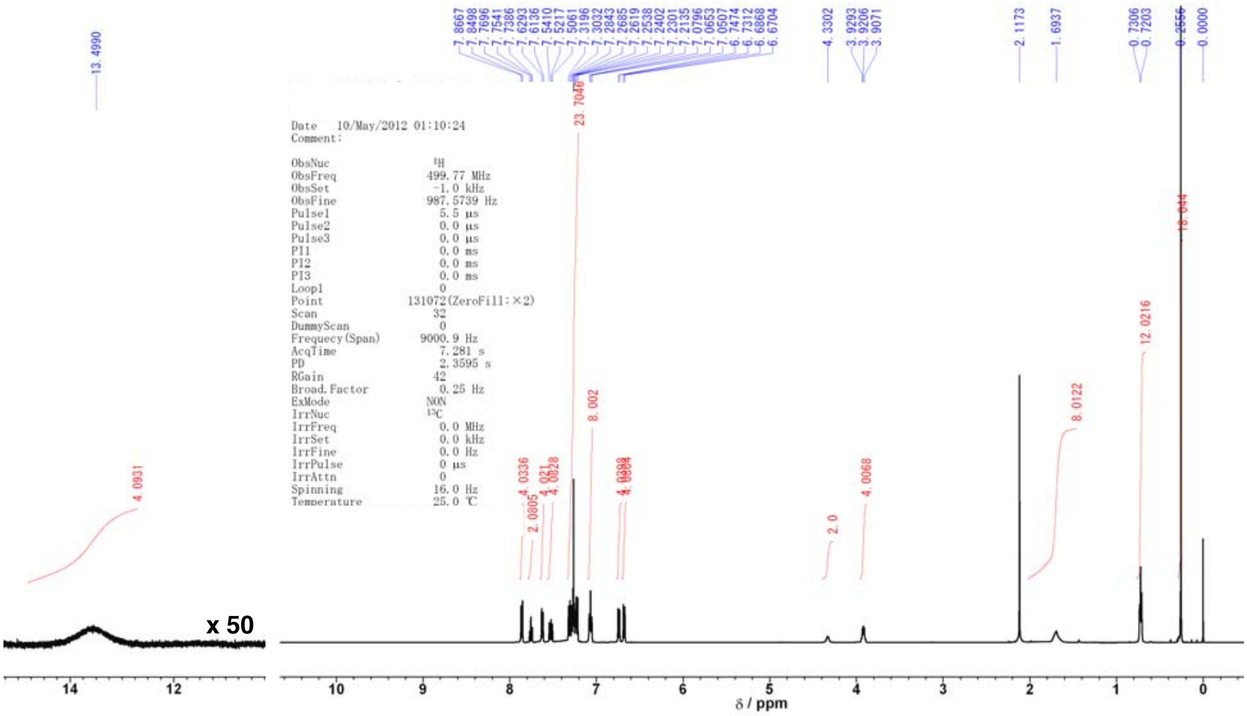

Supplementary Figure 104 | ^{1}H NMR ($CDCl_3$, 500 MHz) spectrum of (*R*,*R*,*meso*,*R*,*R*)-**3e**·$(CH_3CO_2H)_2$.

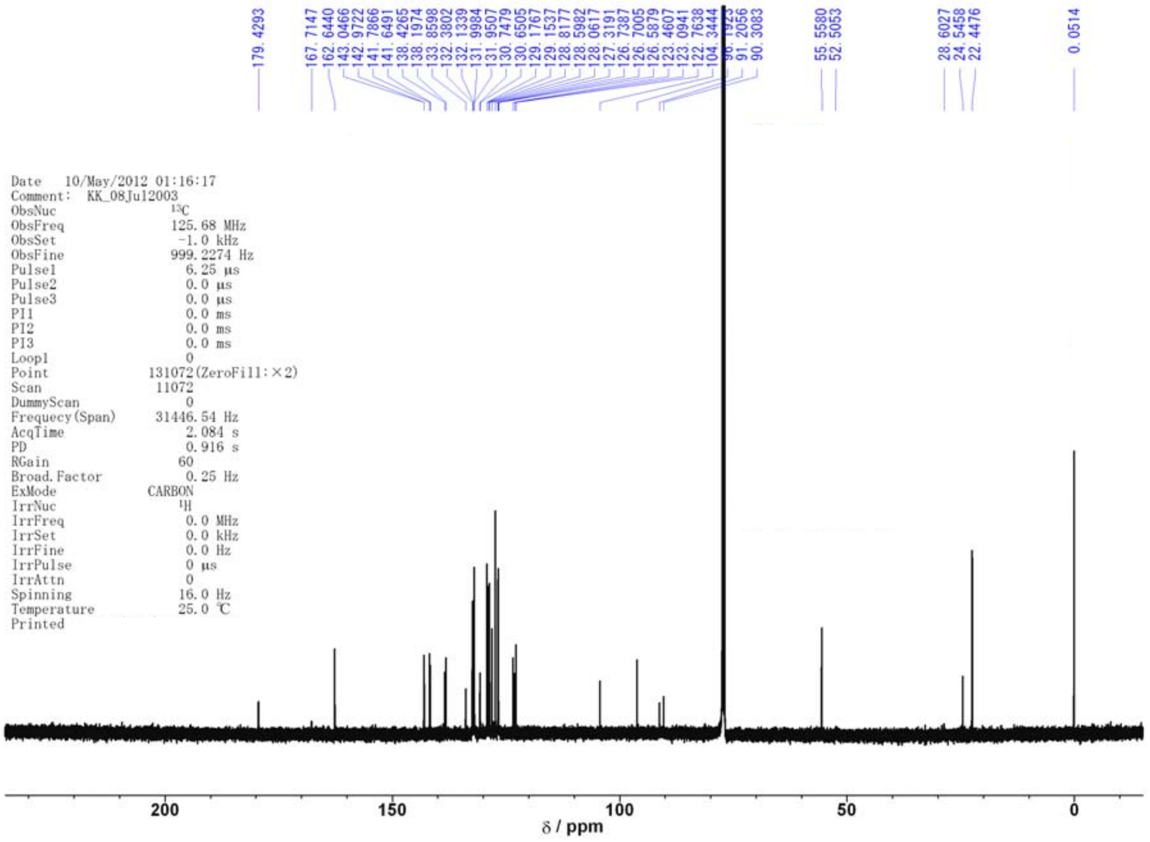

Supplementary Figure 105 | ^{13}C NMR ($CDCl_3$, 125 MHz) spectrum of (*R*,*R*,*meso*,*R*,*R*)-**3e**·$(CH_3CO_2H)_2$.

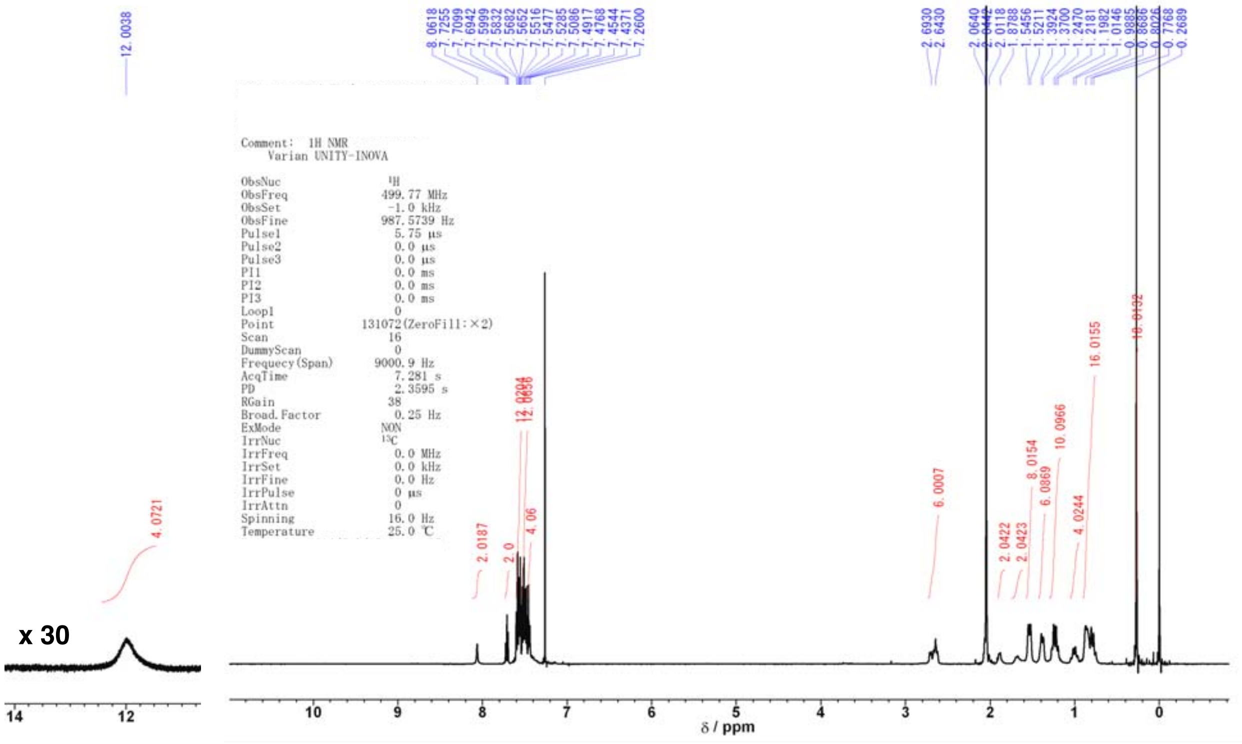

Supplementary Figure 106 | ^{1}H NMR ($CDCl_3$, 500 MHz) spectrum of (*R*,*R*)-**4c**·$(CH_3CO_2H)_2$.

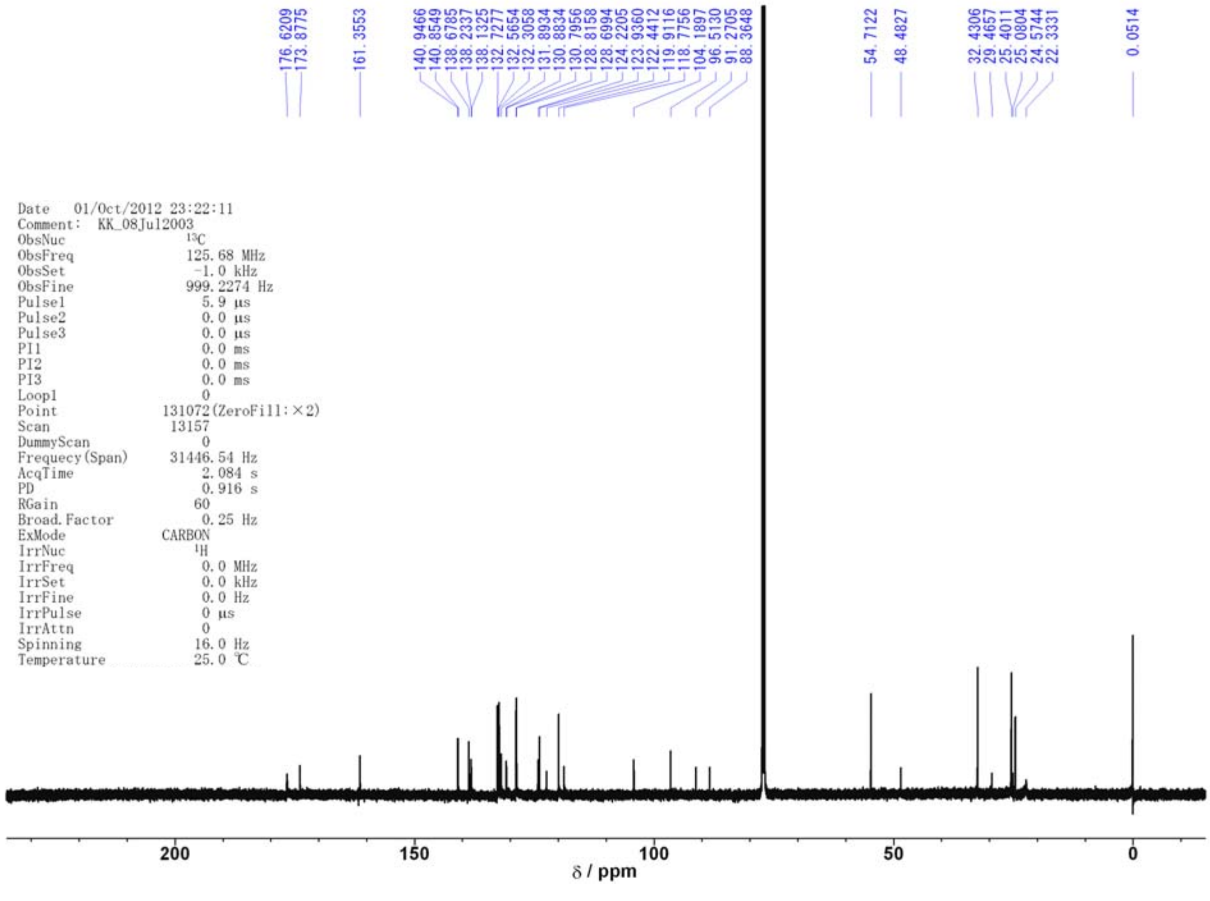

Supplementary Figure 107 | ^{13}C NMR ($CDCl_3$, 125 MHz) spectrum of (*R*,*R*)-**4c**·$(CH_3CO_2H)_2$.

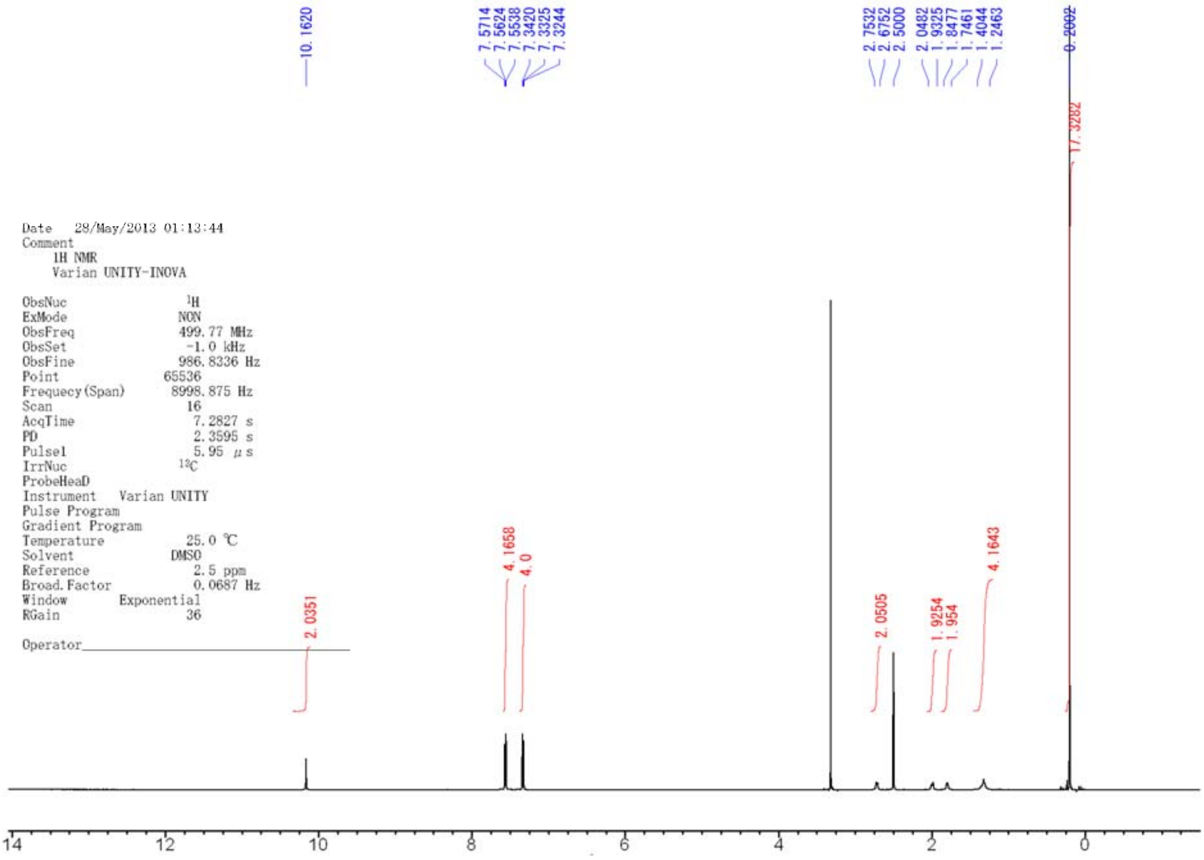

Supplementary Figure 108 | ^{1}H NMR (DMSO-d_6, 500 MHz) spectrum of (R,R)-**M1**.

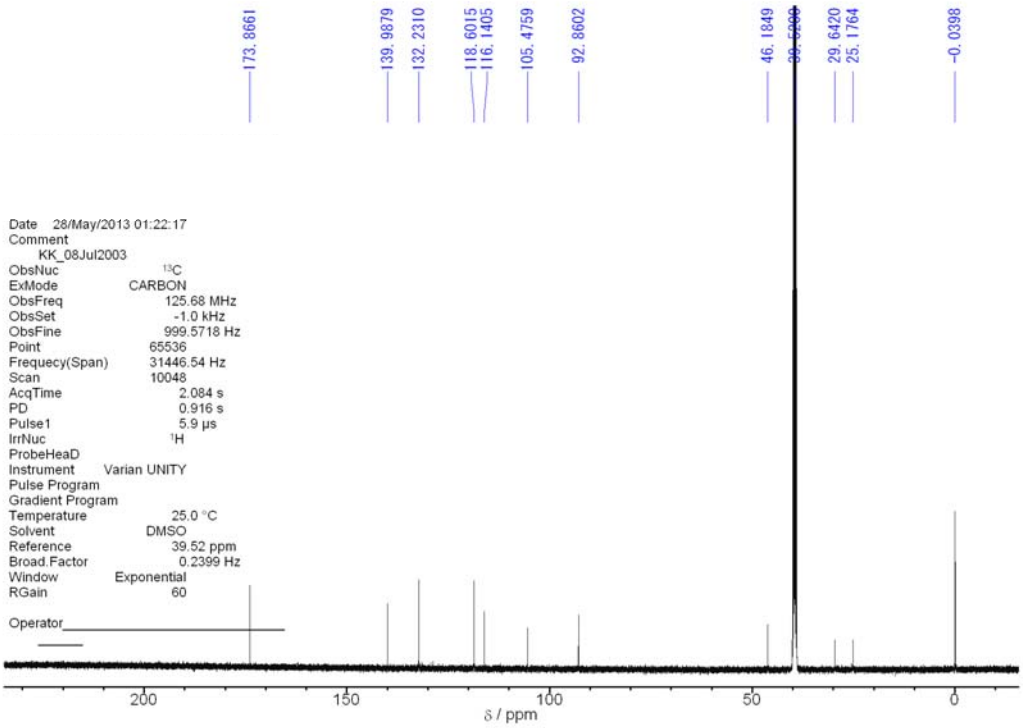

Supplementary Figure 109 | ^{13}C NMR (DMSO-d_6, 125 MHz) spectrum of (R,R)-**M1**.

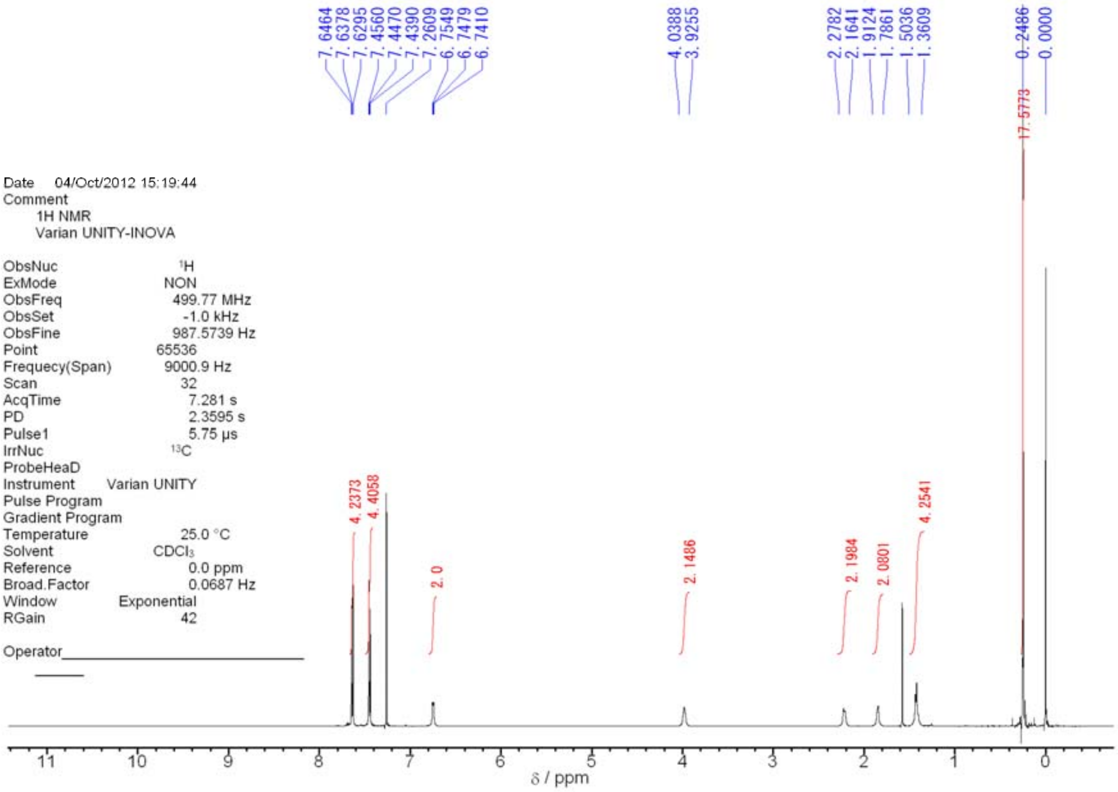

Supplementary Figure 110 | ^{1}H NMR ($CDCl_3$, 500 MHz) spectrum of (*R*,*R*)-**M2**.

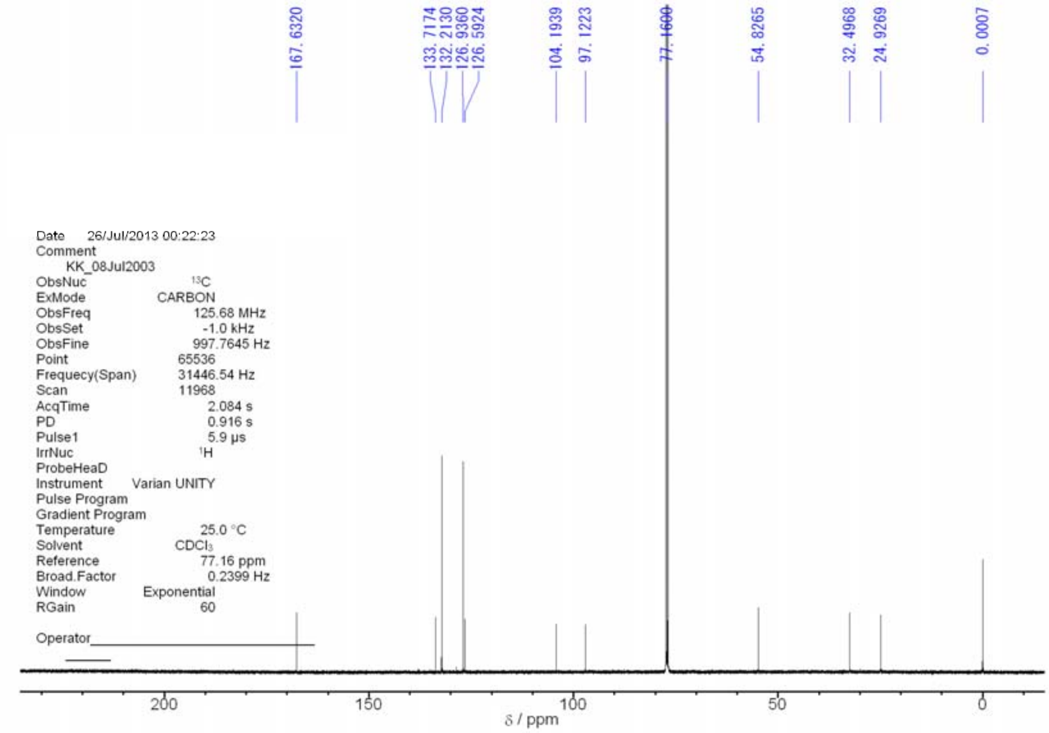

Supplementary Figure 111 | ^{13}C NMR ($CDCl_3$, 125 MHz) spectrum of (*R*,*R*)-**M2**.

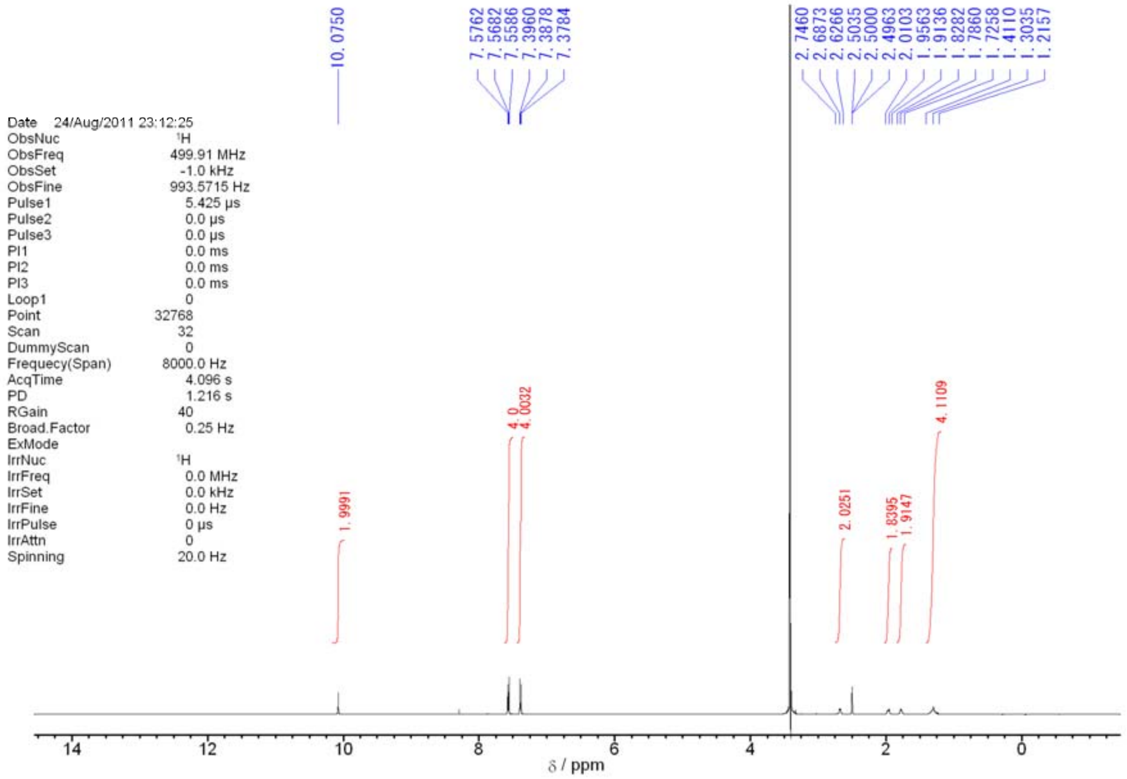

Supplementary Figure 112 | ^{1}H NMR (DMSO-d_6, 500 MHz) spectrum of *rac*-**7**.

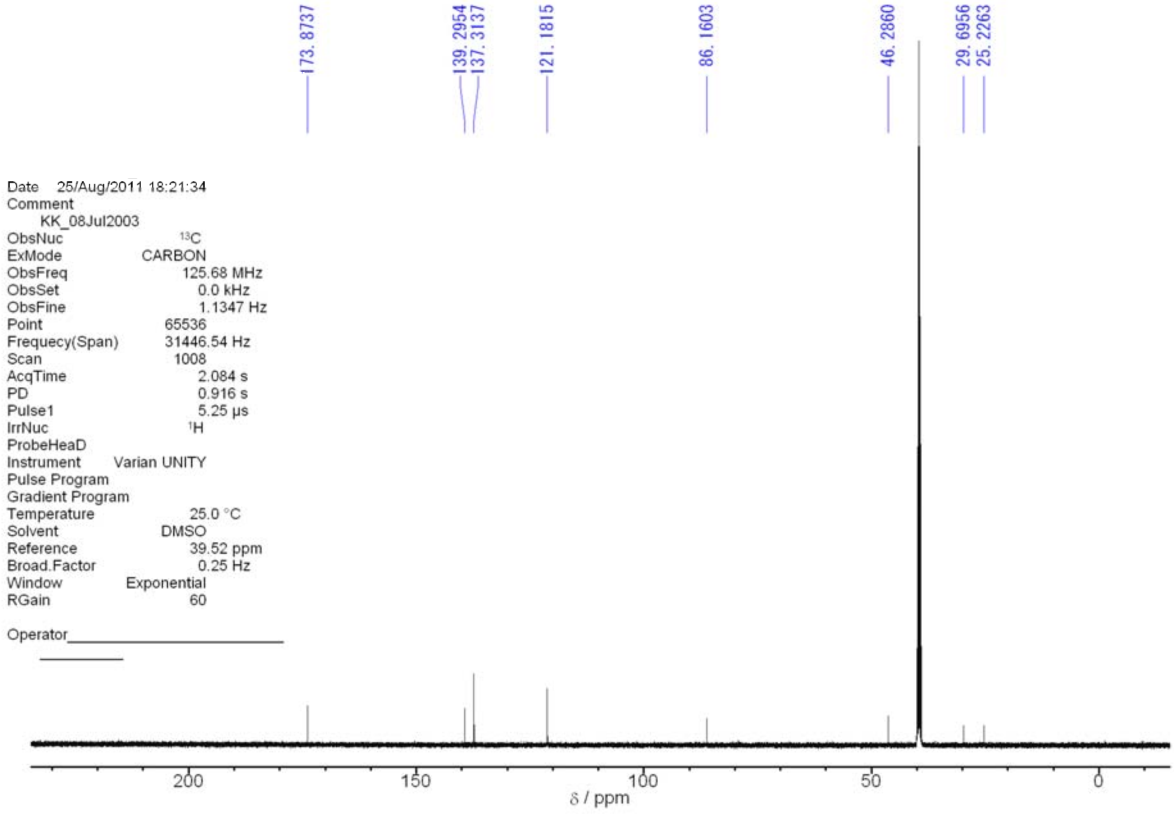

Supplementary Figure 113 | ^{13}C NMR (DMSO-d_6, 125 MHz) spectrum of *rac*-**7**.

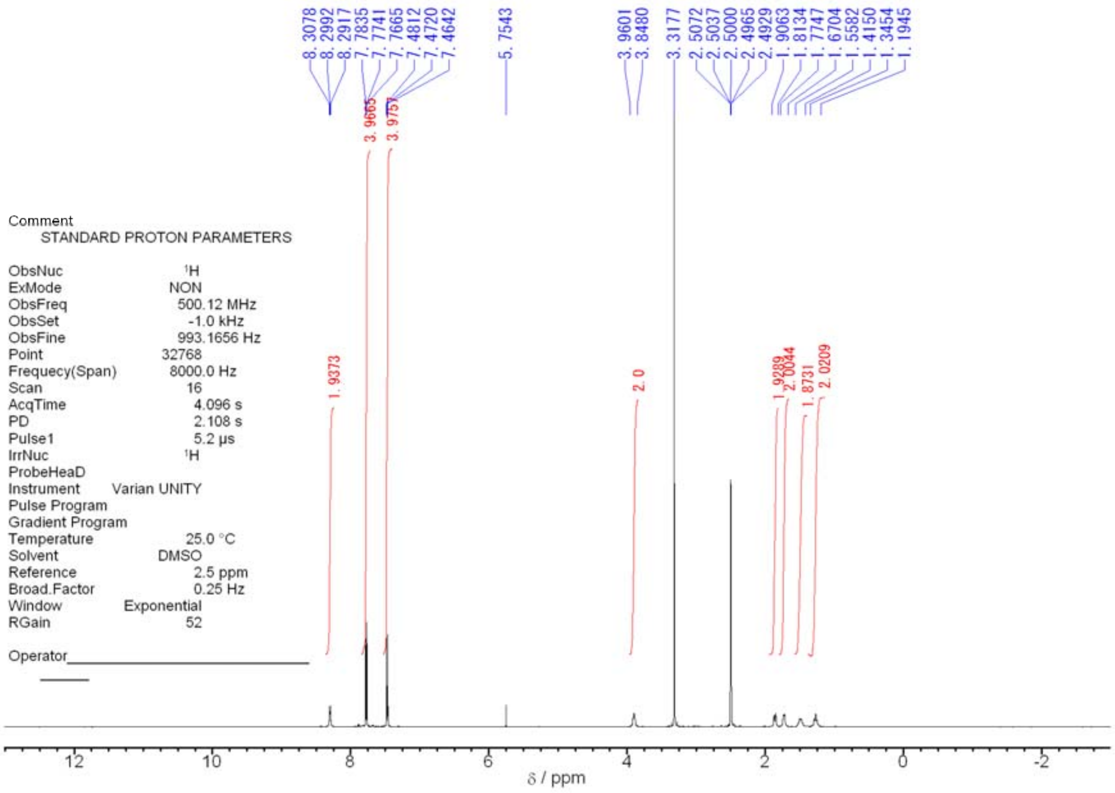

Supplementary Figure 114 | ^{1}H NMR (DMSO-d_6, 500 MHz) spectrum of *rac*-**8**.

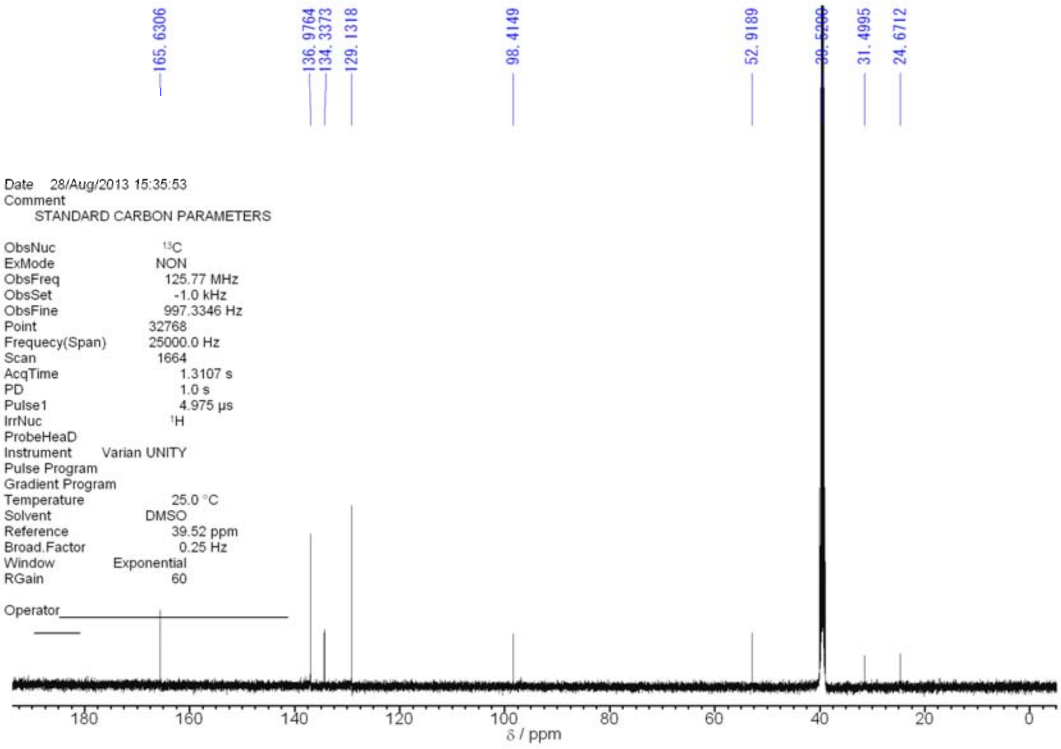

Supplementary Figure 115 | ^{13}C NMR (DMSO-d_6, 125 MHz) spectrum of *rac*-**8**.

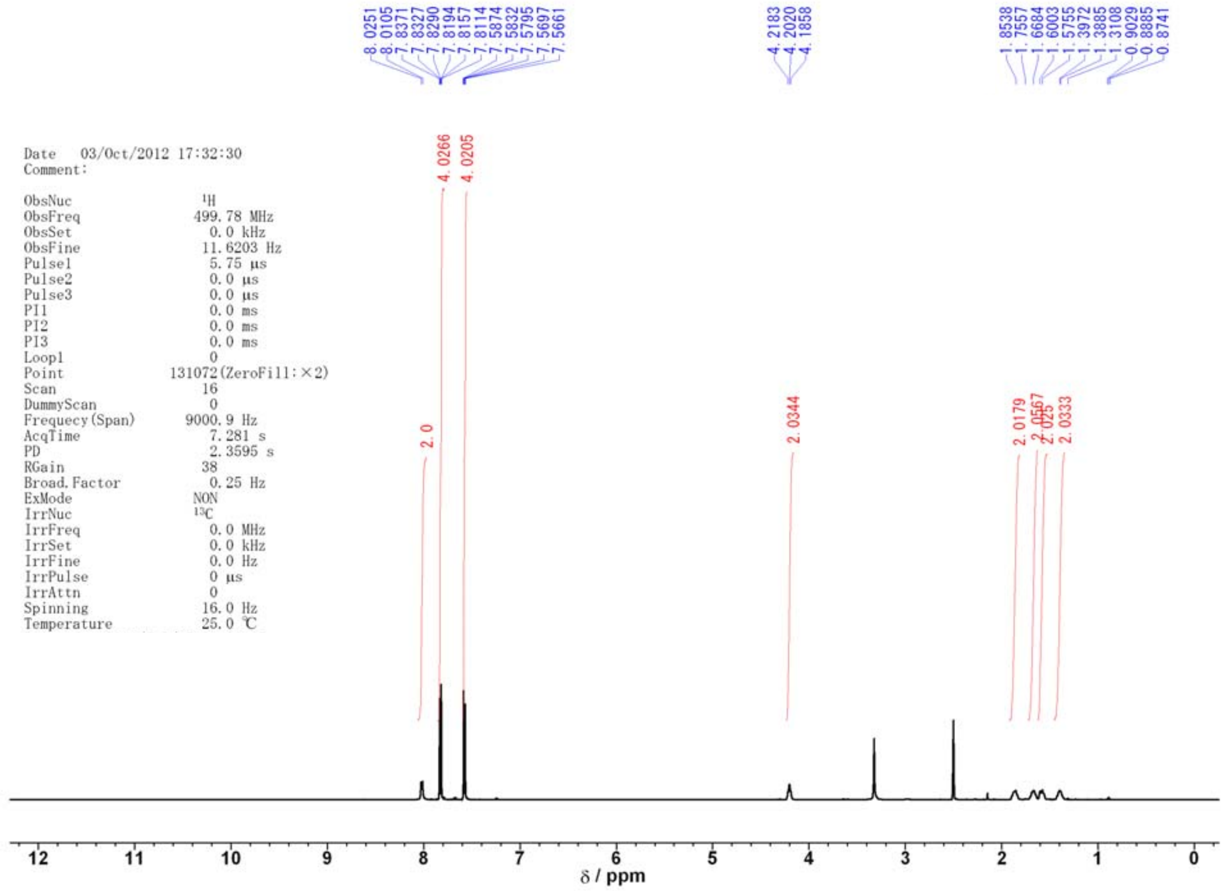

Supplementary Figure 116 | ^{1}H NMR (DMSO-d_6, 500 MHz) spectrum of *meso*-**8**.

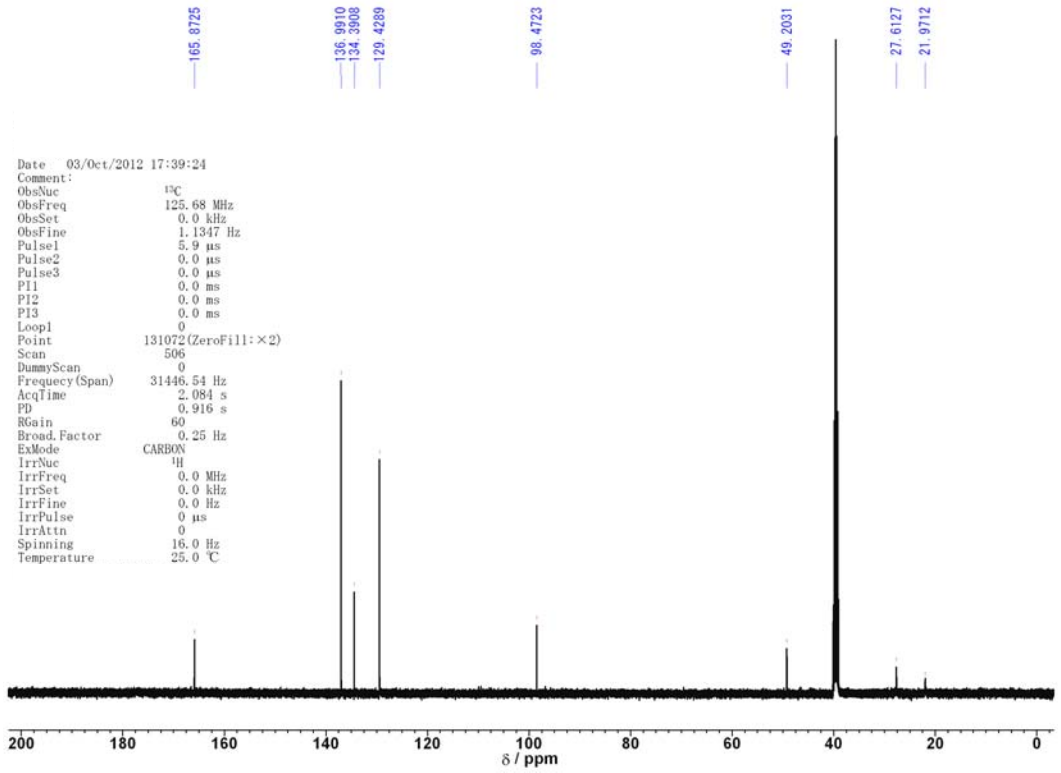

Supplementary Figure 117 | ^{13}C NMR (DMSO-d_6, 125 MHz) spectrum of *meso*-**8**.

2. Supplementary Tables

Supplementary Table 1. Crystal Data and Structure Refinement for (*S*,*S*)-**1a'**.

Elemental formula	$C_{144}H_{124}N_4O_{12}Si_4$, $C_{14}H_{16}$	
Formula weight	2399.10	
Temperature	103(2) K	
Wavelength	0.71075 Å	
Crystal system	Orthorhombic	
Space group	$P2_12_12_1$	
Unit cell dimensions	a = 15.990(2) Å	α = 90°
	b = 28.164(4) Å	β = 90°
	c = 30.875(4) Å	γ = 90°
Volume	13905(3) $Å^3$	
Z	4	
Density (calculated)	1.146 g/cm^3	
Absorption coefficient	0.104 mm^{-1}	
F(000)	5,072	
Crystal size	0.15 × 0.12 × 0.04 mm^3	
Theta range for data collection	3.00 to 26.00°	
Index ranges	$-19 \le h \le 19$, $-31 \le k \le 34$, $-37 \le l \le 37$	
Reflections collected	104,237	
Independent reflections	27,226 [R_{int} = 0.0515]	
Completeness to theta = 26.00°	99.5 %	
Absorption correction	Semi-empirical from equivalents	
Max. and min. transmission	0.9959 and 0.9846	
Refinement method	Full-matrix least-squares on F^2	
Data / restraints / parameters	27,226 / 71 / 1,613	
Goodness-of-fit on F^2	1.125	
Final R indices [I>2sigma(I)]	R_1 = 0.1342, wR_2 = 0.3443	
R indices (all data)	R_1 = 0.1431, wR_2 = 0.3520	
Largest diff. peak and hole	1.641 and −1.005 $eÅ^{-3}$	
Flack parameter 0.0(3)		

Supplementary Table 2. Crystal Data and Structure Refinement for (*S*,*S*)-**2a'**.

Elemental formula	$C_{139}H_{100}N_4O_{12}$, $C_{14}H_{16}$	
Formula weight	2202.50	
Temperature	103(2) K	
Wavelength	0.71075 Å	
Crystal system	Monoclinic	
Space group	$P2_1$	
Unit cell dimensions	a = 12.650(5) Å	α = 90°
	b = 30.179(11) Å	β = 111.130(7)°
	c = 16.608(6) Å	γ = 90°
Volume	5914(4) $Å^3$	
Z	2	
Density (calculated)	1.237 g/cm^3	
Absorption coefficient	0.078 mm^{-1}	
F(000)	2,316	
Crystal size	0.20 × 0.06 × 0.02 mm^3	
Theta range for data collection	3.00 to 26.00°	
Index ranges	$-15 \leq h \leq 15$, $-30 \leq k \leq 37$, $-20 \leq l \leq 20$	
Reflections collected	43,813	
Independent reflections	18,248 [R_{int} = 0.0616]	
Completeness to theta = 26.00°	99.0 %	
Absorption correction	Semi-empirical from equivalents	
Max. and min. transmission	0.9984 and 0.9846	
Refinement method	Full-matrix least-squares on F^2	
Data / restraints / parameters	18,248 / 140 / 1,603	
Goodness-of-fit on F^2	1.149	
Final R indices [I>2sigma(I)]	R_1 = 0.1239, wR_2 = 0.2942	
R indices (all data)	R_1 = 0.1630, wR_2 = 0.3296	
Largest diff. peak and hole	0.333 and -0.341 $eÅ^{-3}$	
Flack parameter	-2(2)	

3. Supplementary Methods

3-1. Materials. All starting materials and dehydrated solvents were purchased from Aldrich, Wako Pure Chemical Industries (Osaka, Japan), and Tokyo Kasei Kogyo (TCI) (Tokyo, Japan) unless otherwise noted. Silica gel (SiO_2) and aminopropyl-modified silica gel (NH-SiO_2) for the flash chromatography were purchased from Merck and Fuji Silysia Chemical Ltd. (Kusanagi, Japan), respectively. Bio-Beads (SX-1) for the size exclusion chromatography (SEC) was purchesed from Bio-Rad Laboratories. The monoethynyl carboxylic acid monomers (**5** and **6**), monoethynyl amidine monomers ((*R*,*R*)-**9** and **10**), chiral linkers ((*R*,*R*)- and (*S*,*S*)-**7**, (*R*,*R*)- and (*S*,*S*)-**8**), and amidine dimers ((*R*,*R*,*R*,*R*,*R*,*R*)-**3a**, (*R*,*R*,*S*,*S*,*R*,*R*)-**3b**, (*R*,*R*)-**3c**, (*R*,*R*)-**3d**, (*R*,*R*,*R*,*R*,*R*,*R*)-**4a**, and (*R*,*R*,*S*,*S*,*R*,*R*)-**4b**) were synthesized according to refs 47, 50, and 53 in the main text.

3-2. Instruments. The melting points were measured using a Yanaco MP-500D melting point apparatus (Kyoto, Japan) and were uncorrected. The NMR spectra were obtained using a Varian UNITY INOVA 500AS spectrometer operating at 500 MHz for ^{1}H and 125 MHz for ^{13}C. Chemical shifts are reported in parts per million (δ) downfield from tetramethylsilane (TMS) as the internal standard in $CDCl_3$ or DMSO-d_6. The recycling preparative HPLC was performed with an LC-928R liquid chromatograph (Japan Analytical Industry, Tokyo, Japan) equipped with two SEC columns (JAIGEL-1H (1 x 60 cm) and JAIGEL-2H (1 x 60 cm)) in series and a UV-visible detector (254 nm, JAI UV-310), and $CHCl_3$ was used as the eluent. The electron spray ionization (ESI) mass spectra were recorded using a JEOL JMS-T100CS spectrometer (Akishima, Japan). The matrix-assisted laser desorption-ionization time-of-flight mass spectra (MALDI-TOF-MS) were obtained using a SHIMADZU AXIMA-CFR Plus mass spectrometer (Kyoto, Japan) with a positive mode using 2,4,6-trihydroxyacetophenone (THAP) as the matrix. The elemental analyses were performed by the laboratory of elemental analyses in the Department of Agriculture, Nagoya University. The IR spectra were recorded using a JASCO Fourier Transform IR-680 spectrophotometer (Hachioji, Japan). The absorption and CD spectra were measured in a 0.01-, 0.05-, 0.1-, 1-, 5-, or 10-cm quartz cell on a JASCO V-570 spectrophotometer and a JASCO J-820 spectropolarimeter, respectively. The optical rotations were taken using a JASCO P-1030 polarimeter in a 2-cm quartz cell equipped with a temperature controller (EYELA NCB-2100). The single-crystal X-ray data were collected on a Rigaku Saturn 724+ CCD diffractometer with Mo Kα radiation (λ = 0.71075 Å) at 103 K. The molecular weight of the duplex ((*R*,*R*,*S*,*S*,*R*,*R*)-**3b**·(*S*,*S*)-**2a**) in $CHCl_3$ was estimated using a Gonotec 070

vapor pressure osmometer (VPO) (Berlin, Germany) with benzil as the standard. The samples were prepared in the range of 4–16 mmol kg^{-1}.

3-3. Synthetic Procedures

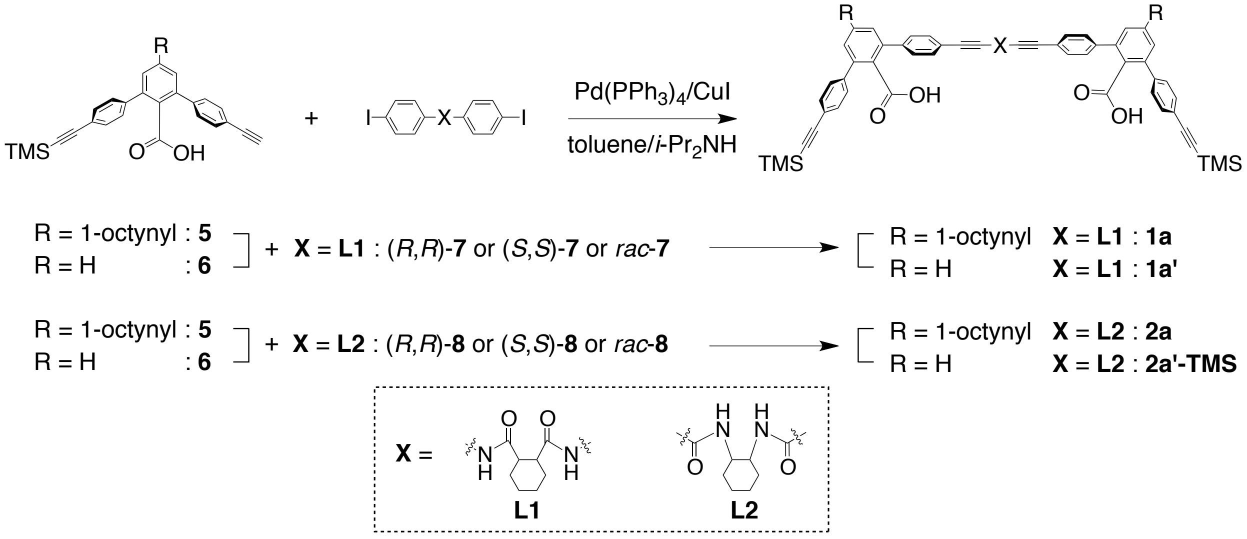

Rac-**7.** Thionyl chloride (1.06 mL, 14.5 mmol) and a catalytic amount of DMF were added to *rac*-1,2-cyclohexanedicarboxylic acid (500 mg, 2.90 mmol), and the mixture was stirred at ambient temperature for 3.5 h. After removing the excess amount of thionyl chloride, the residue was dissolved in anhydrous $CHCl_3$ (5.0 mL), and to this was added an anhydrous $CHCl_3$ solution (2.5 mL) of 4-iodoaniline (1.30 g, 5.94 mmol) and diisopropylamine (2.53 mL, 14.5 mmol) at 0 °C. After the mixture was stirred at ambient temperature for 12 h, the obtained precipitate was collected and washed with water and $CHCl_3$ to afford *rac*-**7** (0.24 g, 14% yield) as a white solid. Mp: 302–304 °C. ^{1}H NMR (500 MHz, DMSO-d_6, 25 °C): δ 10.07 (s, 2H, NH), 7.57 (d, J = 9.0 Hz, 4H, ArH), 7.39 (d, J = 9.0 Hz, 4H, ArH), 2.75–2.63 (m, 2H, COCH), 2.01–1.91 (m, 2H, CH), 1.83–1.73 (m, 2H, CH), 1.41–1.22 (m, 4H, CH). ^{13}C NMR (125 MHz, DMSO-d_6, 25 °C): δ 173.87, 139.30, 137.31, 121.18, 86.16, 46.29, 29.70, 25.23. IR (KBr, cm^{-1}): 3279 ($\nu_{N\text{-}H}$), 1656 ($\nu_{C=O}$). Anal. Calcd for $C_{20}H_{20}I_2N_2O_2$: C, 41.83; H, 3.51; N, 4.88. Found: C, 41.83; H, 3.48; N, 4.94.

Rac-**8.** 4-*N*,*N*-dimethylaminopyridine (35.0 mg, 0.286 mmol) and 1-ethyl-3-(3-dimethylaminopropyl)carbodiimide hydrochloride (647 mg, 3.38 mmol) were added to a suspension of *p*-iodobenzoic acid (745 mg, 3.00 mmol) and *rac*-1,2-cyclohexanediamine (153 mg, 1.34 mmol) in dichloromethane (10 mL) at 0 °C. After the mixture was stirred at

ambient temperature for 4 h, the obtained precipitate was collected and washed with water and $CHCl_3$ to afford *rac*-**8** (0.50 g, 65% yield) as a white solid. Mp: 314–317 °C. ^1H NMR (500 MHz, DMSO-d_6, 25 °C): δ 8.30 (d, J = 8.1 Hz, 2H, NH), 7.77 (d, J = 8.5 Hz, 4H, ArH), 7.47 (d, J = 8.5 Hz, 4H, ArH), 3.96–3.85 (m, 2H, NCH), 1.91–1.81 (m, 2H, CH), 1.77–1.67 (m, 2H, CH), 1.55–1.42 (m, 2H, CH), 1.35–1.19 (m, 2H, CH). ^{13}C NMR (125 MHz, DMSO-d_6, 25 °C): δ 165.63, 136.98, 134.34, 129.13, 98.41, 52.92, 31.50, 24.67. IR (KBr, cm^{-1}): 3278 (ν_{N-H}), 1639 ($\nu_{C=O}$), 1550 (ν_{C-N}). Anal. Calcd for $C_{20}H_{20}I_2N_2O_2$: C, 41.83; H, 3.51; N, 4.88. Found: C, 41.72; H, 3.51; N, 4.90.

General Procedures for the Preparation of Carboxylic Acid Dimers (1a and 2a). A typical experimental procedure is described below. CuI (2.8 mg, 0.015 mmol) was added to a solution of monoethynyl carboxylic acid monomer (**5**) (74.4 mg, 0.148 mmol), *rac*-**8** (42.8 mg, 0.0745 mmol), and $Pd(PPh_3)_4$ (17 mg, 0.015 mmol) in toluene-diisopropylamine (8/2 (v/v), 3.0 mL). After the mixture was stirred at 65 °C for 10 h under nitrogen, the solvents were evaporated to dryness. The residue was then dissolved in $CHCl_3$, and the solution was washed with 1 M HCl and brine, and dried over anhydrous Na_2SO_4. After filtration, the solvent was evaporated to dryness and the residue was purified by column chromatography (SiO_2, $CHCl_3$/MeOH = 99/1 (v/v)), Bio-Beads (SX-1, $CHCl_3$), and preparative recycling HPLC ($CHCl_3$) to afford *rac*-**2a** (22 mg, 24% yield) as a pale yellow solid. In the same way, *rac*-**1a**, (*R*,*R*)-**1a**, (*S*,*S*)-**1a**, (*S*,*S*)-**1a'**, (*R*,*R*)-**2a**, (*S*,*S*)-**2a**, and (*S*,*S*)-**2a'-TMS** were prepared.

Rac-**1a.** 60% yield. Mp: 200 °C (dec.). ^1H NMR (500 MHz, DMSO-d_6, 25 °C): δ 13.11 (s, 2H, CO_2H), 10.21 (s, 2H, NH), 7.65 (d, J = 8.7 Hz, 4H, ArH), 7.57 (d, J = 8.4 Hz, 4H, ArH), 7.51 (d, J = 8.4 Hz, 4H, ArH), 7.47 (d, J = 8.4 Hz, 4H, ArH), 7.45 (d, J = 8.7 Hz, 4H, ArH), 7.42 (d, J = 8.4 Hz, 4H, ArH), 7.38 (s, 2H, ArH), 7.36 (s, 2H, ArH), 2.80–2.70 (m, 2H, COCH), 2.44 (t, J = 7.0 Hz, 4H, $C\equiv CCH_2$), 2.09–1.96 (m, 2H, CH), 1.88–1.75 (m, 2H, CH), 1.57–1.49 (m, 4H, CH_2), 1.46–1.20 (m, 16H, CH and CH_2), 0.86 (t, J = 6.5 Hz, 6H, CH_3), 0.24 (s, 18H, $SiCH_3$). ^{13}C NMR (125 MHz, DMSO-d_6, 25 °C): δ 173.88, 169.41, 139.96, 139.83, 139.27, 138.69, 138.56, 132.99, 132.05, 131.58, 131.40, 131.31, 131.20, 128.62, 128.59, 124.24, 122.09, 121.65, 118.77, 116.10, 104.80, 95.11, 92.73, 90.43, 88.08, 79.45, 46.26, 30.72, 29.66, 27.94, 27.94, 25.18, 21.96, 18.65, 13.89, –0.12. IR (KBr, cm^{-1}): 3296 (ν_{N-H}), 2157 ($\nu_{C\equiv C}$), 1701 ($\nu_{C=O}$). HRMS (ESI): m/z calcd for $[M(C_{88}H_{86}N_2O_6Si_2)–H]^-$, 1321.5946; found 1321.5936.

(R,R)-**1a.** 49% yield. Mp: 200 °C (dec.). $[\alpha]_D^{20}$ –586 (c = 0.1 in $CHCl_3$). ^{1}H NMR (500 MHz, DMSO-d_6, 25 °C): δ 13.11 (s, 2H, CO_2H), 10.21 (s, 2H, NH), 7.65 (d, J = 8.7 Hz, 4H, ArH), 7.57 (d, J = 8.2 Hz, 4H, ArH), 7.50 (d, J = 8.0 Hz, 4H, ArH), 7.46 (d, J = 8.4 Hz, 4H, ArH), 7.45 (d, J = 8.1 Hz, 4H, ArH), 7.42 (d, J = 8.4 Hz, 4H, ArH), 7.38 (s, 2H, ArH), 7.36 (s, 2H, ArH), 2.83–2.68 (m, 2H, COCH), 2.44 (t, J = 7.0 Hz, 4H, C≡CCH_2), 2.10–1.94 (m, 2H, CH), 1.88–1.74 (m, 2H, CH), 1.61–1.49 (m, 4H, CH_2), 1.47–1.20 (m, 16H, CH and CH_2), 0.86 (t, J = 6.5 Hz, 6H, CH_3), 0.24 (s, 18H, $SiCH_3$). ^{13}C NMR (125 MHz, DMSO-d_6, 25 °C): δ 173.90, 169.41, 139.98, 139.82, 139.26, 138.72, 138.60, 132.96, 132.07, 131.60, 131.42, 131.33, 131.22, 128.63, 128.60, 124.29, 122.12, 121.67, 118.78, 116.11, 104.80, 95.14, 92.77, 90.45, 88.09, 79.45, 46.26, 30.73, 29.69, 27.96, 27.96, 25.21, 21.97, 18.66, 13.90, –0.11. IR (KBr, cm^{-1}): 3301 ($\nu_{N\text{–}H}$), 2158 ($\nu_{C\equiv C}$), 1701 ($\nu_{C=O}$). HRMS (ESI): m/z calcd for $[M(C_{88}H_{86}N_2O_6Si_2)–H]^-$, 1321.5946; found 1321.5975.

(S,S)-**1a.** 51% yield. Mp: 200 °C (dec.). $[\alpha]_D^{20}$ 590 (c = 0.1 in $CHCl_3$). ^{1}H NMR (500 MHz, DMSO-d_6, 25 °C): δ 13.13 (s, 2H, CO_2H), 10.23 (s, 2H, NH), 7.65 (d, J = 8.6 Hz, 4H, ArH), 7.57 (d, J = 8.2 Hz, 4H, ArH), 7.51 (d, J = 8.2 Hz, 4H, ArH), 7.47 (d, J = 8.7 Hz, 4H, ArH), 7.45 (d, J = 8.6 Hz, 4H, ArH), 7.42 (d, J = 8.3 Hz, 4H, ArH), 7.39 (s, 2H, ArH), 7.36 (s, 2H, ArH), 2.79–2.69 (m, 2H, COCH), 2.44 (t, J = 7.0 Hz, 4H, C≡CCH_2), 2.07–1.96 (m, 2H, CH), 1.88–1.75 (m, 2H, CH), 1.59–1.49 (m, 4H, CH_2), 1.46–1.19 (m, 16H, CH and CH_2), 0.86 (t, J = 6.5 Hz, 6H, CH_3), 0.24 (s, 18H, $SiCH_3$). ^{13}C NMR (125 MHz, DMSO-d_6, 25 °C): δ 173.90, 169.41, 139.98, 139.82, 139.25, 138.73, 138.61, 132.96, 132.07, 131.60, 131.42, 131.32, 131.22, 128.63, 128.60, 124.29, 122.12, 121.67, 118.78, 116.11, 104.80 95.14, 92.77, 90.46, 88.09, 79.45, 46.25, 30.73, 29.68, 27.96, 27.96, 25.21, 21.97, 18.66, 13.90, –0.12. IR (KBr, cm^{-1}): 3301 ($\nu_{N\text{–}H}$), 2158 ($\nu_{C\equiv C}$), 1701 ($\nu_{C=O}$). HRMS (ESI): m/z calcd for $[M(C_{88}H_{86}N_2O_6Si_2)–H]^-$, 1321.5946; found 1321.5942.

(S,S)-**1a'.** 67% yield. Mp: 211 °C (dec.). $[\alpha]_D^{20}$ 718 (c = 0.1 in $CHCl_3$). ^{1}H NMR (500 MHz, DMSO-d_6, 25 °C): δ 13.00 (br s, 2H, CO_2H), 10.22 (s, 2H, NH), 7.65 (d, J = 8.7 Hz, 4H, ArH), 7.61–7.55 (m, 6H, ArH), 7.51 (d, J = 8.5 Hz, 4H, ArH), 7.49–7.37 (m, 16H, ArH), 2.82–2.70 (m, 2H, COCH), 2.11–1.94 (m, 2H, CH), 1.89–1.74 (m, 2H, CH), 1.51–1.26 (m, 4H, CH), 0.24 (s, 18H, $SiCH_3$). ^{13}C NMR (125 MHz, DMSO-d_6, 25 °C): δ 173.91, 169.88, 140.69, 140.13, 139.95, 138.17, 138.04, 133.67, 132.06, 131.58, 131.19, 129.29, 129.13, 129.02, 128.67, 128.64, 121.84,

121.40, 118.78, 116.14, 104.88, 94.99, 90.31, 88.15, 46.25, 29.70, 25.21, –0.09. IR (KBr, cm^{-1}): 3312 ($\nu_{N–H}$), 2156 ($\nu_{C≡C}$), 1702 ($\nu_{C=O}$). HRMS (ESI): m/z calcd for $[M(C_{72}H_{62}N_2O_6Si_2)–H]^-$, 1105.4068; found 1105.4024.

Rac-**2a.** 24% yield. Mp: 200 °C (dec.). ^{1}H NMR (500 MHz, DMSO-d_6, 25 °C): δ 13.13 (br s, 2H, CO_2H), 8.37 (d, J = 7.0 Hz, 2H, NH), 7.77 (d, J = 8.5 Hz, 4H, ArH), 7.61 (d, J = 7.2 Hz, 4H, ArH), 7.60 (d, J = 8.5 Hz, 4H, ArH), 7.50 (d, J = 8.2 Hz, 4H, ArH), 7.48 (d, J = 7.2 Hz, 4H, ArH), 7.43 (d, J = 8.2 Hz, 4H, ArH), 7.38 (s, 2H, ArH), 7.36 (s, 2H, ArH), 4.02–3.91 (m, 2H, NCH), 2.44 (t, J = 7.0 Hz, 4H, $C≡CCH_2$), 1.96–1.86 (m, 2H, CH), 1.82–1.70 (m, 2H, CH), 1.61–1.48 (m, 6H, CH_2), 1.46–1.37 (m, 4H, CH and CH_2), 1.35–1.19 (m, 10H, CH_2), 0.86 (t, J = 7.0 Hz, 6H, CH_3), 0.24 (s, 18H, $SiCH_3$). ^{13}C NMR (125 MHz, DMSO-d_6, 25 °C): δ 169.48, 165.60, 139.93, 139.91, 138.53, 138.51, 134.77, 133.26, 131.62, 131.57, 131.43, 131.39, 131.16, 128.73, 128.61, 127.55, 124.62, 124.12, 121.62, 121.41, 104.83, 95.08, 92.66, 90.74, 89.54, 79.48, 53.04, 31.55, 30.73, 27.96, 27.96, 24.71, 21.97, 18.66, 13.89, –0.12. IR (KBr, cm^{-1}): 3338 ($\nu_{N–H}$), 2157 ($\nu_{C≡C}$), 1708 ($\nu_{C=O}$). HRMS (ESI): m/z calcd for $[M(C_{88}H_{86}N_2O_6Si_2)–H]^-$, 1321.5946; found 1321.5966.

(*R*,*R*)-**2a.** 30% yield. Mp: 200 °C (dec.). $[\alpha]_D^{20}$ –516 (c = 0.1 in $CHCl_3$). ^{1}H NMR (500 MHz, DMSO-d_6, 25 °C): δ 13.12 (br s, 2H, CO_2H), 8.37 (d, J = 7.0 Hz, 2H, NH), 7.77 (d, J = 8.4 Hz, 4H, ArH), 7.61 (d, J = 8.0 Hz, 4H, ArH), 7.60 (d, J = 8.0 Hz, 4H, ArH), 7.50 (d, J = 7.5 Hz, 4H, ArH), 7.48 (d, J = 7.5 Hz, 4H, ArH), 7.43 (d, J = 8.4 Hz, 4H, ArH), 7.38 (s, 2H, ArH), 7.36 (s, 2H, ArH), 4.04–3.88 (m, 2H, NCH), 2.44 (t, J = 7.0 Hz, 4H, $C≡CCH_2$), 1.98–1.85 (m, 2H, CH), 1.82–1.69 (m, 2H, CH), 1.60–1.48 (m, 6H, CH_2), 1.45–1.37 (m, 4H, CH and CH_2), 1.34–1.22 (m, 10H, CH_2), 0.86 (t, J = 7.0 Hz, 6H, CH_3), 0.24 (s, 18H, $SiCH_3$). ^{13}C NMR (125 MHz, DMSO-d_6, 25 °C): δ 169.39, 165.59, 139.84, 139.81, 138.64, 138.61, 134.80, 133.00, 131.68, 131.60, 131.46, 131.42, 131.17, 128.72, 128.60, 127.55, 124.60, 124.29, 121.67, 121.47, 104.80, 95.14, 92.79, 90.71, 89.57, 79.44, 53.04, 31.55, 30.73, 27.96, 27.96, 24.71, 21.97, 18.66, 13.89, –0.12. IR (KBr, cm^{-1}): 3338 ($\nu_{N–H}$), 2157 ($\nu_{C≡C}$), 1703 ($\nu_{C=O}$). HRMS (ESI): m/z calcd for $[M(C_{88}H_{86}N_2O_6Si_2)–H]^-$, 1321.5946; found 1321.5943.

(*S*,*S*)-**2a.** 55% yield. Mp: 200 °C (dec.). $[\alpha]_D^{20}$ 519 (c = 0.1 in $CHCl_3$). ^{1}H NMR (500 MHz, DMSO-d_6, 25 °C): δ 13.17 (br s, 2H, CO_2H), 8.39 (d, J = 7.2 Hz, 2H, NH), 7.77 (d, J = 8.3 Hz,

4H, ArH), 7.61 (d, J = 8.0 Hz, 4H, ArH), 7.60 (d, J = 8.0 Hz, 4H, ArH), 7.50 (d, J = 8.2 Hz, 4H, ArH), 7.47 (d, J = 8.3 Hz, 4H, ArH), 7.42 (d, J = 8.3 Hz, 4H, ArH), 7.38 (s, 2H, ArH), 7.36 (s, 2H, ArH), 4.05–3.86 (m, 2H, NCH), 2.43 (t, J = 7.0 Hz, 4H, C≡CCH_2), 1.98–1.85 (m, 2H, CH), 1.82–1.70 (m, 2H, CH), 1.61–1.48 (m, 6H, CH_2), 1.46–1.36 (m, 4H, CH and CH_2), 1.34–1.21 (m, 10H, CH_2), 0.85 (t, J = 7.0 Hz, 6H, CH_3), 0.24 (s, 18H, $SiCH_3$). ^{13}C NMR (125 MHz, DMSO-d_6, 25 °C): δ 169.45, 165.60, 139.90, 139.87, 138.57, 138.55, 134.79, 133.19, 131.69, 131.58, 131.45, 131.40, 131.17, 128.73, 128.60, 127.55, 124.61, 124.20, 121.64, 121.44, 104.82, 95.11, 92.71, 90.73, 89.56, 79.47, 53.05, 31.55, 30.73, 27.96, 27.96, 24.71, 21.97, 18.66, 13.89, –0.12. IR (KBr, cm^{-1}): 3314 (ν_{N-H}), 2157 ($\nu_{C≡C}$), 1705 ($\nu_{C=O}$). HRMS (ESI): m/z calcd for [M($C_{88}H_{86}N_2O_6Si_2$)–H]$^-$, 1321.5946; found 1321.5965.

(*S*,*S*)-**2a'-TMS.** 57% yield. Mp: 200 °C (dec.). $[\alpha]_D^{20}$ 603 (c = 0.2 in $CHCl_3$). ^{1}H NMR (500 MHz, DMSO-d_6, 25 °C): δ 13.02 (br s, 2H, CO_2H), 8.39 (d, J = 7.7 Hz, 2H, NH), 7.78 (d, J = 8.6 Hz, 4H, ArH), 7.65–7.56 (m, 10H, ArH), 7.52 (d, J = 8.5 Hz, 4H, ArH), 7.48 (d, J = 8.5 Hz, 4H, ArH), 7.46–7.39 (m, 8H, ArH), 4.04–3.88 (m, 2H, NCH), 1.98–1.85 (m, 2H, CH), 1.82–1.69 (m, 2H, CH), 1.62–1.46 (m, 2H, CH), 1.38–1.21 (m, 2H, CH), 0.24 (s, 18H, $SiCH_3$). ^{13}C NMR (125 MHz, DMSO-d_6, 25 °C): δ 169.87, 165.60, 140.71, 140.67, 138.10, 138.08, 134.79, 133.65, 131.59, 131.45, 131.18, 129.33, 129.13, 128.76, 128.64, 127.57, 124.64, 121.42, 121.21, 104.88, 95.00, 90.79, 89.46, 79.17, 53.03, 31.56, 24.70, –0.09. IR (KBr, cm^{-1}): 3337 (ν_{N-H}), 2156 ($\nu_{C≡C}$), 1707 ($\nu_{C=O}$). HRMS (ESI): m/z calcd for [M($C_{72}H_{62}N_2O_6Si_2$)–H]$^-$, 1105.4068; found 1105.4069.

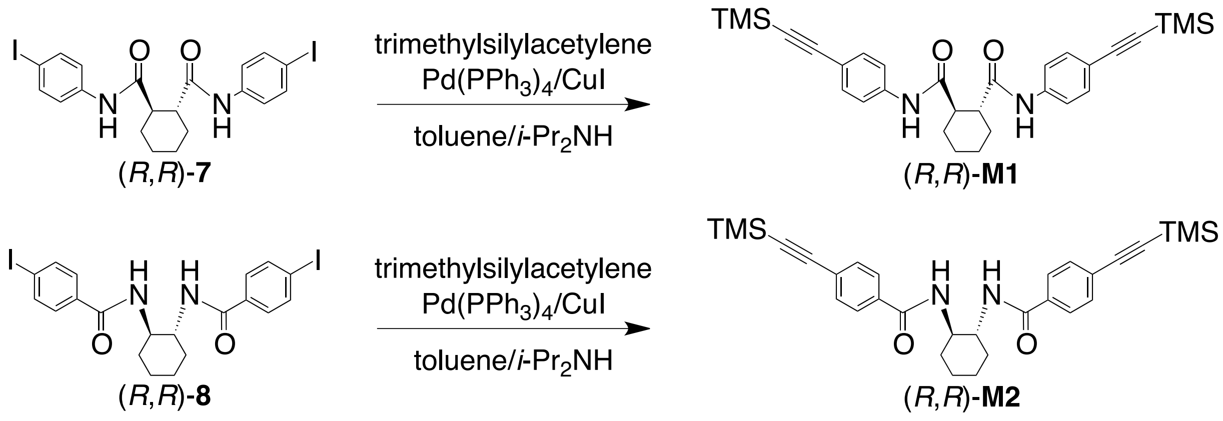

General Procedures for the Preparation of Model Compounds M1 and M2. A typical experimental procedure is described below. Trimethylsilylacetylene (26 μL, 0.18 mmol) was added to a suspension of CuI (2.5 mg, 13 μmol), $Pd(PPh_3)_4$ (15.1 mg, 13.1 μmol), and (*R*,*R*)-**8**

(50.0 mg, 87.1 μmol) in toluene-diisopropylamine (8/2 (v/v), 5.0 mL) at 0 °C. After the mixture was stirred at room tenmperature for 10 h under nitrogen, the solvents were evaporated to dryness. The residue was then purified by column chromatography (SiO_2, $CHCl_3$/MeOH = 100/4 (v/v)) to afford (*R*,*R*)-**M2** (26 mg, 60% yield) as a white solid. In the same way, (*R*,*R*)-**M1** was prepared.

(*R*,*R*)**-M1.** 46% yield. Mp: 310−311 °C. $[\alpha]_D^{20}$ −197 (*c* = 0.5 in $CHCl_3$). ^{1}H NMR (500 MHz, DMSO-d_6, 25 °C): δ 10.16 (s, 2H, NH), 7.56 (d, *J* = 8.8 Hz, 4H, ArH), 7.33 (d, *J* = 8.8 Hz, 4H, ArH), 2.75−2.68 (m, 2H, COCH), 2.05−1.93 (m, 2H, CH), 1.85−1.75 (m, 2H, CH), 1.40−1.25 (m, 4H, CH), 0.20 (s, 18H, $SiCH_3$). ^{13}C NMR (125 MHz, DMSO-d_6, 25 °C): δ 173.87, 139.99, 132.23, 118.60, 116.14, 105.48, 92.86, 46.18, 29.64, 25.18, –0.04. IR (KBr, cm^{-1}): 3296 (ν_{N-H}), 2157 ($\nu_{C\equiv C}$), 1659 ($\nu_{C=O}$). HRMS (ESI): m/z calcd for $[M(C_{30}H_{38}N_2O_2Si_2)+Na]^+$, 537.2369; found 537.2376.

(*R*,*R*)**-M2.** 60% yield. Mp: 310−313 °C. $[\alpha]_D^{20}$ −332 (*c* = 0.2 in $CHCl_3$). ^{1}H NMR (500 MHz, $CDCl_3$, 25 °C, 10 mM): δ 7.64 (d, *J* = 8.5 Hz, 4H, ArH), 7.45 (d, *J* = 8.5 Hz, 4H, ArH), 6.75 (d, *J* = 6.9 Hz, 2H, NH), 4.04−3.93 (m, 2H, NCH), 2.28−2.16 (m, 2H, CH), 1.91−1.79 (m, 2H, CH), 1.50−1.36 (m, 4H, CH), 0.25 (s, 18H, $SiCH_3$). ^{13}C NMR (125 MHz, $CDCl_3$, 25 °C, 10 mM): δ 167.63, 133.72, 132.21, 126.94, 126.59, 104.19, 97.12, 54.83, 32.50, 24.93, 0.00. IR (KBr, cm^{-1}): 3304 (ν_{N-H}), 2159 ($\nu_{C\equiv C}$), 1633 ($\nu_{C=O}$). HRMS (ESI): m/z calcd for $[M(C_{30}H_{38}N_2O_2Si_2)+Na]^+$, 537.2369; found 537.2349.

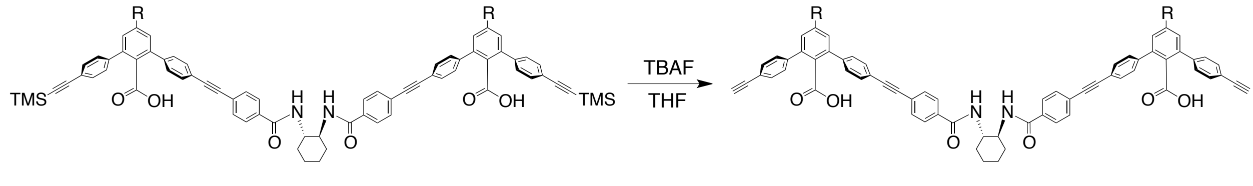

R = 1-octynyl : (*S*,*S*)-**2a**
R = H : (*S*,*S*)-**2a'-TMS**

R = 1-octynyl : (*S*,*S*)-**2c**
R = H : (*S*,*S*)-**2a'**

General Procedures for the Preparation of 2c and 2a’. To a solution of (*S*,*S*)-**2a** (25.2 mg, 19.0 μmol) in THF (2.0 mL) was added tetrabutylammonium fluoride (TBAF) in THF (1.0 M, 60.0 μL, 60.0 μmol) at 0 °C. After the mixture was stirred at ambient temperature for 2 h, the solvent was evaporated to dryness. The residue was then dissolved in $CHCl_3$ and the solution was washed with 1M HCl and brine, and dried over anhydrous Na_2SO_4. After filtration, the

solvent was evaporated to dryness and the residue was purified by column chromatography (SiO_2, $CHCl_3$/MeOH = 9/1 (v/v)) and preparative recycling HPLC ($CHCl_3$) to afford (*S*,*S*)-**2c** (7.8 mg, 35% yield) as a pale yellow solid. In the same way, (*S*,*S*)-**2a**' was also prepared.

(*S*,*S*)**-2c.** 35% yield. Mp: 200 °C (dec.). $[\alpha]_D^{20}$ 512 (c = 0.1 in $CHCl_3$). ^{1}H NMR (500 MHz, DMSO-d_6, 25 °C): δ 13.13 (br s, 2H, CO_2H), 8.39 (d, J = 7.3 Hz, 2H, NH), 7.78 (d, J = 8.4 Hz, 4H, ArH), 7.62 (d, J = 7.8 Hz, 4H, ArH), 7.60 (d, J = 7.5 Hz, 4H, ArH), 7.54 (d, J = 8.2 Hz, 4H, ArH), 7.48 (d, J = 8.3 Hz, 4H, ArH), 7.43 (d, J = 8.3 Hz, 4H, ArH), 7.39 (s, 2H, ArH), 7.37 (s, 2H, ArH), 4.26 (s, 2H, C≡CH), 4.03–3.89 (m, 2H, NCH), 2.44 (t, J = 7.0 Hz, 4H, C≡CCH_2), 1.96–1.86 (m, 2H, CH), 1.80–1.71 (m, 2H, CH), 1.59–1.48 (m, 6H, CH_2), 1.45–1.36 (m, 4H, CH and CH_2), 1.34–1.19 (m, 10H, CH_2), 0.86 (t, J = 6.9 Hz, 6H, CH_3). ^{13}C NMR (125 MHz, DMSO-d_6, 25 °C): δ 169.39, 165.58, 139.85, 139.80, 138.63, 138.59, 134.80, 131.70, 131.46, 131.43, 131.41, 131.17, 131.15, 128.73, 128.61, 127.55, 124.60, 124.26, 121.46, 121.26, 92.77, 90.71, 89.56, 83.09, 81.60, 79.46, 53.03, 31.55, 30.72, 27.04, 27.04, 24.70, 21.97, 18.66, 13.89. IR (KBr, cm^{-1}): 3297 ($\nu_{C≡CH}$), 2228 ($\nu_{C≡C}$), 1704 ($\nu_{C=O}$). HRMS (ESI): m/z calcd for $[M(C_{82}H_{70}N_2O_6)–H]^-$, 1177.5156; found 1177.5109.

(*S*,*S*)**-2a'.** 86% yield. Mp: 237 °C (dec.). $[\alpha]_D^{20}$ 289 (c = 0.1 in THF). ^{1}H NMR (500 MHz, DMSO-d_6, 25 °C): δ 12.98 (br s, 2H, CO_2H), 8.40 (d, J = 7.6 Hz, 2H, NH), 7.77 (d, J = 8.5 Hz, 4H, ArH), 7.65–7.57 (m, 8H, ArH), 7.56–7.48 (m, 10H, ArH), 7.46 (d, J = 8.1 Hz, 4H, ArH), 7.43–7.34 (m, 4H, ArH), 4.23 (s, 2H, C≡CH), 4.01–3.92 (m, 2H, NCH), 1.97–1.85 (m, 2H, CH), 1.82–1.69 (m, 2H, CH), 1.61–1.46 (m, 2H, CH), 1.38–1.26 (m, 2H, CH). ^{13}C NMR (125 MHz, DMSO-d_6, 25 °C): δ 170.31, 165.82, 141.11, 141.06, 137.89, 137.85, 134.78, 131.73, 131.50, 131.28, 129.15, 128.92, 128.80, 127.68, 127.65, 127.60, 124.82, 121.11, 120.91, 91.01, 89.46, 83.36, 81.41, 53.17, 41.43, 31.65, 24.79. IR (film, cm^{-1}): 3292 ($\nu_{C≡CH}$), 2217 ($\nu_{C≡C}$), 1698 ($\nu_{C=O}$). HRMS (ESI): m/z calcd for $[M(C_{66}H_{46}N_2O_6)–H]^-$, 961.3278; found 961.3280.

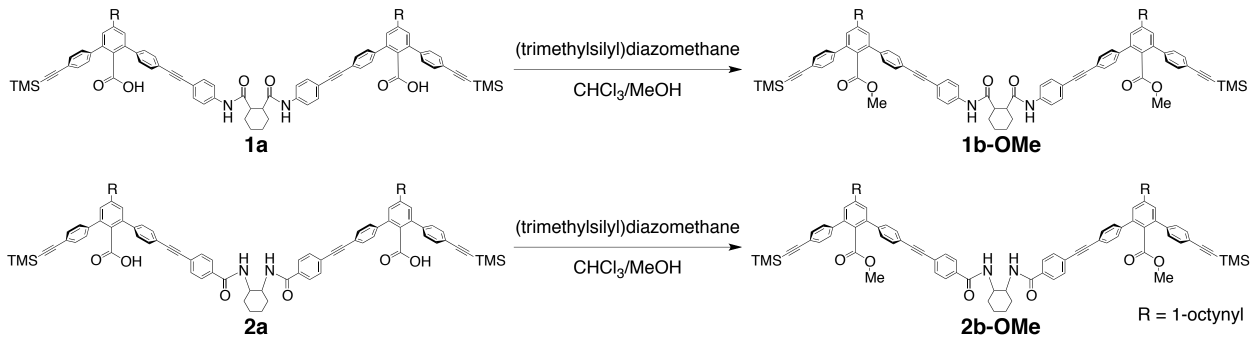

General Procedures for Methyl Esterification of 1 and 2. To a solution of (*R*,*R*)-**2a** (13.0 mg, 9.82 μmol) in $CHCl_3$/MeOH (1/1 (v/v), 1 mL) was added (trimethylsilyl)diazomethane in *n*-hexane (0.6 M, 91 μL, 55 μmol) at ambient temperature. After the mixture was stirred at ambient temperature for 4 h, the solvents were evaporated to dryness. The residue was then purified by column chromatography (NH-SiO_2, $CHCl_3$) to afford (*R*,*R*)-**2b-OMe** (12 mg, 90% yield) as a pale yellow solid. In the same way, (*R*,*R*)-**1b-OMe**, (*S*,*S*)-**1b-OMe**, and (*S*,*S*)-**2b-OMe** were prepared.

(*R*,*R*)-1b-OMe. 68% yield. Mp: 220 °C (dec.). $[\alpha]_D^{20}$ −146 (*c* = 0.2 in $CHCl_3$). ^{1}H NMR (500 MHz, $CDCl_3$, 25 °C, 5.1 mM): δ 7.61 (br s, 2H, NH), 7.52 (d, *J* = 8.4 Hz, 4H, ArH), 7.49 (d, *J* = 8.5 Hz, 4H, ArH), 7.43 (br s, 8H, ArH), 7.39 (s, 2H, ArH), 7.37 (s, 2H, ArH), 7.33 (d, *J* = 8.5 Hz, 4H, ArH), 7.30 (d, *J* = 8.5 Hz, 4H, ArH), 3.37 (s, 6H, CH_3CO_2), 2.73–2.59 (m, 2H, COCH), 2.40 (t, *J* = 7.1 Hz, 4H, C≡CCH_2), 2.11–1.99 (m, 2H, CH), 1.96–1.81 (m, 2H, CH), 1.76–1.53 (m, 8H, CH and CH_2), 1.50–1.21 (m, 16H, CH and CH_2), 0.89 (t, *J* = 7.0 Hz, 6H, CH_3), 0.26 (s, 18H, $SiCH_3$). ^{13}C NMR (125 MHz, $CDCl_3$, 25 °C, 5.1 mM): δ 173.62, 169.34, 140.13, 140.12, 140.06, 139.74, 137.85, 132.50, 132.11, 131.97, 131.89, 131.67, 131.57, 128.46, 128.35, 125.79, 122.98, 122.73, 119.81, 119.09, 104.89, 95.26, 92.95, 90.15, 88.92, 79.67, 52.16, 48.51, 31.49, 29.49, 28.77, 28.71, 25.07, 22.68, 19.60, 14.20, 0.10. IR (film, cm^{-1}): 3304 (ν_{N-H}), 2158 ($\nu_{C\equiv C}$), 1733 ($\nu_{C=O}$). HRMS (ESI): m/z calcd for [M($C_{90}H_{90}N_2O_6Si_2$)+Cl]$^-$, 1385.6026; found 1385.5984.

(*S*,*S*)-1b-OMe. 97% yield. Mp: 223 °C (dec.). $[\alpha]_D^{20}$ 146 (*c* = 0.2 in $CHCl_3$). ^{1}H NMR (500 MHz, $CDCl_3$, 25 °C, 5.2 mM): δ 7.68 (br s, 2H, NH), 7.51 (d, *J* = 8.5 Hz, 4H, ArH), 7.48 (d, *J* = 8.5 Hz, 4H, ArH), 7.42 (br s, 8H, ArH), 7.38 (s, 2H, ArH), 7.37 (s, 2H, ArH), 7.32 (d, *J* = 8.5 Hz, 4H, ArH), 7.30 (d, *J* = 8.5 Hz, 4H, ArH), 3.37 (s, 6H, CH_3CO_2), 2.74–2.61 (m, 2H, COCH), 2.40 (t, *J* = 7.1 Hz, 4H, C≡CCH_2), 2.12–1.99 (m, 2H, CH), 1.97–1.83 (m, 2H, CH), 1.74–1.55 (m, 8H, CH and CH_2), 1.48–1.23 (m, 16H, CH and CH_2), 0.89 (t, *J* = 7.0 Hz, 6H, CH_3), 0.26 (s, 18H,

$SiCH_3$). ^{13}C NMR (125 MHz, $CDCl_3$, 25 °C, 5.2 mM): δ 173.68, 169.34, 140.13, 140.12, 140.06, 139.73, 137.86, 132.49, 132.11, 131.96, 131.89, 131.67, 131.56, 128.45, 128.35, 125.79, 122.98, 122.73, 119.82, 119.07, 104.90, 95.25, 92.95, 90.17, 88.91, 79.67, 52.16, 48.48, 31.49, 29.53, 28.77, 28.71, 25.10, 22.68, 19.60, 14.20, 0.10. IR (film, cm^{-1}): 3303 (ν_{N-H}), 2158 ($\nu_{C\equiv C}$), 1733 ($\nu_{C=O}$). HRMS (ESI): m/z calcd for $[M(C_{90}H_{90}N_2O_6Si_2)+Cl]^-$, 1385.6026; found 1385.6089.

(*R*,*R*)-**2b-OMe.** 90% yield. Mp: 210 °C (dec.). $[\alpha]_D^{20}$ −233 (*c* = 0.2 in $CHCl_3$). ^{1}H NMR (500 MHz, $CDCl_3$, 25 °C, 5.0 mM): δ 7.73 (d, *J* = 8.5 Hz, 4H, ArH), 7.55 (d, *J* = 8.1 Hz, 8H, ArH), 7.49 (d, *J* = 8.4 Hz, 4H, ArH), 7.40 (s, 2H, ArH), 7.38 (s, 2H, ArH), 7.36 (d, *J* = 8.4 Hz, 4H, ArH), 7.31 (d, *J* = 8.4 Hz, 4H, ArH), 6.81 (d, *J* = 7.5 Hz, 2H, NH), 4.10–3.91 (m, 2H, NCH), 3.39 (s, 6H, CH_3CO_2), 2.41 (t, *J* = 7.1 Hz, 4H, $C\equiv CCH_2$), 2.31–2.16 (m, 2H, CH), 1.93–1.78 (m, 2H, CH), 1.65–1.55 (m, 6H, CH_2), 1.51–1.23 (m, 18H, CH and CH_2), 0.89 (t, *J* = 7.0 Hz, 6H, CH_3), 0.26 (s, 18H, $SiCH_3$). ^{13}C NMR (125 MHz, $CDCl_3$, 25 °C, 6.5 mM): δ 169.29, 167.64, 140.29, 140.11, 140.09, 140.01, 133.63, 132.12, 131.98, 131.94, 131.89, 131.85, 131.56, 128.55, 128.33, 127.14, 126.68, 125.83, 122.76, 122.43, 104.87, 95.29, 93.03, 91.59, 89.57, 79.64, 54.89, 52.17, 32.53, 31.49, 28.77, 28.71, 24.94, 22.68, 19.60, 14.20, 0.10. IR (film, cm^{-1}): 3291 (ν_{N-H}), 2158 ($\nu_{C\equiv C}$), 1732 ($\nu_{C=O}$). HRMS (ESI): m/z calcd for $[M(C_{90}H_{90}N_2O_6Si_2)+Cl]^-$, 1385.6026; found 1385.5982.

(*S*,*S*)-**2b-OMe.** 91% yield. Mp: 211 °C (dec.). $[\alpha]_D^{20}$ 241 (*c* = 0.2 in $CHCl_3$). ^{1}H NMR (500 MHz, $CDCl_3$, 25 °C, 5.2 mM): δ 7.73 (d, *J* = 8.5 Hz, 4H, ArH), 7.55 (d, *J* = 8.1 Hz, 8H, ArH), 7.49 (d, *J* = 8.4 Hz, 4H, ArH), 7.40 (s, 2H, ArH), 7.38 (s, 2H, ArH), 7.36 (d, *J* = 8.4 Hz, 4H, ArH), 7.31 (d, *J* = 8.4 Hz, 4H, ArH), 6.80 (d, *J* = 7.5 Hz, 2H, NH), 4.08–3.93 (m, 2H, NCH), 3.39 (s, 6H, CH_3CO_2), 2.41 (t, *J* = 7.1 Hz, 4H, $C\equiv CCH_2$), 2.31–2.16 (m, 2H, CH), 1.95–1.76 (m, 2H, CH), 1.65–1.54 (m, 6H, CH_2), 1.50–1.27 (m, 18H, CH and CH_2), 0.89 (t, *J* = 7.0 Hz, 6H, CH_3), 0.26 (s, 18H, $SiCH_3$). ^{13}C NMR (125 MHz, $CDCl_3$, 25 °C, 5.2 mM): δ 169.31, 167.67, 140.29, 140.11, 140.09, 140.02, 133.63, 132.12, 131.98, 131.94, 131.90 131.85, 131.56, 128.54, 128.33, 127.14, 126.69, 125.83, 122.76, 122.42, 104.86, 95.30, 93.03, 91.59, 89.57, 79.64, 54.87, 52.17, 32.55, 31.48, 28.77, 28.70, 24.93, 22.68, 19.60, 14.20, 0.10. IR (film, cm^{-1}): 3291 (ν_{N-H}), 2158 ($\nu_{C\equiv C}$), 1733 ($\nu_{C=O}$). HRMS (ESI): m/z calcd for $[M(C_{90}H_{90}N_2O_6Si_2)+Cl]^-$, 1385.6026; found 1385.5963.

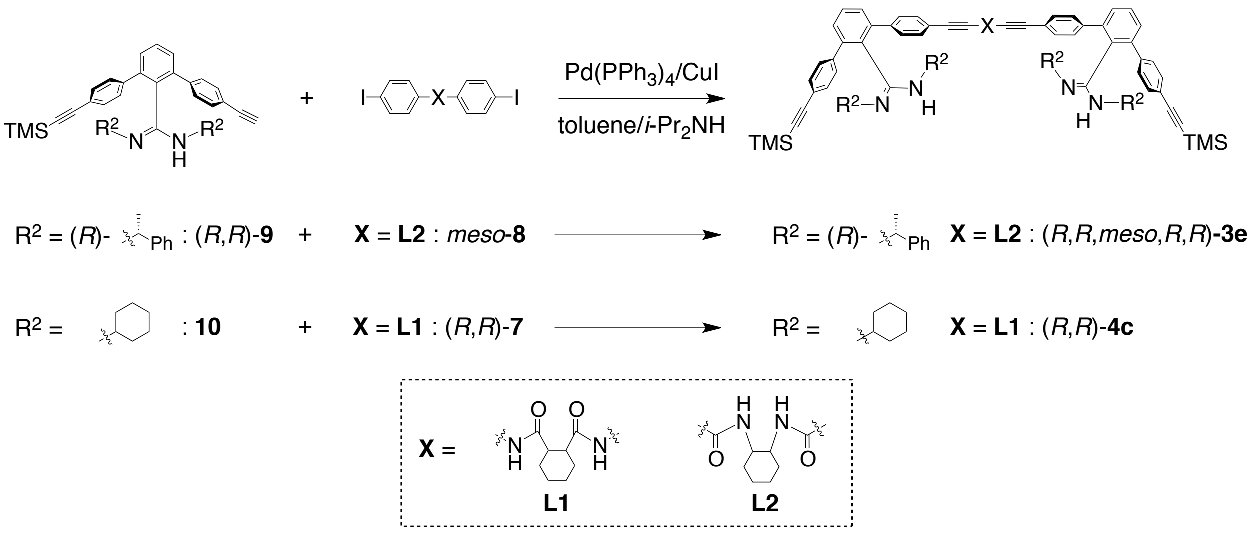

***meso*-8.** Dimethylamino pyridine (35.0 mg, 0.286 mmol) and 1-ethyl-3-(3-dimethylaminopropyl)carbodiimide hydrochloride (647 mg, 3.38 mmol) were added to a suspension of *p*-iodobenzoic acid (745 mg, 3.00 mmol) and *cis*-1,2-cyclohexanediamine (153 mg, 1.34 mmol) in dichloromethane (10 mL) at 0 °C. After the mixture was stirred at ambient temperature for 8.5 h, the obtained precipitate was collected and washed with water and $CHCl_3$ to afford *meso*-**8** (0.73 g, 98% yield) as a white solid. This was used in the next step without further purification. Mp: 211–213 °C. ^{1}H NMR (500 MHz, DMSO-d_6, 25 °C): δ 8.02 (d, J = 7.2 Hz, 2H, NH), 7.83 (d, J = 6.7 Hz, 4H, ArH), 7.58 (d, J = 6.7 Hz, 4H, ArH), 4.23–4.17 (m, 2H, NCH), 1.91–1.82 (m, 2H, CH), 1.72–1.63 (m, 2H, CH), 1.62–1.54 (m, 2H, CH), 1.44–1.35 (m, 2H, CH). ^{13}C NMR (125 MHz, DMSO-d_6, 25 °C): δ 165.87, 136.99, 134.39, 129.43, 98.47, 49.20, 27.61, 21.97. IR (KBr, cm^{-1}): 3303 (ν_{N-H}), 1637 ($\nu_{C=O}$), 1536 (ν_{C-N}). Anal. Calcd for $C_{20}H_{20}I_2N_2O_2$: C, 41.83; H, 3.51; N, 4.88. Found: C, 41.93; H, 3.41; N, 4.86.

General Procedures for the Preparation of Amidine Dimers. A typical experimental procedure is described below. CuI (3.60 mg, 0.0189 mmol) was added to a solution of monoethynyl amidine (*R*,*R*)-**9** (126 mg, 0.210 mmol), *meso*-**8** (60.0 mg, 0.105 mmol), and $Pd(PPh_3)_4$ (15.6 mg, 0.0135 mmol) in toluene-diisopropylamine (8/2 (v/v), 6.0 mL). After the mixture was stirred at 65 °C for 11 h under nitrogen, the solvents were evaporated to dryness. The residue was then purified by column chromatography (NH-SiO_2, *n*-hexane/EtOAc) and Bio-Beads (SX-1, $CHCl_3$) to afford (*R*,*R*,*meso*,*R*,*R*)-**3e** (42 mg, 27% yield) as a pale yellow solid. In the same way, (*R*,*R*)-**4c** was prepared.

***(R,R,meso,R,R)*-3e.** 27% yield. Mp: 169–171 °C. $[\alpha]_D^{20}$ –143 (*c* = 0.1 in $CHCl_3$). ^{1}H NMR (500 MHz, $CDCl_3$, 25 °C, as (*R,R,meso,R,R*)-**3e**·$(CH_3CO_2H)_2$): δ 13.50 (br s, 4H, NH), 7.86 (d, *J* = 8.45 Hz, 4H, ArH), 7.75 (t, *J* = 7.75 Hz, 2H, ArH), 7.62 (d, *J* = 7.85 Hz, 4H, ArH), 7.33–7.20 (m, 24H, ArH), 7.09–7.03 (m, 8H, ArH), 6.74 (d, *J* = 8.10 Hz, 4H, ArH), 6.68 (d, *J* = 8.20 Hz, 4H, ArH), 4.33 (m, 2H, C*H*NH), 3.92 (m, 4H, CHN), 2.12 (s, CH_3CO_2), 1.76–1.58 (m, 8H, CH), 0.73 (d, *J* = 5.50 Hz, 12H, CH_3CHN), 0.25 (s, 18H, $SiCH_3$). ^{13}C NMR (125 MHz, $CDCl_3$, 25 °C, as (*R,R,meso,R,R*)-**3e** ·$(CH_3CO_2H)_2$): δ 179.43, 167.72, 162.64, 143.05, 142.97, 141.79, 141.65, 138.43, 138.20, 133.86, 132.38, 132.13, 132.00, 131.95, 130.75, 130.65, 129.18, 129.15, 128.82, 128.60, 128.06, 127.32, 126.74, 126.70, 126.59, 123.46, 123.09, 122.76, 104.34, 96.19, 91.21, 90.31, 55.56, 52.51, 28.60, 24.55, 22.45, 0.05. IR (KBr, cm^{-1}): 3433 (ν_{N-H}), 2156 ($\nu_{C\equiv C}$), 1638 ($\nu_{C=O}$). HRMS (ESI): m/z calcd for $[M(C_{104}H_{98}N_6O_2Si_2)+H]^+$, 1519.7368; found 1519.7365.

***(R,R)*-4c.** 29% yield. Mp: 177–179 °C. $[\alpha]_D^{20}$ –139 (*c* = 0.1 in $CHCl_3$). ^{1}H NMR (500 MHz, $CDCl_3$, 25 °C, as (*R,R*)-**4c**·$(CH_3CO_2H)_2$): δ 12.00 (br s, 4H, NH), 8.06 (br s, 2H, NH), 7.71 (t, *J* = 8.0 Hz, 2H, ArH), 7.76–7.53 (m, 12H, ArH), 7.52–7.48 (m, 12H, ArH), 7.45 (d, *J* = 8.5 Hz, 4H, ArH), 2.72–2.60 (m, 6H, C*H*NH, C*H*CO), 2.04 (s, CH_3CO_2), 1.91–1.85 (m, 2H, CH), 1.72–1.62 (m, 2H, CH), 1.57–1.49 (m, 8H, CH), 1.42–1.34 (m, 6H, CH), 1.28–1.17 (m, 10H, CH), 1.06–0.95 (m, 4H, CH), 0.90–0.72 (m, 16H, CH), 0.27 (s, 18H, $SiCH_3$). ^{13}C NMR (125 MHz, $CDCl_3$, 25 °C, as (*R,R*)-**4c**·$(CH_3CO_2H)_2$): δ 176.62, 173.88, 161.36, 140.95, 140.86, 138.68, 138.23, 138.13, 132.73, 132.57, 132.31, 131.89, 130.88, 130.80, 128.82, 128.70, 124.22, 123.94, 122.44, 119.91, 118.78, 104.19, 96.51, 91.27, 88.37, 54.71, 48.48, 32.43, 29.47, 25.40, 25.08, 24.57, 22.33, 0.05. IR (KBr, cm^{-1}): 3431 (ν_{N-H}), 2157 ($\nu_{C\equiv C}$), 1637 ($\nu_{C=O}$). HRMS (ESI): m/z calcd for $[M(C_{96}H_{106}N_6O_2Si_2)+H]^+$, 1431.7994; found 1431.7945.

3-4. Theoretical Studies on the Structures of the Homochiral Duplexes ((*S*,*S*)-1a)$_2$ and ((*S*,*S*)-2a)$_2$, Heterochiral Duplexes (*S*,*S*)-1a•(*R*,*R*)-1a and (*S*,*S*)-2a•(*R*,*R*)-2a, and Hetero-Sequenced Duplex (*S*,*S*)-1a•(*S*,*S*)-2a

The molecular modeling was performed on a Windows XP PC with the ArgusLab software[2]. The initial structures, in which the pendant 1-octynyl groups are replaced by hydrogen atoms, were constructed based on the crystal structure of (*S*,*S*)-**1a'** or (*S*,*S*)-**2a'**. The initial models were then fully optimized by the semi-empirical molecular orbital (MO) calculations (PM6 method[3] in MOPAC2012[4]) and further DFT calculations at the B3LYP/6-31G* level using *Gaussian 09* software (Gaussian, Inc., Pittsburgh, PA)[5]. Computer resources for the DFT calculations were provided by the Information Technology Center of Nagoya University. The resultant energy-minimized structures with their total energies are depicted in Supplementary Fig. 21.

3-5. Theoretical Formulae (Fig. 3b)

When the concentrations of (*S*,*S*)-**1a** and (*R*,*R*)-**1a** are s_0 and r_0 ($s_0 + r_0 = 0.50$ mM $= a$, $s_0 \geqq r_0$), respectively, the enantiomeric excess x ($0 \leq x \leq 1$) is given by the following equation.

$$x = (s_0 - r_0)/(s_0 + r_0) = (s_0 - r_0)/a \qquad (1)$$

then s_0 and r_0 are expressed as

$$s_0 = a(1 + x)/2 \qquad (2)$$

$$r_0 = a(1 - x)/2 \qquad (3)$$

(*S*,*S*)-**1a** and (*R*,*R*)-**1a** are assumed to exist as a homochiral- or heterochiral-duplex because their dimerization constants are extremely high (over 10^5 M^{-1}) and then,

$$s_0 = 2[SS] + [RS] \qquad (4)$$

$$r_0 = 2[RR] + [RS] \qquad (5)$$

where [*SS*], [*RR*], and [*RS*] are the concentrations of homochiral duplexes ((*S*,*S*)-**1a**)$_2$ and ((*R*,*R*)-**1a**)$_2$ and a heterochiral duplex (*R*,*R*)-**1a**·(*S*,*S*)-**1a**, respectively. Therefore, [*RS*] and [*RR*] are expressed as

$$[RS] = s_0 - 2[SS] \qquad (6)$$

$$[RR] = (r_0 - s_0)/2 + [SS] \qquad (7)$$

When the dimerization constants for homochiral duplexes (*SS* and *RR*) and a heterochiral duplex (*RS*) are K_{Homo} and K_{Hereto}, respectively, the equilibrium constant (K) is given by the following equation.

$$RR + SS \rightleftharpoons 2RS$$

$$K = [RS]^2/[RR][SS] \qquad (8)$$

$$= (K_{\mathrm{Hereto}} / K_{\mathrm{Homo}})^2 \qquad (9)$$

From eqs (6) – (8),

$$K = (a^2(1 + x)^2 - 8a(1 + x)[SS] + 16[SS]^2) / (4[SS]^2 - 2ax[SS])$$

therefore

$$(4K - 16)[SS]^2 - (2Kx - 8x - 8)a[SS] - a^2(1 + x)^2 = 0$$

then

$$[SS] = [(Kx - 4x - 4) - ((K^2 - 4K)x^2 + 4K)^{0.5}]a/4(K - 4) \qquad (10)$$

Given that y = ([homochiral duplex] – [heterochiral duplex]) / ([homochiral duplex] + [heterochiral duplex]) and then,

$$y = ([SS] + [RR] - [RS]) / ([SS] + [RR] + [RS])$$

$$= 8[SS]/a - 2x - 1 \qquad (11)$$

By using eq (10),

$$y = 2[(Kx - 4x - 4) - ((K^2 - 4K)x^2 + 4K)^{0.5}]/(K - 4) - 2x - 1 \qquad (12)$$

From Fig. 3a (0% e.e.) ($x = 0$), *rac*-**1a** forms homochiral and heterochiral duplexes in 2 : 1 ratio.

Therefore, eqs (11) and (12) are written by the following equations, respectively,

$$y = (2 - 1) / (2 + 1)$$

$$= 1/3 \qquad (13)$$

$$y = 2(2K^{0.5} - 4) / (K - 4) - 1 \qquad (14)$$

From eqs (13) and (14),

$$(K - 1)(K - 4) = 0$$

On the basis of the experimental results (Fig. 3a), $K \neq 4$ and then,

$$K = (K_{\mathrm{Hereto}} / K_{\mathrm{Homo}})^2 = 1 \qquad (15)$$

Therefore,

$$K_{H\mathrm{ereto}} = K_{\mathrm{Homo}}$$

By using eq (15), eq (12) is expressed as

$$y = [-2(4 - 3x^2)^{0.5} + 5]/3$$

(16)

y is also expressed as

$$y = Y/100 = [-2(4 - 3(X/100)^2)^{0.5} + 5]/3 \qquad (17)$$

where X and Y are the % e.e. of **1a** and the % d.e. of $\mathbf{1a}_2$, respectively. Therefore, Y is expressed as

$$Y = 100[-2(4 - 3(X/100)^2)^{0.5} + 5]/3 \qquad (A)$$

3-6. X-ray Crystallographic Data

Crystallographic Data of (*S*,*S*)-**1a'.** X-ray diffraction data set for (*S*,*S*)-**1a'** was collected on a Rigaku Saturn 724+ CCD diffractometer with Mo Kα radiation (λ = 0.71075 Å) at 103 K. Single crystals of (*S*,*S*)-**1a'** [$C_{158}H_{140}N_4O_{12}Si_4$, MW = 2399.10] suitable for X-ray analysis were obtained by the slow diffusion of acetonitrile into a toluene solution of (*S*,*S*)-**1a'**, and a single colorless crystal with dimensions 0.15 × 0.12 × 0.04 mm^3 was selected for intensity measurements. The unit cell was orthorhombic with the space group $P2_12_12_1$. Lattice constants with Z = 4, ρ_{calcd} = 1.146 g cm^{-3}, $\mu(Mo_{K\alpha})$ = 0.104 mm^{-1}, $F(000)$ = 5,072, $2\theta_{max}$ = 54.97° were a = 15.990(2) Å, b = 28.164(4) Å, c = 30.875(4) Å, and V = 13,905(3) $Å^3$. A total of 104,237 reflections was collected, of which 27,226 reflections were independent (R_{int} = 0.0515). The structure was refined to final R_1 = 0.1342 and wR_2 = 0.3443 for 24,265 data [I>2σ(I)] with 1,613 parameters, and R_1 = 0.1431 and wR_2 = 0.3520 for all data, GOF = 1.125, and residual electron density max/min = 1.641/−1.005 e $Å^{-3}$. The ORTEP drawing is shown in Supplementary Fig. 23, and crystal data and structure refinement are listed in Supplementary Table 1.

Data collection and processing were conducted using the Rigaku CrystalClear software package[6]. The structure was solved by direct methods using Sir2004[7] and refined by full-matrix least squares methods on F^2 with SHELXL-97 program[8,9] using Yadokari-XG 2009[10,11]. All non-hydrogen atoms were refined anisotropically. All hydrogen atoms were calculated geometrically and refined using the riding model. Crystallographic data have been deposited at the CCDC (12 Union Road, Cambridge CB2 1EZ, UK) and copies can be obtained on request, free of charge, by quoting the publication citation and the deposition number 1036590 (For x-ray data see Supplementary Data 1).

Crystallographic Data of (*S*,*S*)-**2a'**. X-ray diffraction data set for (*S*,*S*)-**2a'** was collected on a Rigaku Saturn 724+ CCD diffractometer with Mo Kα radiation (λ = 0.71075 Å) at 103 K. Single crystals of (*S*,*S*)-**2a'** [$C_{153}H_{116}N_4O_{12}$, MW = 2202.50] suitable for X-ray analysis were obtained by the slow diffusion of acetonitrile into a toluene solution of (*S*,*S*)-**2a'**, and a single colorless crystal with dimensions 0.20 × 0.06 × 0.02 mm^3 was selected for intensity measurements. The unit cell was monoclinic with the space group $P2_1$. Lattice constants with Z = 2, ρ_{calcd} = 1.237 g cm^{-3}, $\mu(Mo_{K\alpha})$ = 0.078 mm^{-1}, $F(000)$ = 2,316, $2\theta_{max}$ = 54.96° were a = 12.650(5) Å, b = 30.179(11) Å, c = 16.608(6) Å, and V = 5914(4) $Å^3$. A total of 43,813 reflections was collected, of which 18,248 reflections were independent (R_{int} = 0.0616). The structure was refined to final R_1 = 0.1239 and wR_2 = 0.2942 for 12,226 data [I>2σ(I)] with 1,603 parameters, and R_1 = 0.1630 and wR_2 = 0.3296 for all data, GOF = 1.149, and residual electron density max/min = 0.333/-0.341 e $Å^{-3}$. The ORTEP drawing is shown in Supplementary Fig. 24, and crystal data and structure refinement are listed in Supplementary Table 2.

Data collection and processing were conducted using the Rigaku CrystalClear software package[6]. The structure was solved by direct methods using Sir2004[7] and refined by full-matrix least squares methods on F^2 with SHELXL-97 program[8,9] using Yadokari-XG 2009[10,11]. All non-hydrogen atoms were refined anisotropically. All hydrogen atoms were calculated geometrically and refined using the riding model. Crystallographic data have been deposited at the CCDC (12 Union Road, Cambridge CB2 1EZ, UK) and copies can be obtained on request,

free of charge, by quoting the publication citation and the deposition number 1036591 (For x-ray data see Supplementary Data 2). The crystallographic data of (*S*,*S*)-**2a'** contain some short inter H···H contacts between the hydrogen atoms of solvated toluene molecules and disordered phenylene groups of terphenyl groups, which gave rise to six level-A and level-B alerts within PLATON/CheckCIF. We attempted to resolve these problems by refining the related atoms with partial occupancies, but complete refinement of these related atoms was unsuccessful.

3-7. Supporting Data for Complementary Duplex Formations.

General Procedures for Simulating CD Spectra for a Mixture of $\mathbf{3a}_{\mathbf{CONH}}$ and *rac*-$\mathbf{2a}_{\mathbf{CONH}}$

A typical simulation procedure is described below. The simulated CD spectrum (CD_{Sim}) for a mixture of (*R*,*R*,*R*,*R*,*R*,*R*)-**3a** and two equivalents of *rac*-**2a** was obtained by the following equations (1)–(3),

$$CD_{Sim} = v{\cdot}CD_{\mathbf{3a}\cdot(R,R)\text{-}\mathbf{2a}} + w{\cdot}CD_{\mathbf{3a}\cdot(S,S)\text{-}\mathbf{2a}} + x{\cdot}CD_{(R,R)\text{-}\mathbf{2a}} + y{\cdot}CD_{(S,S)\text{-}\mathbf{2a}} + z{\cdot}CD_{\mathbf{3a}} \quad (1)$$

$$v + w + x + y + z = 2 \quad (2)$$

where v, w, x, y, and z are the relative molar ratio of (*R*,*R*,*R*,*R*,*R*,*R*)-**3a**·(*R*,*R*)-**2a**, (*R*,*R*,*R*,*R*,*R*,*R*)-**3a**·(*S*,*S*)-**2a**, (*R*,*R*)-**2a**, (*S*,*S*)-**2a**, and (*R*,*R*,*R*,*R*,*R*,*R*)-**3a** estimated by its ^{1}H NMR integral ratio, respectively (Supplementary Fig. 25), and $CD_{\mathbf{3a}\cdot(R,R)\text{-}\mathbf{2a}}$, $CD_{\mathbf{3a}\cdot(S,S)\text{-}\mathbf{2a}}$, $CD_{(R,R)\text{-}\mathbf{2a}}$, $CD_{(S,S)\text{-}\mathbf{2a}}$, and $CD_{\mathbf{3a}}$ are the CD spectra of separately prepared (*R*,*R*,*R*,*R*,*R*,*R*)-**3a**·(*R*,*R*)-**2a**, (*R*,*R*,*R*,*R*,*R*,*R*)-**3a**·(*S*,*S*)-**2a**, (*R*,*R*)-**2a**, (*S*,*S*)-**2a**, and (*R*,*R*,*R*,*R*,*R*,*R*)-**3a** measured in $CDCl_3$, respectively (Supplementary Fig. 26a). According to the experimentally obtained integral ratio ($v = y = 0.79$, $w = x = 0.21$, and $z = 0$), the CD_{Sim} in eq (1) is expressed as

$$CD_{Sim} = 0.79(CD_{\mathbf{3a}\cdot(R,R)\text{-}\mathbf{2a}} + CD_{(S,S)\text{-}\mathbf{2a}}) + 0.21(CD_{\mathbf{3a}\cdot(S,S)\text{-}\mathbf{2a}} + CD_{(R,R)\text{-}\mathbf{2a}}) \quad (3)$$

The simulated CD spectrum was almost identical to the observed CD (Supplementary Fig. 26b). In the same way, the simulated CD as well as absorption spectra between amidine dimers (**3a**–**4c**) and *rac*-**1a** or *rac*-**2a** were obtained.

We note that only the amidine dimers ($\mathbf{3b}_{\mathbf{CONH}}$, $\mathbf{3c}_{\mathbf{CONH}}$, and $\mathbf{3d}_{\mathbf{CONH}}$) were found to form complementary duplexes as well as a small amount of unknown complexes with $\mathbf{1a}_{\mathbf{NHCO}}$. Although the structures of the latter complexes could not be identified at the present time, the diastereoselectivity (% d.e.) of the main products, complementary duplexes, can be estimated with accuracy by their ^{1}H NMR spectra (Supplementary Figs 34, 42, 44, and 46). Therefore, the simulated CD spectra for a mixture of $\mathbf{3b}_{\mathbf{CONH}}$, $\mathbf{3c}_{\mathbf{CONH}}$, or $\mathbf{3d}_{\mathbf{CONH}}$ and $\mathbf{1a}_{\mathbf{NHCO}}$ were obtained according to eq (1) based on an assumption that CD contributions from the unknown complexes were negligible.

4. Supplementary References

1. Berova, N., Nakanishi, K. & Woody, R. W., Eds. *Circular Dichroism: Principles and Applications, 2nd ed.*, (Wiley-VCH, 2000).
2. Thompson, M. ArgusLab, Planaria Software LLC, Seattle, WA (1996).
3. Stewart, J. J. P. *J. Mol. Model.* **13**, 1173-1213 (2007).
4. Stewart, J. J. P**.** *MOPAC2012*, Stewart Computational Chemistry, Colorado Springs, CO, USA, http://openmopac.net/ (2012).
5. Gaussian 09, Revision D.01, Frisch, M. J., Trucks, G. W., Schlegel, H. B., Scuseria, G. E., Robb, M. A., Cheeseman, J. R., Scalmani, G., Barone, V., Mennucci, B., Petersson, G. A., Nakatsuji, H., Caricato, M., Li, X., Hratchian, H. P., Izmaylov, A. F., Bloino, J., Zheng, G., Sonnenberg, J. L., Hada, M., Ehara, M., Toyota, K., Fukuda, R., Hasegawa, J., Ishida, M., Nakajima, T., Honda, Y., Kitao, O., Nakai, H., Vreven, T., Montgomery, J. A., Jr., Peralta, J. E., Ogliaro, F., Bearpark, M., Heyd, J. J., Brothers, E., Kudin, K. N., Staroverov, V. N., Kobayashi, R., Normand, J., Raghavachari, K., Rendell, A., Burant, J. C., Iyengar, S. S., Tomasi, J., Cossi, M., Rega, N., Millam, N. J., Klene, M., Knox, J. E., Cross, J. B., Bakken, V., Adamo, C., Jaramillo, J., Gomperts, R., Stratmann, R. E., Yazyev, O., Austin, A. J., Cammi, R., Pomelli, C., Ochterski, J. W., Martin, R. L., Morokuma, K., Zakrzewski, V. G., Voth, G. A., Salvador, P., Dannenberg, J. J., Dapprich, S., Daniels, A. D., Farkas, Ö., Foresman, J. B., Ortiz, J. V., Cioslowski, J. & Fox, D. J. Gaussian, Inc., Wallingford CT (2009).
6. CrystalClear, Version 1.36; Molecular Structure Corporation: The Woodlands, TX, 2000 and Rigaku Corporation: Tokyo, Japan.
7. Burla, M. C., Caliandro, R., Camalli, M., Carrozzini, B., Cascarano, G. L., De Caro, L., Giacovazzo, C., Polidori, G. & Spagna, R. SIR2004: an improved tool for crystal structure determination and refinement. *J. Appl. Crystallogr.* **38**, 381–388 (2005).
8. Sheldrick, G. M. SHELXL-97: Program for the Refinement of Crystal Structures; University of Göttingen, Göttingen, Germany (1997).
9. Sheldrick, G. M. A short history of SHELX. *Acta Crystallogr.* **A64**, 112–122 (2008).
10. Wakita, K. *Yadokari-XG,* Program for Crystal Structure Analysis (2000).
11. Kabuto, C., Akine, S. & Kwon, E. Release of software（Yadokari-XG 2009）for crystal structure analyses. *J. Cryst. Soc. Jpn.* **51**, 218–224 (2009).
